# Bioorthogonal Activation of Protein Function through a retro-Cope/Cope Elimination Cascade

**DOI:** 10.1021/jacs.6c05497

**Published:** 2026-06-25

**Authors:** Surached Siriwongsup, Sanghyeon Lee, Yiming Guo, Conrad Wahl, Justin Kim

**Affiliations:** † Department of Cancer Biology, 1855 Dana-Farber Cancer Institute, Boston, Massachusetts 02215, United States; ‡ Department of Biological Chemistry and Molecular Pharmacology, Harvard Medical School, Boston, Massachusetts 02115, United States; § School of Chemistry and Biochemistry, 1372 Georgia Institute of Technology, Atlanta, Georgia 30332, United States

## Abstract

A bioorthogonal click-to-release reaction employing cyclooctynes and *N*,*N*-dialkylhydroxylamines is described. The reaction is characterized by a tandem retro-Cope/Cope elimination reaction sequence in which strain-promoted hydroamination of a cyclooctyne by a *N*,*N*-dialkylhydroxylamine reagent is relayed into Cope elimination of the resulting enamine *N*-oxide. β-Elimination of the *N*-hydroxyenamine product then results in bond cleavage. The reaction is regioselective, and the cleavage is directional. The primary hydroamination reaction exhibits second order rate constants up to 2 M^–1^s^–1^, and the ensuing elimination steps are not rate limiting up to millimolar levels of hydroxylamine. The transformation enables the rapid and complete cleavage of a chemical bond in biologically relevant settings using reagents with a small molecular footprint. We demonstrate the importance of reagent size in an application involving the chemical activation of protein function using hydroxylamines that are tuned for either rapid kinetics or constrained spaces. Access limitations to enzyme active sites are a major determinant of reagent choice in bioorthogonal cleavage reaction applications.

## Graphical abstract

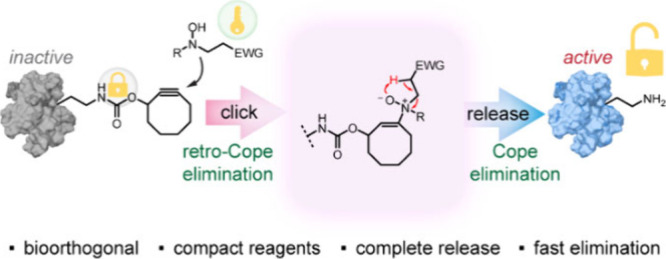

## Introduction

Over the past two decades, bioorthogonal chemistry has undergone rapid development, offering a myriad of technologies that have enabled users to interface with living systems. Early interest in the field spurred the development of associative reactions that serve to ligate chemical components together. Such click reactions facilitated the rapid assembly of chemical matter and vastly expanded the chemical diversity that could be introduced into biological systems. The field of bioorthogonal cleavage reactions (BCRs) has experienced slower maturation by comparison; however, the field has also undergone significant advances in recent years, producing powerful methods and applications.^1–3^ A major focus of BCR chemistry has been the functional control of biological substrates using small-molecule effectors. In these applications, the reactivity of biomolecules is masked with a chemically responsive element, and at a desired time, a small molecule is used to trigger cleavage and restore the function (Figure 1A).

**Figure 1:**
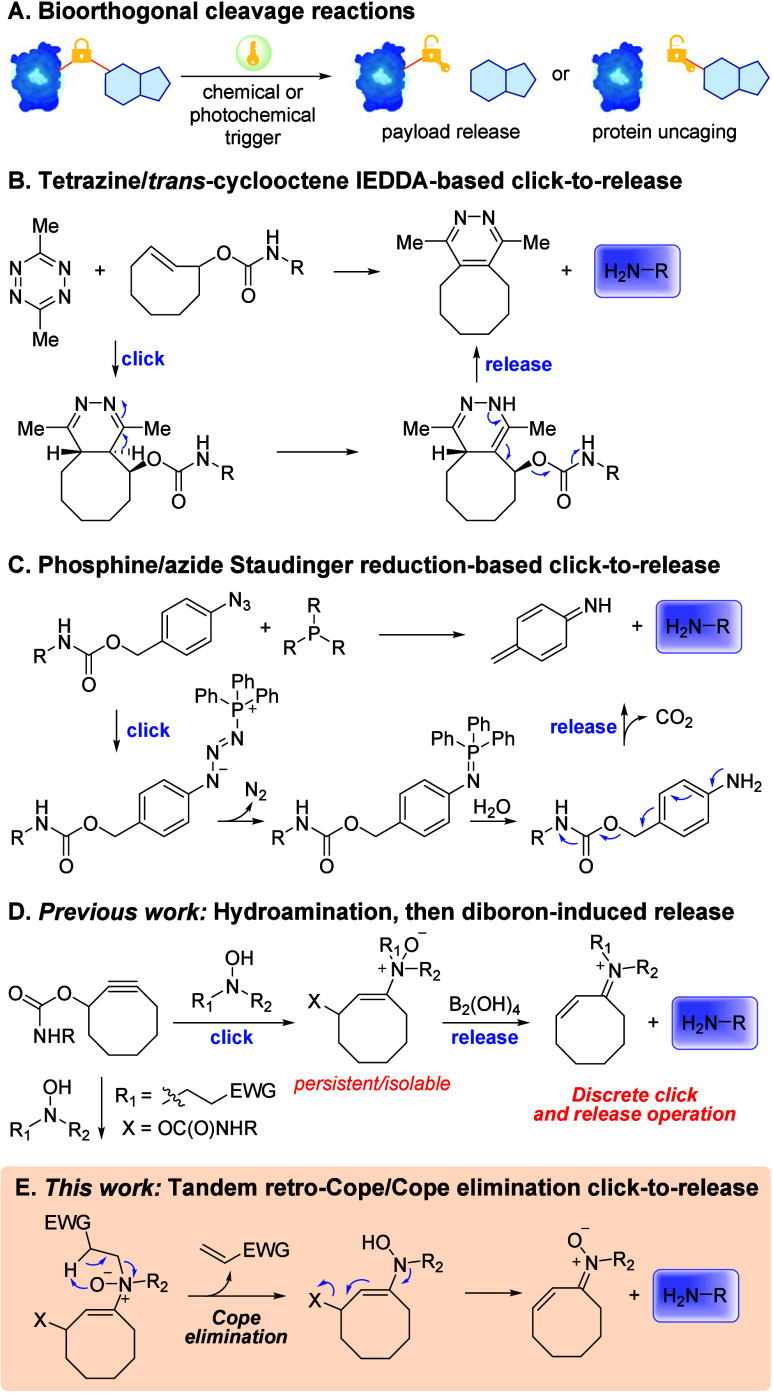
Representative bioorthogonal bond cleavage reactions. (A) Scheme illustrating the general concept of bioorthogonal cleavage reactions (BCRs) and their applications. (B) Tetrazine/*trans*-cyclooctene inverse electron demand Diels–Alder-mediated click-to-release bioorthogonal cleavage reaction. (C) Phosphine-mediated Staudinger reduction. (D) Our previous work on a chemically revertible bioconjugation strategy involving strain-promoted cyclooctyne hydroamination followed by diboron-mediated cleavage. (E) Strain-promoted, tandem retro-Cope/Cope elimination for click-to-release.

BCRs are to bioorganic chemistry what protecting group strategies have been to small molecule chemistry. Just as there is a plethora of protecting groups available to organic chemists, there is a growing collection of BCRs available to chemical biologists (Figure 1B,C). Transition metal-catalyzed deprotection reactions have been described as early as 2006 when Meggers and co-workers reported the first ruthenium-mediated deallylation of allylcarbamates in live cells.^4^ Other transition metal catalysts have found their place in the BCR compendium, including palladium,^5^ copper,^6^ gold,^7^ and platinum.^8^ Beyond transition metals, deprotection strategies have drawn inspiration from several cleavage reactions derived from a corresponding associative bioorthogonal reaction. Adapting the classical bioorthogonal Staudinger ligation^9^ and the inverse electron demand Diels–Alder reaction between *trans*-cyclooctene (TCO) and tetrazine (Tz) for dissociative purposes,^10^ Robillard and co-workers introduced the concept of click-to-release reactions, which relay an initial bioorthogonal reaction into a spontaneous bond cleavage step. Recent examples of bioorthogonal click-to-release mechanisms involve functional groups such as iminosydnones,^11,12^ cycloketones,^13^ isonitriles,^14^ nitrones,^15^ and sulfones.^16^

Applications of BCRs center around the ability to control biological processes through bond cleavage events. Notwithstanding developments in prodrug development, where BCRs drive the ability of various synthetically designed modalities from small molecules to biologics to regain their pharmacological properties in a highly controlled manner, biomolecules endogenous to living systems, such as proteins, can also be made to leverage BCRs. In particular, conditional control over protein function through bond cleavage offers a powerful method to manipulate and study biological processes.^1^ The strategy makes use of the ability to mask the functions of target proteins by chemical protecting groups. Most commonly, an amino acid residue essential for activity of a protein of interest (POI) is caged by an inducibly labile unnatural amino acid (UAA) via genetic code expansion technologies.

Such UAA-based uncaging strategies have long been the domain of photochemical-activation methods. Chin, Chen, Deiters, and others have used this strategy to great effect to cage lysine and cysteine residues on proteins as varied as kinases (e.g., MEK and LCK),^17,18^ T7 RNA polymerase,^19^ and Cas9 nuclease.^20^ The impact of photochemical deprotection strategies is profound, yet challenges exist in terms of cytotoxicity, tissue penetration, reagent stability, and applicability in certain in vivo systems where optical transparency is limited or systemic distribution is required.^2^ Much progress has been made with respect to improving issues such as phototoxicity, exemplified by work by Deiters and co-workers in designing coumarin-based photocaged lysine residues;^21–23^ however, an alternative to placing proteins under photochemical control is to employ chemically responsive switches.^24^

Small-molecule-mediated activation presents an attractive complement to photochemistry-based methods, particularly for applications dealing with large volumes or amorphous geometries. Examples of adapting chemically driven BCRs for protein activation have been well documented. In 2014, Chen and co-workers leveraged the bioorthogonal cleavage reaction between TCO and Tz for protein activation in live cells.^25^ In this case, TCO-caged lysine was incorporated into proteins and then subsequently uncaged by dimethyltetrazine (Figure 1B). Later, Deiters and co-workers revisited the bioorthogonal Staudinger reduction and adapted the reaction for protein activation.^26^ Here, azidobenzyl carbamate-caged lysine was introduced into proteins, and various trisubstituted phosphine reagents were employed to enact bond cleavage and subsequent protein activation (Figure 1C). This work has been highly effective even for in vivo applications in zebrafish and advocates for the broad adoption of chemically activatable systems.^24^ The field is poised to benefit from the additional complementary methods.

Despite the growing toolbox of BCRs developed for protein activation, key challenges persist with respect to reaction completion, biocompatibility, and the size of the reacting components. The difficulty of achieving complete uncaging in the click-to-release reaction involving TCO/Tz due to unproductive tautomerization and subsequent formation of dead-end products has been well documented.^27–30^ This problem can be addressed by modulating the substituents on either the tetrazine or *trans*-cyclooctene moiety but not without a trade-off in the size of the chemical components and stability in water.^27–30^ With regard to the Staudinger reduction, Chen and co-workers reported that azidobenzyl-caged amino acids are metabolically labile and undergo spontaneous uncaging in *E. coli*, exhibiting premature release in 27% of expressed protein.^31^ Hence, chemically activatable systems that function well without background activation would be proven useful. Furthermore, caging protein function is predicated on the active site accommodation of not only the protecting group but also the small-molecule trigger. While recent advances have optimized bioorthogonal reagents for kinetics or conversion, these modifications often impose severe steric burden. Size is a critical, if not the most important, consideration in protein/enzyme activation applications; reagents that cannot engage cannot uncage. This steric compatibility serves as a gatekeeping criterion that, without fulfillment, renders any method moot regardless of its kinetics or conversion efficiency. While powerful, the Staudinger reduction, for example, requires accommodation of azidobenzyltriphenylphosphine adducts in a protein active site. Reagent combinations requiring a smaller molecular footprint would increase the reach of these types of chemical activation schemes (vide infra). Finally, the development of additional BCR chemistry that can be used to cage a broader range of amino acids is important. Recently, amino acids such as tyrosine and tryptophan have been leveraged for chemical uncaging, as Chen et al. demonstrated.^32,33^

## Results and Discussion

Bioorthogonal cleavage strategies favor a priority of parameters distinct from those used for associative transformations. First, a major determinant of the utility of an associative click reaction is its second-order rate constant. Slower reaction rates engender use of higher concentrations of tag, probe, fluorophore, and affinity handle; higher concentrations entail higher nonspecific background labeling and erosion of signal-to-noise, a principal metric of data quality. In cleavage reactions, the chase reagent does not carry a detected signal and therefore does not contribute to background. Insofar as the chase reagent is neither toxic nor disruptive to the underlying biology at operationally relevant concentrations, the decoupling of reagent concentration from experimental observables broadens the range of reasonably practicable second-order reaction rate constants; slower reactions can be compensated for by higher reagent concentrations to a certain extent. Second, in click-to-release reactions, the output signal is tied to the secondary dissociation event, not the initial ligation event. The second-order rate constant governing the initial click event is the primary kinetic parameter if and only if the first step is rate-limiting; however, the secondary release step can be and is rate-determining in many applications (vide infra). Third, click-to-release reactions are, by definition, multistep cascade reaction sequences. Being circumspect to the potential for interception of intermediates postclick is of importance with clear ramifications on the completeness of bond cleavage and product yield.

Herein, we present a chemically controlled protein activation method based on a tandem retro-Cope/Cope elimination reaction sequence. We built on our group’s recent development of a bioorthogonal reaction between strained alkynes and *N*,*N*-dialkylhydroxylamines.^34^ Strained alkynes are well established bioorthogonal reaction components, and unlike hydroxylamine, *O*-alkylhydroxylamines, and *N*-monoalkylhydroxylamines, *N*,*N*-dialkylhydroxylamines are unreactive toward endogenous carbonyl species and therefore do not pose the same concerns of compatibility with biological systems.^35^ *N*,*N*-Dialkylhydroxylamine and strained alkynes selectively react to form stable and isolable enamine *N*-oxide linkages via the retro-Cope elimination reaction (Figure 1D). However, when hydroxylamines possessing an acidic β-proton are employed, we surmised that the subsequent enamine *N*-oxide adducts are unstable and will undergo further Cope elimination at rates comparable to existing bioorthogonal BCRs used in protein activation applications (Figure 1E).

During the development of a chemically revertible bioconjugation strategy using enamine *N*-oxides,^36^ we observed that bioorthogonal hydroamination of cyclooctynyl carbamate **1** and *N*-*tert*-butyl-*N*-methylhydroxylamine produces a transient enamine *N*-oxide adduct that spontaneously undergoes Cope elimination and then 1,4-elimination to release *p*-nitroaniline (**6**) in a tandem reaction cascade. In this sequence, the hydroamination step is directly relayed into enamine *N*-oxide fragmentation without intervention of a reductive stimulus such as tetrahydroxydiboron.^36,37^ Reflective of the rapid kinetics of the Cope elimination reaction and subsequent 1,4-elimination, the enamine *N*-oxide intermediate could not be observed under the reaction conditions. Given the overall simplicity of this dissociative process, we explored whether this process could be optimized for bioorthogonal click-to-release applications.

In the case of *N*-*tert*-butyl-*N*-methylhydroxylamine-cyclooctyne hydroamination, computational studies pointed to steric crowding around the tertiary amine *N*-oxide center as the primary factor driving the Cope elimination reaction.^35^ Steric pressures derived from both the α-alkyl substituent on the enamine *N*-oxide arising from hydroamination of an internal alkyne and the *tert*-butyl group on the *N*-oxide facilitated formation of the cyclic transition state, lowering the activation barrier to Cope elimination. While the stability of the enamine *N*-oxide adduct on cyclooctyne is sensitive to the steric bulk of the native hydroxylamine, the reaction also proved sensitive to the electronics of the eliminating alkyl substituent on the *N*-oxide. In fact, a convenient means for synthesizing hydroxylamines for the bioorthogonal retro-Cope elimination reaction involves the use of a tertiary amine oxidation-Cope elimination sequence.^38^

The Cope elimination reaction is accelerated by the presence of a β-electron withdrawing group on the eliminating arm of a tertiary amine *N*-oxide. While it was observed that increasing the acidity of the β-hydrogen would facilitate this reaction, it was unclear how such changes would impact the preceding hydroamination reaction between the precursor *N*,*N*-dialkylhydroxylamine and cyclooctyne. Understanding the kinetics of the tandem Cope elimination/retro-Cope elimination sequence and the role of substituent effects on the hydroxylamine component would prove critical.

To explore the use of electronically modified hydroxylamines in the tandem retro-Cope/Cope elimination sequence, *N*-cyanoethyl-*N*-methylhydroxylamine (**2**) was synthesized and its reaction with cyclooctynyl *p*-nitrophenyl carbamate **1** in deuterated methanol was monitored by ^1^H NMR (Figure 2A). The reaction was clean and complete with no noticeable side products, and >95% of the desired *p*-nitroaniline (**6**) was formed within 12 h (Figures 2B and S1). Intermediates **3** and **5** also did not accumulate except in the early stages of the reaction, indicating that Cope elimination and 1,4-elimination were fast enough under these conditions for the initial hydroamination step to be rate-determining (Figure 2C). The second-order rate constant was determined to be 0.123 M^–1^ s^–1^ in deuterated methanol, which was promising considering the likely presence of primary kinetic isotope effects in this experimental setup (Figure 2D).

**Figure 2:**
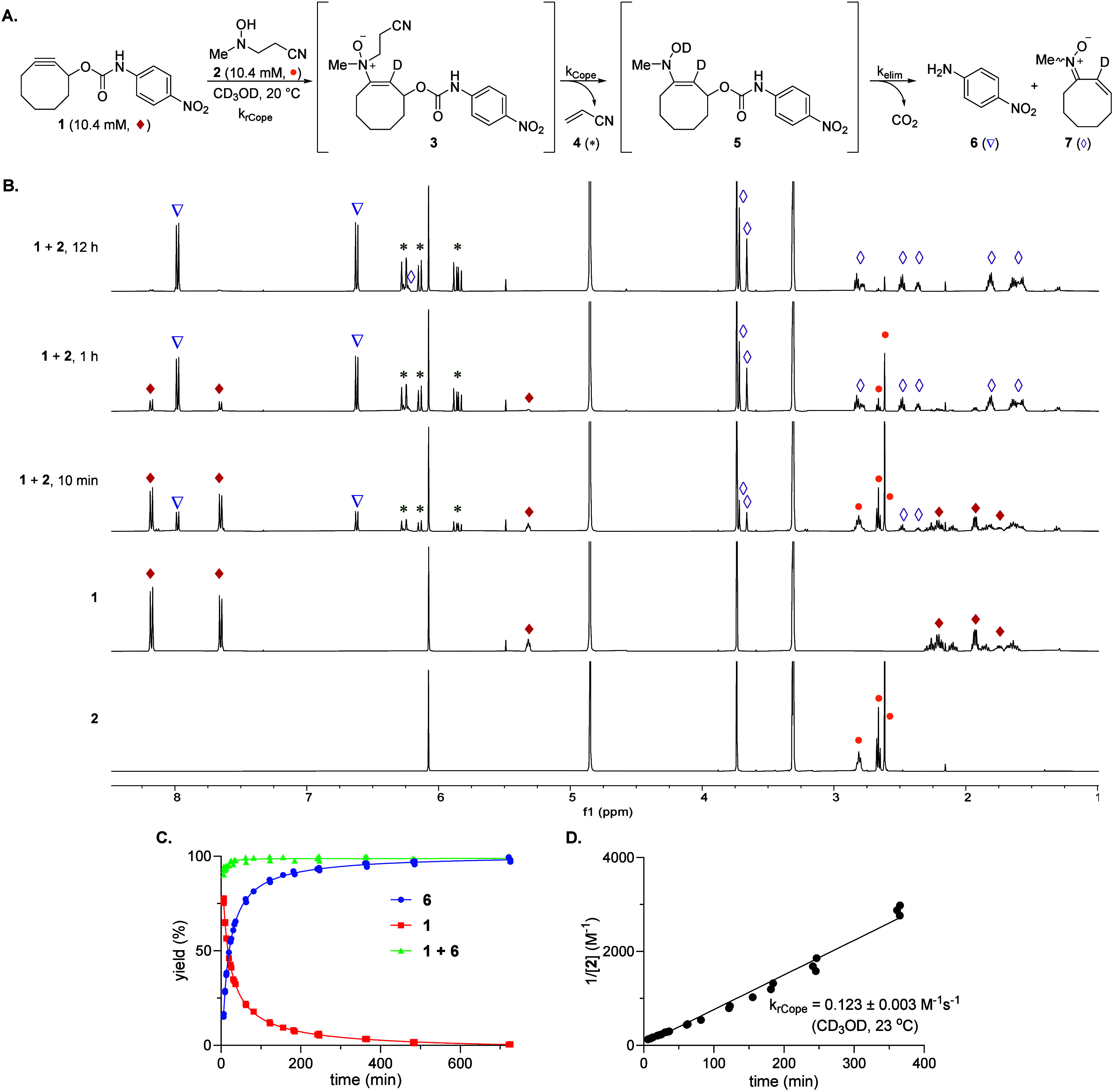
Tandem retro-Cope/Cope elimination reaction characterization and monitoring with NMR. (A) General scheme for the sequential retro-Cope elimination/Cope elimination of cyclooctynyl carbamate **1** and *N*-cyanoethyl-*N*-methylhydroxylamine (**2**). (B) NMR spectra of the reaction depicted in (A). (C) Plot showing percent NMR yield of product **6** release and starting material **1** depletion over time for the reaction depicted in (A). (D) Plot of 1/[hydroxylamine **2**] used to determine the second-order rate constant for the reaction depicted in (A).

To assess the importance of proton transfer and investigate the degree to which Cope elimination is integral to the success of the subsequent 1,4-elimination reaction, we repeated the experiment with *N*-allyl-*N*-methylhydroxylamine (**8**), whose adduct with cyclooctyne **9** is unable to undergo Cope elimination, instead undergoing [2,3]-Meisenheimer rearrangement to afford the *N*-allyloxyenamine intermediate **10** (Figure 3A).^39^ In this case, while hydroamination was even faster, the release of *p*-nitroaniline (**6**) was much slower with <40% released after 18 h (Figures 3B,C and S2). By 18 h, the *N*-allyloxyenamine intermediate **10** had accumulated and become the major species in solution. This species was not as conducive to 1,4-elimination as the *N*-hydroxyenamine counterpart. It is hypothesized that *N*-hydroxyenamines are able to undergo deprotonation in the transition state during 1,4-elimination, facilitating formation of the neutral nitrone dipole in contrast to the charged immonium species of the *N*-allyloxyenamine counterpart formed by [2,3]-Meisenheimer rearrangement. This key mode of charge stabilization is inaccessible to the latter. The preceding Cope elimination and formation of a hydroxylamine substructure are essential for rapid 1,4-elimination in this reaction cascade.

**Figure 3:**
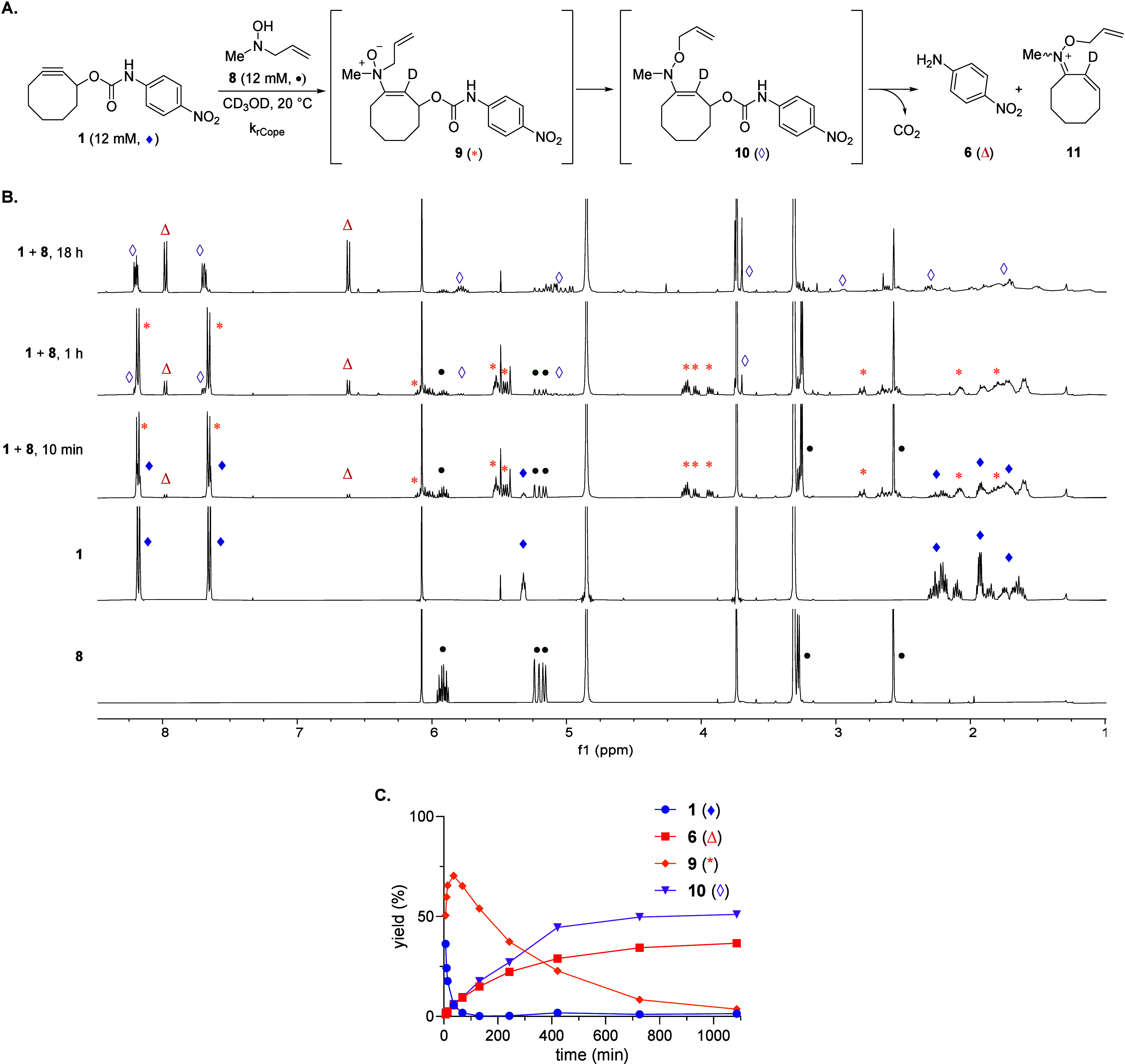
Effect of *O*-alkyl substituents on *N*-alkoxyenamine 1,4-elimination rates as an indication of the significance of the Cope elimination step. (A) General scheme for the retro-Cope elimination reaction between cyclooctyne **1** and *N*-allyl-*N*-methylhydroxylamine (**8**) followed by the tandem [2,3]-Meisenheimer rearrangement. (B) NMR spectra of the reaction depicted in (A). (C) Plot showing percent yield of species **1**, **6**, **9**, and **10** over time for the reaction depicted in (A).

We then continued to explore the effects of the β-activating group on the release rate, this time in aqueous conditions. We synthesized and then reacted the water-soluble cyclooctynyl carbamate ammonium salt **12** with hydroxylamines possessing various β-electron withdrawing groups (nitrile, sulfone, ester, amide; **2** and **14**–**16**) and monitored the reaction by ^1^H NMR (Figure 4A). Hydroamination rate constants (*k*_rCope_) were determined using initial rate data by following the disappearance of cyclooctyne **12** while overall reaction kinetics were evaluated by measuring amine product **13** formation. Among the substrates tested, the nitrile, sulfone, and ester-functionalized derivatives resulted in >95% release of amine **13** within 2 h (Figure 4B,C). While the dimethyl amide-substituted hydroxylamine **15** possessed the fastest hydroamination rate, Cope elimination was much slower and complete release was not attainable by 6 h. Additionally, symmetric hydroxylamines featuring a pair of alkyl substituents with β-electron withdrawing groups (**17**, **18**) were tested to address the possibility that product release is rate-limited by Cope elimination from the slower of the two diastereomeric enamine *N*-oxides potentially resulting from hydroamination. These experiments were met with mixed results with the dinitrile derivative **17** giving both slower hydroamination and slower release rates and the diester derivative **18** having a negligible effect on release kinetics when compared to their mononitrile or monoester counterparts, respectively. Interestingly, the initial rate of the retro-Cope elimination reaction was found to be negatively correlated with the Hammett constant (σ_meta_) of each substituent (Figure 4D). Together, the data indicate that, while hydroamination is favored by electron-rich hydroxylamines, Cope elimination is favored by electron-deficient groups on the eliminating arm of the hydroxylamine.

**Figure 4:**
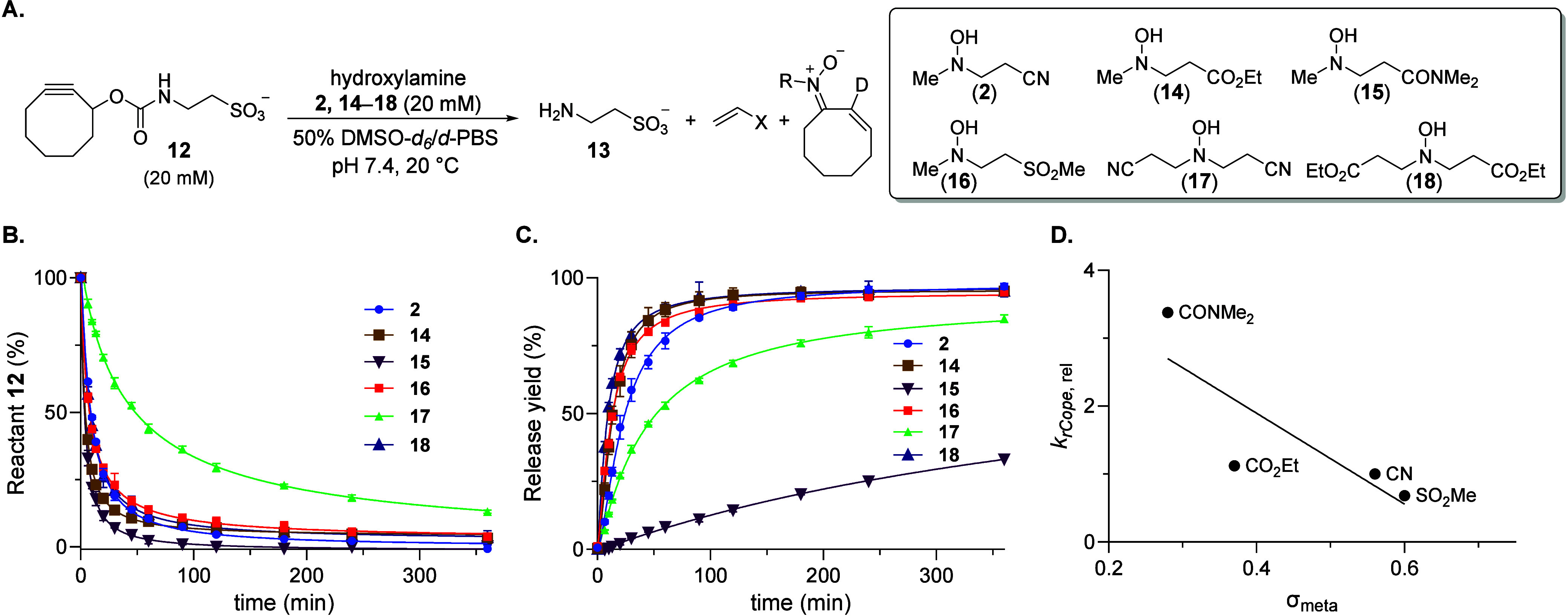
Hydroxylamine *N*-alkyl substituent effects on reaction rate. (A) General scheme for the sequential retro-Cope elimination/Cope elimination of water-soluble cyclooctynyl-carbamate **12** and hydroxylamines **2** and **14**–**18**. (B) Plot showing the remaining starting material **12** over time for the reaction depicted in (A). (C) Plot showing percent yield of release product **13** over time for the reaction depicted in (A). For (B) and (C), percentage was determined by ^1^H NMR peak integration. (D) Hammett constant (σ_meta_) of different β-activating substituents and the relative hydroamination rate (*k*_rCope, rel_) of hydroxylamines **2** and **14**–**16**.

To better understand the nuances of hydroxylamine structure toward reaction rate, we decided to expand the hydroxylamine substrate scope. Although NMR experiments provide detailed information about the fate of reaction intermediates and the reaction mechanism, here, the mechanism probed is subject to primary kinetic isotope effects. Thus, to reliably compare reaction rates between different hydroxylamines and determine rate constants for each, we implemented a fluorogenic assay as an alternative method to measure reaction kinetics. The assay employs the cyclooctynyl glycyl rhodamine **36** as a cleavage-responsive probe (Figure 5A). Upon reaction with various hydroxylamines at different concentrations (100 μM–100 mM), the fluorescence of the released glycyl rhodamine **37** was measured and normalized to the reference fluorescence obtained with the corresponding concentration of rhodamine fluorophore **37** (Figure 5B). Under pseudo-first-order conditions, the observed rate constant (*k*_obs_) was approximated by the rate of rhodamine release and determined at each hydroxylamine concentration (Figure 5C). Using cyanoethyl hydroxylamine **2** as the reference, the plot of *k*_obs_ versus the concentration of hydroxylamine revealed two distinct regimes across the concentration range tested. At lower concentrations (<1 mM of hydroxylamine **2**), the rate of rhodamine release (*k*_obs_) was proportional to hydroxylamine concentration, indicating second-order kinetics. Hence, the bimolecular retro-Cope elimination reaction governs the rate of release and the second-order rate constant (*k*_rCope_) is proportional to *k*_obs_. Beyond 1 mM hydroxylamine, *k*_obs_ starts to decouple from the hydroxylamine concentration with the plot of *k*_obs_ against the concentration of hydroxylamine **2** deviating from linearity. Beyond 50 mM hydroxylamine, the plot reaches a plateau, indicating a transition to first-order kinetics. Combined with the dependence of *k*_obs,max_ on hydroxylamine structure (vide infra), this suggests that the rate-limiting step becomes the subsequent Cope elimination, and the corresponding rate constant can be approximated by the maximum observed rate (*k*_obs,max_). Both values (*k*_rCope_ and *k*_obs,max_) were calculated and tabulated to compare the efficiencies of different hydroxylamine substrates (Figure 5D).

**Figure 5:**
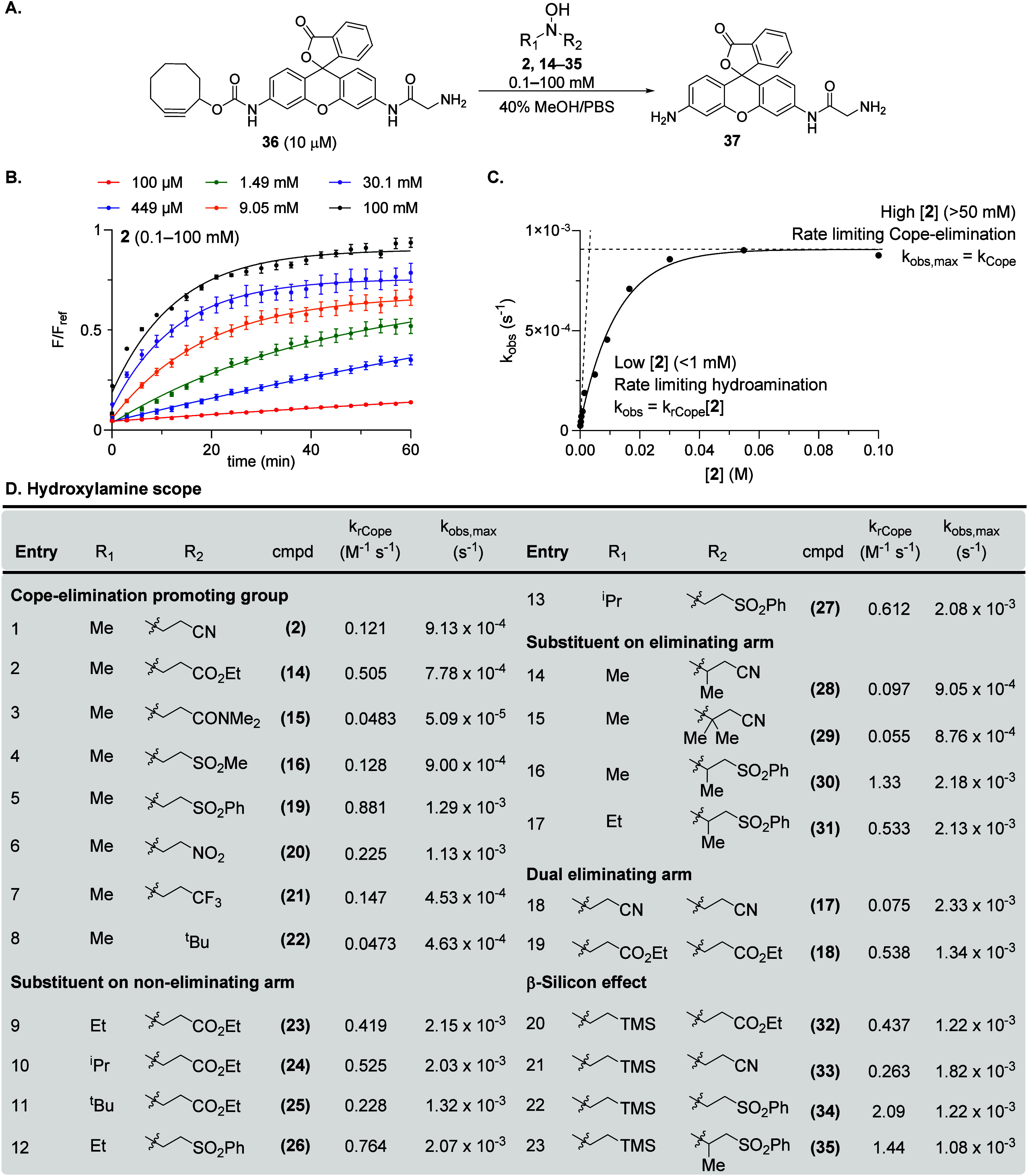
Fluorogenic assay to assess hydroxylamine kinetics. (A) Scheme for the fluorescence uncaging of cyclooctynyl rhodamine glycine **36** with hydroxylamines **2** and **14**–**35** to determine reaction kinetics. (B) Plot showing fluorescence uncaging over time from the reaction of cyclooctynyl rhodamine glycine **36** with reference cyanoethyl hydroxylamine **2**. For clarity, only six concentrations are shown, which include the lowest and highest concentrations tested. Error bars represent standard deviation from three replicates. (C) Plot of *k*_obs_ over [**2**]. Two regimes are demarcated; at [**2**] < 1 mM, *k*_obs_ is proportional to [**2**] and the hydroamination is rate-limiting. At concentrations above 50 mM, *k*_obs_ plateaus to a maximum value (*k*_obs,max_), indicating that Cope-elimination is now rate-limiting. (D). Table showing structures of all hydroxylamines tested in the fluorogenic assay with calculated values for the second-order rate constants (*k*_rCope_) and maximum observed rate (*k*_obs,max_).

We were able to rapidly expand the hydroxylamine substrate scope in support of our mechanistic investigations because of the ease of hydroxylamine derivatization (Scheme 1). Most *N*,*N*-dialkylhydroxylamine reagents employed in this report were synthesized through a single step aza-Michael addition of a hydroxylamine hydrochloride salt into an alkene acceptor with a β-electron withdrawing group, a prerequisite for a majority of substrates under consideration (Scheme 1A). For those inaccessible by conjugate addition, such as *N*-*tert*-butyl-*N*-methylhydroxylamine (**22**), reagents could be obtained by direct alkylation of an alkyl iodide (Scheme 1B). Finally, 2-(trimethylsilyl)­ethylhydroxylamine derivatives could be synthesized by single or double conjugate addition of 2-(trimethylsilyl)­amine hydrochloride into unencumbered or sterically hindered Michael acceptors, respectively. The former was oxidized with peracetic acid to afford the desired hydroxylamine upon Cope elimination while the latter was oxidized by benzoyl peroxide and then transesterified under basic conditions (Scheme 1C). The simplicity and scalability of the syntheses are notable.

**Scheme 1:**
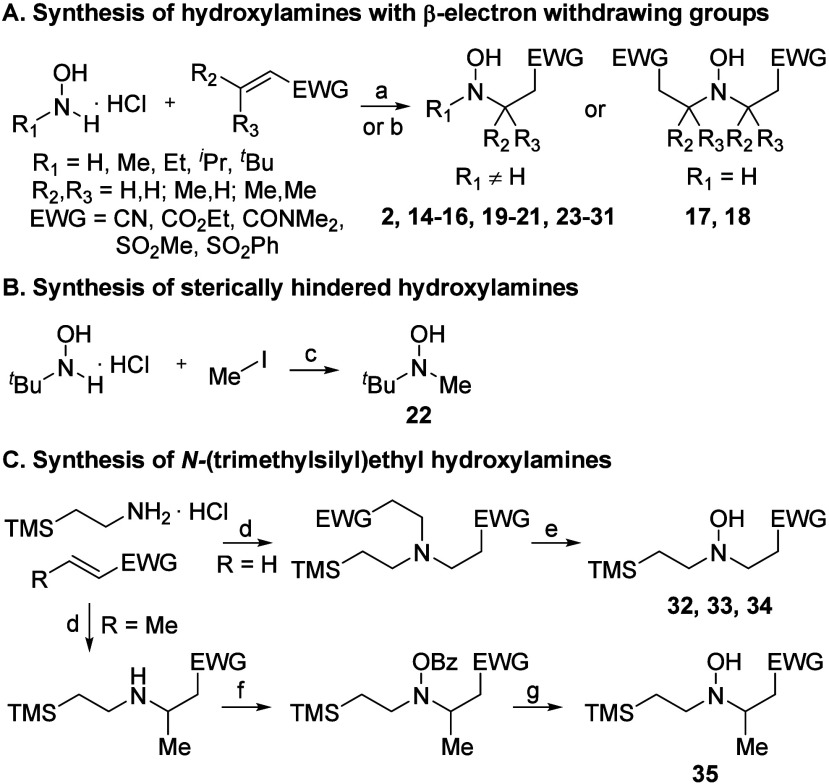
General Synthesis of Hydroxylamine Reagents^a^

a Conditions: (a) hydroxylamine hydrochloride (1.5 equiv), alkene (1 equiv), NEt_3_ (3 equiv), CH_2_Cl_2_, rt; (b) NH_2_OH·HCl, (1 equiv), alkene (2.5 equiv), NEt_3_ (2.5 equiv), MeOH, rt; (c) *N*-*tert*-butylhydroxylamine hydrochloride (1 equiv), MeI (1.2 equiv), Na_2_CO_3_ (3 equiv), DMSO, 50 °C; (d) 2-(trimethylsilyl)­ethylamine hydrochloride (1 equiv), alkene (2.5 equiv), MeOH, rt; (e) CH_3_CO_3_H (1.1 equiv), Na_2_CO_3_ (3 equiv), CH_2_Cl_2_, rt; (f) BzOOBz (2 equiv), K_2_HPO_4_ (6 equiv), DMF, rt; (g) Na_2_CO_3_ (2.5 equiv), MeOH, 0 °C.

With a variety of hydroxylamines in hand, we revisited the initial set of hydroxylamines featuring different β-electron withdrawing groups (**2** and **14**–**18**) alongside the ethyl phenyl sulfonyl-, nitroethyl-, and trifluoropropyl-bearing hydroxylamines (**19**–**21**) as additional substrates (Figure 5D, entries 1–7). Among the seven compounds, phenyl sulfone-functionalized hydroxylamine **19** showed the greatest relative *k*_rCope_ and *k*_obs,max_, suggesting that both electronegativity and hydrophobicity of the β-activating group are important contributors to reaction rate. As an additional comparison, *N*-*tert*-butyl-*N*-methylhydroxylamine (**22**) was found to react more slowly than the reference cyanoethyl hydroxylamine **2** (Figure 5D, entry 8).

Next, the effects of steric bulk on both the noneliminating arm (Figure 5D, entries 9–13) and the eliminating arm (Figure 5D, entries 14–17) were examined. While α-branching on either arm appeared to decrease *k*_rCope_ and moderately increase *k*_obs,max_ to some extent, the effect was neither general nor consistent. It appears that α-branching on hydroxylamines results in a tenuous balance between competing factors. An increase in steric hindrance diminishes the rate of hydroamination, whereas the associated increase in hydrophobicity and electron density at nitrogen enhances it, ultimately leading to mixed trends in reactivity. Interestingly, hydroxylamine **30** bearing an α-branched eliminating arm with a phenyl sulfone β-activating group exhibited a pronounced improvement in both *k*_rCope_ and *k*_obs,max_. With electronic effects seemingly playing a more important role than steric effects, we revisited the hydroxylamine substrates with β-withdrawing groups on both arms (Figure 5D, entries 18–19). The trends were consistent with the NMR experiments, and no appreciable improvement on *k*_rCope_ or *k*_obs,max_ was attained.

Lastly, in a bid to alter both electronegativity and hydrophobicity on the noneliminating arm, the prospect of the β-silicon effect accelerating hydroamination was studied with hydroxylamines bearing a β-trimethylsilyl group on the noneliminating arm (Figure 5D, entries 20–23). Although solubility was mildly compromised, compound **34** exhibited the highest *k*_rCope_ among all of the hydroxylamines tested with more than a 17-fold improvement over that of the reference cyanoethyl hydroxylamine **2**. We note that it is possible to enact synergistic effects on the eliminating and noneliminating arm. An electron-releasing group helps to attenuate the negative impacts of the electron-withdrawing eliminating arm and balance the competing needs of hydroamination versus elimination. A “push-pull” effect on the hydroxylamine coupled with sufficient hydrophobicity provides an optimal chemical environment for the tandem retro-Cope/Cope elimination reaction sequence.

In summary, our kinetic profiling of hydroxylamines allows for the strategic selection of reagents based on the specific demands of downstream applications. For applications requiring high reaction rates at low concentration, such as in in vivo settings where the bimolecular retro-Cope reaction is rate-limiting, hydroxylamine **34** stands out with the fastest kinetics (*k*_rCope_ = 2.09 M^–1^s^–1^). When reagent concentration is not a limiting factor, the α-branched eliminating arm is beneficial and hydroxylamine **30** exhibits the most rapid elimination step (*k*_obs,max_ = 2.18 × 10^–3^ s^–1^). *N*-Methyl-*N*-phenylsulfonylethylhydroxylamine **19** offers a favorable compromise, retaining the electronic benefits of the sulfone series while maintaining a smaller steric profile than **34**. For specific applications where steric hindrance poses a critical challenge, the smallest hydroxylamines with favorable kinetics, hydroxylamines **2** (*k*_rCope_ = 0.121 M^–1^s^–1^) and **20** (*k*_rCope_ = 0.225 M^–1^s^–1^), are preferred.

Based on its superior kinetics, hydroxylamine **34** was nominated for protein uncaging studies, but other derivatives such as hydroxylamines **2**, **19**, and **30** were also considered as they could potentially exhibit greater activity if solubility or protein active site inaccessibility were to prove problematic. Unlike hydroxylamine **34** whose solubility limit is ∼1 mM in aqueous solutions, hydroxylamines **19** and **30** are soluble at higher concentrations (100 mM and 30 mM, respectively). Notably, hydroxylamine **30** was found to have the highest *k*_obs,max_ value and exhibited reaction rates comparable to hydroxylamine **34** at concentrations <1 mM where hydroamination is rate-limiting (Figures 5D, S3, and S4). However, we maintained reservations that the α-branching on **30** may confer an undesired steric penalty when accessing the active site. Given these considerations, we elected to advance hydroxylamines **19** along with **34**, with the ethyl phenylsulfone eliminating arm as the shared feature between the pair.

The stabilities of hydroxylamines **19** and **34** were evaluated in different biologically relevant conditions at various time points (Figure 6A). While hydroxylamine **19** showed complete stability in each of the tested conditions (PBS, 23 or 37 °C; HEK293T cell lysate, 1 mg/mL, 23 or 37 °C; DMEM, 23 °C; human liver S9 fraction, 0.2 mg/mL, 23 °C) up to 24 h, hydroxylamine **34** exhibited roughly 50% degradation at 24 h in both PBS and HEK293T cell lysate. This is consistent with our prior observations that *N*,*N*-dialkylhydroxylamines are susceptible to autoxidation.^34^ Sensitivity of the hydroxylamines is correlated with the electronics of the alkyl substituents, and the introduction of an electron-withdrawing group effectively suppresses this process on the eliminating arm; *N*-methylhydroxylamines like hydroxylamine **19** are resistant to degradation. Substitution of the methyl for an electron-rich trimethylsilylethyl group introduces the potential for oxidation on the noneliminating arm, consistent with the relative instability of hydroxylamine **34**. Nonetheless, both hydroxylamines **19** and **34** are sufficiently stable over the 1 h during which the reaction reaches completion in vitro.

**Figure 6:**
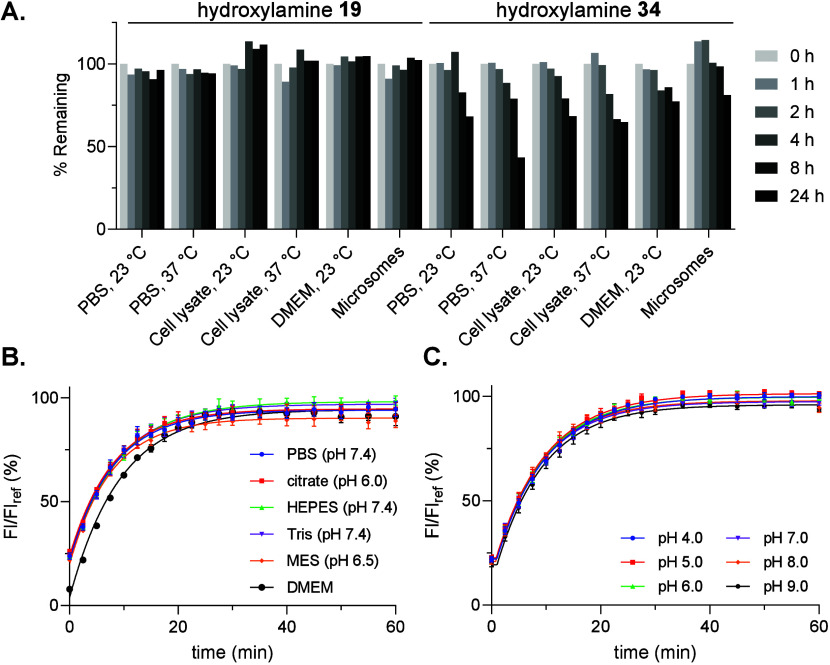
Stability and buffer and pH dependence studies. (A) Stability of hydroxylamines **19** and **34** at various time points and temperatures in PBS (pH 7.4), cell lysate (1 mg/mL), DMEM, and microsomes (0.2 mg/mL). (B) Time-dependent fluorescence activation of cyclooctynyl rhodamine **36** (10 μM) by hydroxylamine **19** (2.5 mM) in 40% (v/v) methanol in various buffers and cell culture media (PBS (pH 7.4), citrate (10 mM, pH 6.0), HEPES (50 mM, pH 7.4), Tris (50 mM, pH 7.4), MES (20 mM, pH 6.5), and serum-free DMEM). (C) Time-dependent fluorescence activation of cyclooctynyl rhodamine **36** (10 μM) by hydroxylamine **19** (2.5 mM) in 40% (v/v) methanol in PBS at various pH’s (pH 4.0, 5.0, 6.0, 7.0, 8.0, 9.0). Error bars represent mean ± SEM of data from biological replicates (*n* = 3).

Finally, additional experiments with the fluorogenic assay showed that the reaction rate is minimally affected by buffer components such as citrate, HEPES, MES, and Tris (Figure 6B) or pHs between 4 and 9 (Figure 6C). The results from the fluorogenic assays in Figure 6B,C demonstrate that the phenyl sulfone-containing hydroxylamine derivatives are exemplary substrates for the reaction and are promising for application toward chemically controlling protein function; these reactions are fast, clean, and complete within 1 h. The reactions reach >95% completion in 20 min.

Having explored the hydroxylamine substrate scope and identified promising hydroxylamine candidates such as the phenyl sulfone-containing derivatives, we sought to illustrate the potential of the reaction in the context of deprotecting cyclooctyne-functionalized amino acid residues on proteins via genetic-code-expansion technology. We used the Y271A/Y349F double mutant of the tRNA synthetase derived from *Methanosarcina mazei*, which was previously reported to incorporate cyclooctynyl carbamate-caged lysine (COTK) effectively in *E. coli*.^40^

A plasmid encoding the synthetase and pyrrolysyl tRNA alongside a second plasmid encoding superfolder green fluorescence protein (sfGFP) containing a C-terminal His6-tag and an amber stop codon at position 150, a surface exposed asparagine residue (N150TAG),^41^ were then cotransformed into *E. coli* BL21­(DE3) and expressed in media supplemented with 1 mM COTK (**38**) (Figure 7A). No sfGFP was expressed when COTK was excluded in the expression media (Figures 7B and S5). After expression and purification of the cyclooctynyl-functionalized protein, aliquots of the protein (10 μM in PBS, pH 7.4) were immobilized on Ni-NTA magnetic beads and then treated with varying concentrations (10 μM–1 mM) of hydroxylamine **34** for 1 h. The supernatant containing hydroxylamine was then removed. To determine the extent of protein uncaging, the samples were incubated with TAMRA-hydroxylamine **39** (100 μM) (Figure 7A,C). The samples were then subjected to SDS-PAGE, in-gel fluorescence visualization, and analysis for diminished capacity for fluorescent labeling (Figures 7D and S6). Significant uncaging was observed with 100 μM hydroxylamine, and complete uncaging was observed at 1 mM. The deprotection was further confirmed by intact mass spectrometry analysis of sfGFP-COTK before and after treatment with 1 mM hydroxylamine **34** for 1 h (Figures 7E and S7). Under this condition, deprotection was quantitative and no remaining cyclooctynyl-protected starting material was detected.

**Figure 7:**
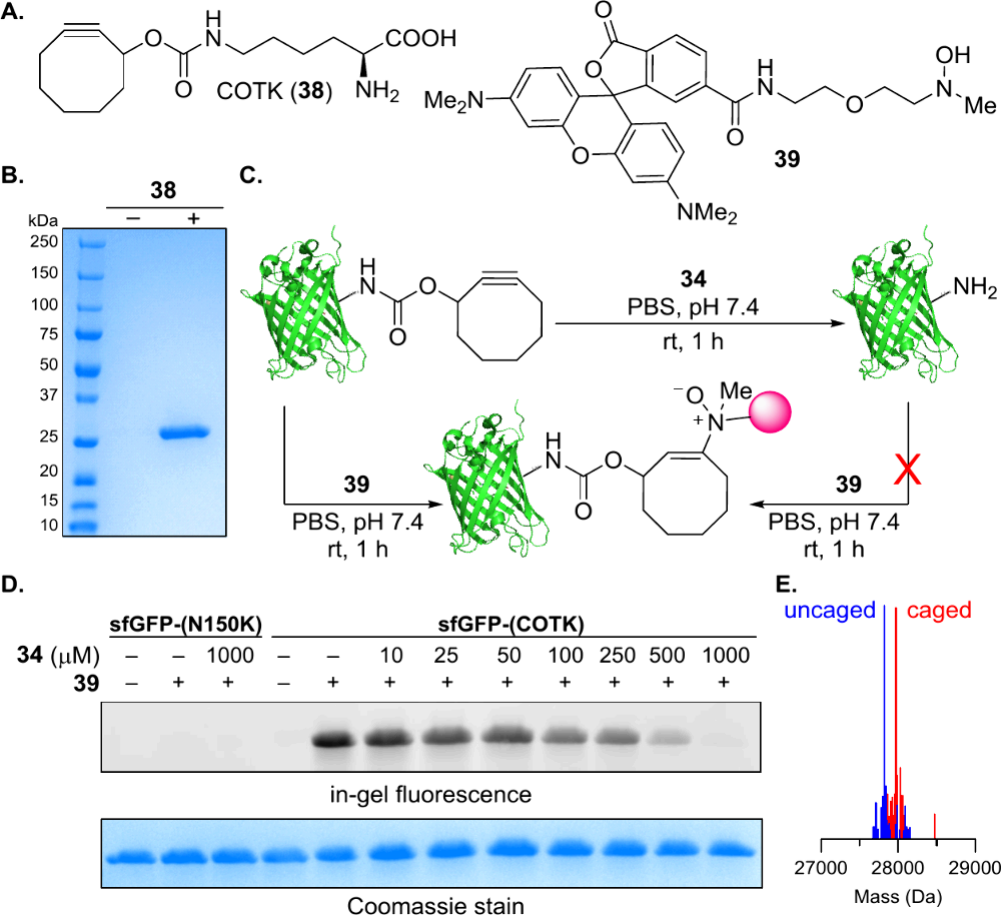
Lysine deprotection on purified proteins. (A) Chemical structure of cyclooctynyl lysine COTK (**38**) and TAMRA-hydroxylamine **39**. (B) Aliquots from clarified lysates of sfGFP­(N150TAG)-His6 expression cultures expressed with or without 1 mM COTK. Cultures were separately purified, and the eluents were subjected to SDS-PAGE and Coomassie staining. (C) General scheme for the deprotection of cyclooctynyl carbamate-functionalized sfGFP with hydroxylamine **34**. TAMRA-hydroxylamine (TAMRA-HA, **39**, 100 μM) is conjugated onto any remaining cyclooctynyl-caged protein. The reagent will not label uncaged protein. (D) Evaluation of reaction completion via the TAMRA-HA chase assay. Complete uncaging is characterized by the absence of TAMRA-conjugated protein in the in-gel fluorescence. The uncaged form of the protein, sfGFP-(N150 K), was also prepared by site-directed mutagenesis of the wildtype construct. It was expressed and purified in parallel as a negative control. (E) Intact mass spectrometry analysis of cyclooctyne-caged sfGFP with and without addition of 1 mM **34** for 1 h. Found mass: sfGFP­(COTK), 27973 Da; sfGFP­(COTK) + **34**, 27824 Da.

With this result in hand, we looked to perform chemical activation of an enzyme in mammalian cells. We chose the well precedented firefly luciferase (FLuc) assay to benchmark our results against those of existing methods.^25,26^ Consistent with previous examples utilizing caged luciferase as the model system to demonstrate controlled enzyme activation in cells, we introduced COTK at catalytic lysine 529, which, when substituted with a sterically demanding protecting group, inhibits catalytic activity.^25^ Accordingly, HEK293T cells were cotransfected with a plasmid encoding the dual luciferase reporter (FLuc­(K529TAG)-RLuc) and the MmPylRS/PylT pair in the presence of COTK (1 mM) in the culture media (Figure 8A). As an additional comparison with a previously characterized system, a separate transfected culture was supplemented with *trans*-cyclooctenyl lysine (TCOK, **41**), which was to be uncaged with dimethyltetrazine (DMTz, **40**) (Figure 8B). To account for variable expression levels, the orthogonal *Renilla* luciferase (RLuc) was coexpressed alongside FLuc as a fusion protein to allow for the normalization of the FLuc signal.

**Figure 8:**
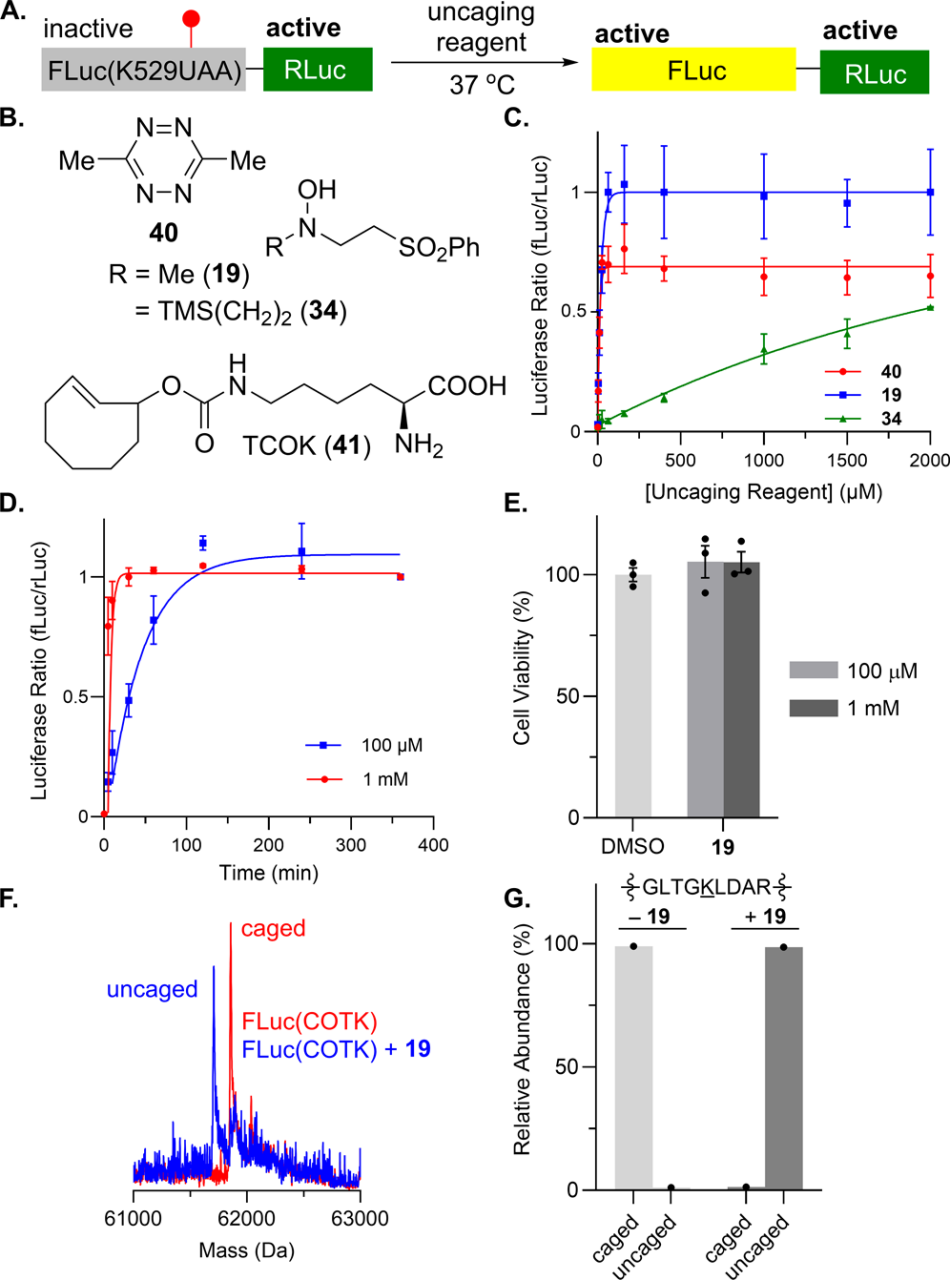
Enzymatic uncaging in mammalian cells. (A) General scheme for the activation of firefly luciferase in live HEK293T cells. (B) Chemical structures of TCOK and chemical uncaging reagents **40**, **19**, and **34**. (C) Concentration dependence of various small molecule uncaging reagents in firefly luciferase activation. HEK293T cells expressing FLuc bearing different caged lysine residues in the active site were treated with their corresponding small molecule uncaging reagent for 3 h. (D) Time-course of luciferase uncaging with hydroxylamine **19** at 100 μM and 1 mM. (E) Cell viability 3 h postluciferase activation with hydroxylamine **19**. (F) Intact mass spectrometry analysis of purified caged luciferase with and without 100 μM **19** treatment for 3 h. (G) Relative abundance of peptides containing K529 obtained from the tryptic digest of affinity-purified samples of caged FLuc with and without 100 μM **19** treatment for 3 h.

Twenty-four hours post-transfection, cells supplemented with COTK were treated with hydroxylamine derivative **19** or **34**, and cells supplemented with TCOK were treated with commercially available dimethyltetrazine (**40**). After 3 h of treatment, the reagents were removed, and the luciferase activity was measured by the Dual-Glo Luciferase assay (Figure 8C).

Interestingly, hydroxylamine **34**, which was shown to have the best reaction rates among the hydroxylamines tested in the in vitro fluorogenic kinetics experiments, did not fully restore FLuc activity after 3 h. In contrast, hydroxylamine **19**, which has a methyl group in lieu of the trimethylsilylethyl group on the noneliminating arm, was able to fully restore FLuc activity with concentrations as low as 100 μM. Indeed, when the experiment was repeated with 100 μM hydroxylamine **19** in a time course experiment, it was observed that the luciferase activity is fully restored by 2 h (Figure 8D). Complete activation can also be attained by 30 min with 1 mM hydroxylamine.

Dissociative transformations in protein deprotection and activation schemes do not face the same challenges as their associative counterparts with respect to nonspecific background labeling arising from the use of excess reagent and are less constrained by their kinetic parameters. As such, while the complete recovery of luciferase activity within 2 h by 100 μM hydroxylamine **19** was satisfactory, we surmised from prior characterization of the retro-Cope/Cope elimination kinetic profile (Figure 5C) that the reaction rate could be further enhanced by increasing the hydroxylamine concentration, insofar as the reagent is well-tolerated by cells at those levels. We therefore conducted cell viability studies in HEK293T cells treated with hydroxylamines **2**, **19**, and **34** for 3 h, an exposure sufficient for complete reaction even at 100 μM. None of the reagents exhibited significant toxicity up to 2.5 mM (Figures 8E and S8A). One can expect near-proportional improvement in reaction rate with increasing hydroxylamine concentrations without experiencing toxicity up to these levels.

In addition to any cytocompatibility issues of hydroxylamines, we were circumspect to the potential toxicity of electrophilic byproducts produced by the retro-Cope/Cope elimination cascade, so their impact on cell viability was also evaluated (Figure S8C–F). Exposure of HEK293T cells to acrylonitrile and phenyl vinyl sulfone for 3 h revealed no significant toxicity up to 1 mM for the former and an IC_50_ of 57 μM for the latter. A more representative evaluation of reaction compatibility was performed by deprotection of *N*,*N*-dimethyl cyclooctynyl carbamate **S14** by hydroxylamines **2** and **19** in the presence of cells. Minimal toxicity was observed up to 100 μM in cyclooctynyl carbamate **S14**, the maximum concentration tested. Cellular exposure to electrophiles produced by this process is governed by the concentration of the limiting cyclooctyne-caged component rather than that of the hydroxylamine trigger in intended applications.

Reengaging the anomalous result that the ostensibly slower hydroxylamine **19** was faster than **34** in uncaging luciferase (Figure 8C), since neither hydroxylamine **19** nor **34** appeared to be challenged by toxicity or membrane permeability issues (Figures S8–S10), we hypothesized that the inefficient deprotection of the caged luciferase with hydroxylamine **34** was due to the steric effects of the trimethylsilylethyl group and the challenges of accommodating a larger enamine *N*-oxide adduct at the catalytic lysine of the luciferase active site. This highlights the critical need for smaller dissociative reaction pairs for use in bioorthogonal cleavage applications. Nevertheless, the less sterically hindered hydroxylamine **19** presents a viable alternative with rates comparable to other bioorthogonal deprotection methods. In tight spaces, smaller hydroxylamines may be optimal for deprotecting relatively buried lysine residues. Furthermore, in our hands, the TCOK/DMTz activation only resulted in 70% of the luciferase activity restored, even with the highest concentration tested. Consistent with in vitro results and literature precedent, this is likely due to the formation of dead-end adducts.^27–30^ Indeed, intact mass spectrometry analyses of purified luciferase expressed in HEK293T cells before and after deprotection show complete deprotection of COTK-caged luciferase with 100 μM of hydroxylamine **19** (Figures 8F and S11). This is further corroborated with peptide-level analysis in which tryptic digestion of affinity-purified, COTK-caged luciferase shows complete restoration of the native K529 residue upon treatment with 100 μM of hydroxylamine **19** (Figures 8G and S12).

Having surmised that size constraints of the small molecule trigger play a crucial role in determining feasibility of uncaging—especially in the context of enzymatic activation—we performed molecular docking studies on a representative panel of caged lysine adducts in order to gauge their steric footprint and quantify the accessibility of their corresponding triggers within the luciferase active site (Figures 9 and S13). While covalent docking models are likely to generate adducts with the specified connectivity and optimized conformation, their positions in the enzymatic active site (relative to the binding of the native substrate) and presence of steric clashes (positive interaction energy terms) with key solvent-exposed residues are two important determinants of their feasibility, which in turn may be reflected experimentally. Here, covalent docking was conducted using Molecular Operating Environment (MOE) software with energy-minimized adducts docked onto residue K529. The pose with the highest docking score was selected, and properties such as the van der Waals volume of the adduct and its interaction energies with key residues that line the binding pocket were calculated and presented.

**Figure 9:**
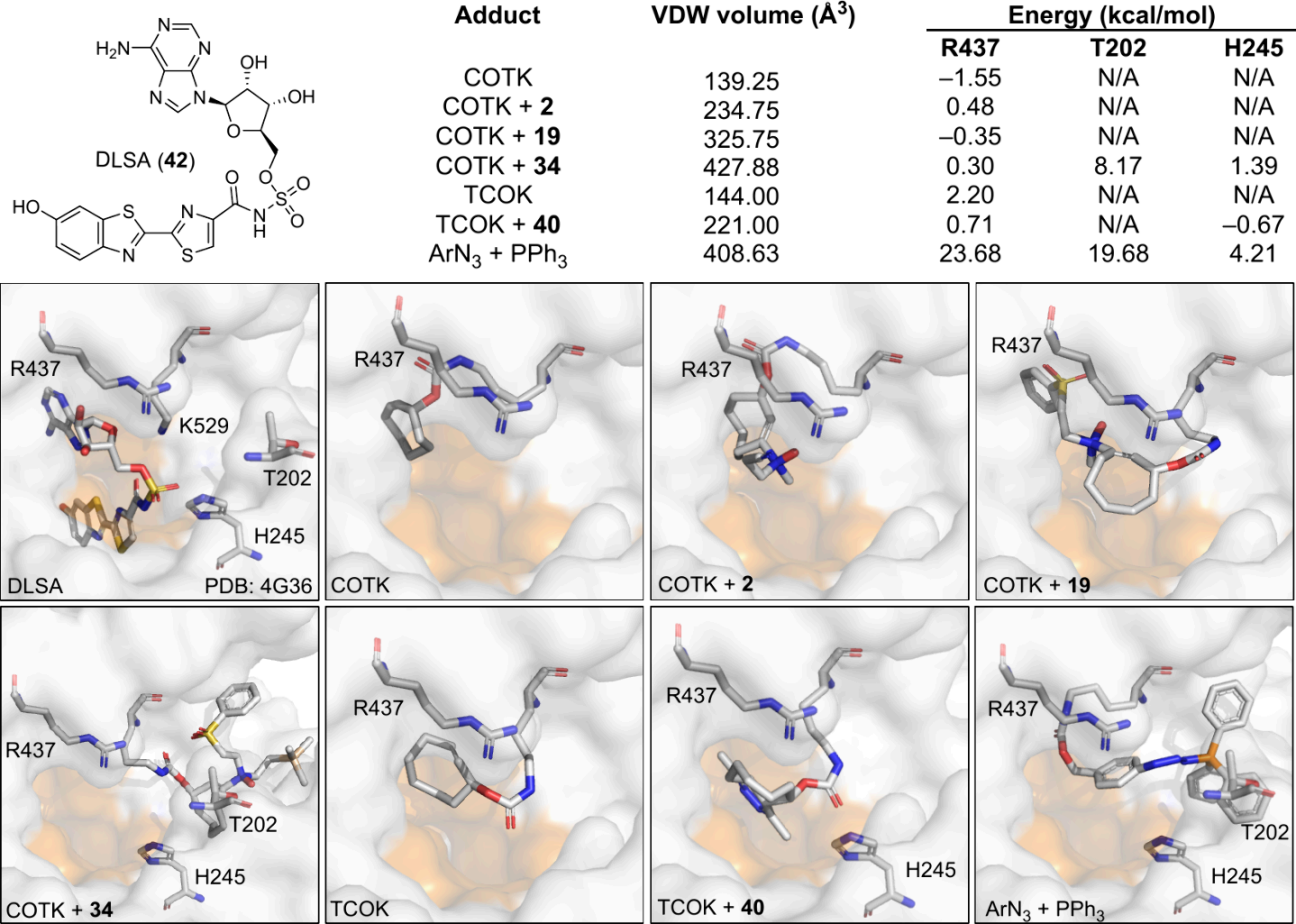
Molecular docking of caged Lys529 adducts with various small molecule triggers in firefly luciferase. The top left panel depicts the crystal structure of firefly luciferase complexed to 5′-*O*-[*N*-(dehydroluciferyl)-sulfamoyl]­adenosine (DLSA, **42**) to illustrate the active site which is highlighted in orange (PDB id: 4G36). Additionally, in each panel, selected residues (R437, T202, H245) that are found to interact with the adduct are shown. Their interaction energies are tabulated in the table alongside the van der Waals volume of the adduct.

From our computations, the steric footprint of COTK and TCOK are similar at 139 and 144 Å^3^, respectively. The TCO-dimethyltetrazine **40** adduct is 221 Å^3^. While smaller than many of the COTK-hydroxylamine adducts, as evidenced experimentally, tetrazine **40** is unable to achieve complete release and subsequent activation of luciferase due to issues of regioisomeric tautomerization. Bisaryltetrazines **S15** and **S16** reportedly mitigate the tautomerization issue but at the expense of size;^30,42^ the volume of their TCOK adducts are 345 and 597 Å^3^, respectively (Figure S13). Importantly, neither adduct can fit in the luciferase ATP or luciferin binding pockets. Both adducts are displaced into an adjacent groove with portions of the adduct solvent exposed. Furthermore, they exhibit energetically costly steric interactions with one or more residues that line the lip of the active site including R437, H245, and T202. Such is the case with hydroxylamine **34**; its adduct with COTK clashes with residue T202 and H245 with energy penalties worth 8.2 and 1.4 kcal/mol, respectively. Meanwhile, the COTK-adduct with the less sterically hindered hydroxylamine **19** does not present significant steric penalties with the aforementioned residues. Lastly, the representative adduct of the Staudinger reduction involving aryl azide and triphenylphosphine also suffers from unfavorable interaction energies with R437, T202, and H245. Overall, results from molecular docking experiments are consistent with experimental results; reagents that cannot engage cannot uncage.

## Conclusions

In this report, we described a bioorthogonal click-to-release reaction cascade employing a retro-Cope/Cope elimination sequence in which strain-promoted hydroamination of a cyclooctyne by an *N*,*N*-dialkylhydroxylamine is relayed into Cope elimination of the resulting enamine *N*-oxide. β-Elimination of the *N*-hydroxyenamine product results in bond cleavage. The reaction is regioselective, and the cleavage is directional. The transformation enables the rapid and complete cleavage of a chemical bond in biologically relevant settings using reagents with a small molecular footprint.

While our previously reported bioorthogonal reduction of enamine *N*-oxides by diboron reagents^36^ remains a superior method for bond cleavage under biologically relevant conditions for applications in which an enamine *N*-oxide linker can be incorporated directly into a compound structure (e.g., prodrugs, protein-small-molecule conjugates, etc.), the current click-to-release strategy addresses a key limitation of size. Size constraints are prominent in chemical matter that needs to traverse or modulate biochemical processes. While small click reagents exist, the corresponding reagents and chemistry that seek to remove or leverage these groups in dissociative processes have room for improvement.

In designing the retro-Cope/Cope elimination sequence, we took advantage of the propensity of amine *N*-oxides to undergo rapid degradation via Cope elimination from alkyl substituents bearing a β-electron withdrawing group. Our linear free energy relationship data indicate a strong correlation between the inductive effects of the β-substituent and the rate of elimination.^36^ Navigating the competing requirement for electron-rich substituents to facilitate the initial retro-Cope elimination-mediated click transformation was the central challenge.

This work provides a collection of hydroxylamines suited for a range of tasks in which speed, size, or a balance thereof is the parameter on which the highest premium is placed. The smallest hydroxylamine with reasonable reaction kinetics (*k*_rCope_ 0.121 M^–1^s^–1^; *k*_obs,max_ 9.13 × 10^–4^ s^–1^) is *N*-cyanoethyl-*N*-methylhydroxylamine **2**; the fastest reacting is *N*-trimethylsilylethyl-*N*-phenylsulfonylethylhydroxylamine **34** (*k*_rCope_ 2.09 M^–1^s^–1^); the fastest eliminating hydroxylamine (*k*_obs,max_ 2.18 × 10^–3^ s^–1^) is *N*-methyl-*N*-phenylsulfonyl-2-methylethylhydroxylamine **30**; and the hydroxylamine exhibiting the best balance between size and kinetics (*k*_rCope_ 0.881 M^–1^s^–1^, *k*_obs,max_ 1.29 × 10^–3^ s^–1^) is *N*-methyl-*N*-phenylsulfonylethylhydroxylamine **19**.

All second-order reaction rates are reasonably fast, comparing favorably to the fastest strain-promoted azide-cyclooctyne cycloadditions known and of practical value. Importantly, the secondary elimination step is rapid such that these dissociative processes are not rate-limited by the subsequent elimination steps up to a concentration of several millimolars of hydroxylamine reagent.

In applications where this reaction would prove most useful, such as in the removal of small organic protecting groups incorporated on metabolites or biochemical inputs that traverse multiple biochemical pathways, we surmise that a principal determinant of its utility will be its size; a fast reaction that is access-limited equates to no reaction at all. We illustrate this principle in an application in which the unnatural amino acid cyclooctynyl lysine is incorporated into luciferase at a key catalytic residue followed by bioorthogonal uncaging using hydroxylamines **19** and **34**. The latter is 2.4-fold faster in solution but markedly slower in this context.

Finally, the reaction we describe is compatible with common biological buffers and culture media that were tested: pH from 4 to 9 and live bacterial and mammalian cells. The two evaluated hydroxylamines (**19** and **34**) did not exhibit significant adverse impacts on mammalian cell viability even at millimolar levels. The combination of reaction rate, completeness of bond cleavage, size of reaction components, compatibility with bacterial and mammalian systems, and the simplicity of reagent synthesis are features that trend positively for this bioorthogonal dissociation reaction.

## Supplementary Material



## Notes

Experimental procedures, synthesis and characterization of compounds, and NMR spectra (PDF)
