# Supplementary material for: Bioorthogonal Activation of Protein Function through a retro-Cope/Cope Elimination Cascade
Source: J Am Chem Soc. 2026 Jun 25;148(26):27442–54. doi: 10.1021/jacs.6c05497 (PMC13352630; doi:10.1021/jacs.6c05497)
Supplement: Supplementary file 1 [file ja6c05497_si_001.pdf]

Bioorthogonal activation of protein function through a retro-Cope/Cope elimination cascade

Surached Siriwongsup,[†1,2] Sanghyeon Lee,[†1,2] Yiming Guo,[3] Conrad Wahl,[1] and Justin Kim*[3]

[1]*Department of Cancer Biology, Dana-Farber Cancer Institute, Boston, MA 02215, United States.*

[2]*Department of Biological Chemistry and Molecular Pharmacology, Harvard Medical School, Boston, MA 02115, United States.*

[3]*School of Chemistry and Biochemistry, Georgia Institute of Technology, Atlanta, GA 30332, United States.*

[†]These authors contributed equally to this work.

Supporting Material

General Synthesis Information S2
General Chemical Materials S2
General Chemical Instrumentation S2
General Biological Materials S2
General Biological Instrumentation S3
Chemical and Biological Procedures S4
Figures S1–S13 S16
Synthetic Procedures and Characterization S28
References S50
Copies of NMR Spectra S51

General Synthesis Information. All reactions were conducted in flame-dried round-bottom flasks under a positive pressure of nitrogen unless otherwise stated. Gas-tight syringes with stainless steel needles or cannulae were used to transfer air- and moisture-sensitive liquids. Flash column chromatography was performed using granular silica gel (60-Å pore size, 40–63 µm, Silicycle). Analytical thin layer chromatography (TLC) was performed using glass plates pre-coated with 0.25 mm silica gel impregnated with a fluorescent indicator (254 nm, Silicycle). TLC plates were visualized by exposure to short wave ultraviolet light (254 nm) and/or an aqueous solution of potassium permanganate ($KMnO_4$). Organic solutions were concentrated at 20 °C on rotary evaporators capable of achieving a minimum pressure of ~2 torr unless otherwise stated. Room temperature is defined as 22.5 ± 2.5 °C. Reaction heating was performed using a UCON fluid heating bath.

General Chemical Materials. All solvents were purchased from Fisher Scientific or Sigma–Aldrich. Unless otherwise stated chemical reagents were purchased from Fisher Scientific, Sigma–Aldrich, Alfa Aesar, Oakwood Chemical, Acros Organics, Combi-Blocks, TCI America, or Chem-Impex. 3,6-dimethyl-1,2,4,5-tetrazine (**40**) was purchased from Ambeed. Trans-cyclooct-2-en-L-lysine (TCOK, **41**) was purchased from Sirius Fine Chemicals (cat. SC-8008). CMA refers to a solution of 80:18:2 v/v/v chloroform:methanol:ammonium hydroxide (28–30% ammonia solution). Chloroform used in CMA solutions and as co-eluents in silica gel column chromatography were stabilized with 0.75% v/v ethanol. Chloroform used in all hydroamination reactions were stabilized with pentene.

General Chemical Instrumentation. Proton nuclear magnetic resonance (^1H NMR) spectra, recorded with a 500 MHz Avance III Spectrometer with multi-nuclear Smart probe, are reported in parts per million on the δ scale, and are referenced from the residual protium in the NMR solvent ($CDCl_3$: δ 7.24, CD_3OD: δ 3.31, DMSO-d_6: δ 2.50). Data are reported as follows: chemical shift [multiplicity (s = singlet, d = doublet, t = triplet, q = quartet, p = quintet, dd = doublet of doublets, dt = doublet of triplets, dq = doublet of quartets, ddd = doublet of doublets of doublets, tt = triplet of triplets, td = triplet of doublets, m = multiplet), coupling constant(s) in Hertz, integration, assignment]. Carbon-13 nuclear magnetic resonance (^{13}C NMR) spectra are referenced from the carbon resonances of the solvent ($CDCl_3$: δ 77.23, CD_3OD: δ 49.15, DMSO-d_6: δ 39.51). Fluorine-19 nuclear magnetic resonance (^{19}F NMR) is calibrated from the fluorine resonances of benzotrifluoride ($CDCl_3$: δ –62.76, CD_3OD: δ –64.24). Data are reported as follows: chemical shift (assignment). Infrared data (IR) were obtained with a Cary 630 Fourier transform infrared spectrometer equipped with a diamond ATR objective and are reported as follows: frequency of absorption (cm^{-1}), intensity of absorption (s = strong, m = medium, w = weak, br = broad). High resolution mass spectra (HRMS) were recorded on a Q Exactive™ Plus Hybrid Quadrupole-Orbitrap™ Mass Spectrometer using an electrospray ionization (ESI), atmospheric pressure chemical ionization (APCI), or electron ionization (EI) source. Automated C18 reverse phase chromatography was performed using an Isolera One (Biotage) purification system. High performance liquid chromatography (HPLC) purification was performed using an Agilent 1260 Infinity system.

General Biological Materials. All solvents and reagents were purchased from commercial suppliers and used as received. Deionized water (>18.2 MΩ) was used to prepare all aqueous buffers and solutions. Short oligonucleotide primers (<80 bp) were synthesized by Millipore Sigma

(St. Louis, MO) while gene blocks (>80 bp) were synthesized by Twist Biosciences (San Francisco, CA). Oligonucleotides were shipped dry and used as received without further desalting. Chemically competent *E. coli* DH5α and BL21(DE3) cells were purchased from New England Biolabs. All plasmid isolations were performed with a miniprep or midiprep kit from Zymo Research. DNA clean and concentrator and DNA gel purification kits were purchased from Zymo Research. Gibson cloning and assembly was performed with the NEBuilder HiFi DNA Assembly Kit (New England Biolabs) and site-directed mutagenesis was performed with the Q5 Site-Directed Mutagenesis kit (New England Biolabs). Sanger sequencing service was performed by Quintara Biosciences (Cambridge, MA). Some His-tagged recombinant proteins were purified using NEBExpress Ni-NTA magnetic beads or NEBExpress Ni spin columns (New England Biolabs). HEK293T cells were purchased from ATCC. Mammalian cell transfection was performed with the TransIT-293 transfection reagent (Mirus Bio). The Parallel Artificial Membrane Permeability Assay (PAMPA) was performed using the BioCoat® Pre-coated PAMPA Plate System (Corning, Cat. No. 353015).

General Biological Instrumentation. All polymerase chain reactions (PCR) were performed on a Bio-Rad Laboratories C1000 thermal cycler. Bacterial cells were lysed using a Fisherbrand Sonicator Model 505. Some His-tagged recombinant proteins were purified with a Bio-Rad NGC chromatography system (FPLC). UV/Vis absorbance measurements for protein A280 determination were acquired on an Agilent Technologies Cary 60 UV-Vis spectrophotometer. Other absorbance measurements (including protein BCA), fluorescence, and luminescence data were acquired on a Clariostar Plus microplate reader (BMG Labtech). In-gel fluorescence imaging was performed on a GE Healthcare Life Sciences Typhoon FLA 9500. Images were processed with Fiji ImageJ software. Coomassie-stained SDS-PAGE gels were imaged on a Bio-Rad Molecular Imager Gel Doc XR+ imaging system.

Chemical and biological procedures

Reaction progress monitoring by ^{1}H NMR spectroscopy

A solution of cyclooctynyl *p*-nitrophenyl carbamate (**1**) (620 µL, ~12 mM in CD_3OD; 10.4 mM final concentration) and internal standard 1,3,5-trimethoxybenzene (40 µL, 70 mM in CD_3OD; 4 mM final concentration) was mixed in an NMR tube. Then, an equimolar solution of hydroxylamine **2** or **8** (~40 µL, 188 mM in CD_3OD; 10.4 mM final concentration) was added at room temperature to bring the total volume to ~700 µL. The progress of the reaction was monitored by ^{1}H NMR spectroscopy (scan number = 4, relaxation delay = 20 s), and the amount of each species was quantified against the 1,3,5-trimethoxybenzene internal standard. The experiment was performed in triplicate.

The click-to-release reactions between water-soluble cyclooctyne carbamate **12** and hydroxylamines **2, 14−18** were conducted in 20% DMSO-d_6/*d*-PBS (v/v) and monitored via ^{1}H NMR spectroscopy using 4-fluorophenol (20 mM) as an internal standard. A solution of cyclooctyne **12** (50 µL, ~240 mM in DMSO-d_6, titrated with internal standard solution; 20 mM final concentration) and 4-fluorophenol (50 µL, 240 mM in DMSO-d_6; 20 mM final concentration) was mixed with *d*-PBS (pH 7.4, 460 µL) in tube. Then an equimolar solution of hydroxylamines **2, 14−18** (~300 mM in 50% DMSO-d_6/*d*-PBS (v/v), titrated with internal standard solution; 20 mM final concentration) was added at room temperature. Reaction progress was monitored by ^{1}H NMR. Cyclooctyne carbamate **12** was quantified based on integration of the δ 5.17 peak (-O-C-H), and amine release product **13** was quantified based on integration of the δ 6.39 peak (N=C-C=C-H). Second-order kinetics experiments were performed in triplicate.

Reaction progress monitoring by liquid chromatography-mass spectrometry (LC-MS).

A solution of cyclooctynyl *p*-nitrophenyl carbamate (**1**) (620 µL, 12 mM in methanol; 10.4 mM final concentration) and internal standard 1,3,5-trimethoxybenzene (40 µL, 70 mM in methanol; 4 mM final concentration) was mixed in a microcentrifuge tube. Then, an equimolar solution of hydroxylamine **2** (40 µL, 188 mM in methanol; 10.4 mM final concentration) was added at room temperature to bring the total volume to 700 µL. The progress of the reaction was monitored by LC-MS (Pursuit 200 Å C18, 4.6× 150 mm, 10 µm particles, 1 mL/min flow rate, eluent: isocratic 0% MeCN/H_2O + 0.1% TFA (1 min), gradient 0→100% MeCN/H2O + 0.1% TFA (16 min), isocratic 100% MeCN/H_2O + 0.1% TFA (1 min)).

Fluorogenic cleavage reaction kinetics study ***(pseudo-first-order kinetics model)***:

Glycinyl rhodamine **37** or cyclooctynyl glycinyl rhodamine **36** (90 µL, 13.3 µM in 40% MeOH/PBS (v/v), pH 7.4) were added to wells of a round-bottom 96-well plate. A 4× solution of hydroxylamine **2, 14–35** (30 µL, 400 µM–400 mM in 40% MeOH/PBS (v/v), pH 7.4) or vehicle control (30 µL) was then added into each well containing **36** or **37**, mixed by pipetting and immediately redistributed to separate wells of a black-walled, opaque 384-well plate (30 µL per well). Pseudo-first-order kinetics experiment was performed by measuring fluorescence intensity with excitation wavelength of 487-14 nm and emission wavelength of 535-30 nm every 3 min for

60 min using a Clariostar Plus microplate reader (BMG Labtech). The gain was adjusted prior to the kinetics measurement. The experiment was performed in triplicate. Percent release was determined as the ratio (F/F_{ref}) between fluorescence intensity in the reaction of **36** with hydroxylamine (F) and the reaction of **37** with hydroxylamine (F_{ref}) at each time point. The second-order rate constant (k_{rCope}) of each hydroxylamine was calculated by taking the rate of rhodamine release (k_{obs}) at each concentration and determining the slope, which is linear at low hydroxylamine concentrations (< 1 mM) using simple linear regression. The plot of k_{obs} at concentrations beyond 1 mM is nonlinear, and an exponential plateau fit was used to estimate the maximum observed rate ($k_{obs,max}$) at higher (> 50 mM) concentrations of hydroxylamine.

For the pH dependence study, the assay was performed as described above but with reaction solvents consisting of 40% MeOH/PBS buffered at different pH's (pH = 4.0, 5.0, 6.0, 7.0, 8.0, 9.0). For the buffer dependence study, the assay was performed with solvents consisting of 40% MeOH / 60% of various aqueous buffers (PBS pH 7.4; 10 mM citrate, pH 6.0; 50 mM Tris, pH 7.4; 50 mM HEPES, pH 7.4; 20 mM MES, pH 6.5), or serum-free DMEM cell culture media .

Hydroxylamine permeability studies

The membrane permeability of hydroxylamines **19**, **28**, **34**, **35** was evaluated via the Parallel Artificial Membrane Permeability Assay (PAMPA) using the BioCoat® Pre-coated PAMPA Plate System (Corning, Cat. No. 353015) following the manufacturer's protocol. Specific details are included below.

First, a 10 mM stock solution of each hydroxylamine and control compounds (caffeine for the high permeability control, famotidine for the low permeability control) in DMSO was diluted with PBS (pH 7.4) to prepare the donor solution (final concentration: 500 µM in 5% DMSO/PBS). The donor solution was added to the wells of the receiver plate (300 µL per well), and acceptor solution (PBS, pH 7.4) was added to the pre-coated filter plate (200 µL per well). The filter plate was then assembled on top of the receiver plate and the plates were incubated at room temperature for 5 h without agitation. After 5 h, the two plates were separated and 150 µL of solution from both receiver plate and filter plate was transferred to wells of a UV-transparent 96-well plate (Corning). The UV absorbance at λ_{max} of each compound (263 nm for famotidine, 273 nm for caffeine, 266 nm for the hydroxylamines) was measured on the microplate reader (Clariostar Plus, BMG Labtech). The final concentrations of compounds in the donor solution and acceptor solution were estimated by UV absorbance. Permeability of the compounds was calculated using the following formula:

$$\text{Permeability (cm s}^{-1}\text{): } P_e = \left(\frac{-\ln\left(\frac{1-C_A(t)}{C_{eq}}\right)}{At\left(\frac{1}{V_D}+\frac{1}{V_A}\right)} \right)$$

where A = filter area (= 0.3 cm^2), V_D = donor well volume (= 0.3 mL), V_A = acceptor well volume (= 0.2 mL), t = incubation time (= 18000 s), $C_A(t)$ = compound concentration in acceptor well at time *t*, $C_D(t)$ = compound concentration in donor well at time t, and $C_{eq} = \left(\frac{C_D(t)\times V_D + C_A(t)\times V_A}{V_D + V_A}\right)$

Hydroxylamine stability studies

All reactions were monitored by HPLC at 0, 1, 2 ,4, 8, and 24 h time points.

HPLC analysis: Stability analyses of hydroxylamine **19** and hydroxylamine **34** were performed using 4-nitroaniline as an internal standard and analyzed by HPLC (Pursuit 200 Å C18, 4.6× 150 mm, 10 µm particles, 1 mL/min flow rate, eluent: isocratic 0% MeCN/H_2O + 0.1% TFA (1 min), gradient 0→20% MeCN/H_2O + 0.1% TFA (1 min), gradient 20→100% MeCN/H2O + 0.1% TFA (16 min), isocratic 100% MeCN/H_2O + 0.1% TFA (1 min)). Hydroxylamines were quantified by their UV absorbance at 220 nm after normalization against the absorbance of 4-nitroaniline.

Stability in PBS: Solutions of either hydroxylamine **19** or hydroxylamine **34** (17.5 µL, 20 mM in 25% v/v methanol/PBS, pH 7.4) and 4-nitroaniline (17.5 µL, 20 mM in 25% v/v methanol/PBS, pH 7.4) were added to PBS (665 µL, pH 7.4; 500 µM final concentration). The mixture was incubated at 23 °C or 37 °C. 50 µl of the mixture was withdrawn for analysis at each time point.

Stability in DMEM and cell lysate: Solutions of either hydroxylamine **19** or hydroxylamine **34** (17.5 µL, 20 mM in 25% v/v methanol/PBS, pH 7.4) and 4-nitroaniline (17.5 µL, 20 mM in 25% v/v methanol/PBS, pH 7.4) were added to DMEM (665 µL; 500 µM final concentration) or HEK293T cell lysate (665 µL, pH 7.4, 1 mg/mL cell lysate; 500 µM final concentration). The mixture was incubated at 23 °C or 37 °C. At each time point, 100 µL of the sample was transferred to a 2 mL microcentrifuge tube, and the reaction was quenched with acetonitrile (100 µL). The mixture was centrifuged (13,000×g) at 4 °C for 5 min, then the supernatant was transferred to an HPLC vial for analysis.

Microsomal assay: A solution of human liver S9 fractions (8 µL, 20 mg/mL in phosphate buffer, pH 7.4, Gibco; 200 µg/mL final concentration) and a solution of NADPH (13.4 µL, 60 mM in 10 mM NaOH solution) were sequentially added to PBS (739 µL, pH 7.4) in a 2 mL microcentrifuge tube. The solution was incubated for 1 h at room temperature to provide solution A. Solutions of either hydroxylamine **19** or hydroxylamine **34** (20 µL, 20 mM in 25% methanol/PBS, pH 7.4; 500 µM final concentration) and 4-nitroaniline (20 µL, 20 mM in 25% v/v methanol/PBS, pH 7.4) were added to solution A. The cap of the microcentrifuge tube was pierced with a 16G needle to maintain an aerobic system. The reaction was incubated at room temperature in the dark. At each time point, 100 µL of the sample was transferred to a 2 mL microcentrifuge tube, and the reaction was quenched with acetonitrile (100 µL). The mixture was centrifuged (13,000×g) at 4 °C for 5 min, then the supernatant was transferred to an HPLC vial for analysis.

Plasmid construction

Primers

Name	Sequence
MmPylRS_vector_F	5'-GGGGATACCCTTGATGTAATGC-3'
MmPylRS_vector_R	5'-GTCAACCCTGAAGATCTGTTTTG-3'
FLuc(K529TAG)_F	5'-TAGCTCGACGCAAGAAAAATCAG-3'
FLuc(K529TAG)_R	5'-TCCGGTAAGACCTTTCGGTAC-3'
FLuc-His6_F	5'-CATCATCATCACCACCACTAATTCTAGAGGGCCCTATTCTATAGTGTC-3'
FLuc-His6_R	5'-ACTCCCTCCCACGGC-3'
sfGFP_N150TAG_F	5'-AACAGCCATTAGGTGTATATTACC-3'
sfGFP_N150TAG_R	5'-GAAATTATATTCCAGTTTATGACCC-3'

GFP_3'_R	5'-GGTCACGCCGCTTCCTCCGCTGCCTTTATACAGTTCATCC-3'
His-Tag_F	5'-CATCATCATCACCACCACTAATAGAATTGG-3'

DNA sequences

MmPylRS(AF) (for mammalian cell expression: the vector pPB was used)
atggactacaaggacgacgacgacaagatggacaagaagcccctgaacaccctgatcagcgccacaggactgtggatgtccagaaccg
gcaccatccacaagatcaagcaccacgaggtgtcccggtccaaaatctacatcgagatggcctgcggcgatcacctggtcgtcaacaaca
gcagaagcagccggacagccagagccctgcggcaccacaagtacagaaagacctgcaagcggtgcagagtgtccgacgaggacctg
aacaagttcctgaccaaggccaacgaggaccagaccagcgtgaaagtgaaggtggtgtccgcccccacccggaccaagaaagccatgc
ccaagagcgtggccagagcccccaagcccctggaaaacaccgaagccgctcaggcccagcccagcggcagcaagttcagccccgcc
atccccgtgtctacccaggaaagcgtcagcgtccccgccagcgtgtccaccagcatctctagcatctcaaccggcgccacagcttctgccc
tggtcaagggcaacaccaaccccatcaccagcatgtctgcccctgtgcaggcctctgccccagccctgaccaagtcccagaccgaccgg
ctggaagtgctcctgaaccccaaggacgagatcagcctgaacagcggcaagcccttccgggagctggaaagcgagctgctgagccggc
ggaagaaggacctccagcaaatctacgccgaggaacgggagaactacctgggcaagctggaaagagagatcacccggttcttcgtgga
ccggggcttcctggaaatcaagagccccatcctgatccccctggagtacatcgagcggatgggcatcgacaacgacaccgagctgagca
agcagattttccgggtggacaagaacttctgcctgcggcccatgctggcccccaacctggccaactacctgcggaaactggatcgcgctct
gcccgaccccatcaagattttcgagatcggcccctgctaccggaaagagagcgacggcaaagagcacctggaagagtttacaatgctgaa
cttttgccagatgggcagcggctgcaccagagagaacctggaatccatcatcaccgactttctgaaccacctggggatcgacttcaagatcg
tgggcgacagctgcatggtgttcggcgacaccctggacgtgatgcacggcgacctggaactgtctagcgccgtcgtgggacccatccctc
tggaccgggagtggggcatcgataagccctggatcggagccggcttcggcctggaacggctgctgaaagtcaagcacgactttaagaac
atcaagcgggctgccagaagcgagagctactacaacggcatctccaccaacctgtga

MmPylRS(AF) (for bacterial expression: the vector pEVOL was used)
atggataaaaaaccactaaacactctgatatctgcaaccgggctctggatgtccaggaccggaacaattcataaaataaaacaccacgaagt
ctctcgaagcaaaatctatattgaaatggcatgcggtgaccaccttgttgtaaacaactccaggagcagcaggactgcaagagcgctcagg
caccacaaatacaggaagacctgcaaacgctgcagggtttcggatgaggatctcaataagttcctcacaaaggcaaacgaagaccagaca
agcgtaaaagtcaaggtcgtttctgcccctaccagaacgaaaaaggcaatgccaaaatccgttgcgagagccccgaaacctcttgagaata
cagaagcggcacaggctcaaccttctggatctaaattttcacctgcgataccggtttccacccaagagtcagtttctgtcccggcatctgtttca
acatcaatatcaagcatttctacaggagcaactgcatccgcactggtaaaagggaatacgaaccccattacatccatgtctgcccctgttcag
gcaagtgcccccgcacttacgaagagccagactgacaggcttgaagtcctgttaaacccaaaagatgagatttccctgaattccggcaagc
ctttcagggagcttgagtccgaattgctctctcgcagaaaaaaagacctgcagcagatctacgcggaagaaagggagaattatctggggaa
actcgagcgtgaaattaccaggttctttgtggacaggggttttctggaaataaaatccccgatcctgatccctcttgagtatatcgaaaggatg
ggcattgataatgataccgaactttcaaaacagatcttcagggttgacaagaacttctgcctgagacccatgcttgctccaaaccttgccaact
acctgcgcaagcttgacagggccctgcctgatccaataaaaatttttgaaataggcccatgctacagaaaagagtccgacggcaaagaaca
cctcgaagagtttaccatgctgaacttctgccagatgggatcgggatgcacacgggaaaatcttgaaagcataattacggacttcctgaacc
acctgggaattgatttcaagatcgtaggcgattcctgcatggtcttcggggatacccttgatgtaatgcacggagacctggaactttcctctgc
agtagtcggacccataccgcttgaccgggaatggggtattgataaaccctggataggggcaggtttcgggctcgaacgccttctaaaggtta
aacacgactttaaaaatatcaagagagctgcaaggtccgagtcttactataacgggatttctaccaacctgtaa

FLuc(K529TAG) (for mammalian cell expression: the vector pcDNA3 was used)
atggaagacgccaaaaacataaagaaaggcccggcgccattctatccgctggaagatggaaccgctggagagcaactgcataaggctat
gaagagatacgccctggttcctggaacaattgcttttacagatgcacatatcgaggtggacatcacttacgctgagtacttcgaaatgtccgtt
cggttggcagaagctatgaaacgatatgggctgaatacaaatcacagaatcgtcgtatgcagtgaaaactctcttcaattctttatgccggtgtt
gggcgcgttatttatcggagttgcagttgcgcccgcgaacgacatttataatgaacgtgaattgctcaacagtatgggcatttcgcagcctacc
gtggtgttcgtttccaaaaaggggttgcaaaaaattttgaacgtgcaaaaaaagctcccaatcatccaaaaaattattatcatggattctaaaac
ggattaccagggatttcagtcgatgtacacgttcgtcacatctcatctacctcccggttttaatgaatacgattttgtgccagagtccttcgatag
ggacaagacaattgcactgatcatgaactcctctggatctactggtctgcctaaaggtgtcgctctgcctcatagaactgcctgcgtgagattc

tcgcatgccagagatcctatttttggcaatcaaatcattccggatactgcgattttaagtgttgttccattccatcacggttttggaatgtttactaca
ctcggatatttgatatgtggatttcgagtcgtcttaatgtatagatttgaagaagagctgtttctgaggagccttcaggattacaagattcaaagtg
cgctgctggtgccaaccctattctccttcttcgccaaaagcactctgattgacaaatacgatttatctaatttacacgaaattgcttctggtggcg
ctcccctctctaaggaagtcggggaagcggttgccaagaggttccatctgccaggtatcaggcaaggatatgggctcactgagactacatc
agctattctgattacacccgaggggggatgataaaccgggcgcggtcggtaaagttgttccatttttttgaagcgaaggttgtggatctggatac
cgggaaaacgctgggcgttaatcaaagaggcgaactgtgtgtgagaggtcctatgattatgtccggttatgtaaacaatccggaagcgacc
aacgccttgattgacaaggatggatggctacattctggagacatagcttactgggacgaagacgaacacttcttcatcgttgaccgcctgaag
tctctgattaagtacaaaggctatcaggtggctcccgctgaattggaatccatcttgctccaacaccccaacatcttcgacgcaggtgtcgca
ggtcttcccgacgatgacgccggtgaacttcccgccgccgttgttgttttggagcacggaaagacgatgacggaaaaagagatcgtggatt
acgtcgccagtcaagtaacaaccgcgaaaaagttgcgcggaggagttgtgtttgtggacgaagtaccgaaaggtcttaccggatagctcga
cgcaagaaaaatcagagagatcctcataaaggccaagaagggcggaaagatcgccgtg

sfGFP(N150TAG)-His6 (for bacterial cell expression: the vector pET28a was used)
atggttagcaaaggtgaagaactgtttaccggcgttgtgccgattctggtggaactggatggtgatgtgaatggccataaatttagcgttcgtg
gcgaaggcgaaggtgatgcgaccaacggtaaactgaccctgaaatttatttgcaccaccggtaaactgccggttccgtggccgaccctggt
gaccaccctgacctatggcgttcagtgctttagccgctatccggatcatatgaaacgccatgatttctttaaaagcgcgatgccggaaggctat
gtgcaggaacgtaccattagcttcaaagatgatggcacctataaaacccgtgcggaagttaaatttgaaggcgataccctggtgaaccgcat
tgaactgaaaggtattgattttaaagaagatggcaacattctgggtcataaactggaatataatttcaacagccattaggtgtatattaccgccg
ataaacagaaaaatggcatcaaagcgaactttaaaatccgtcacaacgtggaagatggtagcgtgcagctggcggatcattatcagcagaa
taccccgattggtgatggcccggtgctgctgccggataatcattatctgagcacccagagcgttctgagcaaagatccgaatgaaaaacgtg
atcatatggtgctgctggaatttgttaccgccgcgggcattacccacggtatggatgaactgtataaaggcagccaccatcatcatcaccatta
a

Synthetic genes and plasmids

All plasmids were obtained from Addgene unless otherwise stated: pEVOL-pylT-N346A/C348A (cat. #127411), Luciferase-pcDNA3 (cat. #18964), pcDNA-RLuc8 (cat. #87121), pET28a-sfGFP-His6 (cat. #85492). The synthetic gene fragment AF_insert was purchased from Twist Bioscience. The plasmid containing the MmPylRS gene with the Y306A/Y384F (AF) double mutation and optimized for mammalian cell expression, pPB-pylT-MmPylRS(AF), was a kind gift from Dr. Chayasith Uttamapinant.

AF_insert
cagatcttcagggttgacaagaacttctgcctgagacccatgcttgctccaaaccttgccaactacctgcgcaagcttgacagggccctgcct
gatccaataaaaatttttgaaataggcccatgctacagaaaagagtccgacggcaaagaacacctcgaagagtttaccatgctgaacttctgc
cagatgggatcgggatgcacacgggaaaatcttgaaagcataattacggacttcctgaaccacctgggaattgatttcaagatcgtaggcga
ttcctgcatggtcttcggggataccсttgatgtaatg

pEVOL-MmPylRS(AF)-pylT

The pEVOL-MmPylRS(AF)-pylT plasmid containing the *E. coli* codon-optimized MmPylRS gene with the Y306A/Y384F (AF) double mutation was derived from pEVOL-pylT-N346A/C348A. The AF_insert gene encoding the portion of MmPylRS with the (Y306A, Y384F) double mutation (replacing the (N346A, C348A) mutation) was synthesized from Twist Bioscience. A pEVOL vector was amplified from the pEVOL-pylT-N346A/C348A plasmid by PCR using

MmPylRS_vector_F and MmPylRS_vector_R primers. The plasmid was assembled via Gibson assembly with the AF_insert gene using the NEBuilder HiFi DNA Assembly Cloning Kit (NEB).

pcDNA3-FLuc(K529TAG)-His6, pcDNA3-FLuc(K529TAG)-RLuc

The pcDNA3-FLuc(K529TAG)-His6 plasmid was derived from Luciferase-pcDNA3 plasmid by sequentially introducing the amber mutation (TAG) and 3' His-tag using the primers (1) FLuc(K529TAG)_F and FLuc(K529TAG)_R, and (2) FLuc-His6_F and FLuc-His6_R and the Q5 Site-Directed Mutagenesis Kit (NEB). The pcDNA3-FLuc(K529TAG)-RLuc plasmid was derived from pcDNA3-FLuc(K529TAG) plasmid by amplifying the RLuc gene from pcDNA-RLuc8 and inserting it into the 3' end of the FLuc gene using the NEBuilder HiFi DNA Assembly Cloning Kit (NEB).

pET28a-sfGFP(N150COTK)-His6, pET28a-sfGFP(N150K)-His6
Both plasmids were derived from pET28a-sfGFP (Addgene). Point mutations (N150TAG and N150K) were introduced using the Q5 Site-Directed Mutagenesis Kit (NEB).

Expression of sfGFP(N150COTK)-His6 with unnatural amino acid incorporation.

The plasmid pET28a-sfGFP(N150TAG)-His6 was co-transformed with pEVOL-pylT-MmPylRS(AF) into chemically competent BL21(DE3) *E. coli* cells (NEB) and selected on dual selection LB/agar plates containing kanamycin and chloramphenicol. After incubating the plates overnight at 37 °C, a single colony was taken and used to inoculate LB media (50 mL) containing kanamycin (50 µg/mL) and chloramphenicol (35 µg/mL) in a 125 mL Erlenmeyer flask. The starter culture was grown overnight until saturation (37 °C, 200 rpm). LB media (250 mL) containing kanamycin (50 µg/mL) and chloramphenicol (35 µg/mL) was inoculated with starter culture (10 mL) and grown at 37 °C for 2 h to reach OD_{600} = 0.6−0.8. Unnatural amino acid COTK (**35**) or TCOK (50 mM in 0.1 M NaOH, final concentration 1 mM) was added to the culture and incubated at 37 °C for 30 min. Protein expression was induced by adding IPTG (1 mM final concentration) and *L*-arabinose (0.2% w/v, final concentration). After induction, the cultures were incubated with shaking for 20 h at 16 °C. Cells were pelleted by centrifugation (5000×g, 30 min, 4 °C). The pellet was collected, flash-frozen in liquid N_2, and stored at –80 °C.

Expression of the non-caged control sfGFP(N150K)-His6 was conducted in parallel. Briefly, the plasmid pET28a-sfGFP(N150K)-His6 was transformed into chemically competent BL21(DE3) *E. coli* cells. Inoculation of a starter culture and overnight growth was the same as previously described above. However, induction was performed simply by adding only IPTG (1 mM final concentration). After induction, the cultures were incubated with shaking for 20 h at 16 °C. Cells were pelleted by centrifugation (5000×g, 30 min, 4 °C). The pellet was collected, flash-frozen in liquid N_2, and stored at –80 °C.

Purification of sfGFP(N150COTK)-His6, **sfGFP(N150K)-His6**

Cell pellets obtained from the expression of sfGFP(N150COTK)-His6 or sfGFP(N150K)-His6 were suspended in 20 mL lysis buffer (50 mM NaH_2PO_4, 300 mM NaCl, 10 mM imidazole, 1 mg/mL lysozyme, 1 mM PMSF, cOMPLETE protease inhibitor tablet, 25 units/mL benzonase, pH

8.0), and lysed by sonication on ice. Cell lysates were clarified by centrifugation (15000×g, 30 min, 4 °C) and the clarified lysate was subsequently purified by a His-Tag affinity column (GE HisTrap FF crude 5 mL, Buffer A : pH 8.0, 50 mM NaH_2PO_4, 300 mM NaCl, 10 mM imidazole, Buffer B : pH 8.0, 50 mM NaH_2PO_4, 300 mM NaCl, 500 mM imidazole). Fractions containing the His-tagged protein was collected, dialyzed into PBS (pH 7.4) overnight using SnakeSkin 10K MWCO Dialysis Tubing (Thermo Fisher Scientific, Waltham, MA), and concentrated using 10 kDa MWCO Amicon Ultra Centrifugal Filter Units (Thermo Fisher Scientific, Waltham, MA). The protein was further purified by size exclusion column chromatography with PBS, pH 7.4. Fractions containing protein were collected and concentrated using 10 kDa MWCO Amicon Ultra Centrifugal Filter Units (Thermo Fisher Scientific, Waltham, MA). The protein solution was aliquoted, flash frozen in liquid N_2, and stored at –80 °C.

Uncaging of recombinant sfGFP(N150COTK)-His6 and TAMRA-chase assay

In a clean microcentrifuge tube, 120 µL of NEBExpress Ni-NTA magnetic bead slurry (New England Biolabs) was added and washed (3 × 500 µL PBS, pH 7.4). A solution of recombinant sfGFP(N150COTK)-His6 (360 µL, 10 µM in PBS, pH 7.4) or sfGFP(N150K)-His6 was added and the resulting suspension was incubated for 30 min at room temperature. The suspension was then distributed to clean microcentrifuge tubes (30 µL per tube), and a 2× solution of hydroxylamine **34** (30 µL, concentration starting at 2 mM, from a 500 mM stock in DMSO and subsequently diluted with PBS, pH 7.4) or vehicle control (30 µL, 0.4% DMSO/PBS) was added to each tube. After 1 h, the reaction medium was removed and the beads were washed with PBS, pH 7.4 (2 × 500 µL). Proteins were eluted by incubating the beads with elution buffer (48 µL, 500 mM imidazole in PBS, pH 7.4) at room temperature for 5 min. Next, 5× sample loading buffer (10 µL) was added to the eluents and then subsequently boiled at 95 °C for 5 min. Once the sample has cooled back down to room temperature, a solution of TAMRA-hydroxylamine **39** (2 µL, 100 µM final concentration, from a stock of 3 mM in 30% EtOH/PBS) was added to each sample and incubated for 1 h at room temperature in the dark. Samples were then loaded directly onto a Bio-Rad Mini-PROTEAN TGX Precast Protein 15-well gel, 4–20% (10 µL) alongside All Blue Prestained Protein Standard (Bio-Rad, 5 µL) as ladder and run in freshly prepared Tris-glycine running buffer (180 V, 45 min). In-gel fluorescence imaging was performed on a Typhoon FLA 9500 (GE) at 532 nm with photomultiplier tube (PMT) setting of 500 V. Pixel densitometry was performed using Image Studio Lite V. 5.2.5 (Licor) and normalized to the band with the highest density within each blot.

Uncaging of recombinant sfGFP(N150COTK)-His6 – intact mass spectrometry analysis.

To a microcentrifuge tube, NEBExpress Ni-NTA magnetic bead slurry (50 µL, New England Biolabs) was added and washed (3 × 500 µL PBS, pH 7.4). Next, a solution of recombinant sfGFP(N150COTK)-His6 (100 µL, 20 µM in PBS, pH 7.4) was added and the resulting suspension was incubated for 30 min at room temperature with end-over-end rotation. The suspension was then distributed to two microcentrifuge tubes (75 µL per tube), and a solution of hydroxylamine **34** (75 µL, 1 mM final concentration, from a 500 mM stock in DMSO and subsequently diluted with PBS, pH 7.4) was added to one tube and vehicle control (75 µL, 0.4% DMSO in PBS) was added to the other. After 1 h incubation, the reaction media was removed and the beads were washed with PBS (2 × 500 µL). The His-tagged sfGFP protein was then eluted by incubating the

beads with elution buffer (100 μL, 500 mM imidazole in PBS, pH 7.4) at room temperature for 5 min. Subsequently, the eluents were each diluted with PBS, pH 7.4 (400 μL) and concentrated via spin filtration (Amicon Ultra-0.5 mL, UFC500324, 10 kDa MWCO) following the manufacturer's recommended protocol. PBS, pH 7.4 was added after each filtration cycle and the spin filtration was repeated for a total of five times. The concentrated protein was then collected in a clean microcentrifuge tube and its concentration determined by NanoDrop (Thermo Fisher Scientific). Samples were then sent to Georgia Tech's Systems Mass Spectrometry Core (SyMS-C)for LC-ESI-MS analysis.

LC-ESI-MS analysis of proteins
The samples of recombinant sfGFP(N150COTK) were prepared in a buffer consisting of 10% acetonitrile (ACN) and 0.1% formic acid (FA) and introduced into the source via a syringe pump at a constant flow rate of 3 uL/min. Data were acquired in positive ion mode (FTMS + p ESI) over a scan range of 800–2000 m/z. The instrument was operated at an Orbitrap resolution of 240,000 with a microscan count of 10 to enhance the signal-to-noise ratio. The Automatic Gain Control (AGC) target was set to 5e6 with a maximum injection time of 200 ms. Total acquisition time per sample was 1 minute. Data analysis was performed using Thermo Scientific FreeStyle software.

Luciferase expression and uncaging in HEK293T cells

Cell culture: HEK293T cells (ATCC) were cultured in DMEM containing 10% FBS (Sigma), 100 units/mL penicillin, and 0.1 mg/mL streptomycin (Sigma) in a humidified chamber at 37 °C under an ambient atmosphere with 5% CO_2. Cells were passaged and dissociated with 0.25% trypsin, 0.1% EDTA in HBSS (Corning). All cells tested negative for mycobacteria with the MycoAlert PLUS Mycoplasma Detection Kit (Lonza) following the manufacturer's protocol.

Cell viability assay: HEK293T cells in DMEM supplemented with 10% FBS (Sigma), penicillin (100 units/mL), streptomycin (0.1 mg/mL) were seeded at a density of 5,000 cells per well in an opaque, white-wall, clear-bottom 96-well plate precoated with poly-*D*-lysine (100 μL). PBS, pH 7.4 (100 μL) was added to the edge wells. The cells were incubated in a humidified chamber at 37 °C under an ambient atmosphere with 5% CO_2. After 24 h, the media was removed and replaced with complete growth media containing hydroxylamine **19** or **34** with 2.5-fold serial dilution across nine wells, starting from 2 mM (100 μL, 0.5% DMSO in complete growth media) with the tenth well containing vehicle control (0.5% DMSO). The plates were incubated at 37 °C for 3 h. After 3 h, the media was removed, and cells were washed once with media. Wells were replaced with complete growth media (100 μL) and the cells were further incubated for another 21 h. Then, the plates were equilibrated to room temperature and CellTiter-Glo 2.0 reagent (50 μL, Promega) was added to each well and mixed gently. The plates were incubated at room temperature for 10 min after which the luminescence signal was measured by a microplate reader (Clariostar Plus, BMG Labtech).

Uncaging of firefly luciferase in cells; Dual-Glo luciferase assay analysis. HEK293T cells were seeded at a density of 4.0×10^5 cells/mL in 12-well plates (1 mL/well). Cells were cultured in DMEM containing 10% FBS (Sigma), 100 units/mL penicillin, and 0.1 mg/mL streptomycin (Sigma) in a humidified chamber at 37 °C under an ambient atmosphere with 5% CO_2. After 24 h,

the cells were co-transfected with two plasmids: pPB-MmPylRS(Y306A,Y384F)-PylT and pcDNA3-FLuc(K529TAG)-RLuc. Transient transfection was performed using the TransIT-293 transfection reagent (Mirus) following the manufacturer's protocol. After 6 h post-transfection, a solution of COTK (**38**) or TCOK (**41**) was added directly to the culture media (1 mM final concentration, diluted with sterile water from a 50 mM stock in sterile 0.1 M NaOH). After 24 h post-transfection, cells were trypsinized then resuspended in complete growth media supplemented with 1 mM COTK (**38**) or TCOK (**41**) (1.1 mL/well). From each well, the resuspended cells were then transferred to a clear, flat-bottom 96-well plate precoated with poly-*D*-lysine (100 µL per well). After incubating the transferred cells for an additional 12 h, the media was removed and cells were washed once with PBS (pH 7.4), and treated with serum-free DMEM media supplemented with penicillin (100 units/mL), streptomycin (0.1 mg/mL), and hydroxylamine **19** or **34** (for COTK-supplemented cells) or 3,6-dimethyl-1,2,4,5-tetrazine (**40**) (for TCOK-supplemented cells) (various concentrations, prepared via serial dilution starting from 2 mM in 0.5% DMSO) or vehicle control (0.5% DMSO). After incubating the treated cells for 3 h at 37 °C, the media was removed and cells were washed twice with PBS (pH 7.4). PBS (60 µL) was added to each well and firefly luciferase activity and *Renilla* luciferase activity were sequentially measured using the Dual-Glo luciferase assay system (Promega) following the manufacturer's protocol. Bioluminescence was measured using a microplate reader (Clariostar Plus, BMG Labtech) and gain was adjusted prior to the measurement. The experiment was performed in three biological replicates.

Time-dependent uncaging of firefly luciferase in cells; Dual-Glo luciferase assay analysis.
HEK293T cells were seeded at a density of 4.0×10^5 cells/mL in 12-well plates (1 mL/well). Cells were cultured in DMEM containing 10% FBS (Sigma), 100 units/mL penicillin, and 0.1 mg/mL streptomycin (Sigma) in a humidified chamber at 37 °C under an ambient atmosphere with 5% CO_2. After 24 h, the cells were co-transfected with two plasmids: pPB-MmPylRS(Y306A,Y384F)-PylT and pcDNA3-FLuc(K529TAG)-RLuc. Transient transfection was performed using the TransIT-293 transfection reagent (Mirus) following the manufacturer's protocol. After 6 h post-transfection, a solution of COTK (**38**) was added directly to the culture media (1 mM final concentration, diluted with sterile water from a 50 mM stock in sterile 0.1 M NaOH). After 24 h post-transfection, cells were trypsinized then resuspended in complete growth media supplemented with 1 mM COTK (**38**) (1.1 mL/well). From each well, the resuspended cells were then transferred to a clear, flat-bottom 96-well plate precoated with poly-*D*-lysine (100 µL per well). After incubating the transferred cells for an additional 12 h, the media was removed and cells were washed once with PBS (pH 7.4), and treated with serum-free DMEM media supplemented with penicillin (100 units/mL), streptomycin (0.1 mg/mL). Incubating the cells at 37 °C ,and at designated time points prior to the endpoint (0 min, 2 h, 4 h, 5 h, 5.5 h, 5 h 50 min, 5 h 55 min for **19**), the media in the assigned wells was replaced with serum-free DMEM media supplemented with penicillin (100 units/mL), streptomycin (0.1 mg/mL), and 100 µM **19**. After 24 h incubation, the media was removed and cells were washed twice with PBS (pH 7.4). PBS (60 µL) was added to each well and firefly luciferase activity and *Renilla* luciferase activity were sequentially measured using the Dual-Glo luciferase assay system (Promega) following the manufacturer's protocol. Bioluminescence was measured using a microplate reader (Clariostar Plus, BMG Labtech) and gain was adjusted prior to the measurement. The experiment was performed in three biological replicates.

<u>Uncaging of firefly luciferase in cells and intact mass spectrometry analysis.</u> HEK293T cells were cultured in DMEM containing 10% FBS (Sigma), 100 units/mL penicillin, and 0.1 mg/mL streptomycin (Sigma) in a humidified chamber at 37 °C under an ambient atmosphere with 5% CO_2. After culturing to >80% confluency in 100 mm plates, cells were co-transfected with two plasmids: pPB-MmPylRS(Y306A,Y384F)-PylT and pcDNA3-FLuc(K529TAG)-His6**.** Transient transfection was performed using the TransIT-293 transfection reagent (Mirus) following the manufacturer's protocol. After 6 h post-transfection, a solution of COTK (**38**) or TCOK (**41**) was added directly to the culture media (1 mM final concentration, diluted from a 50 mM stock in sterile 0.1 M NaOH). After 36 h post-transfection, the media was removed and cells were washed once with PBS (pH 7.4), and treated with serum-free DMEM media supplemented with penicillin (100 units/mL), streptomycin (0.1 mg/mL), and hydroxylamine **19** or **34** (for COTK-supplemented cells) or 3,6-dimethyl-1,2,4,5-tetrazine (**40**) (for TCOK-supplemented cells) (2 mM in 0.5% DMSO) or vehicle control (0.5% DMSO). After incubating the treated cells for 3 h at 37 °C, the media was removed and cells were washed twice with PBS (pH 7.4) then lysed with cold NP40 lysis buffer supplemented with PMSF (1 mM), imidazole (10 mM), and unnatural amino acids **38** or **41** (1 mM) (amount of lysis buffer: 1 mL per plate) then transferred to microcentrifuge tubes on ice. The cell lysate was spun down (14000×g, 15 min, 4 °C), and then the His-tagged FLuc protein was purified with NEBExpress Ni-NTA magnetic beads (NEB) following the manufacturer's protocol. After eluting the protein from the beads with NP40 lysis buffer containing 500 mM imidazole, the protein solution was buffer-exchanged into PBS, pH 7.4 using 10K MWCO Amicon Ultracentrifugal Filter Units (Thermofisher Scientific). The samples were flash-frozen in liquid nitrogen and sent to the Emory Glycomics and Molecular Interactions Core for LC-ESI-MS analysis

<u>*LC-ESI-MS analysis of proteins*</u>: LC-ESI-MS analysis was conducted on an Agilent 1290 Infinity II LC system coupled to an Agilent 6545XT AdvancedBio Q-TOF system with a Dual Agilent Jet Stream source. Samples (2 µL) were first resolved with an Agilent PLRP-S column heated to 60 °C (cat# 1912-1502, 1000 Å C18, 2.1 × 50 mm, 5 µm particles, 0.4 mL/min flow rate) using a 5 min elution gradient (gradient 2→20% MeCN + 0.1% TFA (0–1.25 min), gradient 20→50% MeCN + 0.1% TFA (1.25–2.6 min), isocratic 50% MeCN + 0.1% TFA (2.6–3 min), gradient 50%→90% MeCN + 0.1% TFA (3–4 min), isocratic 2% MeCN + 0.1% TFA (4–5 min)) before injecting to Q-TOF (ESI). The MS acquisition parameters are summarized in **Table S1** below:

**Table S1:** MS Acquisition Parameters, Agilent 6545XT AdvancedBio LC/Q-TOF System	
Gas Temp (°C)	365
Drying Gas (L/min)	12
Nebulizer (psi)	35
Sheath Gas Temp (°C)	300
Sheath Gas Flow (L/min)	12
VCap (V)	5500
Nozzle Voltage (Expt) (V)	2000
Fragmentor (V)	300
Skimmer (V)	220
Oct 1 RF Vpp (V)	750
Mode	MS, positive
Mass Range	100-10000

Acquisition Rate (spectra/s)	1
Acquisition Time (ms/spectrum)	1000
Acquisition Mode	Extended (10,000) mass range, 2 GHz

Data processing was conducted using MassHunter BioConfirm v10.0 software and the protein deconvolute workflow. From the total ion chromatogram, the range of the major elution peak was selected, and the corresponding raw mass spectrum and deconvoluted mass spectrum (generated from the selected scans using the maximum entropy algorithm and converting the charge-state distribution into a zero-charge mass spectrum) were obtained from 59000 Da–63000 Da.

Uncaging of firefly luciferase in cells and LC-MS/MS analysis of digested proteins.
Sample preparation: HEK293T cells were cultured in DMEM containing 10% FBS (Sigma), 100 units/mL penicillin, and 0.1 mg/mL streptomycin (Sigma) in a humidified chamber at 37 °C under an ambient atmosphere with 5% CO_2. After culturing to >80% confluency in 100 mm plates, cells were co-transfected with two plasmids: pPB-MmPylRS(Y306A,Y384F)-PylT and pcDNA3-FLuc(K529TAG)-His6**.** Transient transfection was performed using the TransIT-293 transfection reagent (Mirus) following the manufacturer's protocol. After 6 h post-transfection, a solution of fCOTK (**38**) was added directly to the culture media (1 mM final concentration, diluted from a 50 mM stock in sterile 0.1 M NaOH). After 36 h post-transfection, the media was removed and cells were washed once with PBS (pH 7.4), and treated with serum-free DMEM media supplemented with penicillin (100 units/mL), streptomycin (0.1 mg/mL), and hydroxylamine **19** or vehicle control (0.5% DMSO). After incubating the treated cells for 3 h at 37 °C, the media were removed, and the cells were washed twice with PBS (pH 7.4) and dissociated with trypsin (37 °C, 5 min). Finally, complete growth medium was added to quench the trypsin and the cell pellet was collected. Cell pellets were centrifuged twice at 500×g for 5 min.

Cell pellets were then lysed using cold M-PER lysis buffer (0.5 mL, Thermofisher, cat# 78501) containing 1 mM PMSF and cOmplete EDTA-free protease inhibitor cocktail (Roche). The lysate was centrifuged (17,000×g, 10 min, 4 °C) and the protein concentration in the supernatant was determined using the BCA assay (Thermofisher). Subsequently, to 1 mg of lysate (2 mg/mL) in lysis buffer was added HisPur™ Ni-NTA magnetic beads (50 µL, Thermofisher) and the samples were incubated further at room temperature for 30 min. Next, tris(2-carboxyethyl)phosphine (TCEP, 10 mM final) was added to the suspension and incubated for 30 min, followed by iodoacetamide (15 mM final) and incubated for 45 min for on-bead reduction/alkylation. The supernatant was removed, and beads were washed with TBS-T, pH 8 containing 20 mM imidazole (3 × 1 mL) and Milli-Q water (2 × 1 mL). The beads were resuspended in 50 mM TEAB, pH 8 (300 µL) after which mass spectrometry grade trypsin (2.5 µg, Promega) was added. The beads were incubated for 16 h at 37 °C with shaking (800 rpm) after which the supernatant (containing digested peptides) was subjected to desalting using C18 ZipTips (Thermofisher) following the manufacturer's protocol. The elutions were vacuum-centrifuged to dryness before proceeding to LC-MS/MS analysis.

Data acquisition: Prior to LC-MS/MS analysis, dried samples were reconstituted in 2% acetonitrile (MeCN), 0.2% formic acid (FA) solution (30 µL), sonicated for 3 min, and centrifuged at 20,000×g for 1 min. The peptide concentration was then determined using the Pierce™ Quantitative Colorimetric Peptide Assay (Thermofisher). Subsequently, peptides (normalized to 0.6 µg/µL) was

then transferred to LCMS-vials, and 5 μL (containing 3 μg peptides) was subsequently loaded onto a Vanquish Neo UHPLC system (ThermoFisher) coupled to an Orbitrap Eclipse mass spectrometer (ThermoFisher). Peptides were separated on a Waters nanoEase column heated to 50 °C (cat# 186009259, 130 Å C18, 300 μm × 150 mm, 1.7 μm particles, 4 μL/min flow rate) using a 130-minute elution gradient (gradient 2%→35% MeCN + 0.2% FA (3.5−123.5 min), gradient 35%→60% MeCN + 0.2% FA (123.5–124 min), isocratic 60% MeCN + 0.2% FA (124–127 min), isocratic 100% MeCN + 0.2% FA (127–130 min, wash)). MS1 data were acquired in Orbitrap mode with a resolution of 120,000, standard AGC target, and auto maximum injection time. Charge states from 2+ to 7+ were included, and a dynamic exclusion time of 30 seconds was used. MS2 scans were isolated with the quadrupole and fragmented using HCD with a fixed collision energy of 30% and a 1.6 m/z isolation window. The normalized AGC target for MS2 was set to 250%, and fragment ions were detected in the Orbitrap at a resolution of 15,000 with a defined first mass of m/z 100.

LC-MS/MS data processing and analysis: The raw data was analyzed by Proteome Discoverer v2.5 (ThermoFisher). Sequest HT was employed for peptide identification searches against firefly luciferase containing a *C*-terminal His6-tag. The search parameters allowed for a maximum of two missed cleavages and peptides with a minimum length of 6 amino acids. Precursor and fragment mass tolerances were set to 10 ppm and 0.01 Da, respectively. Dynamic modifications included methionine oxidation, *N*-terminal acetylation, *N*-terminal methionine loss, and the addition of the cyclooctynyl-caged lysine (COTK, 150.0681 Da). Cysteine alkylation was set as a static modification. Peptide-spectrum match (PSM) validation was performed using the Target Decoy PSM Validator with a strict false discovery rate (FDR) of 0.01. For quantification, area under the curve (AUC) of the precursor ion (MS1) was used to calculate peptide abundance.

Molecular docking of caged lysine adducts with small molecule triggers

Molecular docking was performed using Molecular Operating Environment (MOE 2022, v2.5). The crystal structure of firefly luciferase complexed to DLSA (PDB: 4G36) was loaded from the Protein Data Bank and prepared using the “Quickprep” function. Ligands were drawn in ChemDraw and their 3D structure was generated, energy-minimized, and saved to MOE as a new database. The van der Waals volume was calculated for each ligand using the “Calculate Descriptors” tool in the database.

The reactive site (K529) was temporarily mutated to a cysteine, and the thiol was selected to undergo docking via the beta-mercapto carbonyl 1,4-addition reaction template with the database ligands (modified appropriately to contain a Michael acceptor). Docking was performed using the “rigid receptor” refinement with the Triangle Matcher algorithm, Amber10:EHT force field, London dG scores for placement, and GBVI/WSA dG scores for refinement. The pose with the lowest S score was selected and presented for each docked ligand. The native lysine was restored, and atoms were edited using the Builder tool to reflect the correct atomic connectivity in the adduct. The docked structure was energy-minimized (Amber10:EHT force field) to furnish the final docked pose. The interaction (and interaction energies) between docked ligands and the protein was analyzed using the Ligand_Interactions and Contacts modules. The final figures were generated in Pymol from docking poses exported as PDB files. For illustrative purposes in the figure panels, the luciferin/ATP binding site was defined and highlighted as residues 244–252, 310–319, 337–355.[1]

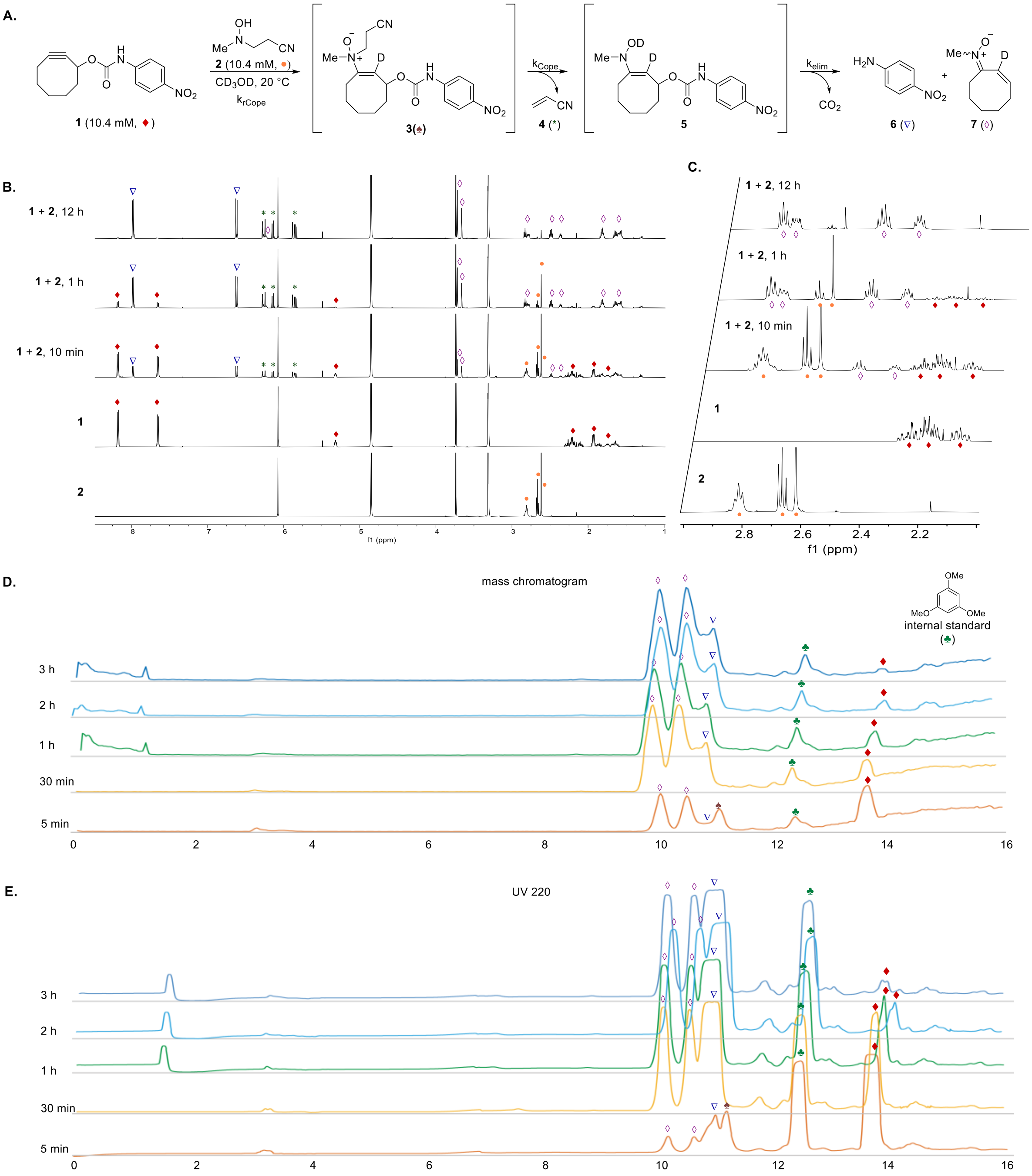

Figure S1. Characterization of the tandem retro-Cope/Cope elimination reaction by ^{1}H NMR and LCMS. (A) General scheme for the retro-Cope elimination/Cope elimination of cyclooctynyl carbamate **1** and *N*-cyanoethyl-*N*-methylhydroxylamine (**2**). (B) Progress of the reaction between 10.4 mM of **1** and **2** in CD_3OD was monitored by ^{1}H NMR over 12 h. (C) An inset of the ^{1}H NMR spectrum in panel B with offset axis for clarity. (D) Progress of the reaction was monitored by LCMS over 3 h. The mass chromatogram is displayed with the y-axis representing the relative total ion count. Peak assignments indicate consistency of the mass-to-charge ratio of the major ion present in the peak with the presence of the indicated species. (E) UV chromatogram from the LCMS-based reaction monitoring experiment with the y-axis representing absorbance at 220 nm.

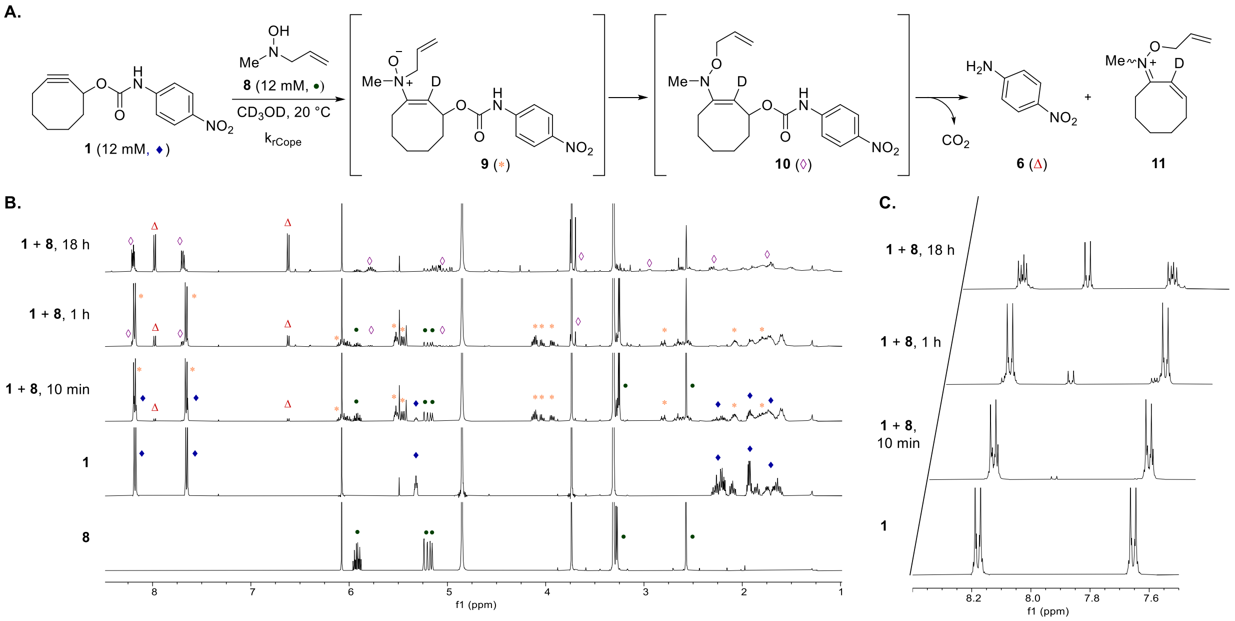

Figure S2. Characterization of the tandem retro-Cope/[2,3]-Meisenheimer rearrangement reaction by ^{1}H NMR. (A) General scheme for the reaction between cyclooctynyl carbamate **1** and *N*-allyl-*N*-methylhydroxylamine (**8**). (B) Progress of the reaction between 12 mM cyclooctynyl carbamate **1** and hydroxylamine **2** in CD_3OD was monitored by ^{1}H NMR spectroscopy over 18 h. (C) An inset of the ^{1}H NMR spectrum in panel B with offset axis for clarity.

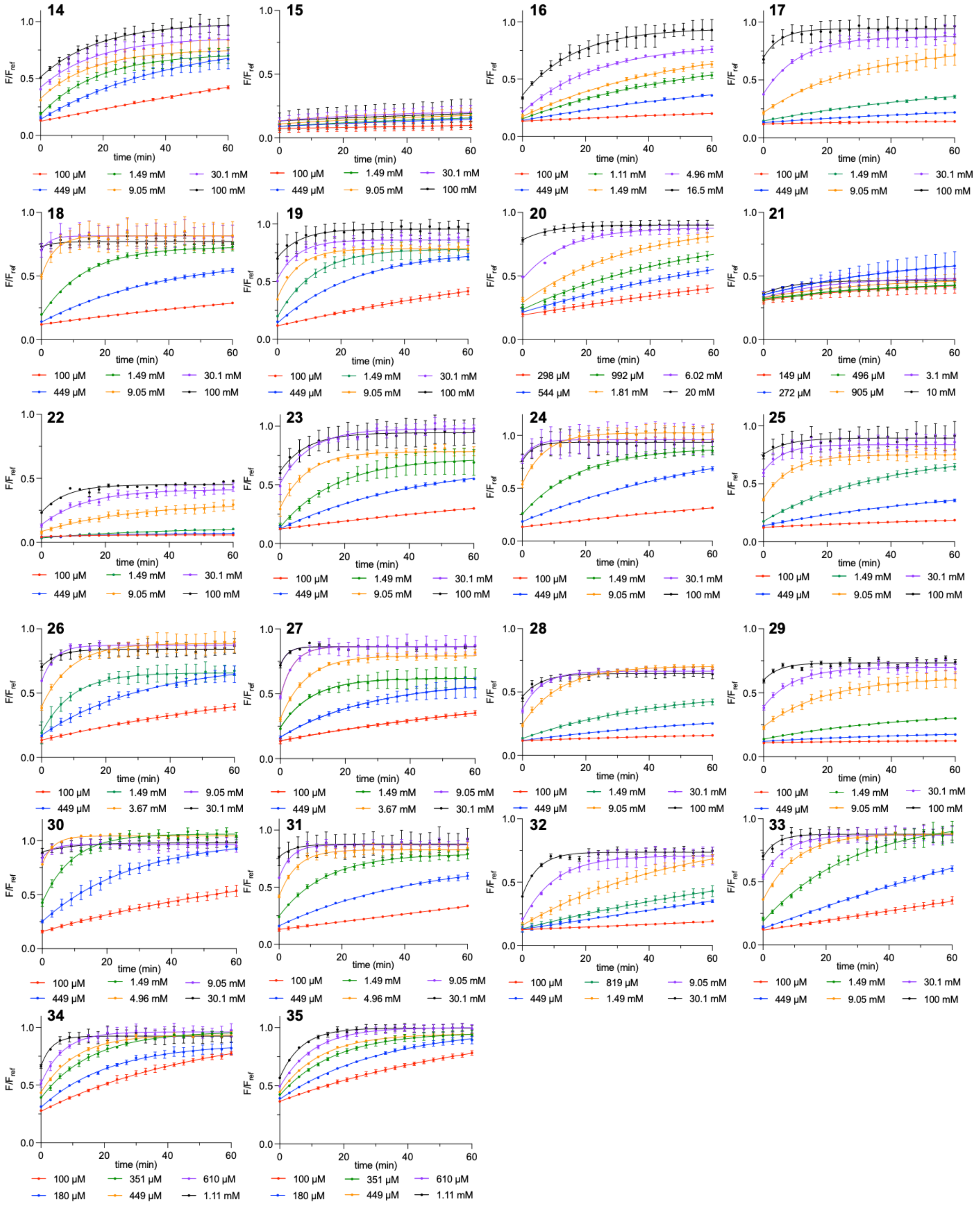

Figure S3. Plots showing fluorescence uncaging over time for hydroxylamines **14−35** based on the rhodamine fluorescence assay described in **Figure 5**. For clarity, only six concentrations are shown and includes the lowest and highest concentrations tested. Error bars represent standard deviation from three replicate experiments.

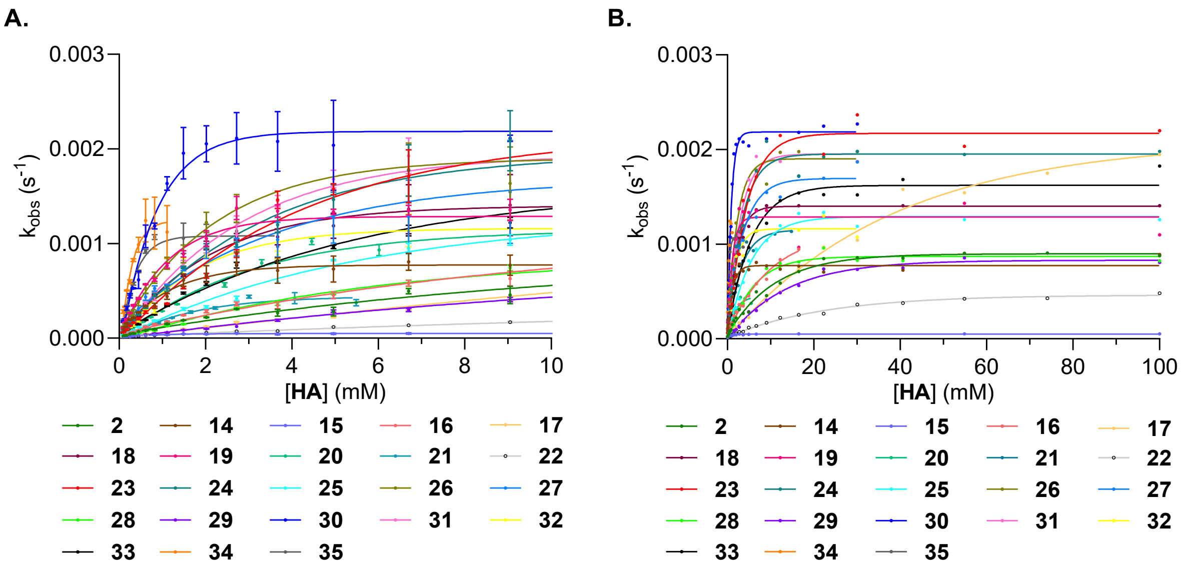

Figure S4. Plots of k_{obs} over hydroxylamine (HA) concentration for hydroxylamines **2**, **14−35** as determined by the rhodamine fluorescence assay described in **Figure 5**. Due to solubility constraints, k_{obs} measurements at higher concentrations could not be determined for certain hydroxylamines. A) *x*-axis range from 0.1–10 mM, B) *x*-axis range from 0.1–100 mM. Values are an average of three replicates. In B), the error bars are omitted for clarity.

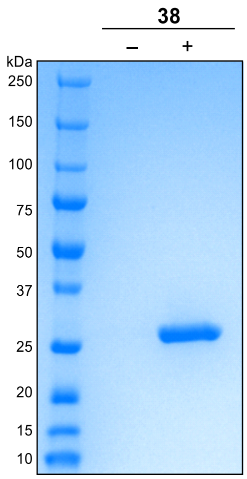

Figure S5. Full Coomassie stain of recombinant sfGFP(N150COTK), expressed with or without addition of 1 mM cyclooctynyl lysine (COTK, **38**) as depicted in Figure 7B. 1 mL aliquots of clarified lysates from bacterial cultures expressed with or without **38** were separately purified and the purified proteins were then subjected to SDS-PAGE.

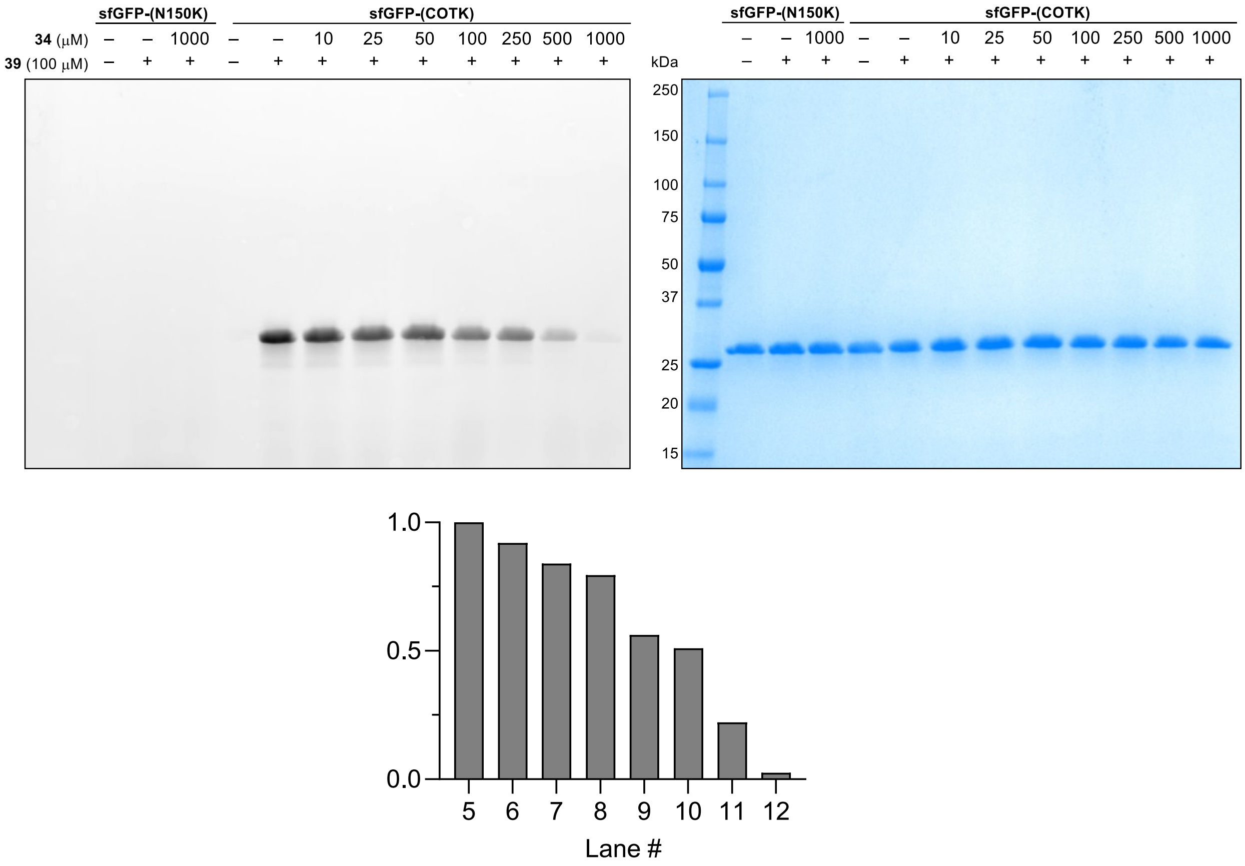

Figure S6. Full in-gel fluorescence and Coomassie stain images for Figure 7D. Both images were taken from the same gel. Densitometry measurements for the in-gel fluorescence are also included and are normalized to lane 5.

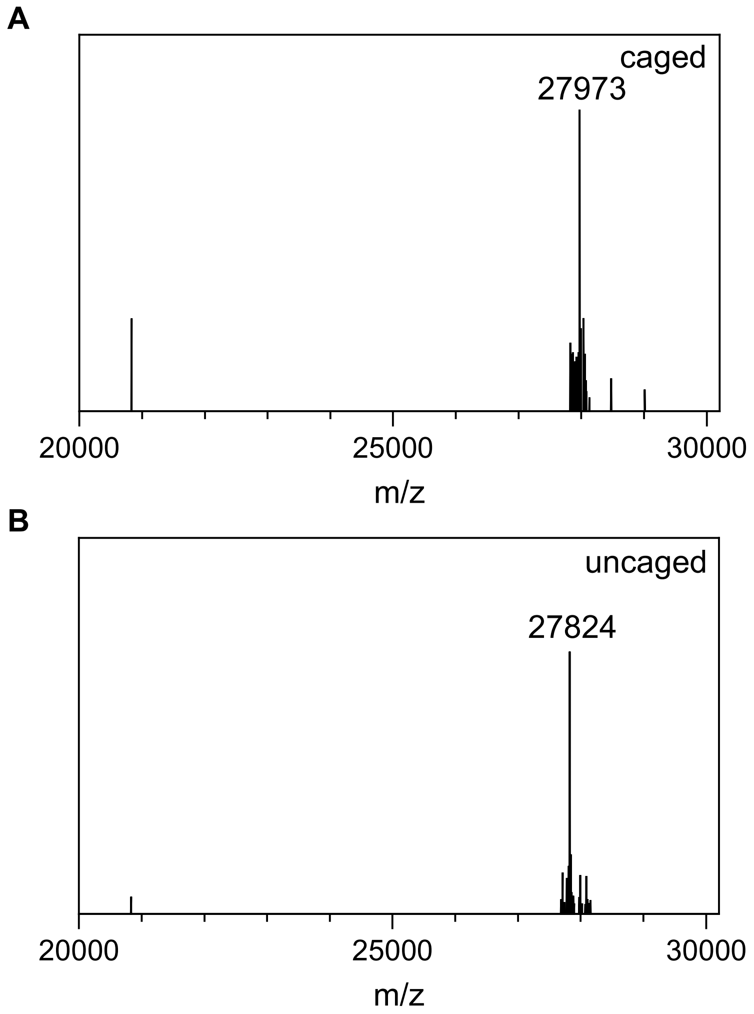

Figure S7. (A) Full intact mass spectrometry spectrum of cyclooctyne-caged sfGFP. (B) Full intact mass spectrometry spectrum of cyclooctyne-caged sfGFP with addition of 1 mM **34** for 1 h.

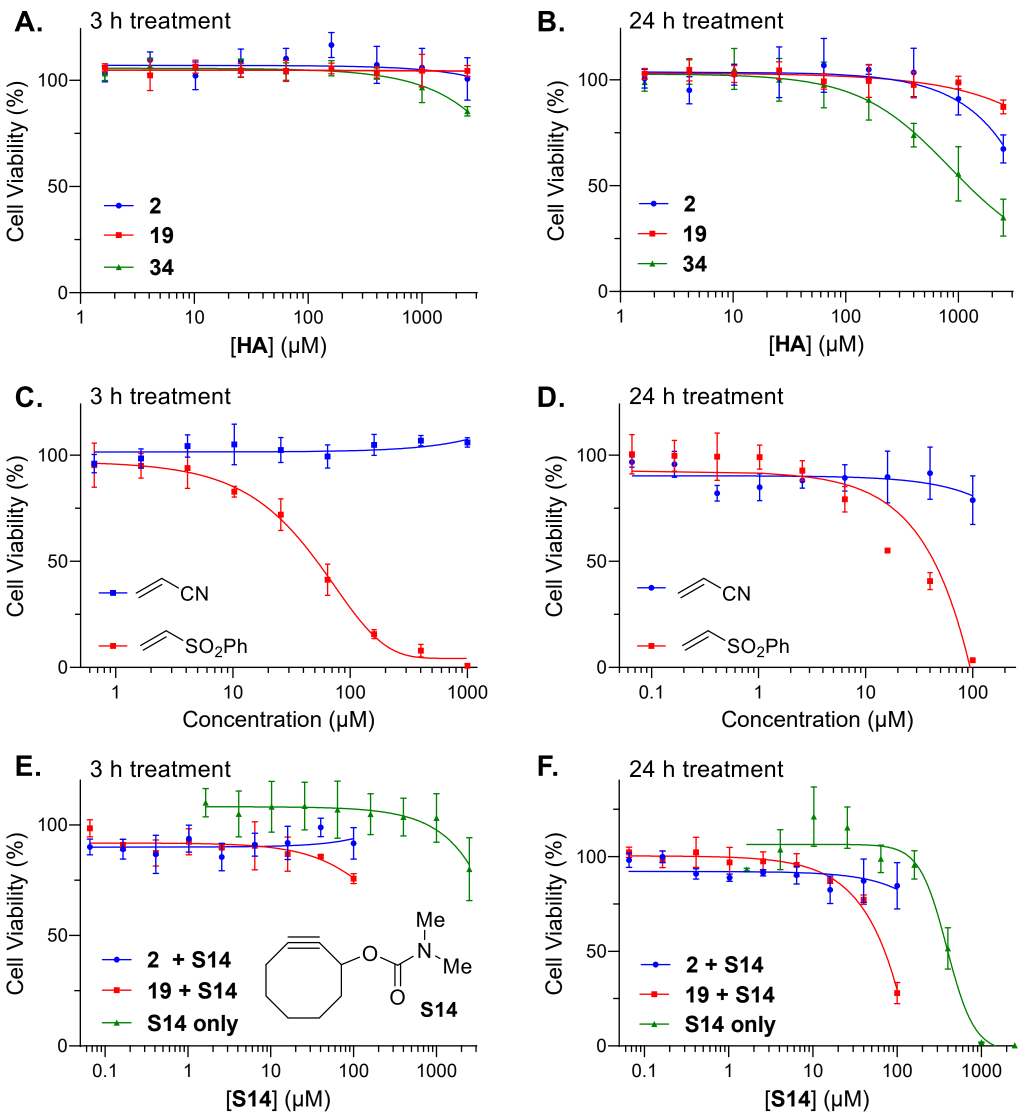

Compound / condition	IC_{50} (µM)
**34**, 24 h	847
Phenyl vinyl sulfone, 3 h	57
Phenyl vinyl sulfone, 24 h	36
**19** + **S14**, 24 h	68
**S14** only, 24 h	341

Figure S8. Dose response curves from cell viability assays in HEK293T cells. Where applicable, IC_{50} values are reported. (A,B) Hydroxylamines **2**, **19**, and **34** were treated in cells for 3 h (A) or 24 h (B). (C,D) The electrophile acrylonitrile or phenyl vinyl sulfone was treated in cells for 3 h (C) or 24 h (D). (E,F) Cyclooctynyl carbamate **S14** was treated in cells with or without a fixed concentration of hydroxylamine **2** or **19** (100 µM) for 3 h (E) or 24 h (F). Error bars represent mean ± SEM of data from biological replicates (n=3). HA = hydroxylamine.

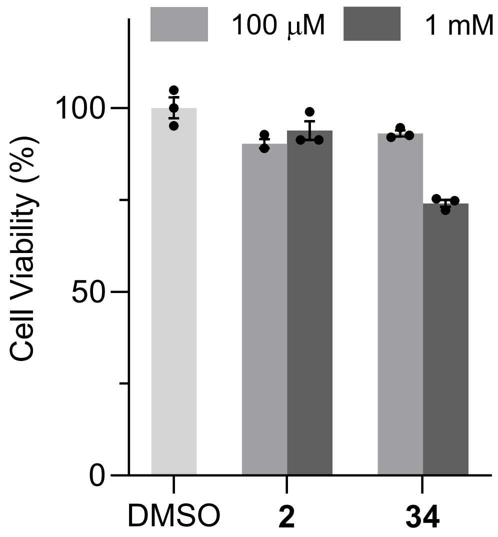

Figure S9. Cell viability in HEK293T cells determined 3 h post-luciferase activation with hydroxylamines **2** and **34**.

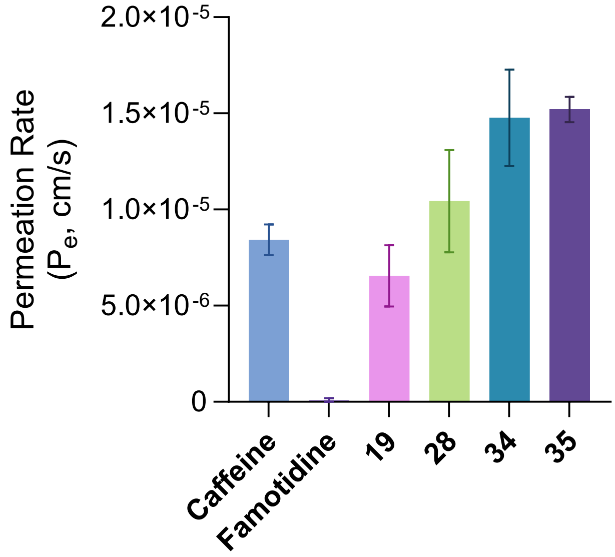

Figure S10. Membrane permeation rate of hydroxylamines **19, 28, 34, 35**, and two reference standard compounds, famotidine (negative control) and caffeine, as determined by the PAMPA assay. Error bars represent the standard deviation from triplicate experiments.

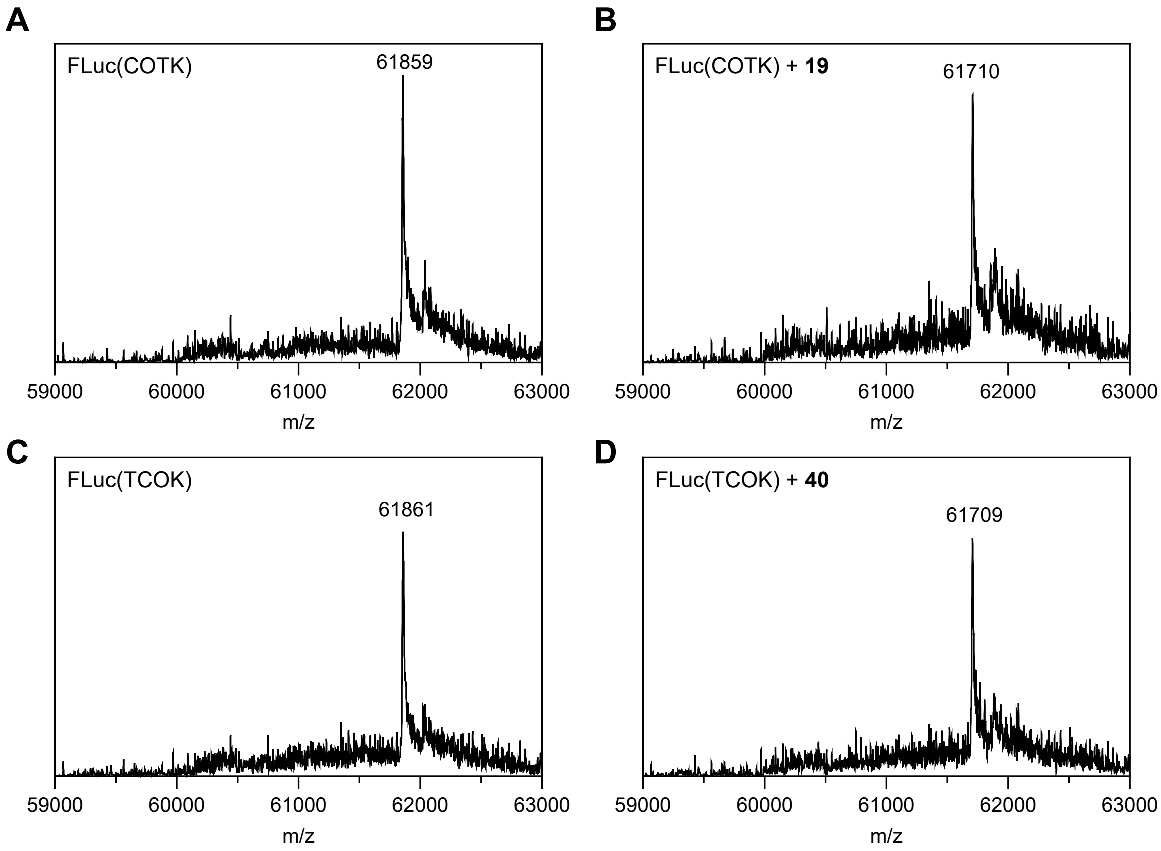

Figure S11. Full intact mass spectrometry spectra of firefly luciferase expressed and uncaged in HEK293T cells. (A,B) FLuc caged with COTK before (A) and after (B) treatment of 100 μM hydroxylamine **19** for 3 h. (C,D) FLuc caged with TCOK before (C) and after (D) treatment of 100 μM tetrazine **40** for 3 h.

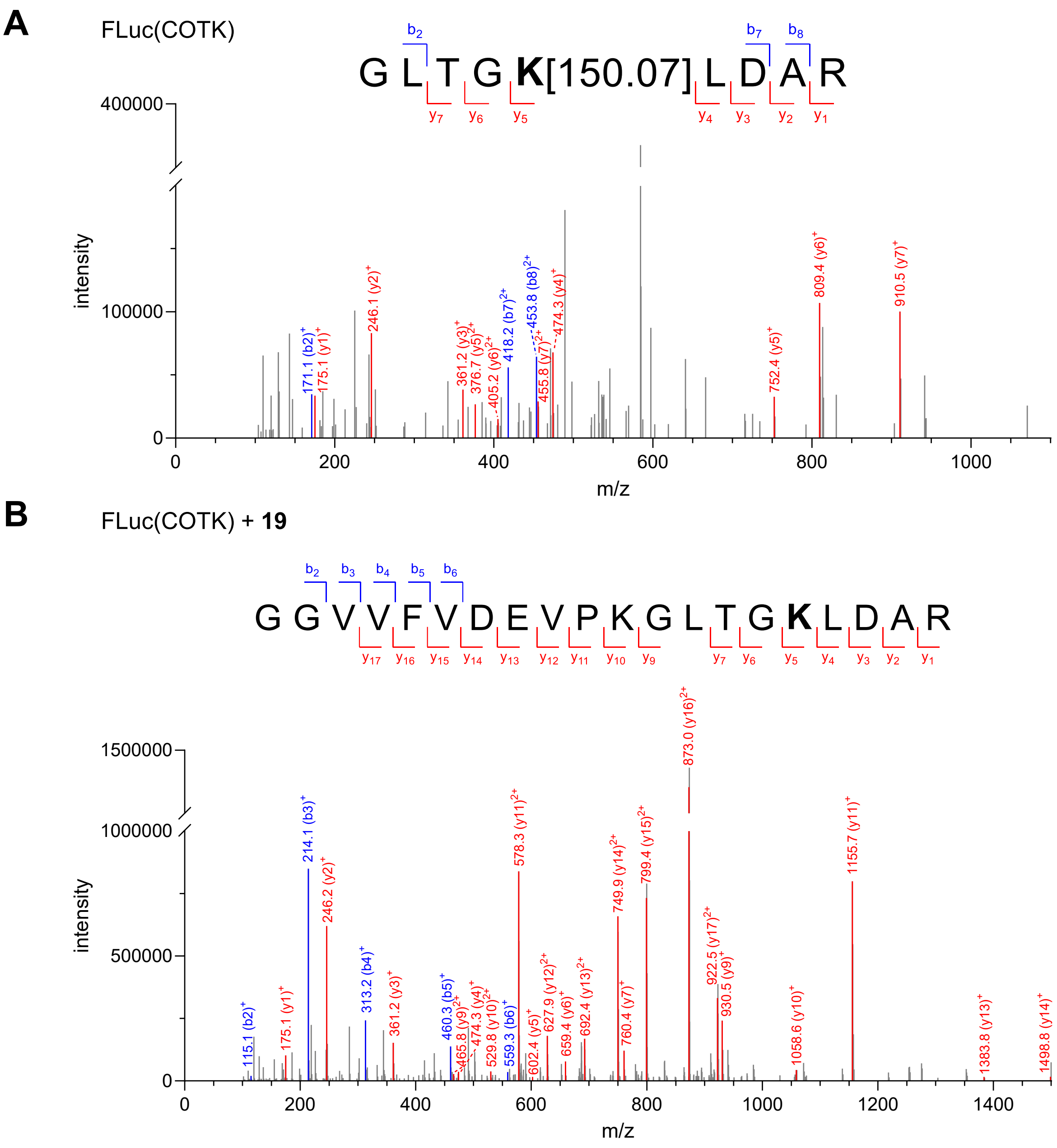

Figure S12. Representative annotated MS/MS spectra for firefly luciferase (FLuc) expressed and uncaged in HEK293T cells. (A) MS/MS spectrum showing FLuc(K529) caged with cyclooctynyl lysine (COTK) which represents the majority of peptides containing this residue. (B) MS/MS spectrum for caged FLuc treated with 100 µM **19** for 3 h. The majority of peptides containing K529 exhibits the native lysine.

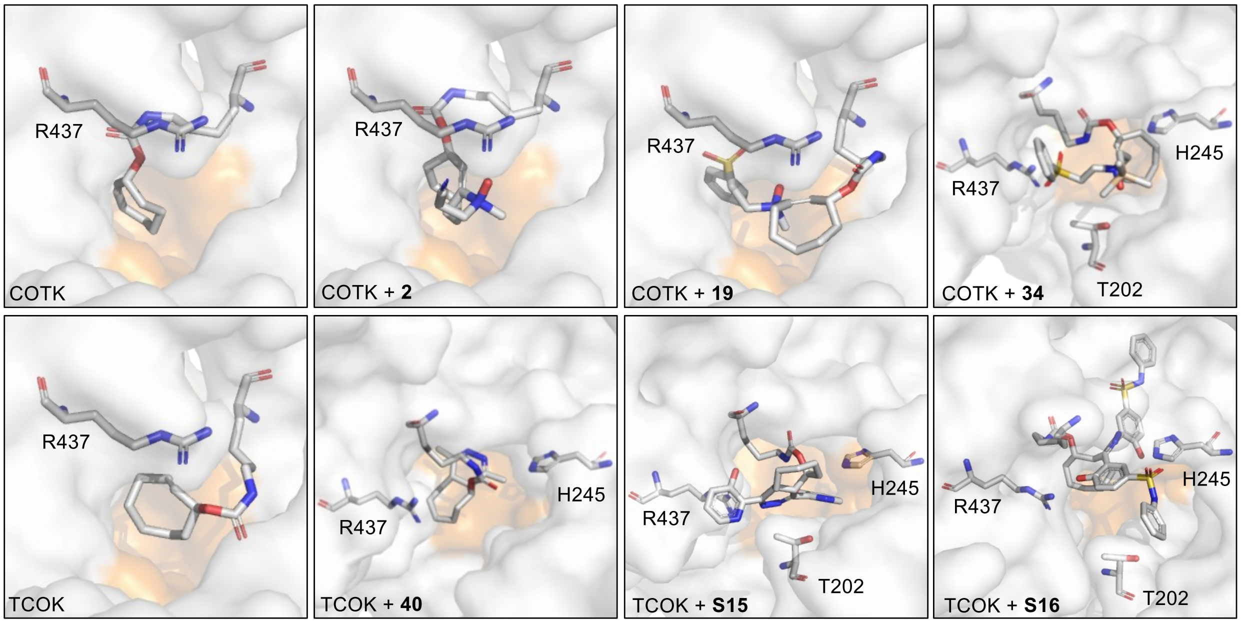

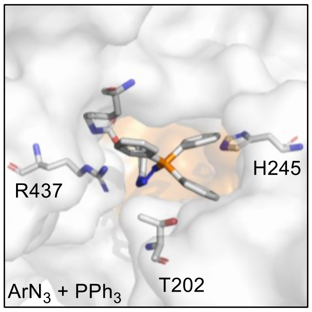

Adduct	VDW volume (Å³)	Energy (kcal/mol)		
		R437	T202	H245
COTK	139.25	–1.55	N/A	N/A
COTK + **2**	234.75	0.48	N/A	N/A
COTK + **19**	325.75	–0.35	N/A	N/A
COTK + **34**	427.88	0.30	8.17	1.39
TCOK	144.00	2.20	N/A	N/A
TCOK + **40**	221.00	0.71	N/A	–0.67
TCOK + **S15**	345.75	1.24	–1.01	16.25
TCOK + **S16**	596.63	106.37	1.77	–0.62
ArN_3 + PPh_3	408.63	23.68	19.68	4.21

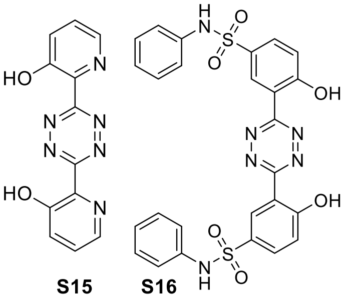

S15 (ref. 2) **S16** (ref. 3)

Figure S13. Molecular docking of caged lysine adducts with various small molecule triggers and their interaction energies with selected residues. In all panels, an alternative view from an angle different from that depicted in Figure 9 is presented. Additional entries TCOK + **S15**[2] and TCOK + **S16**[3] have been included.

Synthetic Procedures and Characterization

Cyclooct-2-yn-1-yl (4-nitrophenyl)carbamate (1):

The compound was synthesized according to literature procedure.[4]

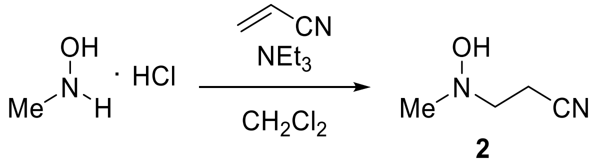

2

3-(Hydroxy(methyl)amino)propanenitrile (2):

Acrylonitrile (392 µL, 5.99 mmol, 1 equiv) was added to a solution of *N*-methylhydroxylamine hydrochloride (750 mg, 8.98 mmol, 1.50 equiv) and triethylamine (2.50 mL, 18.0 mmol, 3.00 equiv) in dichloromethane (30 mL). The resulting mixture was stirred for 1 h at room temperature. The mixture was diluted with hexanes and purified by flash column chromatography on silica gel (eluent: 30% acetone in hexanes) to yield product **2** (534 mg, 89%) as a white solid.

^{1}H NMR (500 MHz, CD_3OD) δ 2.81 (t, J = 6.5 Hz, 2H), 2.66 (t, J = 6.5 Hz, 2H), 2.62 (s, 3H).

^{13}C NMR (126 MHz, CD_3OD) δ 120.4, 58.2, 49.1, 16.7.

FTIR (thin film) cm^{-1}: 3403 (br), 2963 (w), 2855 (m), 2799 (w), 2251 (w), 1670 (w), 1442 (s), 1372 (m), 1330 (w), 1196 (w), 1136 (w), 1103 (m), 1010 (m), 880 (m), 820 (m), 768 (s).

HRMS (ESI) (*m*/*z*): calc'd for $C_4H_8N_2O$ $[M+H]^+$: 101.0715, found: 101.0713.

TLC (30% acetone in hexanes), R*f*: 0.31 ($KMnO_4$).

2-(((Cyclooct-2-yn-1-yloxy)carbonyl)amino)ethane-1-sulfonate ammonium salt (12):

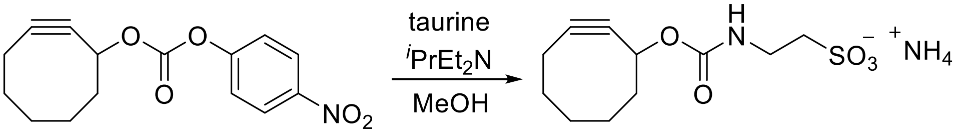

S1 12

N,N-Diisopropylethylamine (DIPEA, 380 µL, 2.77 mmol, 3.00 equiv) was added to a solution of **S1** (267 mg, 0.923 mmol, 1 equiv)[4-5] and taurine (138 mg, 1.11 mmol, 1.20 equiv) in methanol (9.2 mL) at 0 °C. The solution was stirred for 1.5 h, then concentrated under reduced pressure, diluted with water, and purified by automated C_{18} reverse phase column chromatography (30 g C_{18} silica gel, 25 µm spherical particles, eluent: H_2O+0.2% NH_4OH (5 CV), gradient 0→100% $MeCN/H_2O$+0.2% NH_4OH (15 CV)) Fractions containing the desired product were collected and concentrated *in vacuo* to provide ammonium sulfate salt **12** (192 mg, 66%) as a colorless solid.

^{1}H NMR (500 MHz, CD_3OD, 25 °C) δ 6.84 (t, J = 6.0 Hz, N-H), 5.16 (t, J = 5.9 Hz, 1H), 3.50 (t, J = 7.0 Hz, 2H), 2.96 (t, J = 7.0 Hz, 2H), 2.25 (dt, J = 13.5, 6.4 Hz, 1H), 2.21–2.05 (m, 2H), 1.98 (ddd, J = 14.3, 8.9, 6.5 Hz, 1H), 1.89 (qq, J = 7.0, 4.4, 3.5 Hz, 2H), 1.85–1.74 (m, 1H), 1.75–1.58 (m, 2H), 1.60–1.47 (m, 1H).

^{13}C NMR (126 MHz, DMSO-*d*$_6$, 25 °C) δ 155.1, 101.1, 91.9, 65.9, 50.7, 41.7, 37.2, 33.9, 29.3, 25.9, 20.1.

FTIR (thin film) cm^{-1}: 3198 (br), 3068 (br), 2926 (w), 2851 (w), 2218 (w), 1700 (m), 1521 (w), 1439 (w), 1256 (m), 1159 (s), 1036 (s), 984 (m), 775 (w), 749 (w).

HRMS (ESI) (*m*/*z*): calc'd for $C_{11}H_{17}NO_5S$ $[M+H]^+$: 276.0906, found: 276.0900.

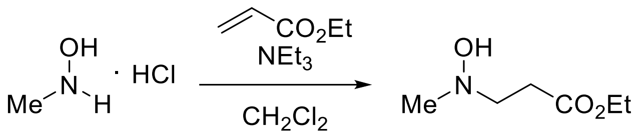

14

Ethyl 3-(hydroxy(methyl)amino)propanoate (14):

Ethyl acrylate (210 μL, 2.00 mmol, 1 equiv) was added to a solution of *N*-methylhydroxylamine hydrochloride (250 mg, 3.00 mmol, 1.50 equiv) and triethylamine (836 μL, 6.00 mmol, 3.00 equiv) in dichloromethane (4 mL). The reaction mixture was stirred at room temperature for 2 h. The mixture was diluted with hexanes and purified by flash column chromatography on silica gel (eluent: 50% ethyl acetate in hexanes) to provide hydroxylamine **14** (272 mg, 92%) as a colorless oil.

^{1}H NMR (500 MHz, CD_3OD) δ 4.13 (q, *J* = 7.1 Hz, 2H), 2.87 (d, *J* = 7.1 Hz, 2H), 2.59 (s, 3H), 2.56 (t, *J* = 6.9 Hz, 2H), 1.25 (t, *J* = 7.1 Hz, 3H).

^{13}C NMR (126 MHz, CD_3OD) δ 174.3, 61.7, 58.5, 49.2, 33.7, 14.6.

FTIR (thin film) cm^{-1}: 3451 (br), 3209 (br), 2982 (w), 2855 (w), 1737 (s), 1446 (w), 1379 (w), 1319 (w), 1256 (w), 1182 (s), 1096 (w), 1032 (w).

HRMS (ESI) (*m*/*z*): calc'd for $C_6H_{13}NO_3$ $[M+H]^+$: 148.0974, found: 148.0968.

TLC (50% ethyl acetate in hexane), R*f*: 0.28 ($KMnO_4$).

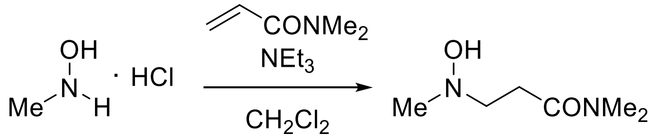

15

3-(Hydroxy(methyl)amino)-*N*,*N*-dimethylpropanamide (15):

N,*N*-Dimethyl acrylamide (210 μL, 2.00 mmol, 1 equiv) was added to a solution of *N*-methylhydroxylamine hydrochloride (250 mg, 3.00 mmol, 1.50 equiv) and triethylamine (836 μL,

6.00 mmol, 3.00 equiv) in dichloromethane (4 mL). The reaction mixture was stirred at room temperature for 6 h. The resulting mixture was diluted with dichloromethane and purified by flash column chromatography on silica gel (eluent: 2→5% methanol in dichloromethane) to provide hydroxylamine **15** (135 mg, 46 %) as a colorless oil.

^{1}H NMR (500 MHz, CD_3OD) δ 3.09 (s, 3H), 2.93 (s, 3H), 2.92–2.82 (m, 2H), 2.65 (t, J = 7.2 Hz, 2H), 2.61 (s, 3H).

^{13}C NMR (126 MHz, CD_3OD) δ 174.3, 59.0, 49.3, 38.0, 35.9, 32.3.

FTIR (thin film) cm^{-1}: 3284 (br), 2922 (w), 2851 (w), 1617 (s), 1498 (m), 1401 (s), 1263 (w), 1155 (m), 1088 (w), 980 (w).

HRMS (ESI) (*m/z*): calc'd for $C_6H_{14}N_2O_2$ [M+H]$^+$: 147.1134, found: 147.1128.

TLC (10% methanol in dichloromethane), R*f*: 0.33 ($KMnO_4$).

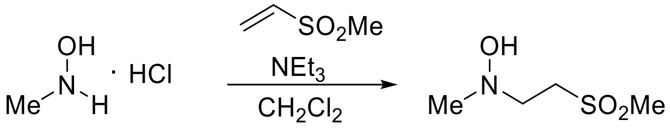

16

N-methyl-N-(2-(methylsulfonyl)ethyl)hydroxylamine (16):

Methyl vinyl sulfone (525 μL, 5.99 mmol, 1 equiv) was added to a solution of *N*-methylhydroxylamine hydrochloride (750 mg, 8.98 mmol, 1.50 equiv) and triethylamine (2.50 mL, 18.0 mmol, 3.00 equiv) in dichloromethane (30 mL). The reaction mixture was stirred at room temperature for 1 h. The mixture was diluted with hexanes and purified by flash column chromatography on silica gel (eluent: 100% ethyl acetate) to provide hydroxylamine **16** (793 mg, 86%) as a white solid.

^{1}H NMR (500 MHz, DMSO-d_6) δ 8.10 (s, 1H), 3.31 (t, J = 6.8 Hz, 2H), 3.06 (s, 3H), 2.94 (t, J = 6.9 Hz, 2H), 2.57 (s, 3H).

^{13}C NMR (126 MHz, DMSO-d_6) δ 54.9, 51.9, 48.5, 41.5.

FTIR (thin film) cm^{-1}: 3444 (br), 3011 (w), 2963 (w), 2930 (w), 2855 (w), 1446 (w), 1413 (w), 1372 (w), 1286 (s), 1185 (w), 1129 (s), 962 (m), 872 (w).

HRMS (ESI) (*m/z*): calc'd for $C_4H_{11}NO_3S$ [M+H]$^+$: 154.0538, found: 154.0532.

TLC (100% ethyl acetate), R*f*: 0.50 ($KMnO_4$).

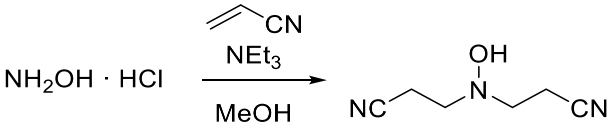

17

3,3'-(Hydroxyazanediyl)dipropanenitrile (17):
Acrylonitrile (1.77 mL, 27.0 mmol, 2.50 equiv) was added to a solution of hydroxylamine hydrochloride (750 mg, 10.8 mmol, 1 equiv) and triethylamine (3.76 mL, 27.0 mmol, 2.50 equiv) in methanol (100 mL). The reaction mixture was stirred at room temperature for 1 h. The resulting mixture was concentrated under reduced pressure, then the resulting crude residue was purified by flash column chromatography on silica gel (eluent: 100% diethyl ether) to provide **17** (1.25 g, 83%) as a yellow oil.

^{1}H NMR (500 MHz, CD_3OD) δ 2.91 (t, *J* = 6.5 Hz, 4H), 2.69 (t, *J* = 6.5 Hz, 4H).

^{13}C NMR (126 MHz, CD_3OD) δ 120.3, 56.9, 16.8.

FTIR (thin film) cm^{-1}: 3407 (br), 2945 (w), 2903 (w), 2855 (w), 2251 (s), 1446 (m), 1420 (s), 1360 (s), 1271 (w), 1126 (w), 1051 (w), 1006 (w), 883 (m), 835 (w), 790 (w), 753 (w).

HRMS (ESI) (*m/z*): calc'd for $C_6H_9N_3O$ $[M+H]^+$: 140.0824, found: 140.0819.

TLC (100% diethyl ether), R*f*: 0.43 ($KMnO_4$).

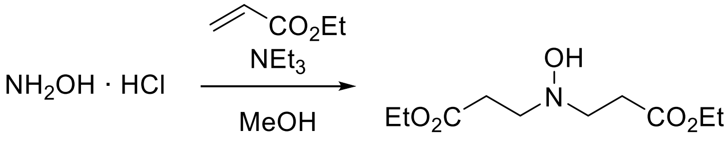

18

Diethyl 3,3'-(hydroxyazanediyl)dipropionate (18):
Ethyl acrylate (2.88 mL, 27.0 mmol, 2.50 equiv) was added to a solution of hydroxylamine hydrochloride (750 mg, 10.8 mmol, 1 equiv) and triethylamine (3.76 mL, 27.0 mmol, 2.50 equiv) in methanol (100 mL). The reaction mixture was stirred at room temperature for 1 h. The mixture was concentrated under reduced pressure, then the resulting crude residue was purified by flash column chromatography on silica gel (eluent: 60% diethyl ether in hexanes) to provide **18** (2.43 g, 96 %) as a colorless liquid.

^{1}H NMR (500 MHz, CD_3OD) δ 4.12 (q, *J* = 7.1 Hz, 4H), 2.92 (t, *J* = 6.9 Hz, 4H), 2.57 (t, *J* = 6.9 Hz, 4H), 1.25 (t, *J* = 7.1 Hz, 6H).

^{13}C NMR (126 MHz, CD_3OD) δ 174.4, 61.6, 57.2, 33.8, 14.6.

FTIR (thin film) cm^{-1}: 3448 (br), 2982 (w), 2907 (w), 1729 (s), 1446 (w), 1370 (m), 1260 (m), 1182 (s), 1096 (w), 1029 (m), 861 (w).

HRMS (ESI) (*m/z*): calc'd for $C_{10}H_{19}NO_5$ $[M+H]^+$: 234.1341, found: 234.1336.

TLC (60% diethyl ether in hexanes), R*f*: 0.36 ($KMnO_4$).

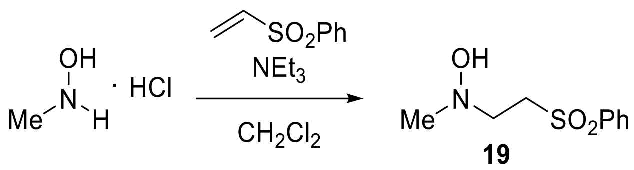

<u>*N*-Methyl-*N*-(2-(phenylsulfonyl)ethyl)hydroxylamine (19):</u>

Phenyl vinyl sulfone (336 mg, 2.00 mmol, 1 equiv) was added to a solution of *N*-methylhydroxylamine hydrochloride (250 mg, 3.00 mmol, 1.50 equiv) and triethylamine (836 µL, 6.00 mmol, 3.00 equiv) in dichloromethane (4 mL). The reaction mixture was stirred at room temperature for 3 h. The resulting mixture was diluted with hexanes and purified by flash column chromatography on silica gel (eluent: 30% acetone in hexanes) to provide hydroxylamine **19** (397 mg, 92%) as a white solid.

^{1}H NMR (500 MHz, CD_3OD) δ 8.00–7.89 (m, 2H), 7.78–7.70 (m, 1H), 7.65 (dd, J = 8.5, 7.1 Hz, 2H), 3.54–3.40 (m, 2H), 2.89 (dd, J = 9.1, 5.5 Hz, 2H), 2.53 (s, 3H).

^{13}C NMR (126 MHz, CD_3OD) δ 140.9, 135.3, 130.7, 129.2, 56.2, 55.0, 49.0.

FTIR (thin film) cm^{-1}: 3459 (br), 2989 (w), 2959 (w), 2855 (w), 1446 (m), 1409 (w), 1371 (w), 1305 (m), 1245 (w), 1185 (w), 1144 (s), 1085 (m).

HRMS (ESI) (*m*/*z*): calc'd for $C_9H_{13}NO_3S$ [M+H]$^+$: 216.0694, found: 216.0690.

TLC (30% acetone in hexanes), R*f*: 0.26 (UV, $KMnO_4$).

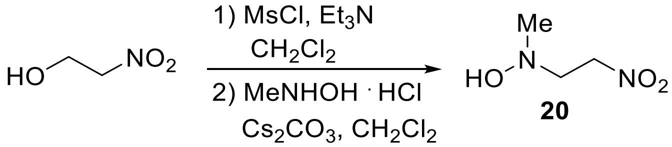

<u>*N*-Methyl-*N*-(2-nitroethyl)hydroxylamine (20):</u>

2-Nitroethan-1-ol (100 mg, 1.10 mmol, 1.10 equiv) was added to dichloromethane (2 mL), and the solution was cooled to 0 °C. Triethylamine (0.290 ml, 2.2 mmol, 2 equiv) and methanesulfonyl chloride (132 mg, 1.15 mmol, 1.05 equiv) were then sequentially added to the solution dropwise. After the mixture was stirred at 0 °C for 2 h, *N*-methylhydroxylamine hydrochloride (137 mg, 1.65 mmol, 1.50 equiv) and cesium carbonate (539 mg, 1.65 mmol, 1.50 equiv) were sequentially added. The solution was warmed to room temperature and stirred for 3 h. The mixture was washed with brine (3 × 5 mL), dried over anhydrous sodium sulfate, filtered, and concentrated. The concentrated residue was diluted with pentane and purified by flash column chromatography on silica gel (eluent: 30% diethyl ether in pentane) to provide hydroxylamine **20** (80 mg, 60%) as a white solid.

^{1}H NMR (500 MHz, $CDCl_3$) δ 4.61 (t, J = 5.7, 2H), 3.22 (t, J = 5.8, 2H), 2.70 (s, 3H).

^{13}C NMR (126 MHz, $CDCl_3$) δ 73.5, 58.1, 49.0.

FTIR (thin film) cm^{-1}: 3138 (br), 2845 (br), 1541 (s), 1431 (s), 1362 (s), 1230 (m), 1192 (m), 1086 (m), 908 (m), 878 (m), 787 (m), 690 (w), 617 (m), 478 (m), 435 (s),

HRMS (ESI) (*m*/*z*): calc'd for $C_3H_9N_2O_3$ $[M+H]^+$: 121.0608, found: 121.0608.

TLC (30% acetone in hexanes), R*f*: 0.26 ($KMnO_4$).

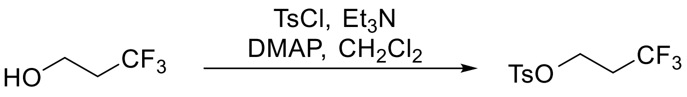

S2

3,3,3-Trifluoropropyl-4-methylbenzenesulfonate (S2):

4-Toluenesulfonyl chloride (184 mg, 0.964 mmol, 1.10 equiv) was added to a solution of 3,3,3-trifluoropropan-1-ol (100 mg, 0.877 mmol, 1 equiv), 4-(dimethylamino)pyridine (21.4 mg, 0.175 mmol, 0.20 equiv) and triethylamine (146 µL, 1.05 mmol, 1.20 equiv) in dichloromethane (2 mL). The solution was stirred at room temperature for 1 h. The resulting mixture was diluted with hexanes and purified by flash column chromatography on silica gel (eluent: 12.5% ethyl acetate in hexanes) to provide tosylate **S2** (200 mg, 85%) as a pale-yellow oil.

^{1}H NMR (500 MHz, $CDCl_3$) δ 7.80 (d, *J* = 8.4, 2H), 7.37 (d, *J* = 8.3, 2H), 4.22 (t, *J* = 6.5, 2H), 2.52 (dtd, *J* = 10.2, 6.5, 3.2) 2.46 (s, 3H).

^{13}C NMR (126 MHz, $CDCl_3$) δ 145.5, 132.5, 128.1, 123.8, 62.5, 33.8, 21.8.

FTIR (thin film) cm^{-1}: 2360 (w), 1599 (w), 1363 (m), 1251 (s), 1176 (s), 1157(s), 1132 (s), 1062(m), 989 (s), 904 (s), 813 (m), 761 (m), 664 (s), 554 (s), 451 (w),

HRMS (ESI) (*m*/*z*): calc'd for $C_{10}H_{15}F_3NO_3S$ $[M+NH_4]^+$: 286.0719, found: 286.0720.

TLC (12.5% ethyl acetate in hexanes), R*f*: 0.51 (UV, $KMnO_4$).

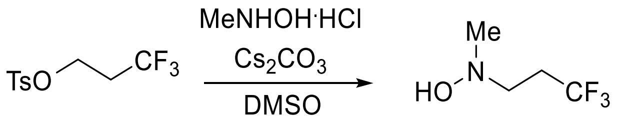

S2 **21**

3,3,3-Trifluoropropyl-4-Methylbenzenesulfonate (21):

Cesium carbonate (485 mg, 1.49 mmol, 2.0 equiv) was added to a solution of tosylate **S2** (200 mg, 0.745 mmol, 1 equiv) and *N*-methylhydroxylamine hydrochloride (93.5 mg, 1.12 mmol, 1.50 equiv) in dimethylsulfoxide (3 mL). The reaction mixture was stirred at 60 °C for 3 h. The solution was diluted with saturated aqueous ammonium chloride solution (5 mL) then extracted with diethyl ether (3 × 5 mL). The combined organic layers were washed with brine (3 × 5 mL), dried over anhydrous sodium sulfate, filtered, and concentrated. The concentrated residue was diluted with

hexanes and purified by flash column chromatography on silica gel (eluent: 30% diethyl ether in pentane) to provide hydroxylamine **21** (42 mg, 39%) as a pale-yellow oil.[1]

HRMS (ESI) (*m*/*z*): calc'd for $C_4H_9F_3NO$ $[M+H]^+$: 144.0631, found: 144.0632.

^{1}H NMR (500 MHz, $CDCl_3$) δ 2.88–2.84(m, 2H), 2.69 (s, 3H), 2.51–2.41 (m, 2H).

^{13}C NMR (126 MHz, $CDCl_3$) δ 126.6 (q, J = 275 Hz), 54.6, 49.0, 32.1 (q, J = 28.4 Hz).

FTIR (thin film) cm^{-1}: 3120 (br), 2825 (br), 1462 (w), 1440 (w), 1417 (w), 1394 (w), 1340 (m), 1247 (s), 1149 (s), 1122 (s), 1072 (m), 1031 (s), 995 (s), 972 (m), 819 (m), 655 (s).

TLC (30% diethyl ether in pentane), R*f*: 0.23 ($KMnO_4$).

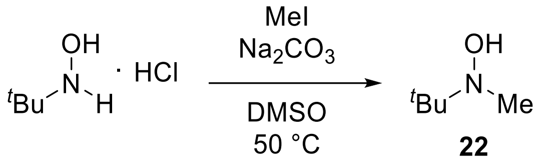

22

***N*-(Tert-butyl)-*N*-methylhydroxylamine (22):**

Iodomethane (150 µL, 2.40 mmol, 1.20 equiv) was added to a suspension of *N*-(*tert*-butyl)hydroxylamine hydrochloride (251 mg, 2.00 mmol, 1 equiv) and sodium carbonate (635.9 mg, 6.00 mmol, 3.00 equiv) in dimethyl sulfoxide (2 mL). The reaction mixture was stirred at 50 °C for 3 h. The mixture was diluted with diethyl ether (20 mL) and washed with saturated aqueous sodium bicarbonate (20 mL), extracted with diethyl ether (2 × 20 mL). The combined organic layers were washed with brine (2 × 50 mL), dried over magnesium sulfate, filtered, and concentrated under reduced pressure. The resulting crude residue was purified by flash column chromatography on silica gel (eluent: 50% diethyl ether in pentanes) to provide **22** (30.0 mg, 14%) as a volatile colorless oil.

^{1}H NMR (500 MHz, CD_3OD) δ 2.53 (s, 3H), 1.09 (s, 9H).

^{13}C NMR (126 MHz, CD_3OD) δ 40.6, 26.3, 25.0.

FTIR (thin film) cm^{-1}: 3142 (br), 2971 (s), 2911 (m), 1718 (m), 1685 (m), 1453 (m), 1387 (m), 1360 (s), 1215 (s), 1156 (s), 1081 (w), 1006 (w), 898 (w), 820 (w), 704 (m).

HRMS (ESI) (*m*/*z*): calc'd for $C_5H_{13}NO$ $[M+H]^+$: 104.1075, found: 104.1074.

TLC (50% diethyl ether in pentanes), R*f*: 0.40 ($KMnO_4$).

---

[1] Diethyl ether, which was used in flash column chromatography, along with the associated ethanol impurity, was not completely removed because of the volatility of the hydroxylamine product. Ethanol: ^{13}C NMR (126 MHz, $CDCl_3$) δ 18.5, 58.5. Diethyl ether: ^{13}C NMR (126 MHz, $CDCl_3$) δ 15.4, 66.0.

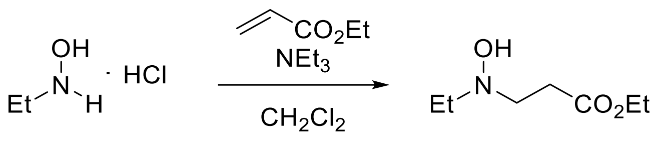

23

Ethyl 3-(ethyl(hydroxy)amino)propanoate (23):

Ethyl acrylate (106 µL, 1.00 mmol, 1 equiv) was added to a solution of *N*-ethylhydroxylamine hydrochloride (146 mg, 1.50 mmol, 1.50 equiv) and triethylamine (418 µL, 3.00 mmol, 3.00 equiv) in dichloromethane (2 mL). The reaction mixture was stirred at room temperature for 2 h. The mixture was diluted with hexanes and purified by flash column chromatography on silica gel (eluent: 33% ethyl acetate in hexanes) to provide **23** (141 mg, 88 %) as a colorless oil.

^{s1}H NMR (500 MHz, CD_3OD) δ 4.13 (q, *J* = 7.1 Hz, 2H), 2.96–2.84 (m, 2H), 2.68 (q, *J* = 7.1 Hz, 2H), 2.58 (t, *J* = 7.0 Hz, 2H), 1.25 (t, *J* = 7.1 Hz, 3H), 1.12 (t, *J* = 7.1 Hz, 3H).

^{13}C NMR (126 MHz, CD_3OD) δ 174.5, 61.7, 56.8, 56.0, 33.7, 14.6, 12.7.

FTIR (thin film) cm^{-1}: 3440 (br), 2978 (w), 2937 (w), 2904 (w), 2848 (w), 1733 (s), 1446 (w), 1372 (m), 1252 (m), 1182 (s), 1096 (m), 1029 (m), 909 (w), 861 (w), 768 (w).

HRMS (ESI) (*m*/*z*): calc'd for $C_7H_{15}NO_3$ $[M+H]^+$: 162.1130, found: 162.1125.

TLC (50% ethyl acetate in hexane), R*f*: 0.60 ($KMnO_4$).

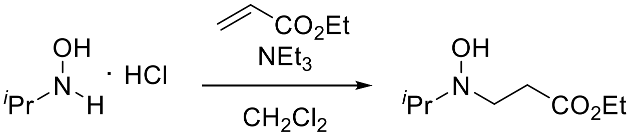

24

Ethyl 3-(hydroxy(isopropyl)amino)propanoate (24):

Ethyl acrylate (213 µL, 2.00 mmol, 1 equiv) was added to a solution of *N*-isopropylhydroxylamine hydrochloride (352 mg, 3.00 mmol, 1.50 equiv) and triethylamine (836 µL, 6.00 mmol, 3.00 equiv) in dichloromethane (4 mL). The reaction mixture was stirred at room temperature for 1.5 h. The mixture was diluted with hexanes and purified by flash column chromatography on silica gel (eluent: 33% ethyl acetate in hexanes) to provide **24** (333 mg, 95%) as a colorless oil.

^{1}H NMR (500 MHz, CD_3OD) δ 4.13 (q, *J* = 7.1 Hz, 2H), 2.93 (t, *J* = 7.0 Hz, 2H), 2.82 (hept, *J* = 6.4 Hz, 1H), 2.57 (t, *J* = 6.9 Hz, 2H), 1.25 (t, *J* = 7.1 Hz, 3H), 1.08 (d, *J* = 6.4 Hz, 6H).

^{13}C NMR (126 MHz, CD_3OD) δ 174.7, 61.6, 59.2, 53.0, 34.1, 19.1, 14.6.
FTIR (thin film) cm^{-1}: 3444(br), 2974 (m), 2937 (w), 1733 (s), 1446 (w), 1372 (m), 1316 (m), 1271 (m), 1182 (s), 1096 (w), 1032 (m), 962 (w), 909 (w), 865 (w), 790 (w).

HRMS (ESI) (*m*/*z*): calc'd for $C_8H_{17}NO_3$ $[M+H]^+$: 176.1287, found: 176.1282.

TLC (50% ethyl acetate in hexane), R*f*: 0.43 (UV, $KMnO_4$).

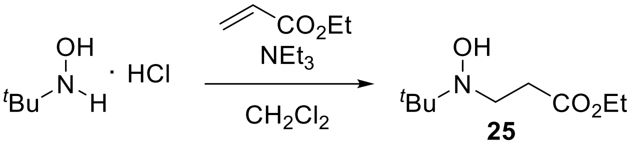

25

Ethyl 3-(tert-butyl(hydroxy)amino)propanoate (25):

Ethyl acrylate (213 µL, 2.00 mmol, 1 equiv) was added to a solution of *N*-(*tert*-butyl)hydroxylamine hydrochloride (384 mg, 3.00 mmol, 1.50 equiv) and triethylamine (836 µL, 6.00 mmol, 3.00 equiv) in dichloromethane (4 mL). The reaction mixture was stirred at room temperature for 3.5 h. The mixture was diluted with hexanes and purified by flash column chromatography on silica gel (eluent: 25% ethyl acetate in hexanes) to provide **25** (326 mg, 86%) as a colorless oil.

^{1}H NMR (500 MHz, CD_3OD) δ 4.12 (q, J = 7.1 Hz, 2H), 2.91 (t, J = 7.0 Hz, 2H), 2.55 (t, J = 6.8 Hz, 2H), 1.25 (t, J = 7.1 Hz, 3H), 1.09 (s, 9H).

^{13}C NMR (126 MHz, CD_3OD) δ 174.9, 61.6, 59.8, 48.9, 34.9, 25.7, 14.7.

FTIR (thin film) cm^{-1}: 3429 (br), 2974 (m), 2911 (w), 1718 (s), 1446 (w), 1360 (m), 1274 (m), 1218 (m), 1178 (s), 1096 (w), 1066 (m), 1029 (m), 909 (w).

HRMS (ESI) (*m*/*z*): calc'd for $C_9H_{19}NO_3$ $[M+H]^+$: 190.1443, found: 190.1438.

TLC (33% ethyl acetate in hexanes), R*f*: 0.30 ($KMnO_4$).

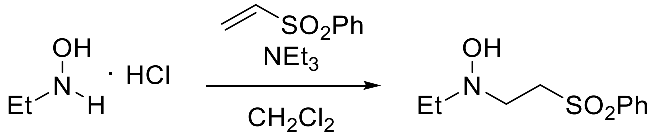

26

***N*-Ethyl-*N*-(2-(phenylsulfonyl)ethyl)hydroxylamine (26):**

Phenyl vinyl sulfone (168 mg, 1.00 mmol, 1 equiv) was added to a solution of *N*-ethylhydroxylamine hydrochloride (146 mg, 1.50 mmol, 1.50 equiv) and triethylamine (418 µL, 3.00 mmol, 3.00 equiv) in dichloromethane (2 mL). The reaction mixture was stirred at room temperature for 2 h. The mixture was diluted with hexanes and purified by flash column chromatography on silica gel (eluent: 50% ethyl acetate in hexanes) to provide **26** (221 mg, 96%) as a white solid.

^{1}H NMR (500 MHz, CD_3OD) δ 7.98–7.88 (m, 2H), 7.77–7.70 (m, 1H), 7.65 (dd, J = 8.4, 7.1 Hz, 2H), 3.54–3.41 (m, 2H), 2.91 (t, J = 7.3 Hz, 2H), 2.61 (q, J = 7.1 Hz, 2H), 1.05 (t, J = 7.1 Hz, 3H).

^{13}C NMR (126 MHz, CD_3OD) δ 140.9, 135.2, 130.7, 129.2, 55.9, 55.1, 54.6, 12.7.

FTIR (thin film) cm^{-1}: 3463 (br), 2974 (w), 2848 (w), 2937 (w), 1446 (w), 1301 (m), 1141 (s), 1085 (m), 1021 (w), 924 (w), 831 (w), 745 (m), 690 (m).

HRMS (ESI) (*m/z*): calc'd for $C_{10}H_{15}NO_3S$ $[M+H]^+$: 230.0851, found: 230.0846.

TLC (50% ethyl acetate in hexane), R*f*: 0.27 (UV, $KMnO_4$)

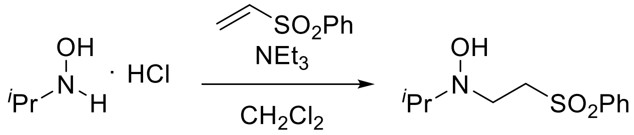

27

<u>*N*-Isopropyl-*N*-(2-(phenylsulfonyl)ethyl)hydroxylamine (27):</u>

Phenyl vinyl sulfone (168 mg, 1.00 mmol, 1 equiv) was added to a solution of *N*-isopropylhydroxylamine hydrochloride (167 mg, 1.50 mmol, 1.50 equiv) and triethylamine (418 µL, 3.00 mmol, 3.00 equiv) in dichloromethane (2 mL). The reaction mixture was stirred at room temperature for 3.5 h. The mixture was diluted with hexanes and purified by flash column chromatography on silica gel (eluent: 50% ethyl acetate in hexanes) to provide **27** (230 mg, 95%) as a colorless oil.

^{1}H NMR (500 MHz, CD_3OD) δ 7.98–7.88 (m, 2H), 7.78–7.70 (m, 1H), 7.69–7.58 (m, 2H), 3.50–3.41 (m, 2H), 3.00–2.88 (m, 2H), 2.75 (hept, J = 6.4 Hz, 1H), 1.00 (d, J = 6.4 Hz, 6H).

^{13}C NMR (126 MHz, CD_3OD) δ 141.0, 135.2, 130.7, 129.2, 59.0, 55.4, 51.1, 19.0.

FTIR (thin film) cm^{-1}: 3470 (br), 2974 (w), 2933 (w), 1446 (w), 1305 (m), 1141 (s), 1085 (m), 824 (w), 768 (w), 742 (m), 690 (m).

HRMS (ESI) (*m/z*): calc'd for $C_{11}H_{17}NO_3S$ $[M+H]^+$: 244.1007, found: 244.1002.

TLC (50% ethyl acetate in hexanes), R*f*: 0.43 (UV, $KMnO_4$).

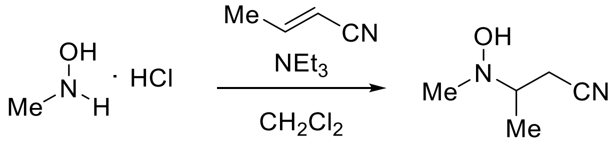

28

<u>3-(Hydroxy(methyl)amino)butanenitrile (28):</u>

Crotonitrile (172 µL, 2.00 mmol, 1 equiv) was added to a solution of *N*-methyl hydroxylamine hydrochloride (250 mg, 3.00 mmol, 1.50 equiv) and triethylamine (836 µL, 6.00 mmol, 3.00 equiv) in dichloromethane (4 mL). The reaction mixture was stirred at room temperature for 6 h. The resulting mixture was diluted with hexanes and purified by flash column chromatography on silica gel (eluent: 60% ethyl acetate in hexanes) to provide **28** (173 mg, 76%) as a colorless oil.

^{1}H NMR (500 MHz, CD_3OD) δ 2.91 (h, J = 6.4 Hz, 1H), 2.70 (dd, J = 16.8, 5.2 Hz, 1H), 2.63 (dd, J = 16.8, 6.4 Hz, 1H), 2.60 (s, 3H), 1.19 (d, J = 6.5 Hz, 3H).

^{13}C NMR (126 MHz, CD_3OD) δ 120.1, 61.2, 45.5, 22.3, 15.8.

FTIR (thin film) cm^{-1}: 3429 (br), 2978 (m), 2863 (m), 2251 (w), 1442 (m), 1379 (m), 1349 (m), 1290 (w), 1230 (w), 1196 (w), 1148 (w), 1066 (w), 1032 (m), 920 (w), 850 (w), 768 (w).

HRMS (ESI) (*m*/*z*): calc'd for $C_5H_{10}N_2O$ $[M+H]^+$: 115.0871, found: 115.0869.

TLC (66% ethyl acetate in hexane), R*f*: 0.52 ($KMnO_4$).

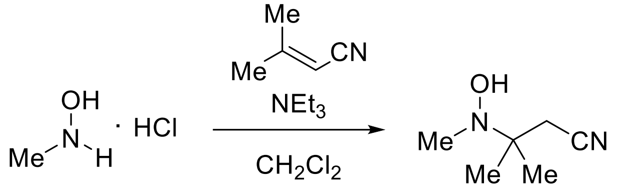

29

3-(Hydroxy(methyl)amino)-3-methylbutanenitrile (29):

3-Methylbut-2-enenitrile (299 mg, 3.00 mmol, 1 equiv) was added to a solution of *N*-methyl hydroxylamine hydrochloride (243 mg, 3.00 mmol, 1.00 equiv) and triethylamine (840 µL, 6.00 mmol, 2.00 equiv) in dichloromethane (2 mL). The reaction mixture was stirred at room temperature for 24 h. The resulting mixture was diluted with hexanes and purified by flash column chromatography on silica gel (eluent: 50% ethyl acetate in hexanes) to provide **29** (73.7 mg, 19%) as a pale yellow oil.

^{1}H NMR (500 MHz, CD_3OD) δ 2.65 (s, 2H), 2.55 (s, 3H), 1.20 (s, 6H).

^{13}C NMR (126 MHz, CD_3OD) δ 120.2, 61.3, 40.9, 27.5, 22.5.

FTIR (thin film) cm^{-1}: 3433 (br), 3220 (br), 2978 (s), 2941 (m), 2889 (m), 2251 (w), 1469 (m), 1442 (m), 1387 (m), 1300 (w), 1274 (w), 1237 (w), 1141 (m), 1070 (w), 898 (w), 809 (w), 723 (w).

HRMS (ESI) (*m*/*z*): calc'd for $C_6H_{12}N_2O$ $[M+H]^+$: 129.1028, found: 129.1024.

TLC (50% ethyl acetate in hexanes), R*f*: 0.38 ($KMnO_4$).

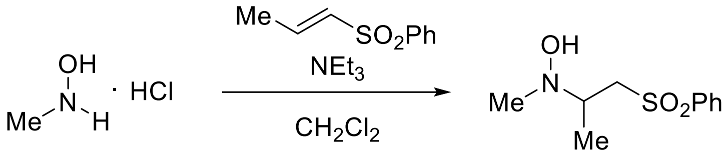

30

***N*-Methyl-*N*-(1-(phenylsulfonyl)propan-2-yl)hydroxylamine (30):**

(*E*)-1-(Phenylsulfonyl)-1-propene[6] (364 mg, 2.00 mmol, 1 equiv) was added to a solution of *N*-methyl hydroxylamine hydrochloride (250 mg, 3.00 mmol, 1.50 equiv) and triethylamine (836 µL, 6.00 mmol, 3.00 equiv) in dichloromethane (4 mL). The reaction mixture was stirred at room temperature for 4 h, then concentrated under reduced pressure. The resulting crude residue was purified by flash column chromatography on silica gel (eluent: 2% methanol in dichloromethane) to provide **30** (427 mg, 93%) as a white solid.

^{1}H NMR (500 MHz, CD_3OD) δ 7.99–7.88 (m, 2H), 7.77–7.69 (m, 1H), 7.68–7.59 (m, 2H), 3.70–3.58 (m, 1H), 3.21–3.06 (m, 2H), 2.46 (s, 3H), 1.21 (d, J = 6.4 Hz, 3H).

^{13}C NMR (126 MHz, CD_3OD) δ 141.4, 135.1, 130.6, 129.1, 59.2, 59.2, 44.2, 16.6.

FTIR (thin film) cm^{-1}: 3470 (br), 3163 (br), 3064 (w), 2982 (w), 2937 (w), 2885 (w), 1446 (m), 1401 (w), 1305 (s), 1256 (w), 1148 (s), 1085 (m), 1025 (w), 842 (w), 753 (m), 719 (w), 690 (m).

HRMS (ESI) (*m/z*): calc'd for $C_{10}H_{15}NO_3S$ $[M+H]^+$: 230.0851, found: 230.0846.

TLC (2.5% methanol in dichloromethane), R*f*: 0.38 (UV, $KMnO_4$).

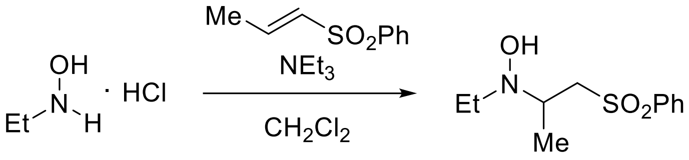

31

<u>*N*-Ethyl-*N*-(1-(phenylsulfonyl)propan-2-yl)hydroxylamine (31):</u>
(*E*)-1-(Phenylsulfonyl)-1-propene[6] (75.2 mg, 413 μmol, 1 equiv) was added to a solution of *N*-ethylhydroxylamine hydrochloride (60.5 mg, 620 μmol, 1.50 equiv) and triethylamine (173 μL, 1.24 mmol, 3.00 equiv) in dichloromethane (1.24 mL). The reaction mixture was stirred at room temperature for 3 h. The resulting mixture was diluted with hexanes and purified by flash column chromatography on silica gel (eluent: 66% ethyl acetate in hexanes) to provide **31** (95.7 mg, 95%) as a white solid.

^{1}H NMR (500 MHz, CD_3OD) δ 8.02–7.90 (m, 2H), 7.78–7.69 (m, 1H), 7.63 (dd, J = 8.5, 7.0 Hz, 2H), 3.63 (dd, J = 14.3, 3.2 Hz, 1H), 3.25 (tdd, J = 9.7, 7.2, 4.8 Hz, 1H), 3.14 (dd, J = 14.3, 7.9 Hz, 1H), 2.55 (qd, J = 7.0, 5.0 Hz, 2H), 1.21 (d, J = 6.6 Hz, 3H), 1.01 (t, J = 7.0 Hz, 3H).

^{13}C NMR (126 MHz, CD_3OD) δ 141.5, 135.1, 130.7, 129.2, 59.7, 58.0, 51.1, 13.1.

FTIR (thin film) cm^{-1}: 3466 (br), 2978 (w), 2937 (w), 2881 (w), 1446 (m), 1401 (w), 1305 (s), 1148 (s), 1085 (m), 924 (w), 887 (w), 842 (w), 749 (m), 719 (w), 690 (m).

HRMS (ESI) (*m/z*): calc'd for $C_{11}H_{17}NO_3S$ $[M+H]^+$: 244.1002, found: 244.1003.

TLC (50% ethyl acetate in hexane), R*f*: 0.37 ($KMnO_4$).

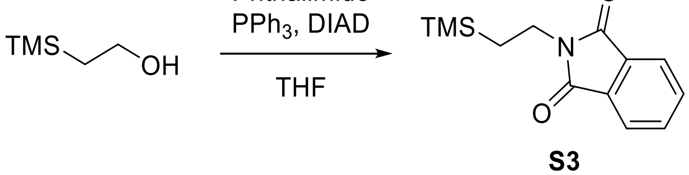

S3

2-(2-(Trimethylsilyl)ethyl)isoindoline-1,3-dione (S3):
Diisopropyl azodicarboxylate (DIAD, 2.17 mL, 11.0 mmol, 1.10 equiv) was added dropwise to a solution of 2-(trimethylsilyl)ethan-1-ol (1.43 mL, 10.0 mmol, 1 equiv), phthalimide (1.62 g, 11.0 mmol, 1.10 equiv), and triphenylphosphine (2.89 g, 11.0 mmol, 1.10 equiv) in tetrahydrofuran (36 mL) at 0 °C. The reaction mixture was stirred at room temperature for 24 h. The mixture was concentrated under reduced pressure. The crude mixture was dissolved in a minimal amount of 50% diethyl ether in hexanes until white precipitates formed. The precipitates were removed by filtration over a pad of Celite and washed with 50% diethyl ether in hexanes. The filtrate was concentrated under reduced pressure and the residue was purified by flash column chromatography on silica gel (eluent: 10→20% ethyl acetate in hexanes) to provide product **S3**(1.92 g, 77%) as a white solid.

^{1}H NMR (500 MHz, $CDCl_3$) δ 7.81 (dd, *J* = 5.4, 3.0 Hz, 2H), 7.67 (dd, *J* = 5.5, 3.0 Hz, 2H), 3.75–3.62 (m, 2H), 1.07–0.91 (m, 2H), 0.05 (s, 9H).

^{13}C NMR (126 MHz, $CDCl_3$) δ 168.5, 134.0, 132.6, 123.3, 34.7, 17.3, −1.5.

FTIR (thin film) cm^{-1}: 2952 (w), 2896 (w), 1770 (w), 1707 (s), 1613 (w), 1442 (w), 1394 (m), 1349 (m), 1249 (m), 1181 (w), 1070 (m), 853 (m), 764 (w), 708 (m).

HRMS (ESI) (*m*/*z*): calc'd for $C_{13}H_{17}NO_2Si$ $[M+H]^+$: 248.1107, found: 248.1101.

TLC (25% ethyl acetate in hexanes), R*f*: 0.75 (UV).

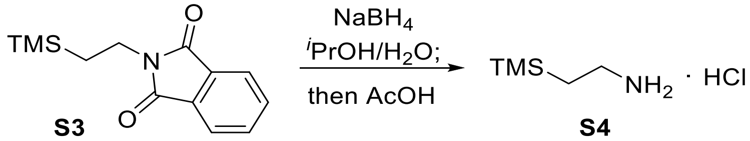

2-(Trimethylsilyl)ethan-1-amine hydrochloride (S4):
Sodium borohydride (1.18 g, 31.3 mmol, 5.00 equiv) was added to a solution of **S3** (1.55 g, 6.27 mmol, 1 equiv) in a mixture of isopropanol (54 mL) and water (9 mL). The reaction mixture was stirred at room temperature for 12 h, after which glacial acetic acid (6.3 mL) was added dropwise at room temperature over a period of 5 min. Once gas evolution has ceased, the reaction mixture was heated to 85 °C for 9 h. The reaction mixture was then basified with 1 M aqueous sodium hydroxide (125 mL), and extracted with dichloromethane (2 × 125 mL). The combined organic layers were sequentially washed with brine (50 mL) and water (50 mL), and then acidified with 1 M solution of hydrochloric acid (100 mL). The aqueous layer was separated and sequentially washed with dichloromethane (50 mL) and ethyl acetate (50 mL). The resulting aqueous layer was concentrated *in vacuo* to provide **S4** (813 mg, 84 %) as a white, crystalline solid.

^{1}H NMR (500 MHz, CD_3OD) δ 3.01–2.93 (m, 2H), 1.07–0.86 (m, 2H), 0.09 (s, 9H).

^{13}C NMR (126 MHz, CD_3OD) δ 38.3, 17.1, −1.9.

FTIR (thin film) cm^{-1}: 3325 (br), 2945 (w), 2833 (w), 1633 (w), 1379 (w), 1252 (w), 1118 (w), 1021 (s), 835 (m), 764 (w), 697 (w).

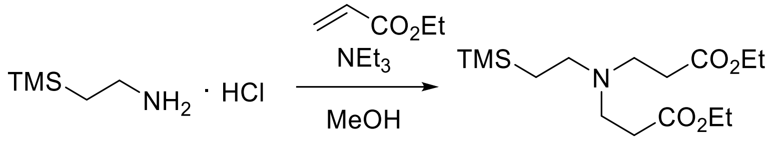

S4 **S5**

Diethyl 3,3'-((2-(trimethylsilyl)ethyl)azanediyl)dipropionate (S5):

Ethyl acrylate (532 µL, 5.00 mmol, 2.50 equiv) was added to a solution of **S4** (307 mg, 2.00 mmol, 1 equiv) and triethylamine (557 µL, 4.00 mmol, 2.00 equiv) in methanol (4 mL). The resulting solution was stirred at room temperature for 24 h. The resulting mixture was concentrated under reduced pressure and purified by flash column chromatography on silica gel (eluent: 25% ethyl acetate in hexanes) to provide **S5** (352 mg, 55%) as a pale yellow oil.

^{1}H NMR (500 MHz, $CDCl_3$) δ 4.10 (q, J = 7.1 Hz, 4H), 2.74 (t, J = 7.3 Hz, 4H), 2.54–2.45 (m, 2H), 2.40 (t, J = 7.3 Hz, 4H), 1.23 (t, J = 7.1 Hz, 6H), 0.74–0.54 (m, 2H), −0.03 (s, 9H).

^{13}C NMR (126 MHz, $CDCl_3$) δ 172.9, 60.5, 49.1, 48.5, 32.8, 14.4, 13.7, −1.4.

FTIR (thin film) cm^{-1}: 2952 (w), 2818 (w), 1733 (s), 1372 (m), 1249 (m), 1174 (s), 1096 (w), 1044 (m), 939 (s), 857 (s).

HRMS (ESI) (*m*/*z*): calc'd for $C_{15}H_{31}NO_4Si$ $[M+H]^+$: 318.2101, found: 318.2094.

TLC (25% ethyl acetate in hexanes), R*f*: 0.26 ($KMnO_4$).

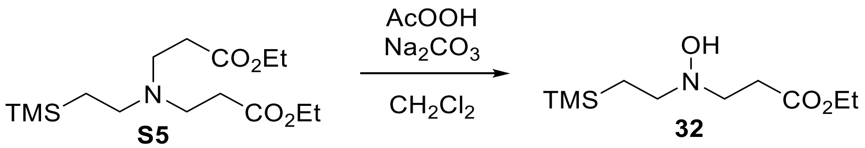

S5 **32**

Ethyl 3-(hydroxy(2-(trimethylsilyl)ethyl)amino)propanoate (32):

Peracetic acid (39% in acetic acid, 24.6 µL, 228 µmol, 1.10 equiv) was added to a solution of **S5** (65.8 mg, 207 µmol, 1 equiv) and sodium carbonate (65.8 mg, 621 µmol, 3.00 equiv) in dichloromethane (1 mL) at 0 °C. The resulting solution was stirred at room temperature 3 h. The resulting mixture was diluted with hexanes and purified by flash column chromatography on silica gel (eluent: 25% ethyl acetate in hexanes) to provide **32** (15.1 mg, 31%) as a pale yellow oil.

^{1}H NMR (500 MHz, CD_3OD) δ 4.13 (q, *J* = 7.1 Hz, 2H), 2.98–2.80 (m, 2H), 2.72–2.65 (m, 2H), 2.58 (t, *J* = 6.9 Hz, 2H), 1.25 (t, *J* = 7.1 Hz, 3H), 0.85 (t, *J* = 8.9 Hz, 2H), 0.02 (s, 9H).

^{13}C NMR (126 MHz, CD_3OD) δ 174.6, 61.7, 57.9, 56.3, 33.8, 15.7, 14.6, −1.4.

FTIR (thin film) cm^{-1}: 3455 (br), 2952 (w), 2896 (w), 2848 (w), 1737 (s), 1372 (w), 1249 (s), 1178 (s), 1096 (w), 1036 (w), 861 (s), 764 (w), 742 (w), 693 (w).

HRMS (ESI) (*m*/*z*): calc'd for $C_{10}H_{23}NO_3Si$ $[M+H]^+$: 234.1525, found: 234.1520.

TLC (33% ethyl acetate in hexanes), R*f*: 0.63 ($KMnO_4$).

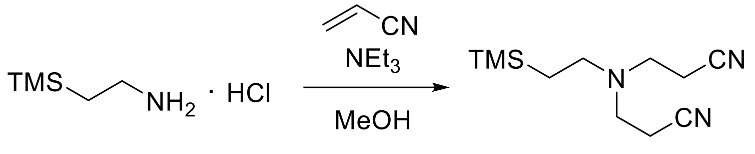

S4 **S6**

3,3'-((2-(trimethylsilyl)ethyl)azanediyl)dipropanenitrile (S6):

Acrylonitrile (327 µL, 5.00 mmol, 2.50 equiv) was added to a solution of **S4** (307 mg, 2.00 mmol, 1 equiv) and triethylamine (557 µL, 4.00 mmol, 2.00 equiv) in methanol (4 mL). The resulting solution was stirred at room temperature for 3 h. The resulting mixture was concentrated under reduced pressure and purified by flash column chromatography on silica gel (eluent: 33% ethyl acetate in hexanes) to provide **S6** (384 mg, 86%) as a colorless liquid.

^{1}H NMR (500 MHz, $CDCl_3$) δ 2.84 (t, J = 6.9 Hz, 4H), 2.65–2.57 (m, 2H), 2.46 (t, J = 6.9 Hz, 4H), 0.75–0.67 (m, 2H), 0.02 (s, 9H).

^{13}C NMR (126 MHz, $CDCl_3$) δ 118.8, 48.9, 48.9, 17.1, 13.8, −1.5.

FTIR (thin film) cm^{-1}: 2952 (w), 2833 (w), 2247 (w), 1420 (w), 1361 (w), 1249 (m), 1126 (m), 1044 (w), 857 (m), 835 (s), 760 (m), 693 (m).

HRMS (ESI) (*m*/*z*): calc'd for $C_{11}H_{21}N_3Si$ [M+H]$^+$: 224.1578, found: 224.1580.

TLC (33% ethyl acetate in hexanes), R*f*: 0.31 ($KMnO_4$).

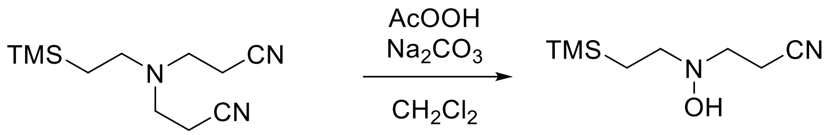

S6 **33**

3-(hydroxy(2-(trimethylsilyl)ethyl)amino)propanenitrile (33):

Peracetic acid (39% in acetic acid, 181 µL, 1.67 mmol, 1.10 equiv) was added to a solution of **S6** (340 mg, 1.52 mmol, 1 equiv) and sodium carbonate (484 mg, 4.57 mmol, 3.00 equiv) in dichloromethane (7.6 mL) at 0 °C. The resulting solution was stirred at room temperature for 3 h. The reaction was then diluted with hexanes and purified by flash column chromatography on silica gel (eluent: 15% acetone in hexanes) to provide **33** (90.3 mg, 32%) as a pale yellow solid.

^{1}H NMR (500 MHz, CD_3OD) δ 2.83 (d, J = 6.7 Hz, 2H), 2.75–2.58 (m, 4H), 0.91–0.78 (m, 2H), 0.03 (s, 9H).

^{13}C NMR (126 MHz, CD_3OD) δ 120.4, 57.6, 55.9, 16.6, 15.5, −1.5.

FTIR (thin film) cm^{-1}: 3425 (br), 2952 (w), 2896 (w), 2848 (w), 2251 (w), 1420 (w), 1349 (w), 1249 (m), 1066 (w), 936 (w), 831 (s), 753 (w), 693 (w).

HRMS (ESI) (*m/z*): calc'd for $C_8H_{18}N_2OSi$ $[M+H]^+$: 187.1261, found: 187.1263.

TLC (15% acetone in hexanes), R*f*: 0.25 ($KMnO_4$).

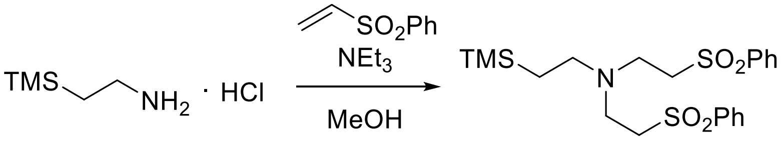

S4 **S7**

2-(Phenylsulfonyl)-*N*-(2-(phenylsulfonyl)ethyl)-*N*-(2-(trimethylsilyl)ethyl)ethan-1-amine (S7):

Phenyl vinyl sulfone (420 mg, 2.50 mmol, 2.50 equiv) was added to a solution of **S4** (154 mg, 1.00 mmol, 1 equiv) and triethylamine (280 µL, 2.00 mmol, 2.00 equiv) in methanol (2 mL). The resulting solution was stirred at room temperature for 18 h. The resulting mixture was concentrated under reduced pressure and purified by flash column chromatography on silica gel (eluent: 33% ethyl acetate in hexanes) to provide **S7** (381 mg, 84%) as a pale yellow foam.

^{1}H NMR (500 MHz, CD_3OD) δ 8.01–7.93 (m, 4H), 7.83–7.74 (m, 2H), 7.72–7.63 (m, 4H), 3.64 (dd, J = 8.5, 6.1 Hz, 4H), 3.41 (dd, J = 8.5, 6.0 Hz, 4H), 3.14–3.00 (m, 2H), 0.83–0.63 (m, 2H), 0.03 (s, 9H).

^{13}C NMR (126 MHz, CD_3OD) δ 139.9, 136.0, 131.0, 129.5, 53.3, 50.9, 46.7, 12.8, −1.8.

FTIR (thin film) cm^{-1}: 3064 (w), 2956 (w), 1670 (m), 1446 (w), 1416 (w), 1308 (m), 1252 (w), 1179 (m), 1144 (s), 1085 (m), 835 (m), 798 (w), 745 (m), 719 (m), 686 (m).

HRMS (ESI) (*m/z*): calc'd for $C_{21}H_{31}NO_4S_2Si$ $[M+H]^+$: 454.1542, found: 454.1535.

TLC (50% ethyl acetate in hexanes), R*f*: 0.53 (UV, $KMnO_4$).

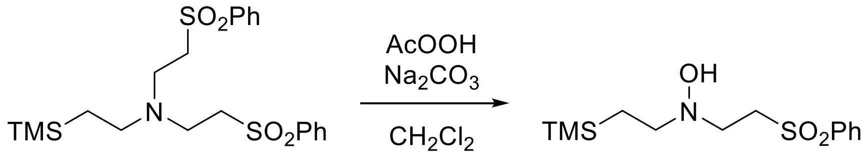

S7 **34**

***N*-(2-(Phenylsulfonyl)ethyl)-*N*-(2-(trimethylsilyl)ethyl)hydroxylamine (34):**

Peracetic acid (39% in acetic acid, 119 µL, 1.10 mmol, 1.10 equiv) was added to a solution of **S7** (467 mg, 1.00 mmol, 1 equiv) and sodium carbonate (318 mg, 3.00 mmol, 3.00 equiv) in dichloromethane (5 mL) at 0 °C. The resulting solution was stirred at room temperature for 3 h. The resulting mixture was then diluted with hexanes and purified by flash column chromatography on silica gel (eluent: 20% acetone in hexanes) to provide **34** (104 mg, 34%) as a pale yellow solid.

^{1}H NMR (500 MHz, CD_3OD) δ 7.97–7.91 (m, 2H), 7.78–7.71 (m, 1H), 7.65 (t, *J* = 7.8 Hz, 2H), 3.47 (t, *J* = 7.5 Hz, 2H), 2.97–2.83 (m, 2H), 2.65–2.58 (m, 2H), 0.85–0.69 (m, 2H), −0.01 (s, 9H).

^{13}C NMR (126 MHz, CD_3OD) δ 140.9, 135.3, 130.7, 129.2, 57.8, 55.1, 54.0, 15.7, −1.4.

FTIR (thin film) cm^{-1}: 3459 (br), 3068 (w), 2952 (w), 2896 (w), 2848 (w), 1446 (m), 1305 (m), 1249 (m), 1144 (s), 1085 (m), 835 (s), 745 (m), 690 (m).

HRMS (ESI) (*m*/*z*): calc'd for $C_{13}H_{23}NO_3SSi$ $[M+H]^+$: 302.1246, found: 302.1237.

TLC (20% acetone in hexanes), R*f*: 0.26 (UV, $KMnO_4$).

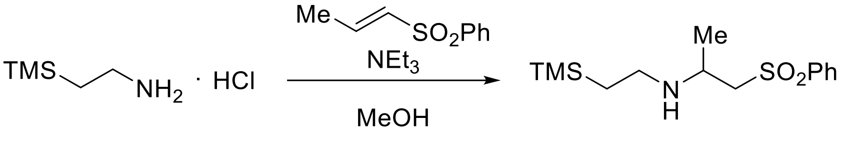

S4 **S8**

1-(Phenylsulfonyl)-N-(2-(trimethylsilyl)ethyl)propan-2-amine (S8):

(*E*)-1-(Phenylsulfonyl)-1-propene[6] (547 mg, 3.00 mmol, 1.50 equiv) was added to a solution of **S4** (307 mg, 2.00 mmol, 1 equiv) and triethylamine (560 µL, 4.00 mmol, 2.00 equiv) in methanol (4 mL). The resulting solution was stirred at room temperature for 18 h. The resulting mixture was concentrated under reduced pressure and purified by flash column chromatography on silica gel (eluent: 3% methanol in dichloromethane) to provide product **S8** (485 mg, 81%) as a colorless oil.

^{1}H NMR (500 MHz, CD_3OD) δ 8.04–7.93 (m, 2H), 7.82–7.73 (m, 1H), 7.73–7.61 (m, 2H), 3.66–3.54 (m, 2H), 3.53–3.41 (m, 1H), 2.97–2.83 (m, 2H), 1.40 (d, J = 6.6 Hz, 3H), 0.93–0.79 (m, 2H), 0.06 (s, 9H).

^{13}C NMR (126 MHz, CD_3OD) δ 140.7, 135.8, 131.0, 129.3, 59.5, 50.1, 43.9, 18.8, 16.4, −1.7.
FTIR (thin film) cm^{-1}: 3332(br), 2952 (w), 2900 (w), 1446 (m), 1305 (s), 1245 (m), 1144 (s), 1085 (m), 835 (s), 745 (m), 690 (s).

HRMS (ESI) (*m*/*z*): calc'd for $C_{14}H_{25}NO_2SSi$ $[M+H]^+$: 300.1448, found: 300.1451.

TLC (5% methanol in dichloromethane), R*f*: 0.16 (UV, $KMnO_4$).

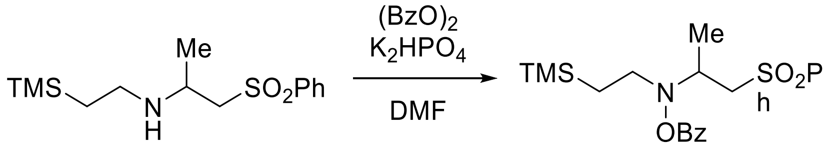

S8 **S9**

***O*-benzoyl-*N*-(1-(phenylsulfonyl)propan-2-yl)-*N*-(2-(trimethylsilyl)ethyl)hydroxylamine (S9):**

Benzoyl peroxide (182 mg, 750 µmol, 1.50 equiv) was added to a solution of **S8** (150 mg, 500 µmol, 1 equiv) and potassium phosphate dibasic (348 mg, 2.00 mmol, 4.00 equiv) in *N,N*-dimethylformamide (2.50 mL). The resulting solution was stirred at room temperature for 4 h after which additional benzoyl peroxide (60.6 mg, 250 µmol, 0.50 equiv) and potassium phosphate dibasic (174 mg, 1.00 mmol, 2.00 equiv) were added. After stirring for an additional 12 h at room temperature, the resulting mixture was diluted with ethyl acetate, washed with saturated aqueous

sodium bicarbonate, and extracted with ethyl acetate (3 × 10 mL). The combined organic layers were washed with brine (2 × 50 mL), dried over anhydrous magnesium sulfate, filtered, and concentrated under reduced pressure. The resulting crude residue was purified by flash column chromatography on silica gel (eluent: 20% ethyl acetate in hexanes) to provide product **S9** (197 mg, 94%) as a colorless gum.

^{1}H NMR (500 MHz, CD_3OD) δ 8.04–7.79 (m, 4H), 7.70–7.63 (m, 1H), 7.62 (td, J = 7.4, 1.3 Hz, 1H), 7.58 (t, J = 7.8 Hz, 2H), 7.52–7.43 (m, 2H), 3.78 (dd, J = 14.5, 2.1 Hz, 1H), 3.67 (tt, J = 8.7, 6.6 Hz, 1H), 3.30 (dd, J = 14.6, 8.5 Hz, 1H), 3.03–2.83 (m, 2H), 1.35 (d, J = 6.6 Hz, 3H), 0.79–0.53 (m, 2H), −0.03 (s, 9H).

^{13}C NMR (126 MHz, CD_3OD) δ 167.3, 140.8, 135.3, 134.9, 130.7, 130.6, 129.9, 129.8, 129.3, 59.0, 56.8, 51.7, 17.7, 15.3, −1.5, −1.7.

FTIR (thin film) cm^{-1}: 3064 (w), 2952 (w), 2896 (w), 1741 (m), 1450 (w), 1305 (m), 1245 (s), 1178 (w), 1148 (s), 1085 (m), 1055 (m), 1025 (m), 839 (m), 749 (w), 708 (m).

HRMS (ESI) (*m*/*z*): calc'd for $C_{21}H_{29}NO_4SSi$ $[M+H]^+$: 420.1665, found: 420.1662.

TLC (33% ethyl acetate in hexanes), R*f*: 0.60 (UV, $KMnO_4$).

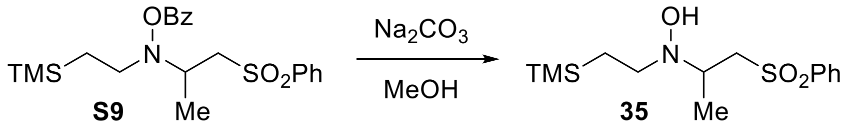

***N*-(1-(phenylsulfonyl)propan-2-yl)-*N*-(2-(trimethylsilyl)ethyl)hydroxylamine (35):**

Sodium carbonate (53.0 mg, 500 µmol, 2.50 equiv) was added to a solution of **S9** (83.9 mg, 200 µmol, 1 equiv) in methanol (2 mL) at 0 °C. The resulting solution was stirred at room temperature for 16 h. The resulting mixture was concentrated under reduced pressure and the resulting residue was purified by flash column chromatography on silica gel (eluent: 25% ethyl acetate in hexanes) to provide **35** (52.4 mg, 83%) as a colorless oil.

^{1}H NMR (500 MHz, CD_3OD) δ 7.98–7.90 (m, 2H), 7.78–7.70 (m, 1H), 7.68–7.60 (m, 2H), 3.61 (dd, J = 14.5, 2.9 Hz, 1H), 3.30–3.22 (m, 1H), 3.12 (dd, J = 14.4, 8.0 Hz, 1H), 2.68–2.48 (m, 2H), 1.22 (d, J = 6.6 Hz, 3H), 0.67 (td, J = 7.7, 4.6 Hz, 2H), −0.02 (s, 9H).

^{13}C NMR (126 MHz, CD_3OD) δ 141.4, 135.1, 130.7, 129.2, 59.5, 57.0, 53.0, 16.7, 15.8, −1.3.
FTIR (thin film) cm^{-1}: 3466 (br), 2952 (w), 2892 (w), 1446 (w), 1305 (m), 1249 (m), 1148 (s), 1085 (m), 928 (w), 835 (s), 749 (m), 690 (m).

HRMS (ESI) (*m*/*z*): calc'd for $C_{14}H_{25}NO_3SSi$ $[M+H]^+$: 316.1403, found: 316.1396.

TLC (33% ethyl acetate in hexanes), R*f*: 0.40 (UV, $KMnO_4$).

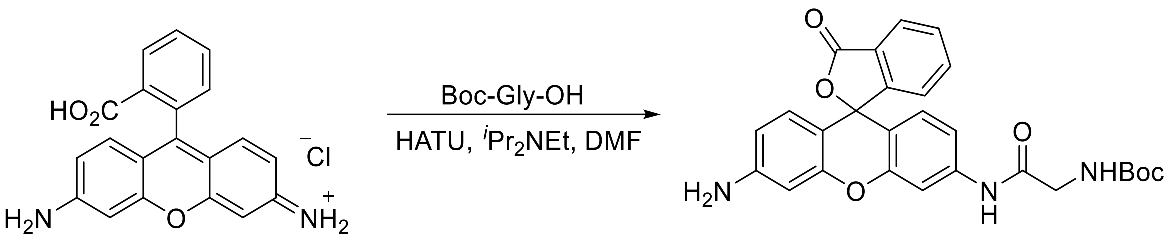

S10 **S11**

***tert*-Butyl (2-((3'-amino-3-oxo-3H-spiro[isobenzofuran-1,9'-xanthen]-6'-yl)amino)-2-oxoethyl)carbamate (S11):**

1-[Bis(dimethylamino)methylene]-1H-1,2,3-triazolo[4,5-b]pyridinium 3-oxide hexafluorophosphate (HATU, 126 mg, 330 µmol, 1.10 equiv) was added to a solution of rhodamine 110 hydrochloride (**S10**, 110 mg, 300 µmol, 1 equiv), *N*-(*tert*-butoxycarbonyl)glycine (57.8 mg, 330 µmol, 1.10 equiv), and *N*,*N*-diisopropylethylamine (DIPEA, 157 µL, 900 µmol, 3.00 equiv) in *N*,*N*-dimethylformamide (3 mL). The resulting mixture was stirred for 1 h after which it was diluted with water and purified by automated C_{18} reverse phase column chromatography (30 g C_{18} silica gel, 25 µm spherical particles, eluent: H_2O + 0.1% TFA (5 CV), gradient 0→100% MeCN/H_2O + 0.1% TFA (15 CV)). Fractions containing the desired product were collected and concentrated under reduced pressure. The resulting residue was then further purified by flash column chromatography on silica gel (eluent: 10→20% CMA in chloroform) to provide the product **S11** (77.0 mg, 53%) as an orange solid.

^{1}H NMR (500 MHz, $CDCl_3$) δ 7.96 (d, J = 7.8 Hz, 1H), 7.66–7.58 (m, 2H), 7.56 (td, J = 7.4, 1.1 Hz, 1H), 7.09 (d, J = 7.5 Hz, 1H), 6.96 (dd, J = 8.6, 2.2 Hz, 1H), 6.62 (d, J = 8.6 Hz, 1H), 6.48 (d, J = 8.5 Hz, 1H), 6.42 (d, J = 2.3 Hz, 1H), 6.28 (dd, J = 8.5, 2.3 Hz, 1H), 3.94 (d, J = 9.5 Hz, 1H), 3.90 (s, 2H), 1.43 (s, 9H).

^{13}C NMR (126 MHz, $CDCl_3$, peaks in parenthesis are split from rotamer) δ 170.1, (168.5, 168.4), 156.8, 153.4, 152.6, 152.0, 149.2, (139.6, 139.5), 135.3, 129.8, 129.1, 128.7, 126.9, 125.1, 124.2, (115.3, 115.3), 114.9, 111.9, (108.2, 108.0), 107.9, 101.7, 84.0, 80.9, 45.6, 28.5.

FTIR (thin film) cm^{-1}: 3358 (m), 3056 (w), 2978 (w), 2929 (w), 1744 (m), 1684 (m), 1610 (s), 1505 (s), 1453 (m), 1416 (s), 1368 (m), 1286 (m), 1249 (s), 1163 (s), 1110 (s), 1051 (m), 1028 (w), 943 (w), 864 (m), 790 (m), 734 (m), 693 (m).

HRMS (ESI) (*m*/*z*): calc'd for $C_{27}H_{25}N_3O_6$ $[M+H]^+$: 488.1822, found: 488.1818.

TLC (20% CMA in chloroform), R*f*: 0.24 (UV, $KMnO_4$).

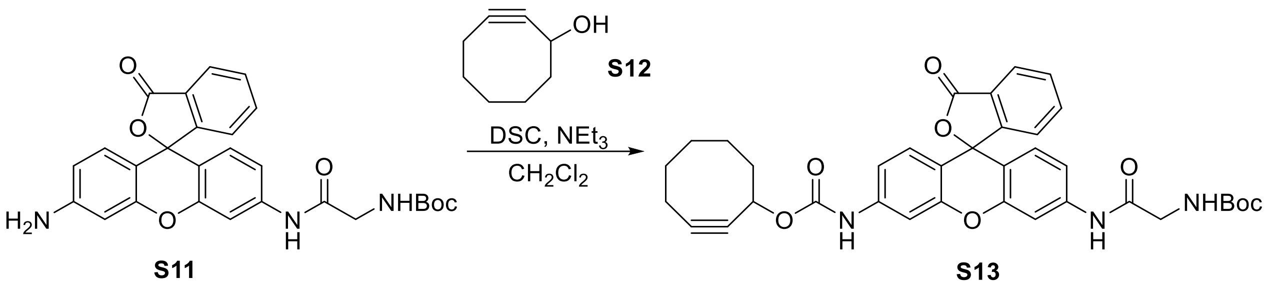

tert-Butyl (2-((3'-(((cyclooct-2-yn-1-yloxy)carbonyl)amino)-3-oxo-3H-spiro[isobenzofuran-1,9'-xanthen]-6'-yl)amino)-2-oxoethyl)carbamate (S13):

Triethylamine (88.0 µl, 632 µmol, 4.00 equiv) and *N,N'*-disuccinimidyl carbonate (80.9 mg, 316 µmol, 2.00 equiv) were added to a solution of cyclooct-2-yn-1-ol (**S12**, 39.2 mg, 316 µmol, 2.00 equiv)[4, 7] in dichloromethane (1.60 mL) at 0 °C. The resulting mixture was warmed to room temperature and stirred for 3 h after which Boc-protected glycinyl rhodamine **S11** (77.0 mg, 158 µmol, 1 equiv) was added to the reaction mixture. The solution was stirred for an additional 12 h. The resulting mixture was concentrated under reduced pressure, and the resulting crude residue was purified by flash column chromatography on silica gel (eluent: 50% ethyl acetate in hexanes→5% methanol in dichloromethane) to provide product **S13** (51.8 mg, 51%) as a pale yellow solid.

^{1}H NMR (500 MHz, CD_3OD) δ 8.01 (d, J = 7.6 Hz, 1H), 7.80–7.73 (m, 2H), 7.70 (td, J = 7.5, 1.0 Hz, 1H), 7.57 (dd, J = 7.1, 2.2 Hz, 1H), 7.20 (d, J = 7.5 Hz, 1H), 7.16 (dd, J = 8.6, 2.2 Hz, 1H), 7.06 (ddd, J = 9.0, 7.4, 2.2 Hz, 1H), 6.70 (d, J = 8.6 Hz, 1H), 6.66 (d, J = 8.7 Hz, 1H), 5.37–5.23 (m, 1H), 3.87 (s, 2H), 2.26 (dtd, J = 17.0, 6.5, 1.8 Hz, 1H), 2.23–2.13 (m, 2H), 2.08 (ddd, J = 14.2, 8.9, 6.4 Hz, 1H), 1.96–1.87 (m, 2H), 1.90–1.78 (m, 1H), 1.79–1.68 (m, 1H), 1.70–1.54 (m, 1H), 1.46 (s, 9H).

^{13}C NMR (126 MHz, CD_3OD) δ 171.5, 171.0, 158.8, 154.8, 154.6, 153.3, 153.2, 143.0, 142.2, 136.9, 131.4, 129.6 (2C overlapped), 127.9, 126.1, 125.3, 116.8, 115.8, 115.6, 114.3, 108.8, 107.1, 102.7, 92.1, 84.5, 80.9, 68.6, 45.2, 43.0, 35.5, 31.0, 28.9, 27.5, 21.3.

FTIR (thin film) cm^{-1}: 3447 (br), 2930 (w), 2855 (w), 2482 (w), 1696 (m), 1614 (m), 1510 (m), 1416 (s), 1394 (s), 1323 (m), 1260 (m), 1163 (m), 1126 (w), 1062 (m), 991 (w), 872 (w), 760 (w), 693 (w).

HRMS (ESI) (*m/z*): calc'd for $C_{36}H_{35}N_3O_8$ $[M+H]^+$: 638.2502, found: 638.2500.

TLC (50% ethyl acetate in hexanes), R*f*: 0.50 (UV, $KMnO_4$).

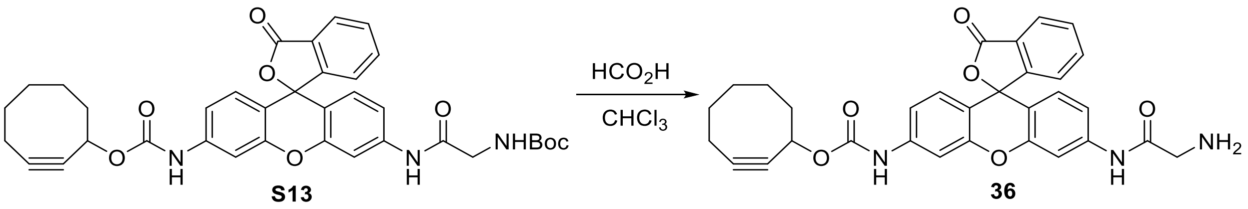

Cyclooctynyl glycinyl rhodamine (36):

A solution of formic acid in chloroform (70% v/v, 1 mL) was added via syringe to a vial charged with Boc-protected cyclooctynyl glycinyl rhodamine **S13** (24.2 mg, 37.9 µmol, 1 equiv) at room temperature. The reaction mixture was stirred at room temperature for 24 h then concentrated under reduced pressure. The crude mixture was diluted with water and purified by automated C_{18} reverse phase column chromatography (30 g C_{18} silica gel, 25 µm spherical particles, eluent: H_2O + 0.1% TFA (5 CV), gradient 0→100% MeCN/H_2O + 0.1% TFA (15 CV)). Fractions containing the desired product were collected and concentrated under reduced pressure. The resulting residue was further purified by flash column chromatography on silica gel (eluent: 60% CMA in chloroform) to provide product **36** (11.6 mg, 57%) as a pale yellow solid.

^{1}H NMR (500 MHz, CD_3OD) δ 8.02 (d, *J* = 7.8 Hz, 1H), 7.81 (d, *J* = 2.1 Hz, 1H), 7.77 (td, *J* = 7.5, 1.2 Hz, 1H), 7.71 (td, *J* = 7.5, 1.0 Hz, 1H), 7.59 (dd, *J* = 7.2, 2.1 Hz, 1H), 7.21 (dd, *J* = 7.7, 1.0 Hz, 1H), 7.17 (dd, *J* = 8.7, 2.1 Hz, 1H), 7.06 (ddd, *J* = 9.2, 7.4, 2.2 Hz, 1H), 6.71 (d, *J* = 8.6 Hz, 1H), 6.67 (d, *J* = 8.6 Hz, 1H), 5.33–5.26 (m, 1H), 3.44 (s, 2H), 2.27 (dtd, *J* = 17.1, 6.4, 1.8 Hz, 1H), 2.24–2.14 (m, 2H), 2.08 (ddd, *J* = 14.2, 8.8, 6.4 Hz, 1H), 1.96–1.88 (m, 2H), 1.91–1.80 (m, 1H), 1.80–1.69 (m, 1H), 1.71–1.54 (m, 2H).

^{13}C NMR (126 MHz, $CDCl_3$) δ 171.3, 169.8, 153.5, 152.5, 152.0, 151.9, 140.2, 139.8, 135.4, 130.0, 128.8, 128.7, 126.5, 125.2, 124.1, 115.3, 114.5, 114.4, 113.7, 107.6, 106.5, 102.6, 90.6, 68.0, 45.2, 42.0, 34.4, 29.8, 26.3, 20.9, 18.6.

FTIR (thin film) cm^{-1}: 3310 (br), 2930 (m), 2851 (w), 1744 (m), 1614 (s), 1528 (m), 1409 (s), 1323 (w), 1290 (m), 1230 (s), 1085 (w), 1051 (m), 872 (w), 760 (w), 693 (w).

HRMS (ESI) (*m/z*): calc'd for $C_{31}H_{27}N_3O_6$ $[M+H]^+$: 538.1978, found: 538.1975.

TLC (70% CMA in chloroform), R*f*: 0.55 (UV, $KMnO_4$).

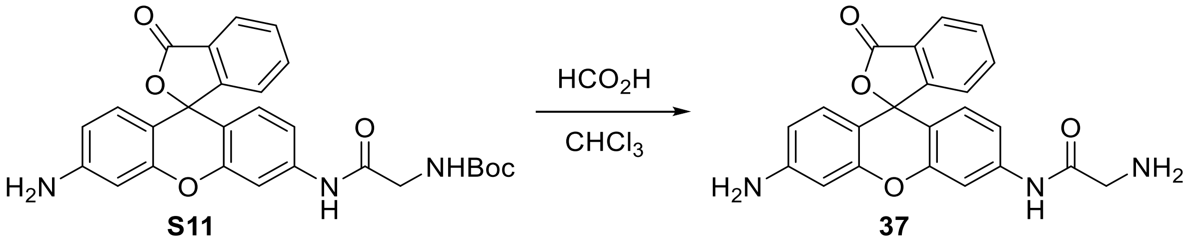

Glycinyl rhodamine (37):

Formic acid in chloroform (70% v/v, 500 µL) was added via syringe to a vial charged with **S11** (10 mg, 20.5 µmol, 1 equiv) at room temperature. The reaction mixture was stirred for 24 h then concentrated under reduced pressure. The crude mixture was diluted with water and purified by automated C_{18} reverse phase column chromatography (30 g C_{18} silica gel, 25 µm spherical particles, eluent: H_2O + 0.1% TFA (5 CV), gradient 0→100% MeCN/H_2O + 0.1% TFA (15 CV)). Fractions containing the desired product were collected and concentrated under reduced pressure. The resulting residue was further purified by flash column chromatography on silica gel (eluent: 100% CMA) to provide **37** (7.4 mg, 93%) as a red solid.

^{1}H NMR (500 MHz, CD_3OD) δ 8.00 (d, *J* = 7.6 Hz, 1H), 7.79 (d, *J* = 2.1 Hz, 1H), 7.76 (td, *J* = 7.5, 1.2 Hz, 1H), 7.73–7.66 (m, 1H), 7.20 (d, *J* = 7.6 Hz, 1H), 7.13 (dd, *J* = 8.7, 2.1 Hz, 1H), 6.69

(d, J = 8.6 Hz, 1H), 6.56 (d, J = 2.2 Hz, 1H), 6.46 (d, J = 8.6 Hz, 1H), 6.42 (dd, J = 8.6, 2.2 Hz, 1H), 3.62 (s, 2H).

^{13}C NMR (126 MHz, CD_3OD) δ 168.4, 167.1, 163.4, 162.2, 161.1, 156.4, 147.0, 136.7, 134.5, 134.2, 132.3, 132.0, 131.9, 131.4, 131.0, 121.0, 119.1, 119.0, 118.3, 107.6, 99.1, 42.8.

FTIR (thin film) cm^{-1}: 3351 (br), 3224 (w), 2922 (w), 1744 (m), 1689 (w), 1610 (s), 1506 (n), 1453 (w), 1413 (m), 1342 (w), 1290 (w), 1252 (w), 1226 (w), 1193 (w), 1111 (w), 865 (w), 831 (w), 790 (w), 760 (w), 693 (w).

HRMS (ESI) (*m*/*z*): calc'd for $C_{22}H_{17}N_3O_4$ $[M+H]^+$: 388.1297, found: 388.1292.

TLC (100% CMA), R*f*: 0.41 (UV, $KMnO_4$).

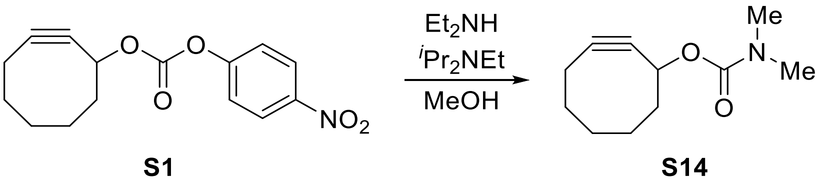

Cyclooct-2-yn-1-yl dimethylcarbamate (S14):
Dimethylamine (35.1 mg, 0.778 mmol, 1.50 equiv) and diisopropylethylamine (134.0 mg, 1.04 mmol, 2.00 equiv) were added sequentially to a solution of carbonate **S1** (150 mg, 0.518 mmol, 1 equiv), in methanol (6 mL) at 0 °C. The reaction mixture was stirred at room temperature for 1.5 h then concentrated under reduced pressure. The residue was purified by flash column chromatography on silica gel (eluent: 15% ethyl acetate in hexanes) to provide carbamate **S14** (80 mg, 76%) as a colorless oil.

^{1}H NMR (500 MHz, $CDCl_3$) δ 5.28–5.25 (ddd, J = 7.02, 4.50, 2.14, 1H), 2.89 (s, 6H), 2.29–2.20 (m, 1H), 2.03–1.99 (m, 1H), 1.82–1.85 (m, 2H), 1.85–1.81 (m, 1H), 1.68–1.64 (m, 2H), 1.53–1.50 (m, 1H).

^{13}C NMR (126 MHz, $CDCl_3$) δ 155.6, 100.9, 91.4, 67.3, 41.81, 36.24, 35.75, 29.6, 26.1, 20.6.

FTIR (thin film) cm^{-1}: 2928 (br), 2853 (w), 1699 (s), 1489 (w), 1394 (m), 1340 (w), 1273 (w), 1182 (s), 1045 (m), 957 (m), 900 (w), 766 (m), 623(w), 544(w), 406(m).

HRMS (ESI) (m/z): calc'd for $C_{11}H_{18}NO_2$ $[M+H]^+$: 196.1332, found: 196.1334.

TLC (15% ethyl acetate in hexane), Rf: 0.42 ($KMnO_4$).

N^6-((Cyclooct-2-yn-1-yloxy)carbonyl)-*L*-lysine (COTK, 38):
The compound was synthesized according to literature procedure.[5]

TAMRA-hydroxylamine (39):
The compound was synthesized according to literature procedure.[4]

References

(1) Nakatsu, T.; Ichiyama, S.; Hiratake, J.; Saldanha, A.; Kobashi, N.; Sakata, K.; Kato, H., Structural Basis for the Spectral Difference in Luciferase Bioluminescence. *Nature* **2006**, *440*, 372-6.

(2) Wilkovitsch, M.; Kuba, W.; Keppel, P.; Sohr, B.; Löffler, A.; Kronister, S.; Del Castillo, A. F.; Goldeck, M.; Dzijak, R.; Rahm, M.; Vrabel, M.; Svatunek, D.; Carlson, J. C. T.; Mikula, H., Transforming Aryl-Tetrazines into Bioorthogonal Scissors for Systematic Cleavage of trans-Cyclooctenes. *Angew. Chem. Int. Ed.* **2025**, *64*, e202411707.

(3) Rahm, M.; Keppel, P.; Šlachtová, V.; Dzijak, R.; Dračínský, M.; Bellová, S.; Reyes-Gutiérrez, P. E.; Štěpánová, S.; Raffler, J.; Tloušťová, E.; Mertlíková-Kaiserová, H.; Mikula, H.; Vrabel, M., Sulfonated Hydroxyaryl-Tetrazines with Increased pK(a) for Accelerated Bioorthogonal Click-to-Release Reactions in Cells. *Angew. Chem. Int. Ed.* **2025**, *64*, e202411713.

(4) Kang, D.; Cheung, S. T.; Wong-Rolle, A.; Kim, J., Enamine N-Oxides: Synthesis and Application to Hypoxia-Responsive Prodrugs and Imaging Agents. *ACS Cent. Sci.* **2021**, *7*, 631–640.

(5) Plass, T.; Milles, S.; Koehler, C.; Schultz, C.; Lemke, E. A., Genetically Encoded Copper-Free Click Chemistry. *Angewandte Chemie International Edition* **2011**, *50*, 3878-3881.

(6) Lee, J. W.; Lee, C.-W.; Jung, J. H.; Oh, D. Y., Facile Synthesis of Vinyl Sulfones from β-Bromo Alcohols. *Synth. Commun.* **2000**, *30*, 2897-2902.

(7) Hagendorn, T.; Bräse, S., A Route to Cyclooct-2-ynol and Its Functionalization by Mitsunobu Chemistry. *Eur. J. Org. Chem.* **2014**, *2014*, 1280-1286.

Parameter	Value
Solvent	MeOD
Temperature	298.2
Pulse Sequence	zg45
Experiment	1D
Number of Scans	4
Relaxation Delay	1.0000
Spectrometer Frequency	500.13
Nucleus	1H

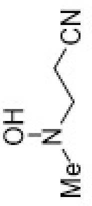

2

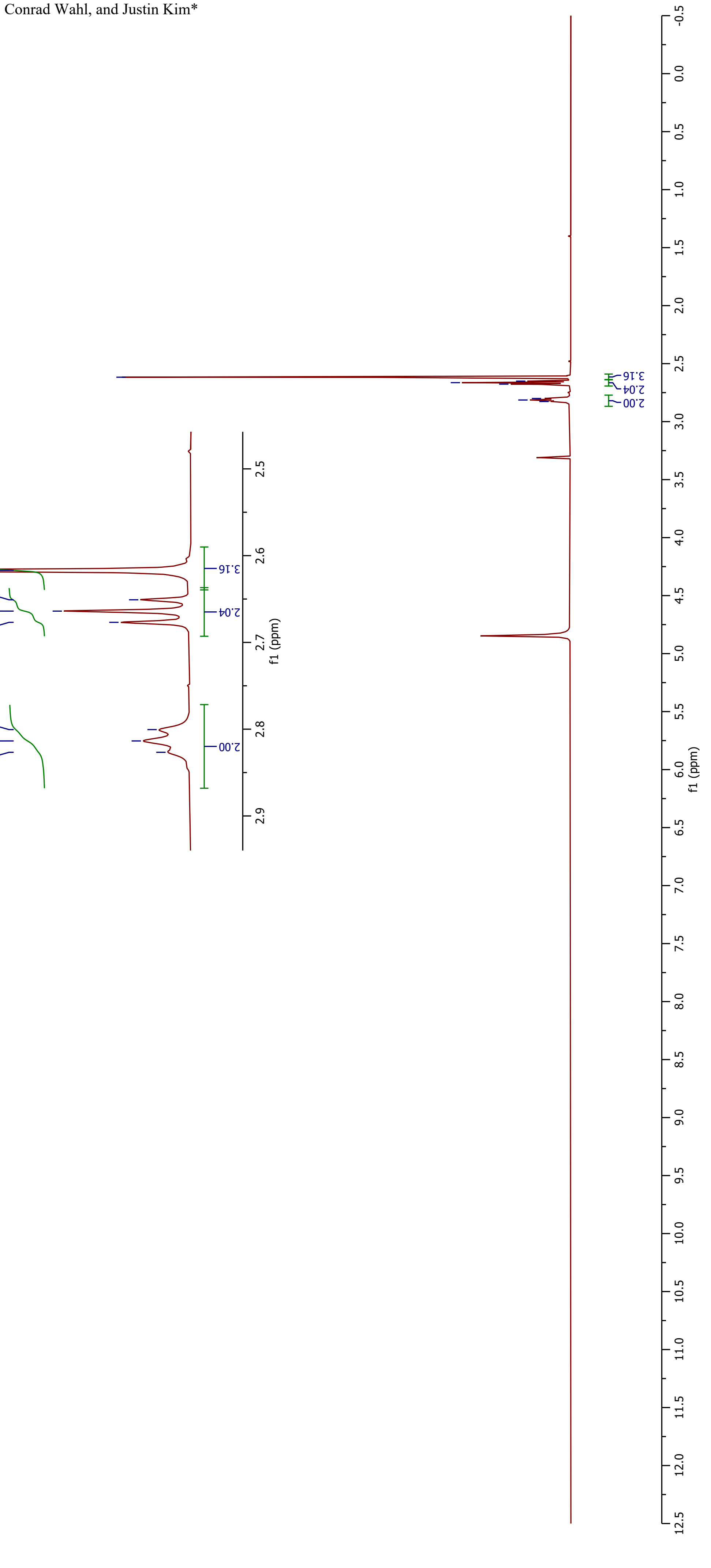

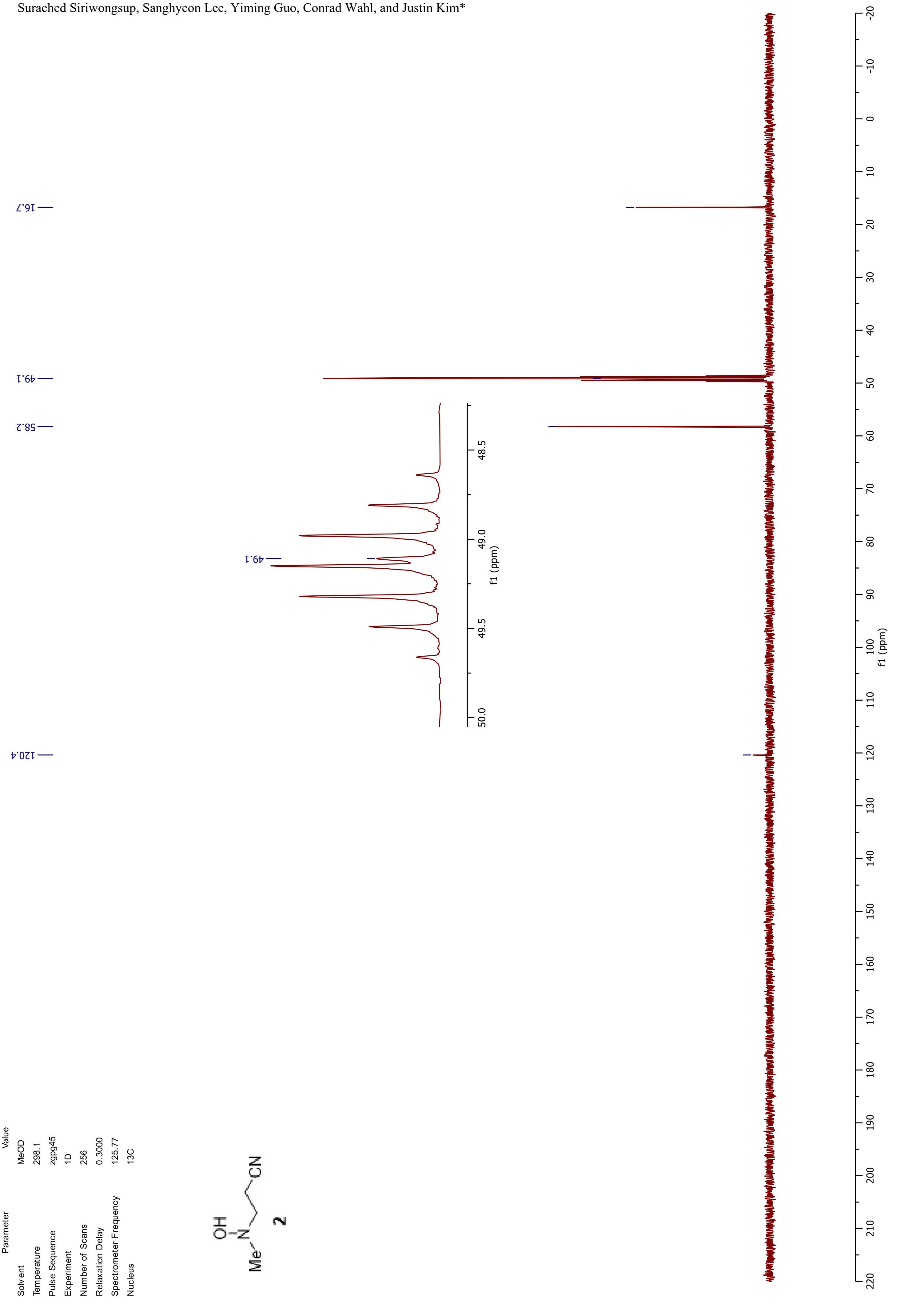

2

Parameter	Value
Solvent	MeOD
Temperature	298.1
Pulse Sequence	zgpg45
Experiment	1D
Number of Scans	256
Relaxation Delay	0.3000
Spectrometer Frequency	125.77
Nucleus	13C

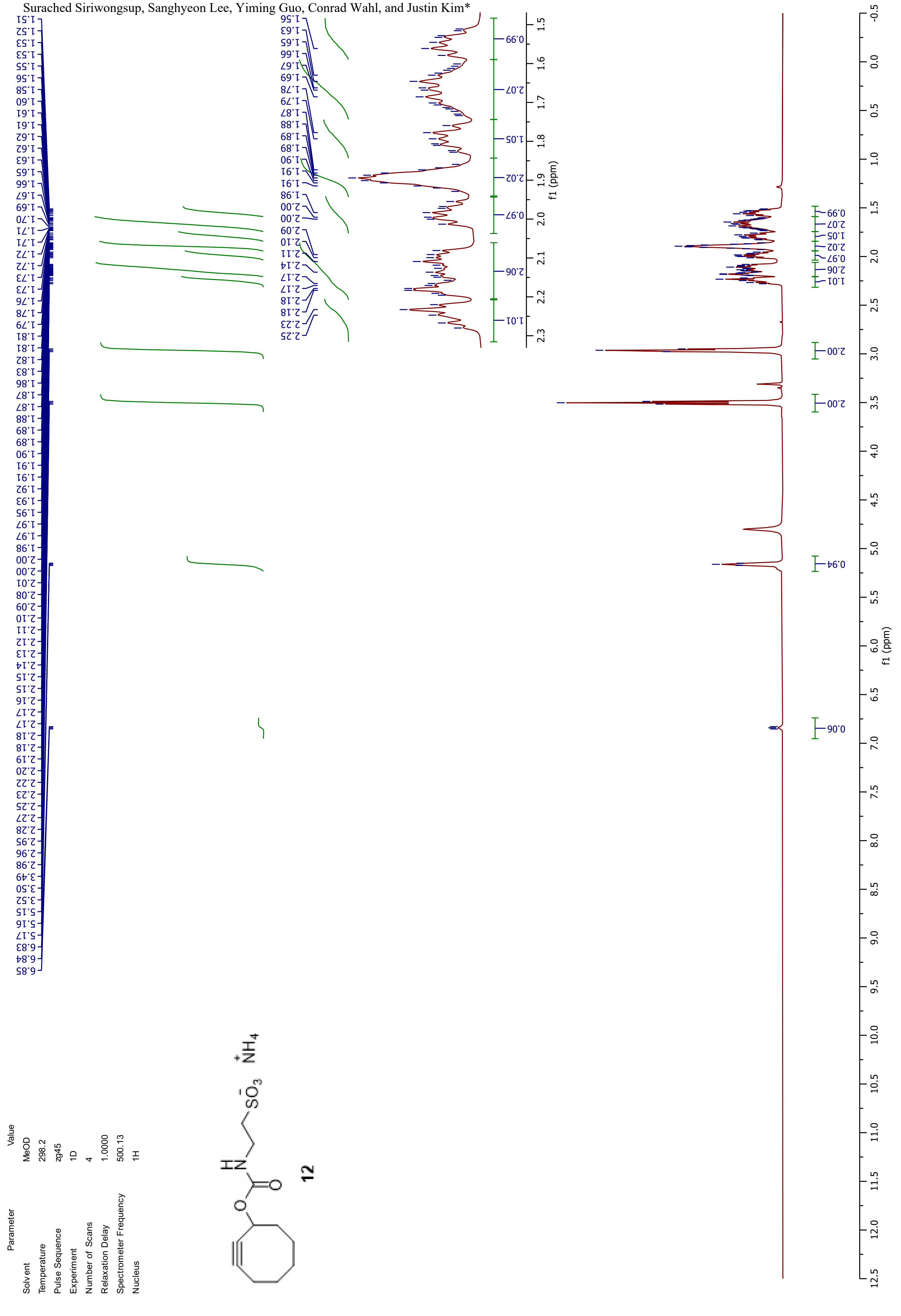
Parameter | Value
Solvent | MeOD
Temperature | 298.2
Pulse Sequence | zg45
Experiment | 1D
Number of Scans | 4
Relaxation Delay | 1.0000
Spectrometer Frequency | 500.13
Nucleus | 1H
12
f1 (ppm)
6.85 6.84 6.83 5.17 5.16 5.15 3.52 3.50 3.49 2.98 2.96 2.95 2.28 2.27 2.25 2.23 2.22 2.20 2.19 2.18 2.18 2.17 2.17 2.16 2.15 2.15 2.14 2.13 2.12 2.11 2.10 2.09 2.08 2.01 2.00 2.00 1.98 1.97 1.97 1.95 1.93 1.92 1.91 1.91 1.90 1.89 1.89 1.88 1.87 1.87 1.86 1.83 1.82 1.81 1.81 1.79 1.78 1.76 1.73 1.73 1.72 1.72 1.71 1.71 1.70 1.69 1.67 1.66 1.65 1.63 1.62 1.62 1.61 1.61 1.60 1.58 1.56 1.55 1.53 1.53 1.52 1.51
0.06 0.94 2.00 2.00 1.01 2.06 0.97 2.02 1.05 2.07 0.99

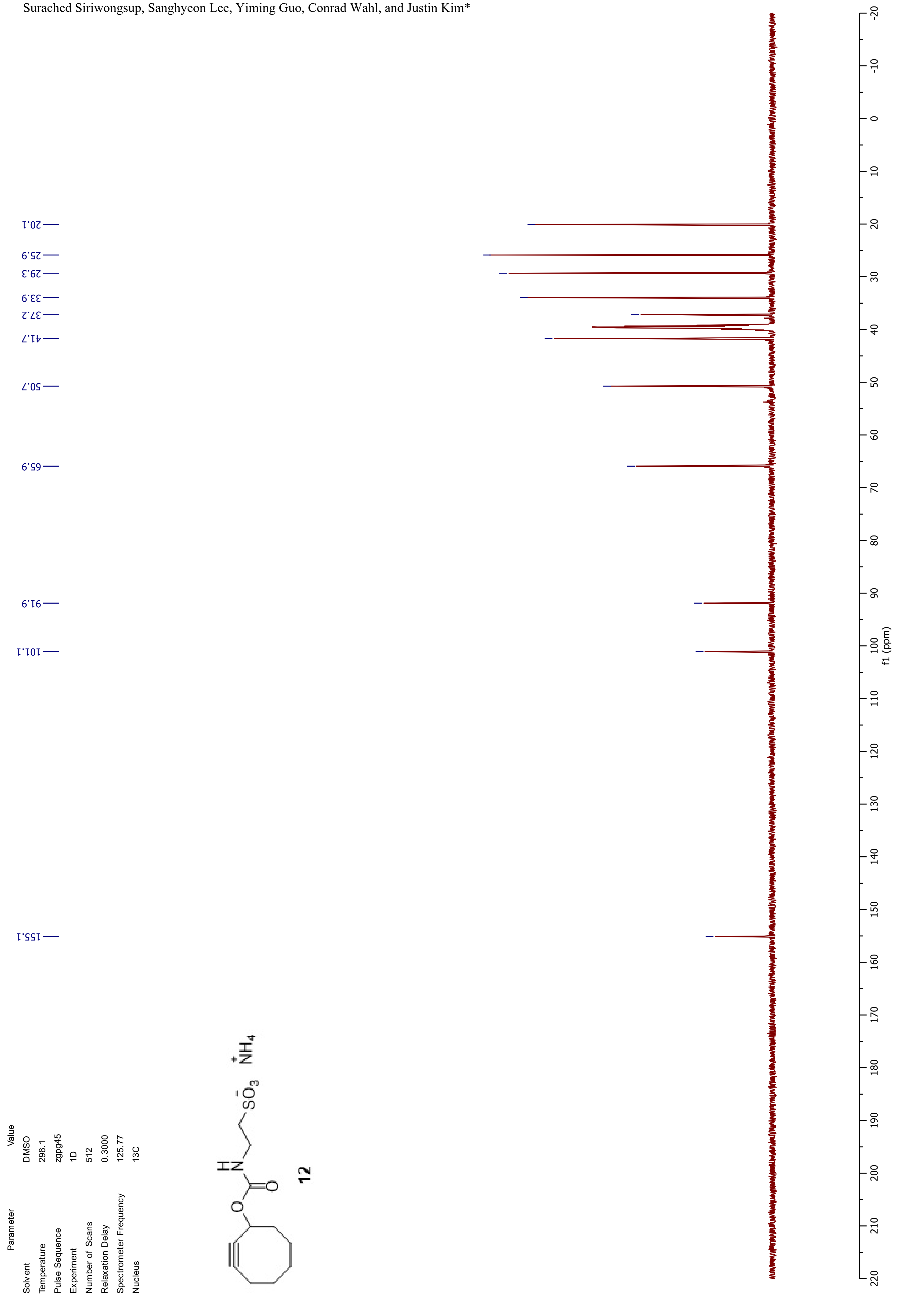
20.1
25.9
29.3
33.9
37.2
41.7
50.7
65.9
91.9
101.1
155.1
f1 (ppm)
Parameter
Value
Solvent
DMSO
Temperature
298.1
Pulse Sequence
zgpg45
Experiment
1D
Number of Scans
512
Relaxation Delay
0.3000
Spectrometer Frequency
125.77
Nucleus
13C
12

Parameter	Value
Solvent	MeOD
Temperature	298.2
Pulse Sequence	zg45
Experiment	1D
Number of Scans	16
Relaxation Delay	1.0000
Spectrometer Frequency	500.13
Nucleus	1H

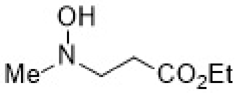

14

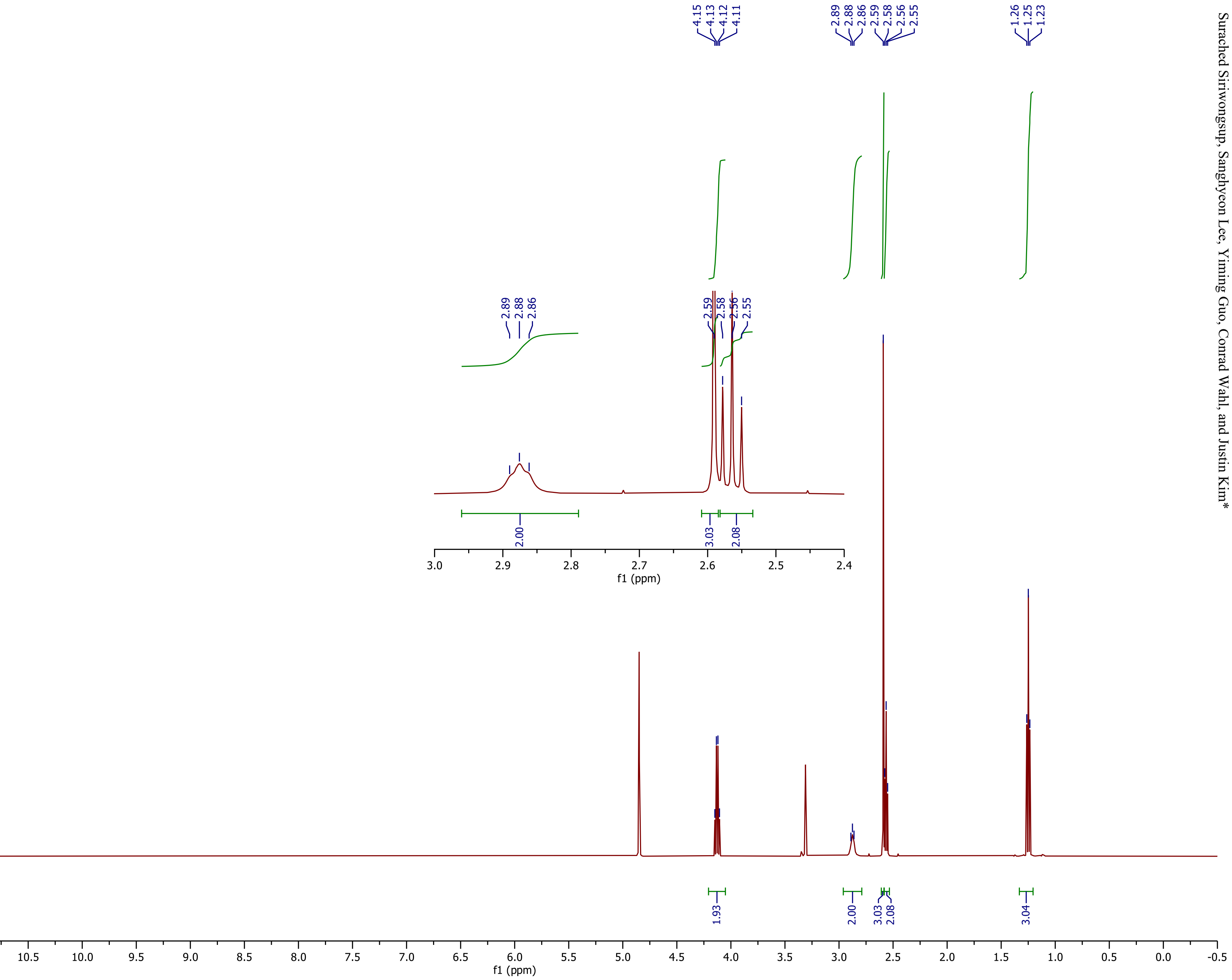

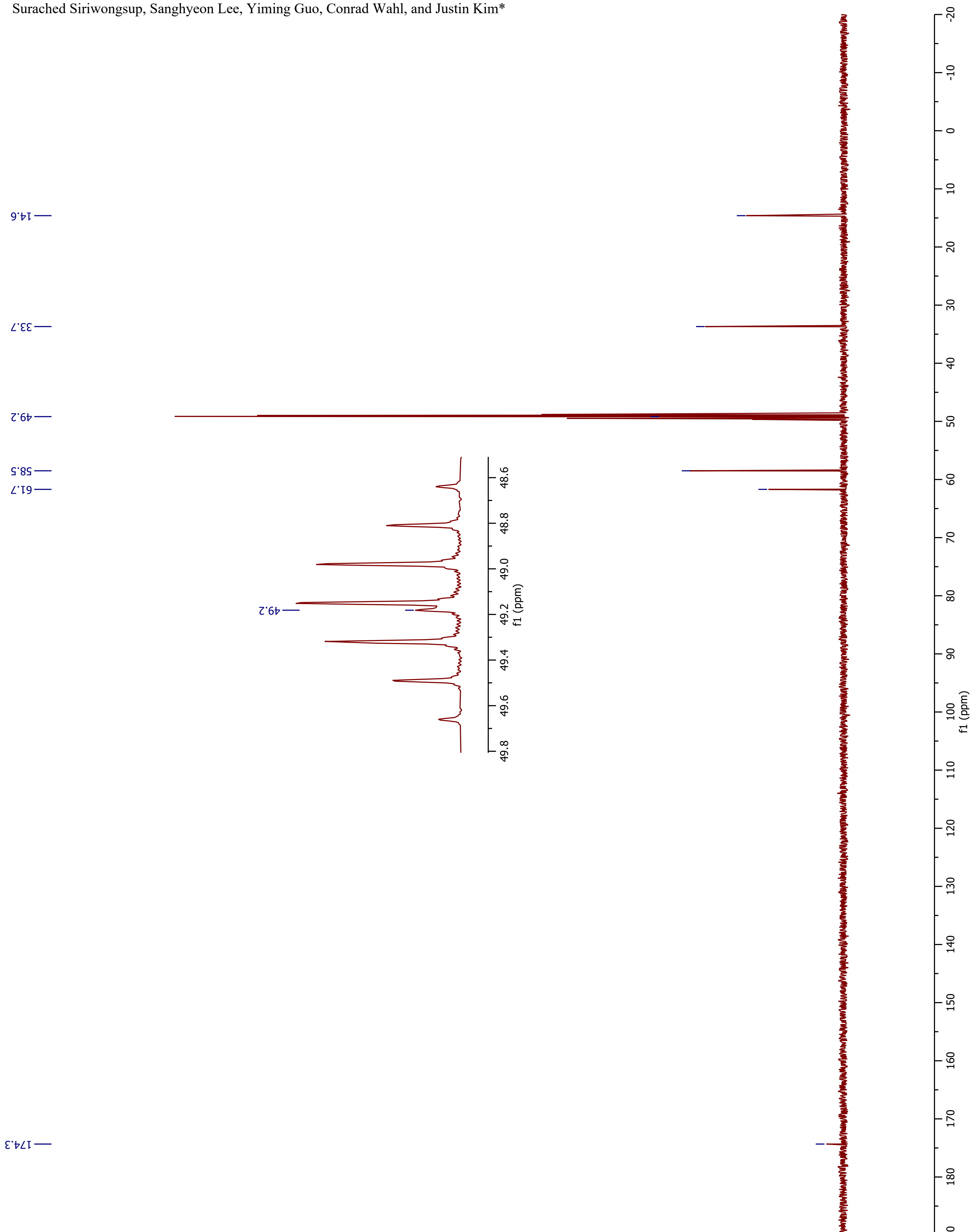

Parameter	Value
Solvent	MeOD
Temperature	298.1
Pulse Sequence	zgpg45
Experiment	1D
Number of Scans	512
Relaxation Delay	0.3000
Spectrometer Frequency	125.77
Nucleus	13C

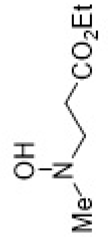

14

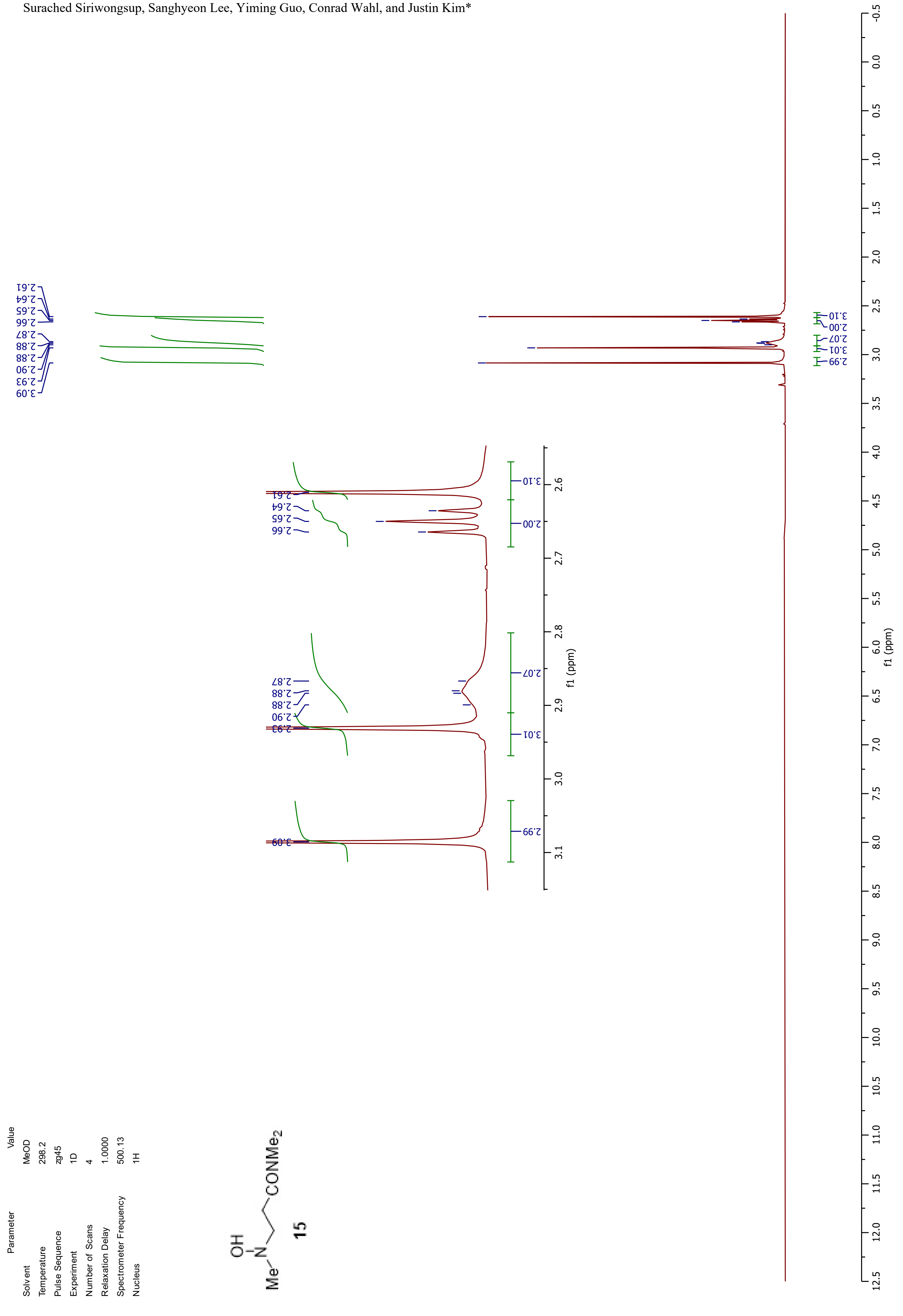
Parameter Value
Solvent MeOD
Temperature 298.2
Pulse Sequence zg45
Experiment 1D
Number of Scans 4
Relaxation Delay 1.0000
Spectrometer Frequency 500.13
Nucleus 1H
Me–N(OH)–CH2CH2–CONMe2
15
3.09
2.93
2.90
2.88
2.88
2.87
2.66
2.65
2.64
2.61
3.10
2.00
2.07
3.01
2.99
f1 (ppm)

Parameter	Value
Solvent	MeOD
Temperature	298.2
Pulse Sequence	zgpg45
Experiment	1D
Number of Scans	64
Relaxation Delay	0.3000
Spectrometer Frequency	125.77
Nucleus	13C

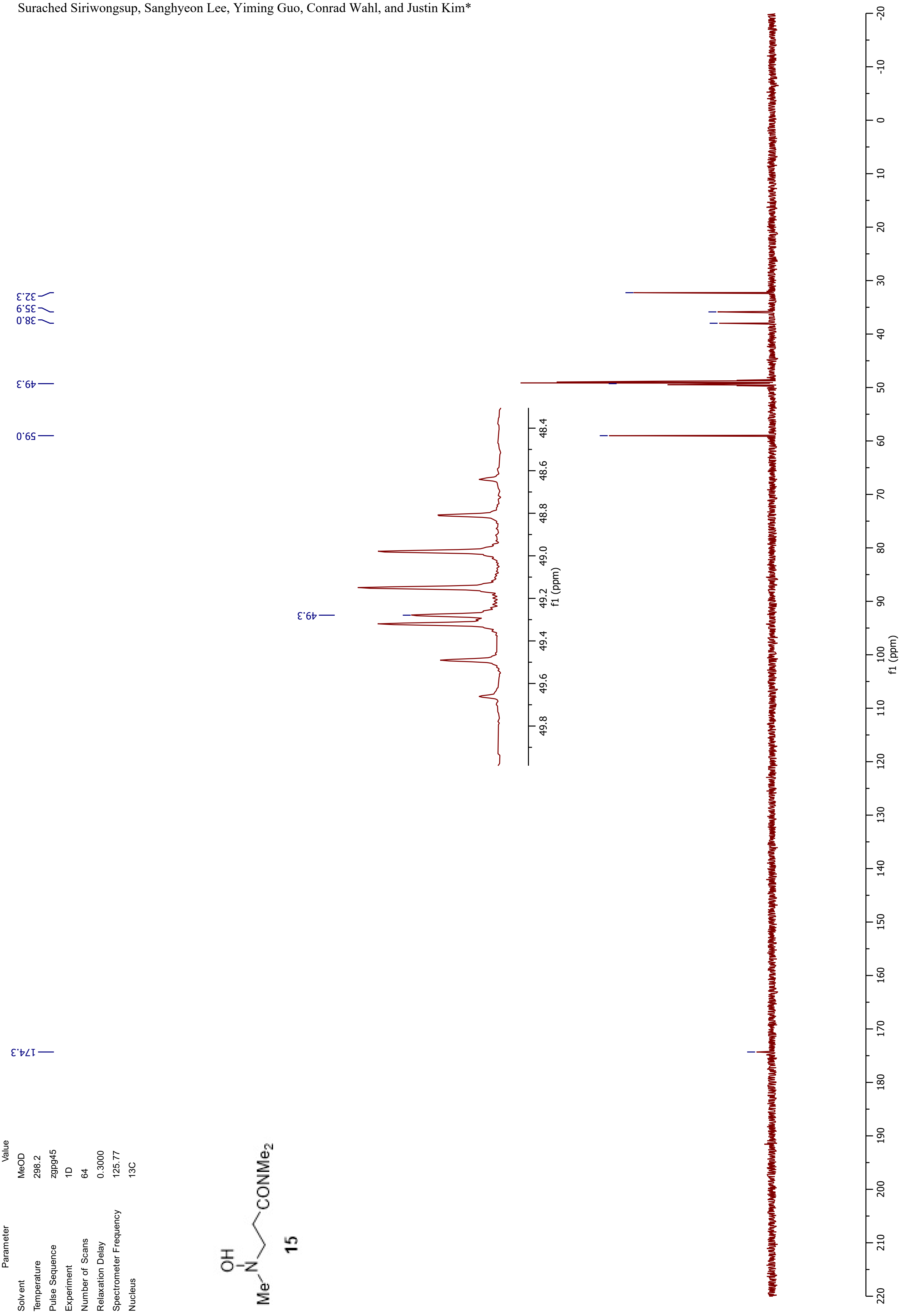

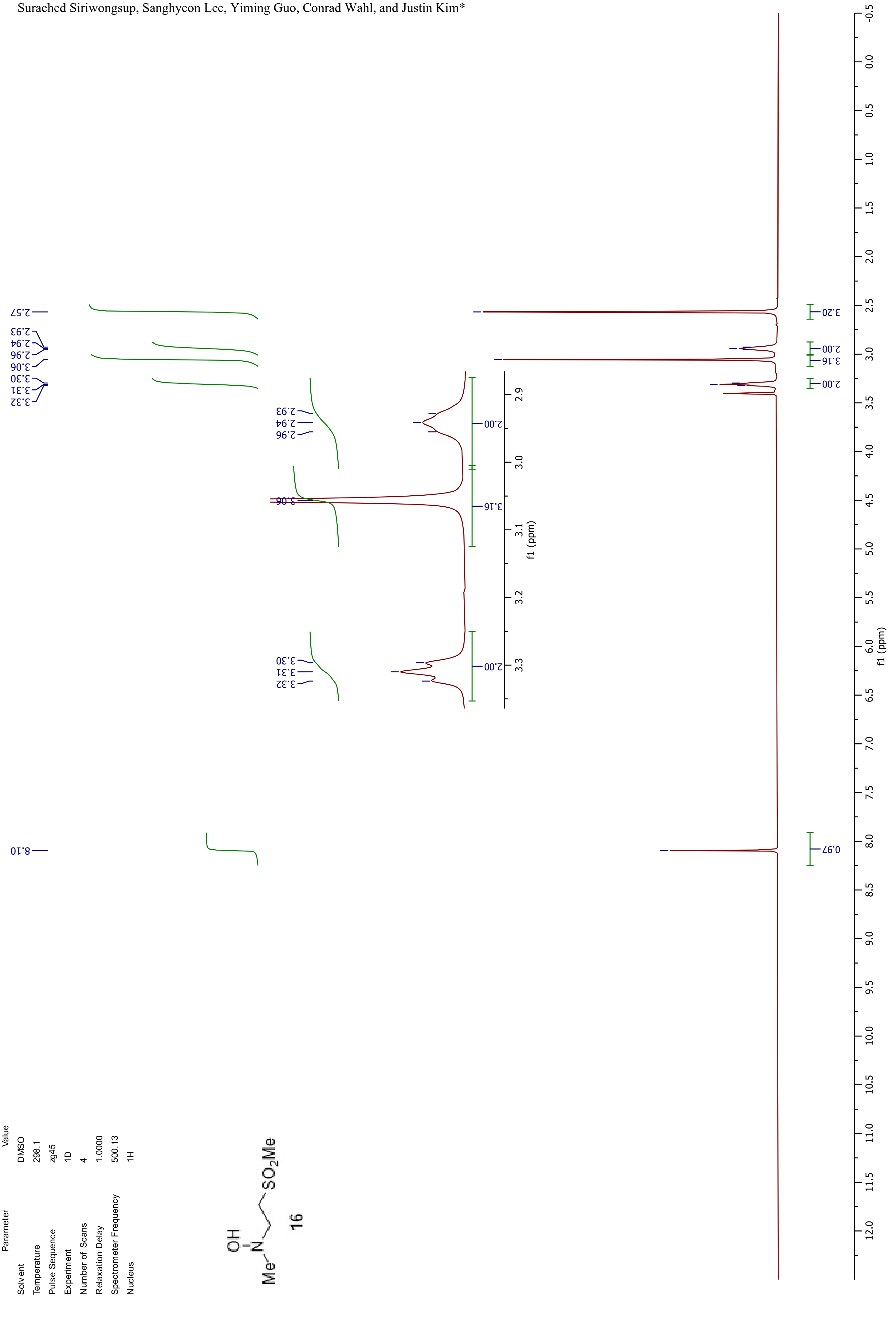
Parameter Value
Solvent DMSO
Temperature 298.1
Pulse Sequence zg45
Experiment 1D
Number of Scans 4
Relaxation Delay 1.0000
Spectrometer Frequency 500.13
Nucleus 1H
16
2.57
2.93
2.94
2.96
3.06
3.30
3.31
3.32
8.10
3.20
2.00
3.16
2.00
0.97
f1 (ppm)

Parameter	Value
Solvent	DMSO
Temperature	298.2
Pulse Sequence	zgpg45
Experiment	1D
Number of Scans	256
Relaxation Delay	0.3000
Spectrometer Frequency	125.77
Nucleus	13C

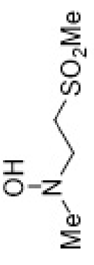

16

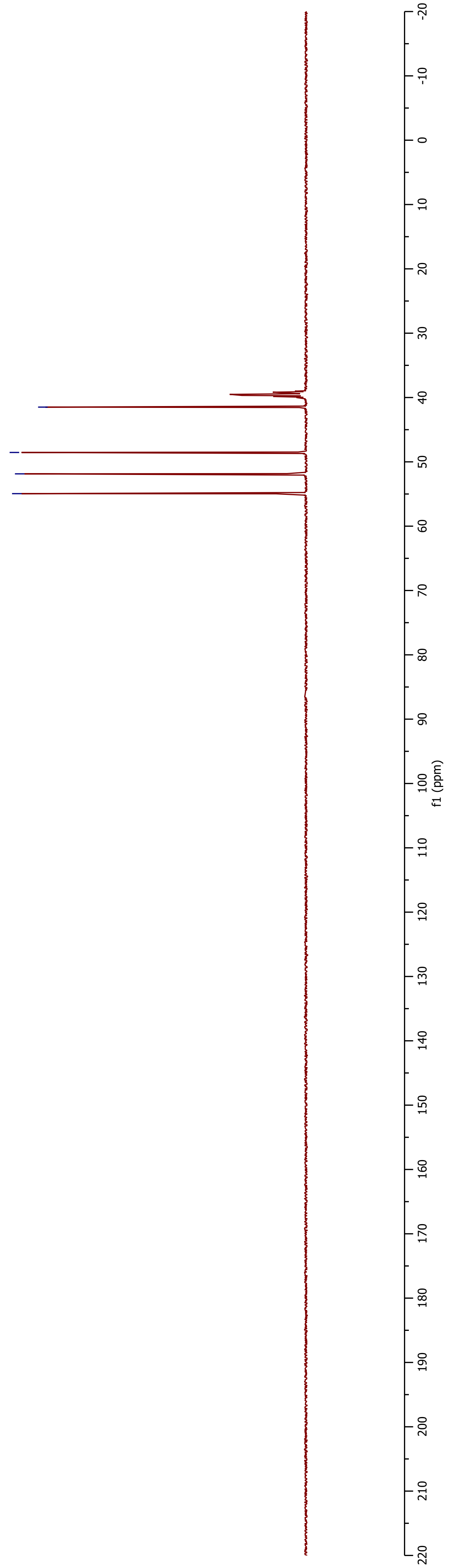

Parameter	Value
Solvent	MeOD
Temperature	298.1
Pulse Sequence	zg45
Experiment	1D
Number of Scans	4
Relaxation Delay	1.0000
Spectrometer Frequency	500.13
Nucleus	1H

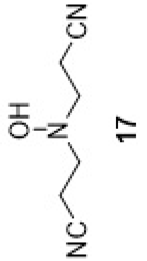

17

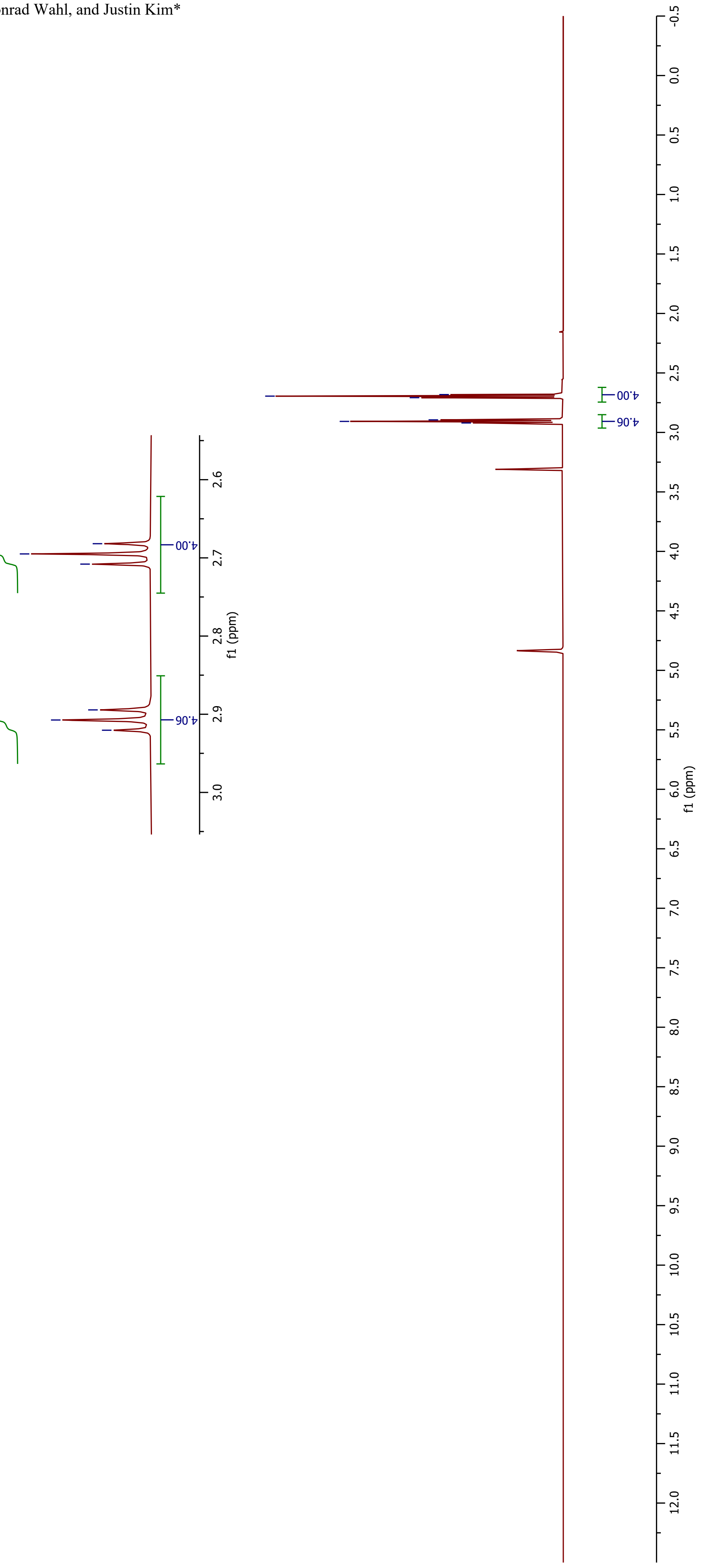

Parameter	Value
Solvent	MeOD
Temperature	298.2
Pulse Sequence	zgpg45
Experiment	1D
Number of Scans	64
Relaxation Delay	0.3000
Spectrometer Frequency	125.77
Nucleus	13C

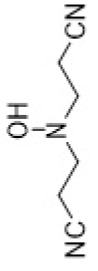

17

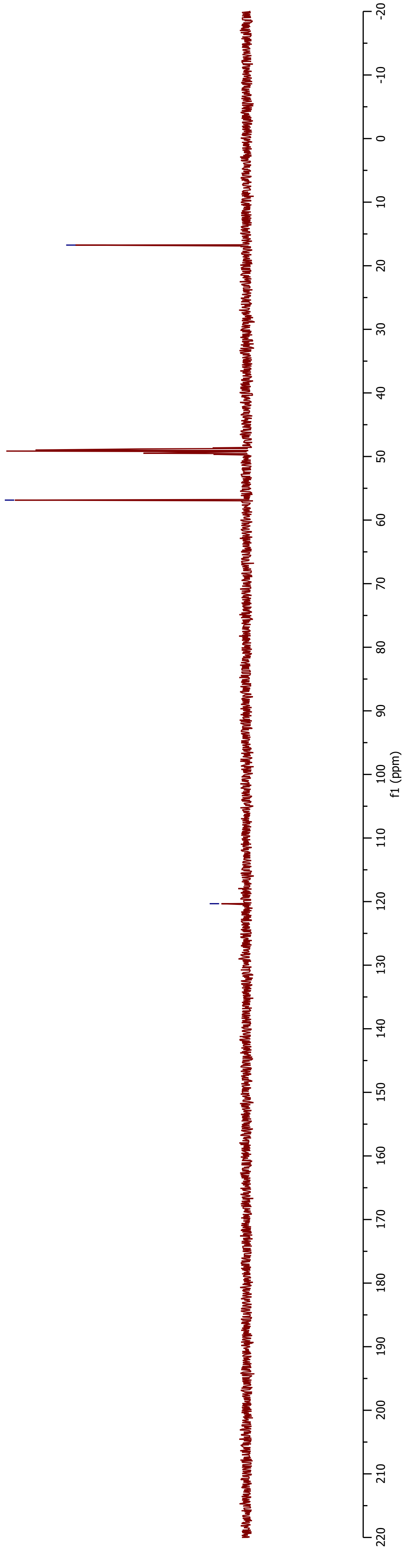

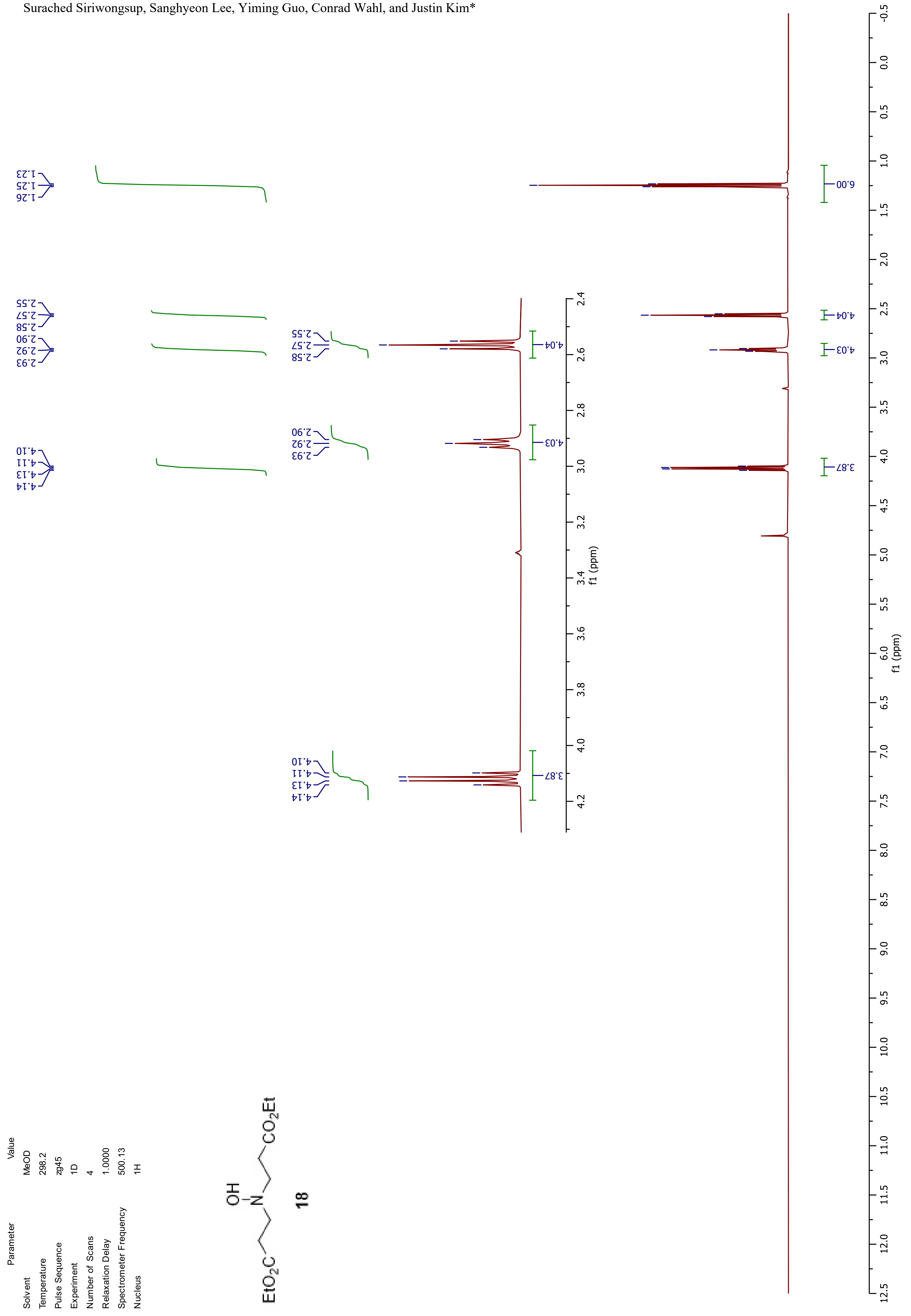
Parameter
Value
Solvent
MeOD
Temperature
298.2
Pulse Sequence
zg45
Experiment
1D
Number of Scans
4
Relaxation Delay
1.0000
Spectrometer Frequency
500.13
Nucleus
1H
EtO2C
OH
N
CO2Et
18
1.23
1.25
1.26
2.55
2.57
2.58
2.90
2.92
2.93
4.10
4.11
4.13
4.14
6.00
4.04
4.03
3.87
f1 (ppm)

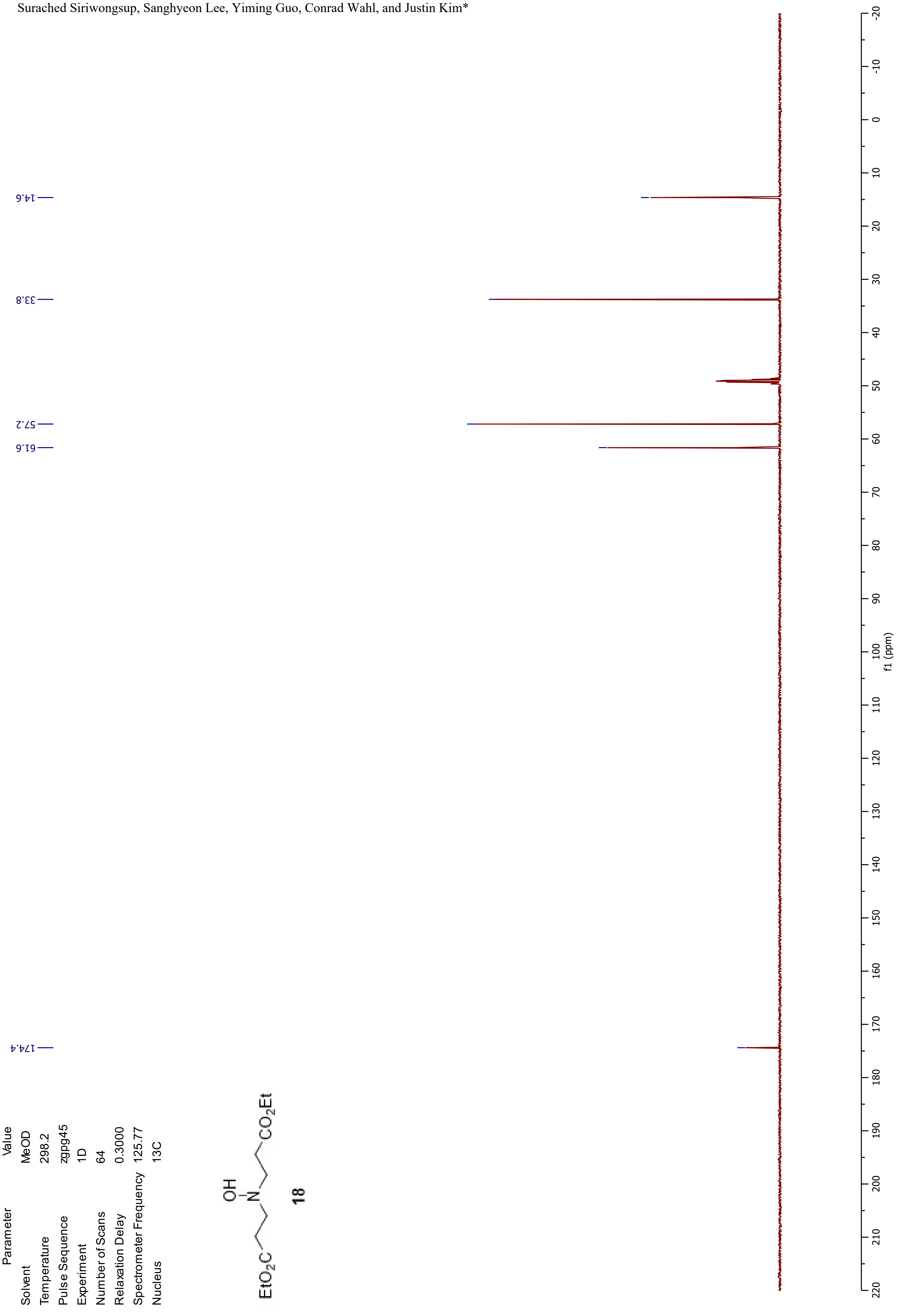
14.6
33.8
57.2
61.6
174.4
f1 (ppm)
Parameter Value
Solvent MeOD
Temperature 298.2
Pulse Sequence zgpg45
Experiment 1D
Number of Scans 64
Relaxation Delay 0.3000
Spectrometer Frequency 125.77
Nucleus 13C
OH
N
EtO_2C
CO_2Et
18

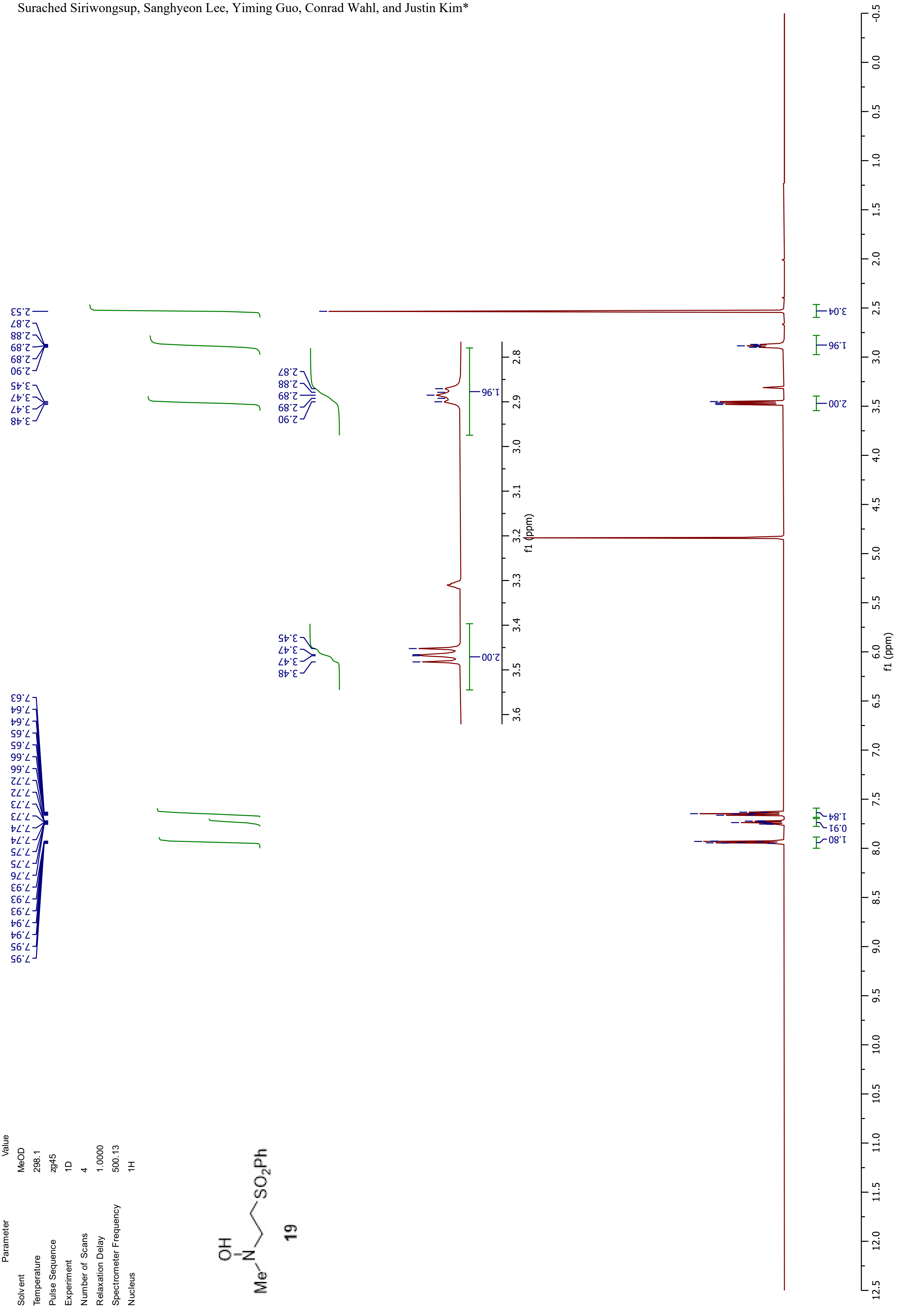
Parameter Value
Solvent MeOD
Temperature 298.1
Pulse Sequence zg45
Experiment 1D
Number of Scans 4
Relaxation Delay 1.0000
Spectrometer Frequency 500.13
Nucleus 1H
19
f1 (ppm)
2.53
2.87
2.88
2.89
2.89
2.90
3.45
3.47
3.47
3.48
7.63
7.64
7.64
7.65
7.65
7.66
7.66
7.72
7.72
7.73
7.73
7.74
7.74
7.75
7.75
7.76
7.93
7.93
7.93
7.94
7.94
7.95
7.95
3.04
1.96
2.00
1.84
0.91
1.80

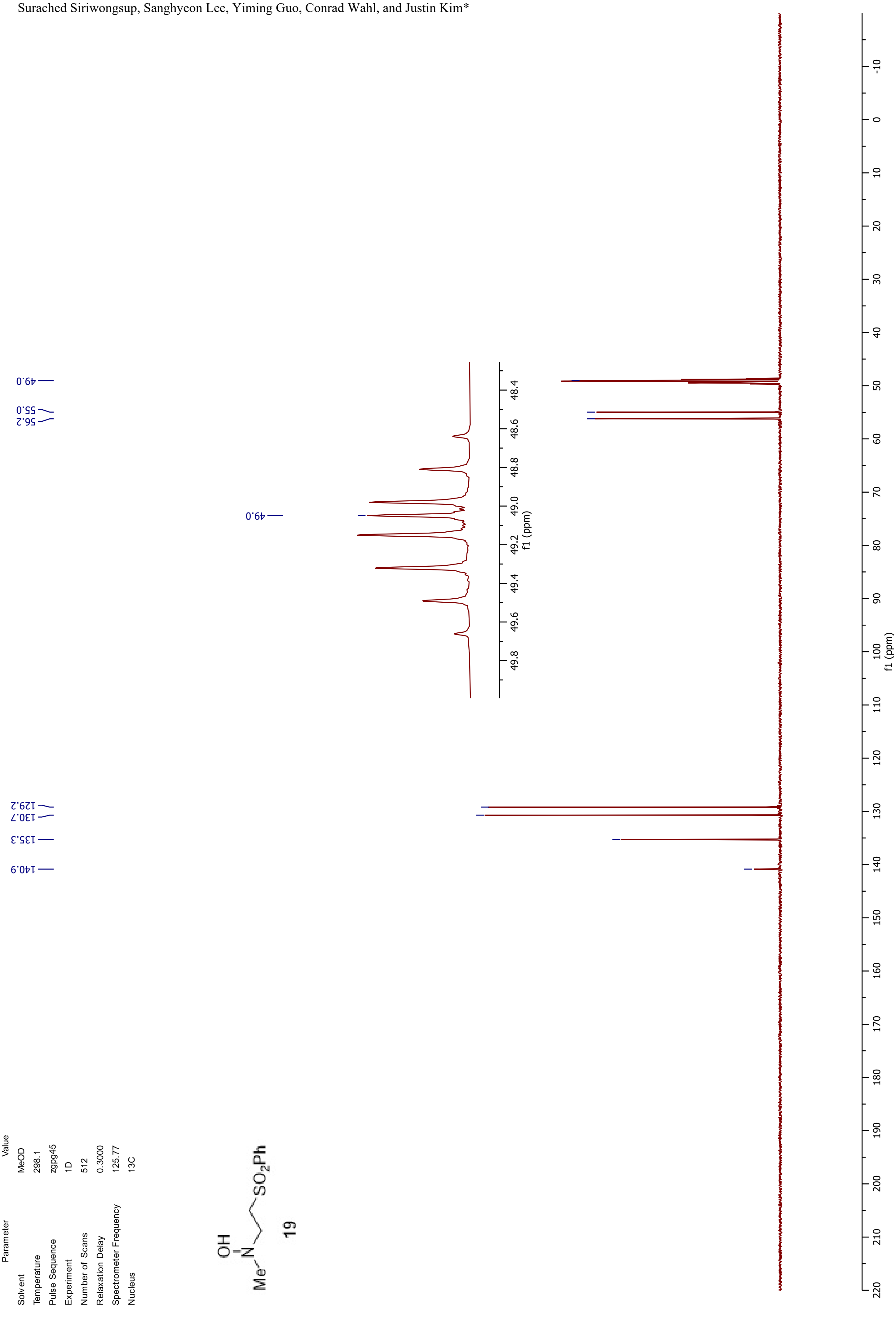
Parameter Value
Solvent MeOD
Temperature 298.1
Pulse Sequence zgpg45
Experiment 1D
Number of Scans 512
Relaxation Delay 0.3000
Spectrometer Frequency 125.77
Nucleus 13C
19
49.0
55.0
56.2
129.2
130.7
135.3
140.9
f1 (ppm)

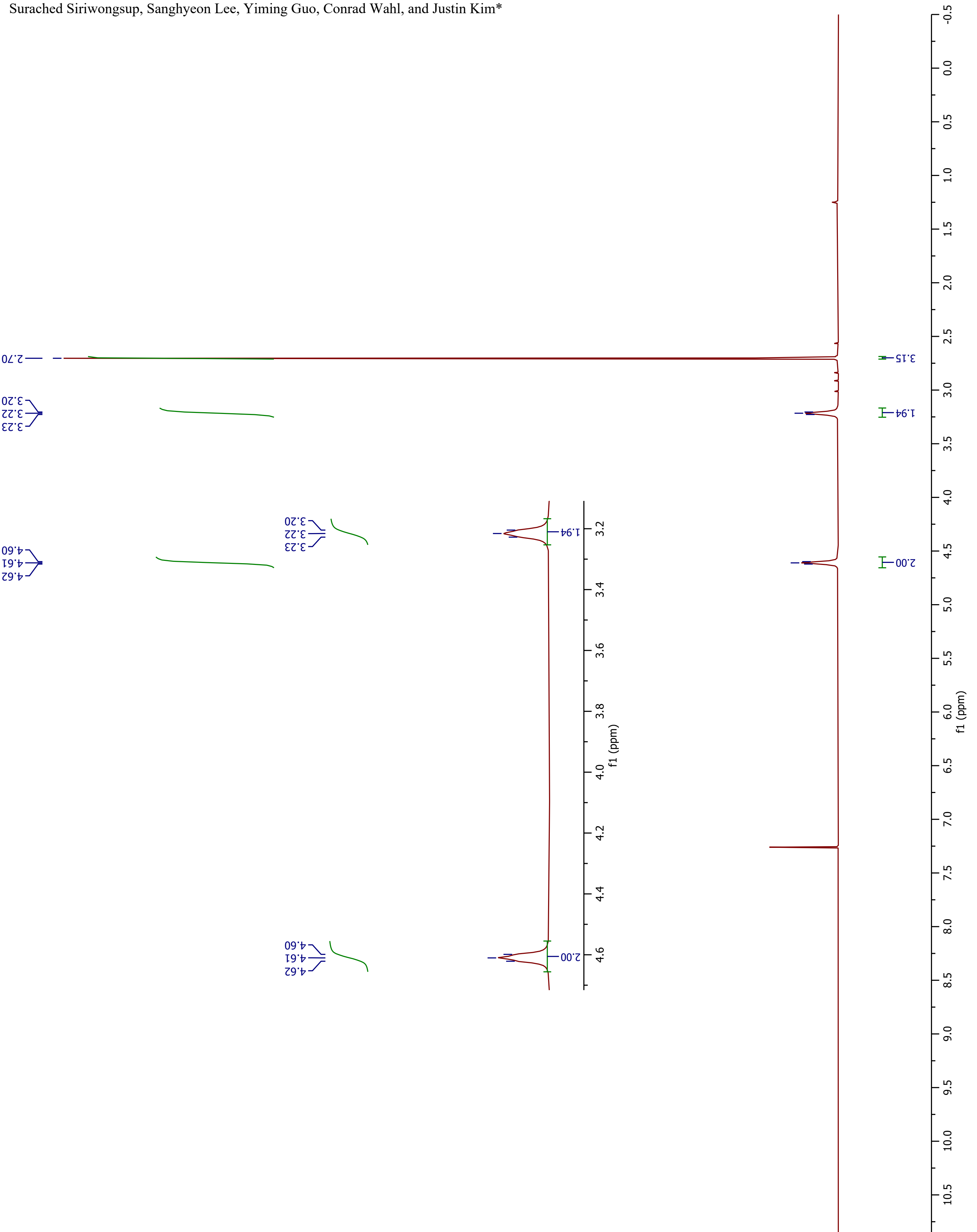

Parameter	Value
Solvent	CDCl3
Temperature	298.2
Pulse Sequence	zg30
Experiment	1D
Number of Scans	16
Relaxation Delay	1.0000
Spectrometer Frequency	500.27
Nucleus	1H

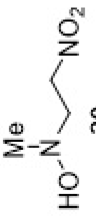

20

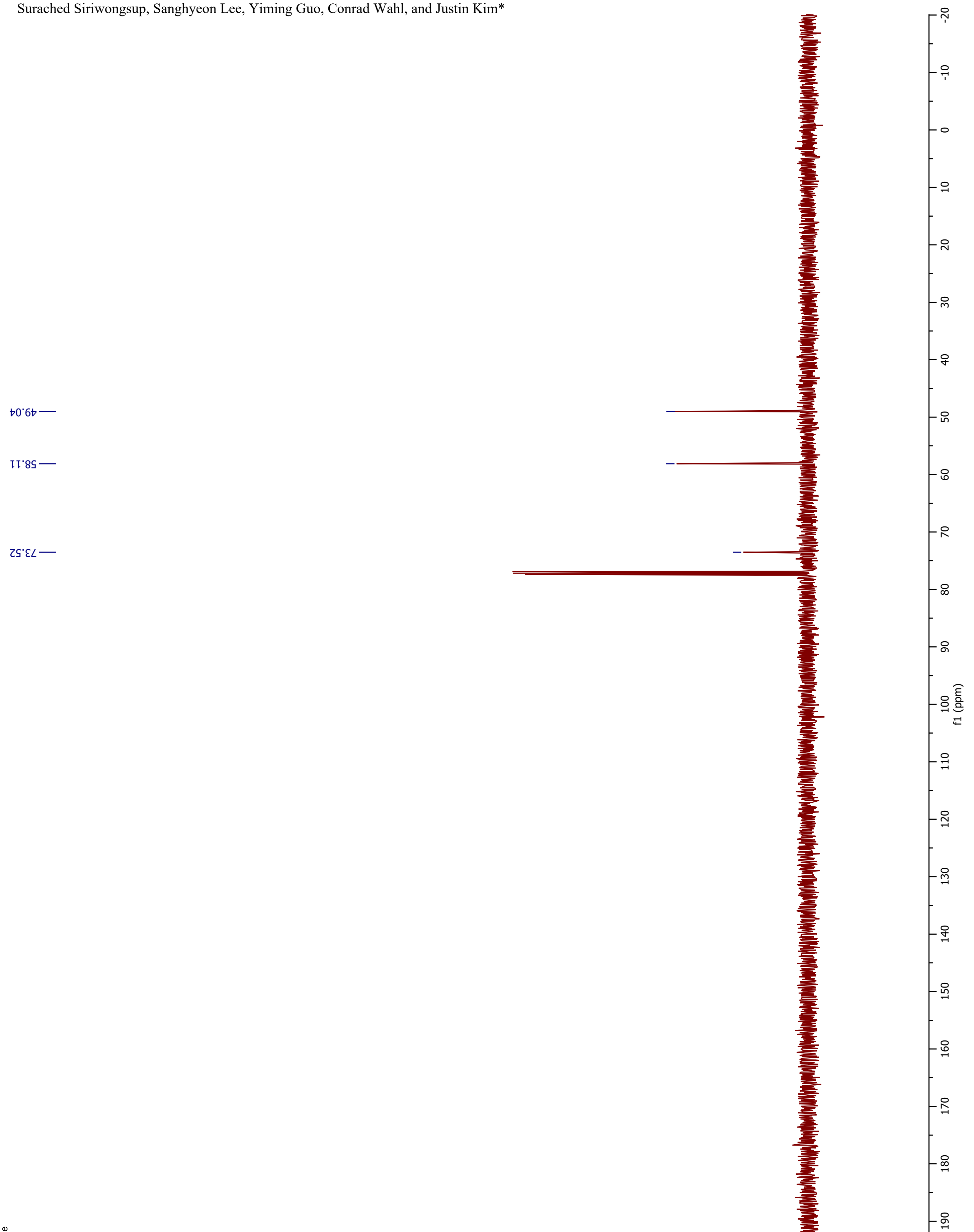

Parameter	Value
Solvent	MeOD
Temperature	298.2
Pulse Sequence	zg45
Experiment	1D
Number of Scans	4
Relaxation Delay	1.0000
Spectrometer Frequency	500.13
Nucleus	1H

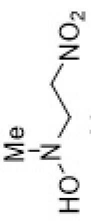

20

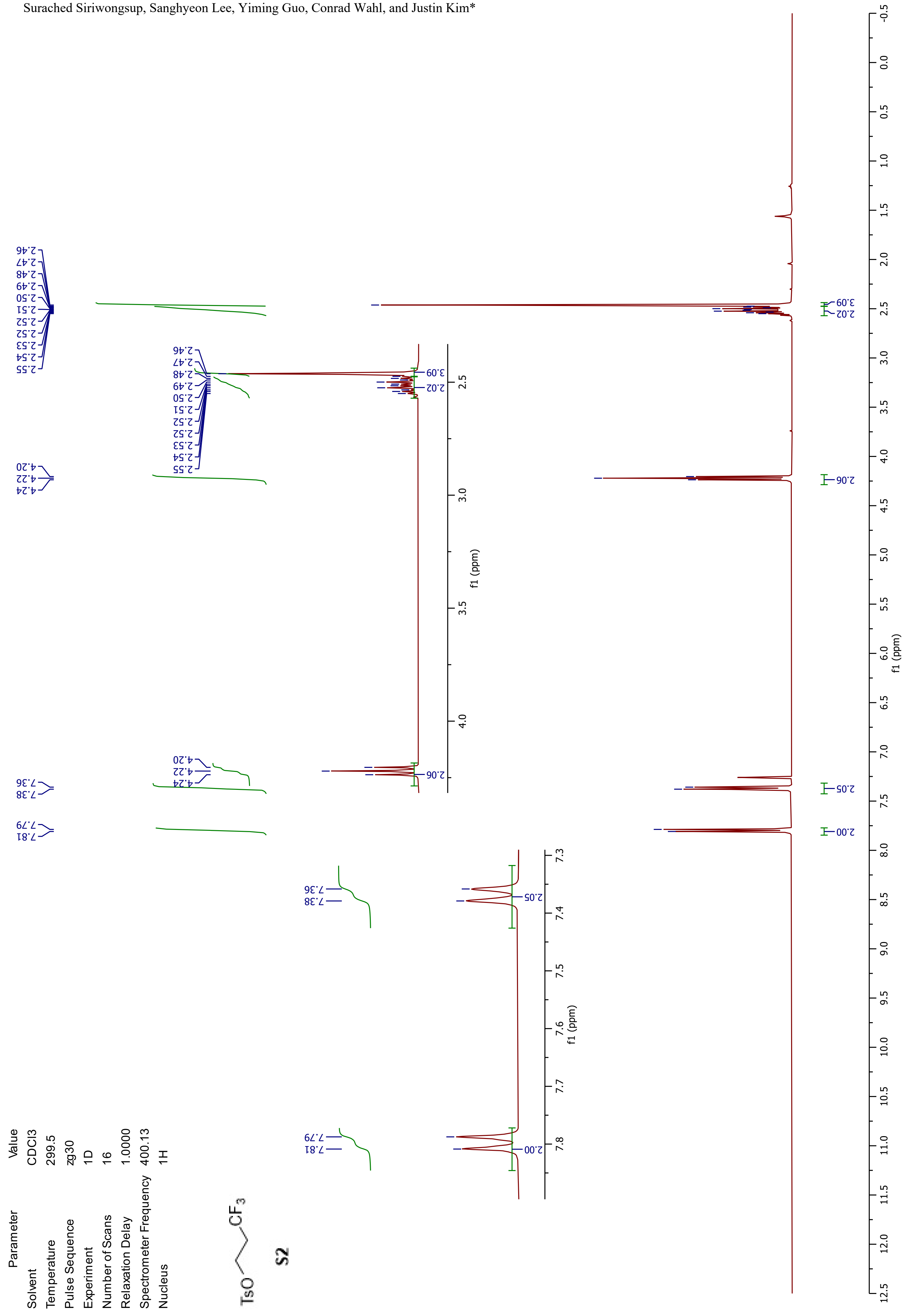
Parameter Value
Solvent CDCl3
Temperature 299.5
Pulse Sequence zg30
Experiment 1D
Number of Scans 16
Relaxation Delay 1.0000
Spectrometer Frequency 400.13
Nucleus 1H
TsO CF3
S2
f1 (ppm)

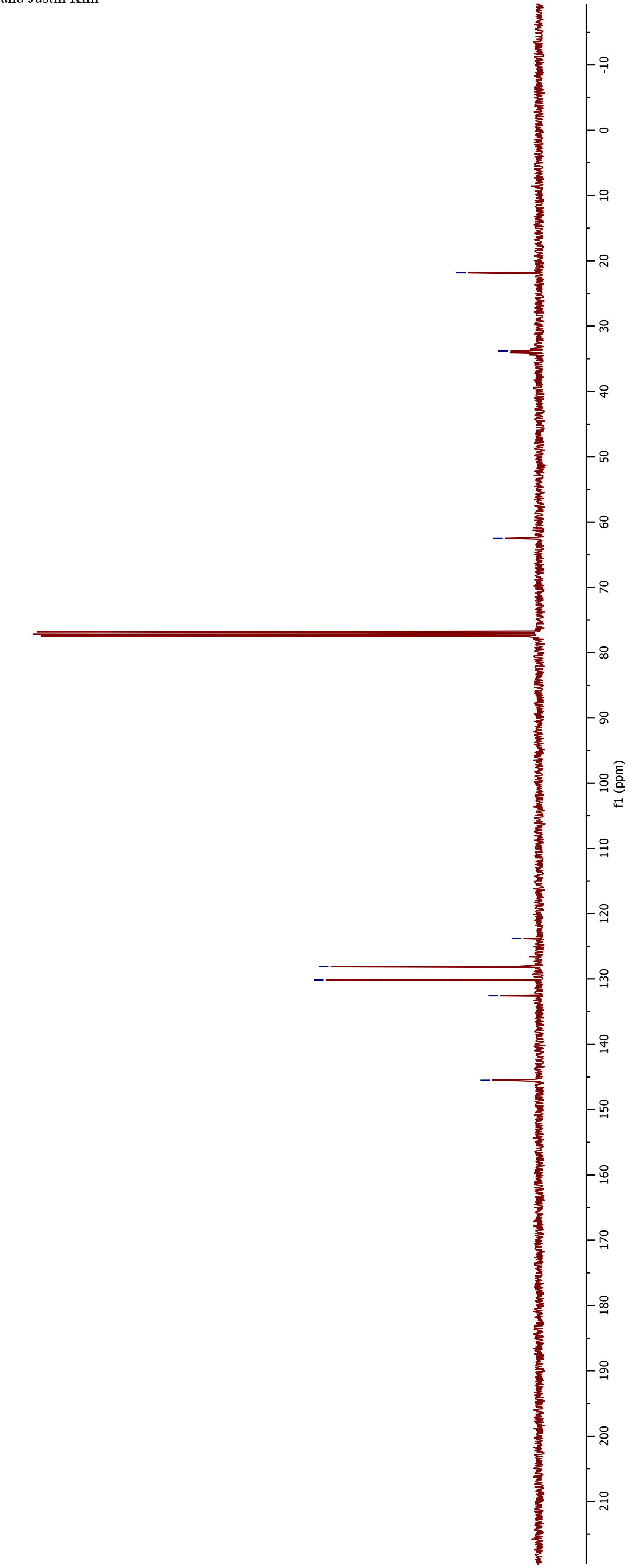

Parameter	Value
Solvent	CDCl3
Temperature	299.9
Pulse Sequence	zgpg30
Experiment	1D
Number of Scans	256
Relaxation Delay	2.0000
Spectrometer Frequency	100.62
Nucleus	13C

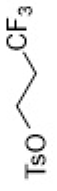

S2

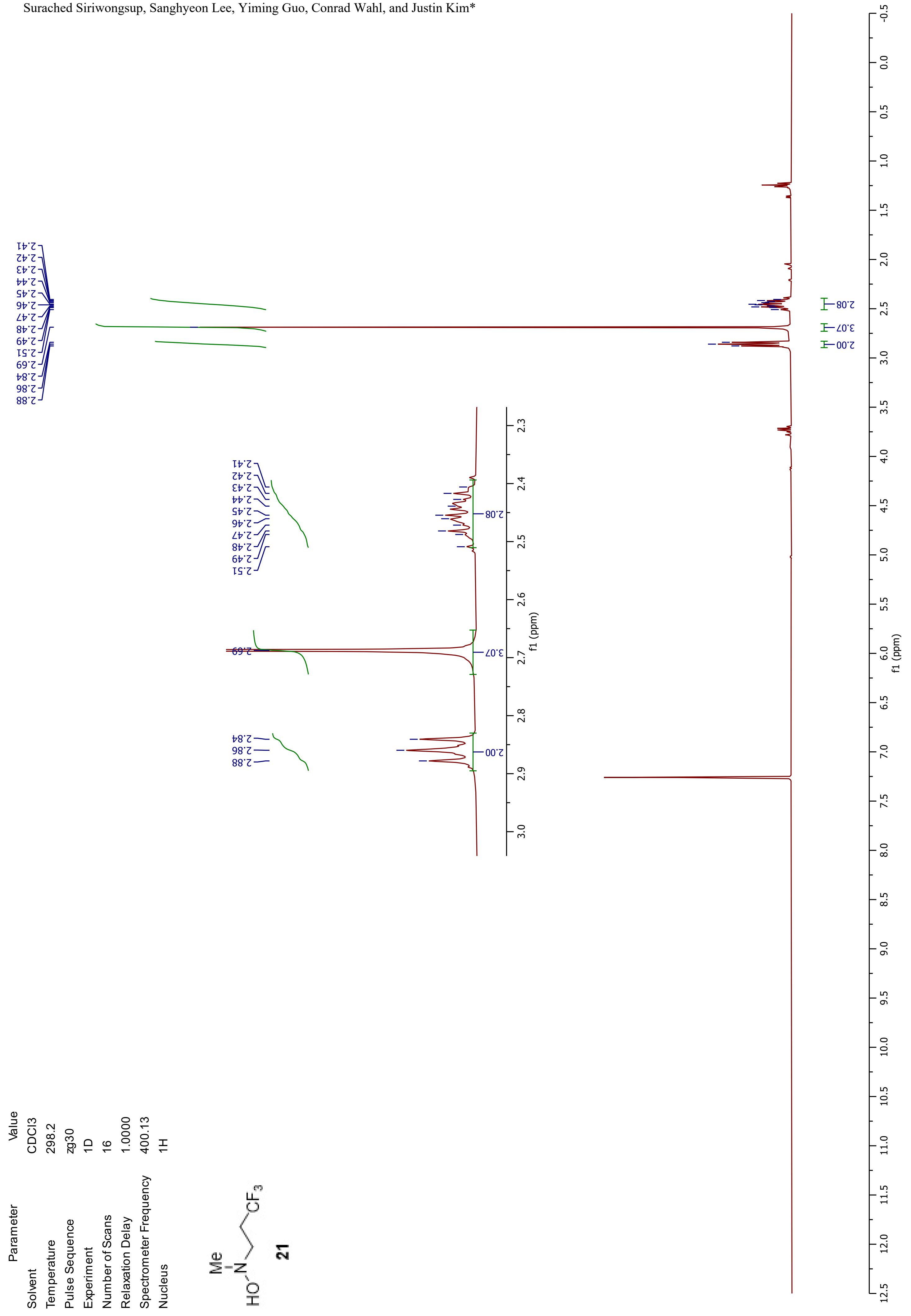
Parameter
Value
Solvent
CDCl3
Temperature
298.2
Pulse Sequence
zg30
Experiment
1D
Number of Scans
16
Relaxation Delay
1.0000
Spectrometer Frequency
400.13
Nucleus
1H
21
f1 (ppm)
2.41
2.42
2.43
2.44
2.45
2.46
2.47
2.48
2.49
2.51
2.69
2.84
2.86
2.88
2.08
3.07
2.00

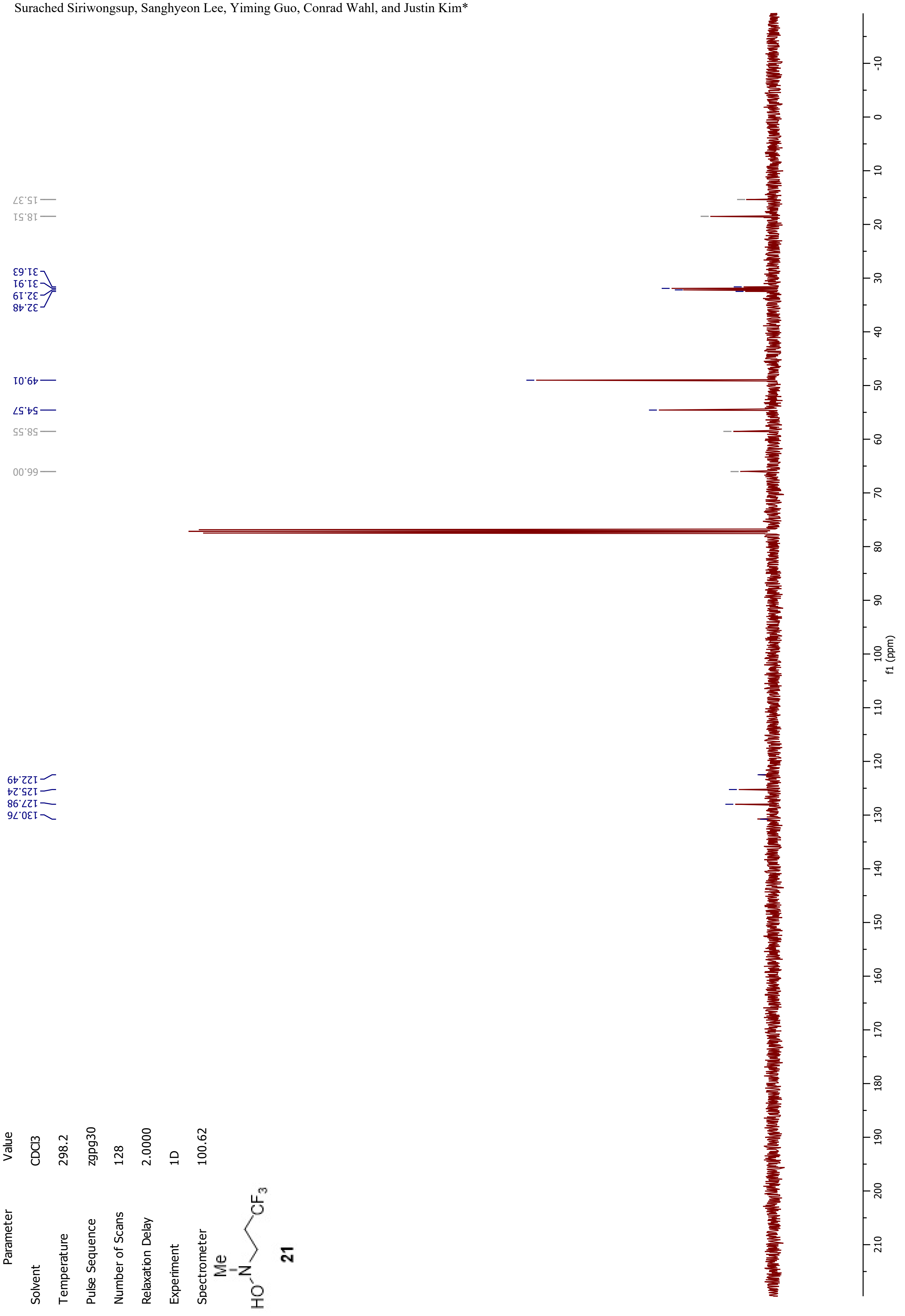
15.37
18.51
31.63
31.91
32.19
32.48
49.01
54.57
58.55
66.00
122.49
125.24
127.98
130.76
Parameter
Value
Solvent
CDCl3
Temperature
298.2
Pulse Sequence
zgpg30
Number of Scans
128
Relaxation Delay
2.0000
Experiment
1D
Spectrometer
100.62
Me
HO
N
CF3
21
f1 (ppm)

Parameter	Value
Solvent	MeOD
Temperature	298.1
Pulse Sequence	zg45
Experiment	1D
Number of Scans	4
Relaxation Delay	1.0000
Spectrometer Frequency	500.13
Nucleus	1H

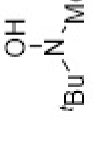

22

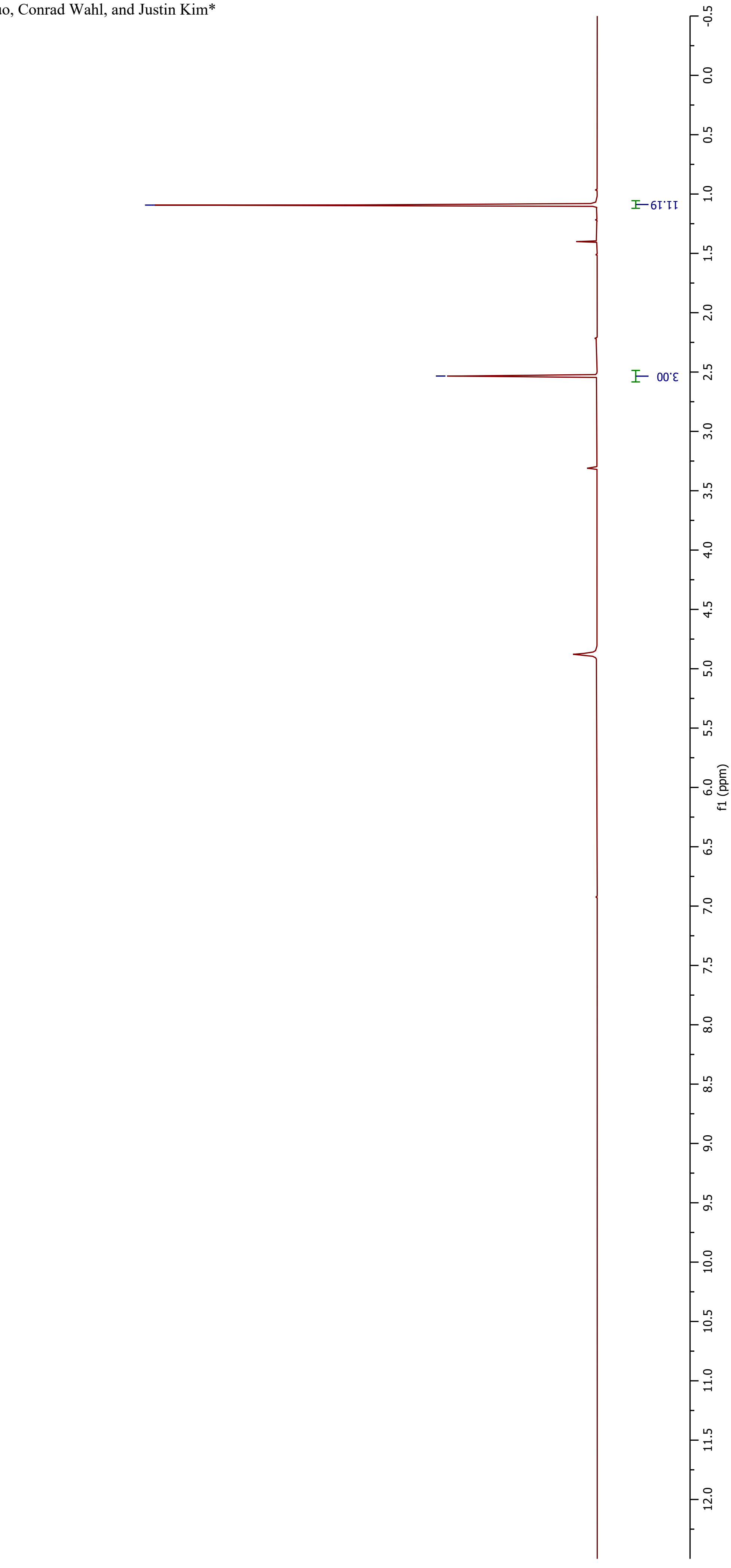

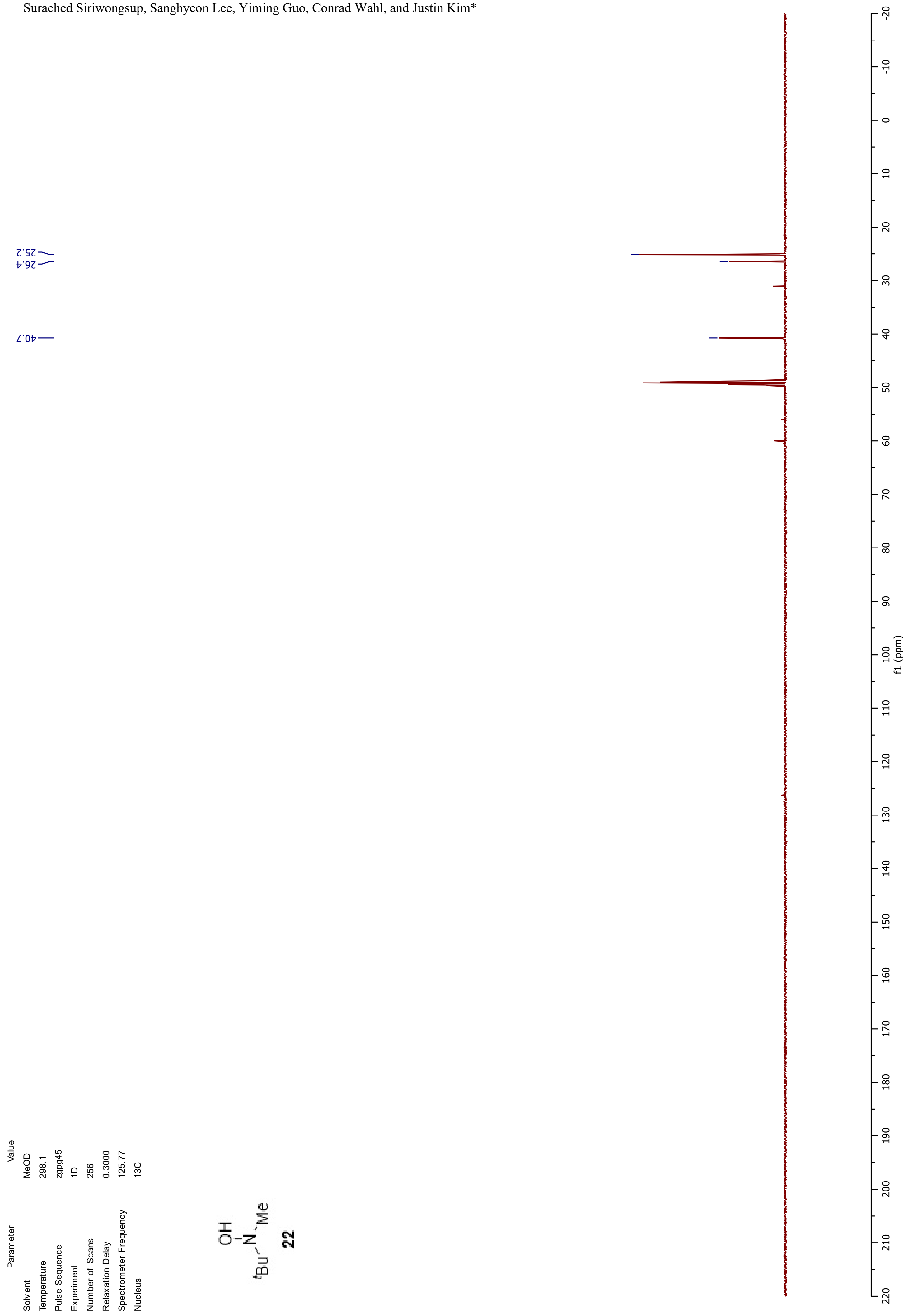
25.2
26.4
40.7
Parameter
Value
Solvent
MeOD
Temperature
298.1
Pulse Sequence
zgpg45
Experiment
1D
Number of Scans
256
Relaxation Delay
0.3000
Spectrometer Frequency
125.77
Nucleus
13C
OH
N
tBu
Me
22
f1 (ppm)

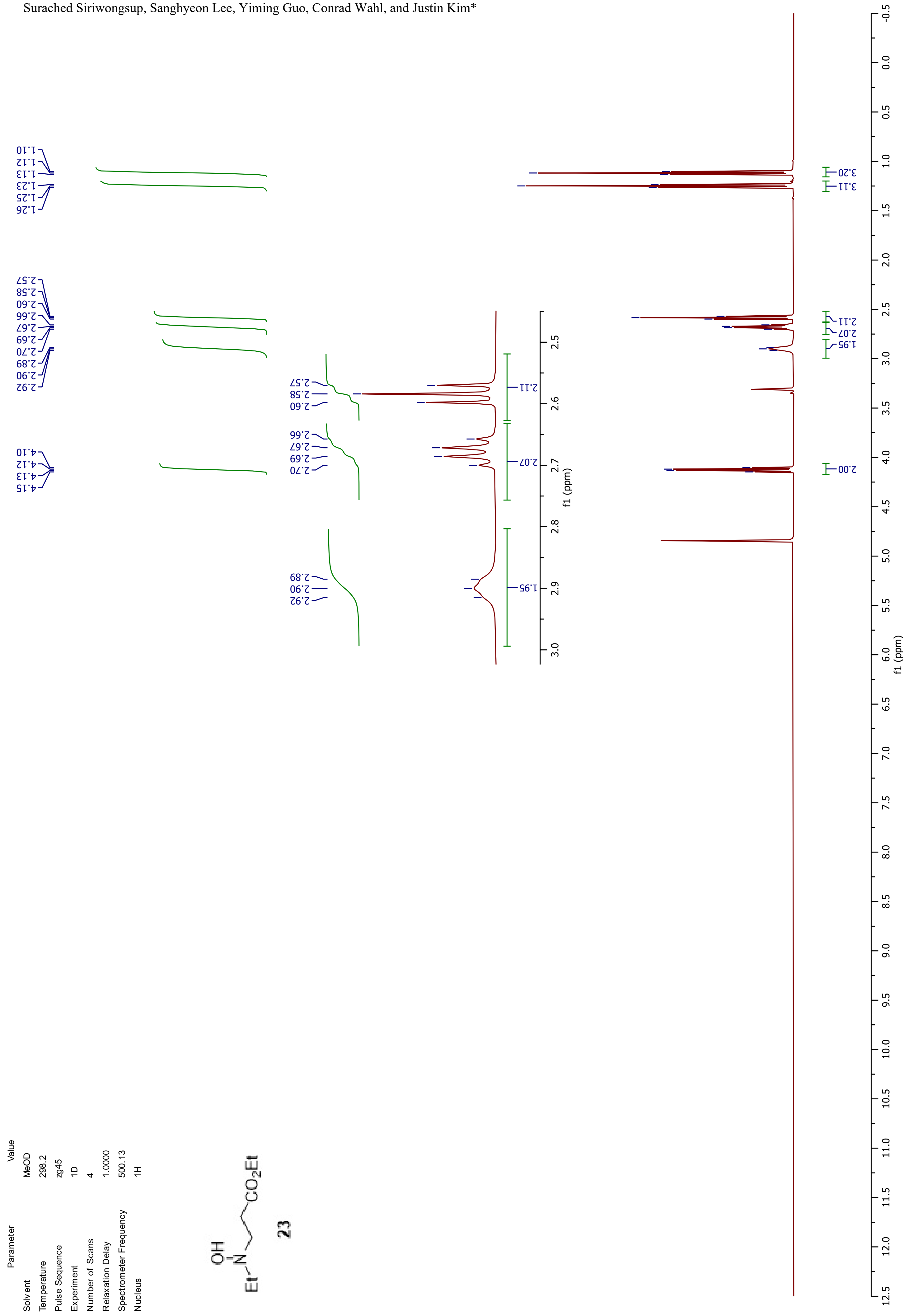
Parameter Value
Solvent MeOD
Temperature 298.2
Pulse Sequence zg45
Experiment 1D
Number of Scans 4
Relaxation Delay 1.0000
Spectrometer Frequency 500.13
Nucleus 1H
23
f1 (ppm)

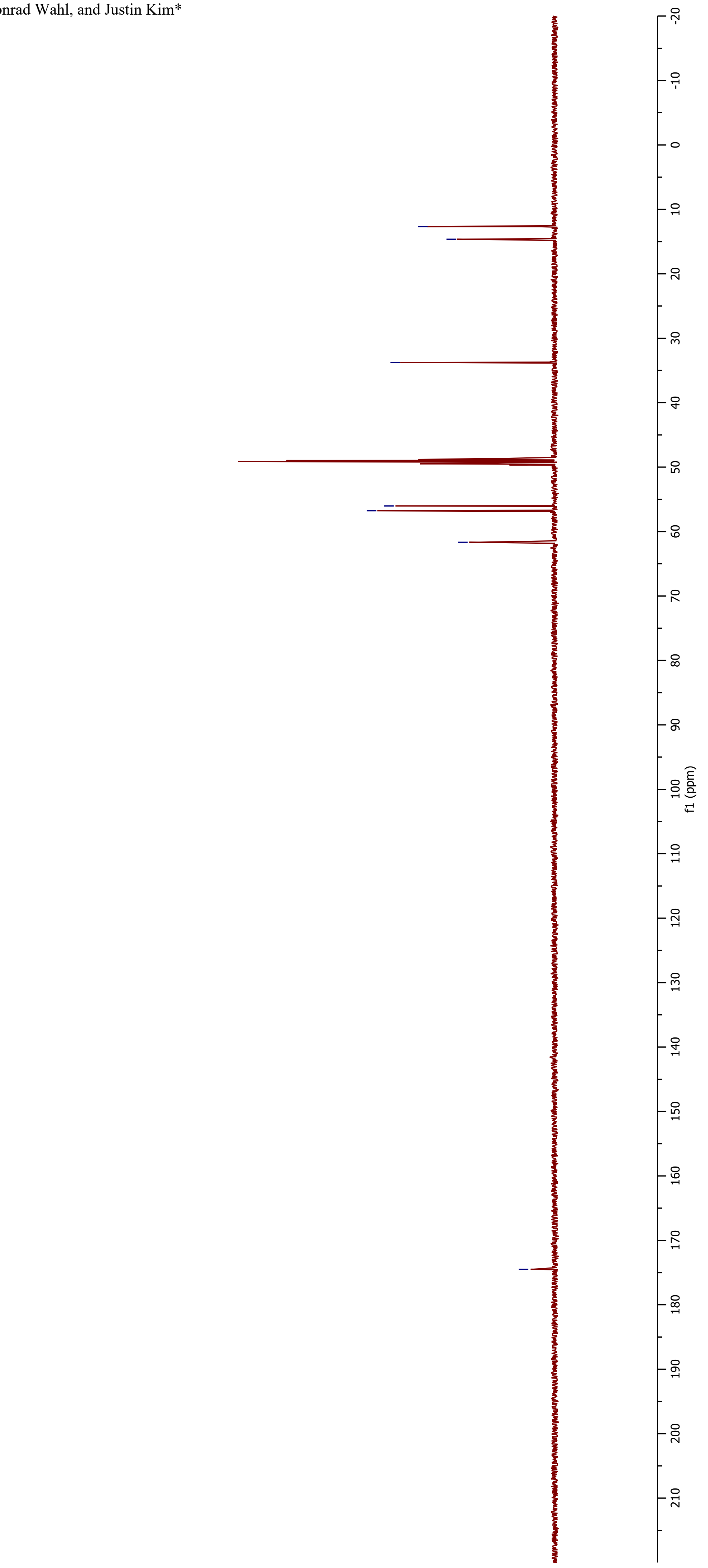

Parameter	Value
Solvent	MeOD
Temperature	298.1
Pulse Sequence	zgpg45
Experiment	1D
Number of Scans	256
Relaxation Delay	0.3000
Spectrometer Frequency	125.77
Nucleus	13C

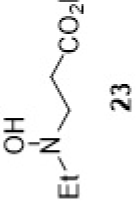

23

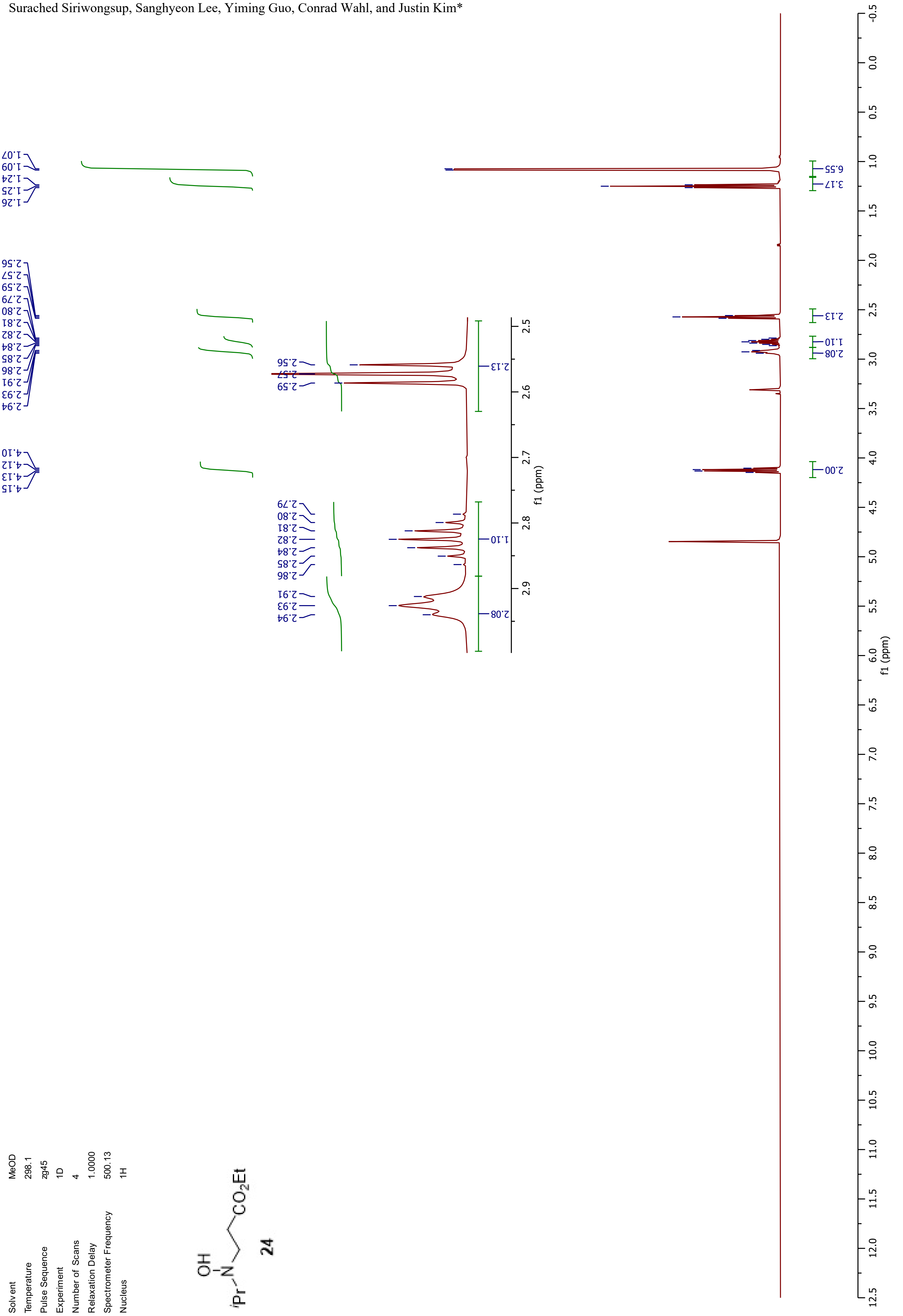

Parameter	Value
Solvent	MeOD
Temperature	298.1
Pulse Sequence	zg45
Experiment	1D
Number of Scans	4
Relaxation Delay	1.0000
Spectrometer Frequency	500.13
Nucleus	1H

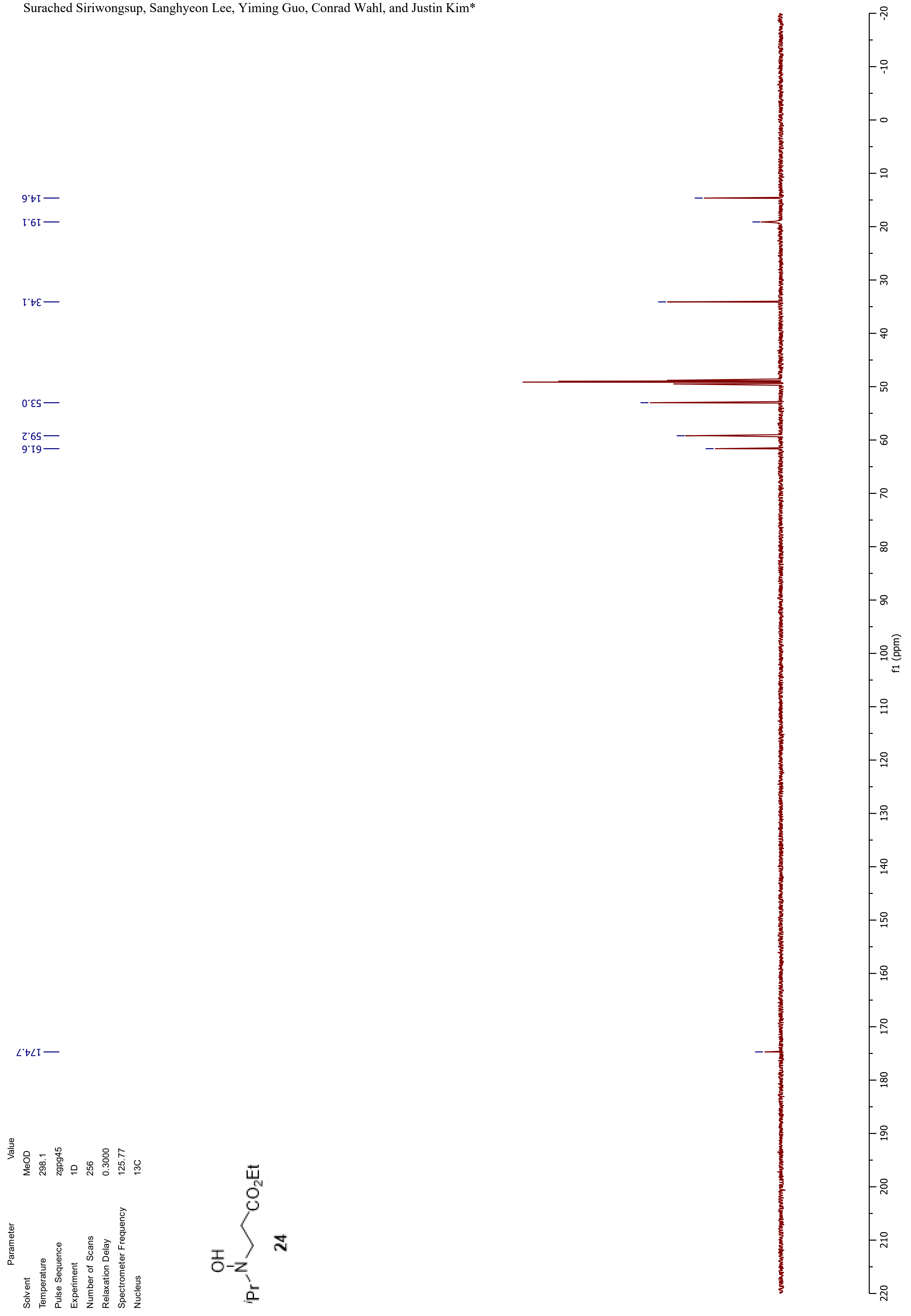
14.6
19.1
34.1
53.0
59.2
61.6
174.7
Parameter
Value
Solvent
MeOD
Temperature
298.1
Pulse Sequence
zgpg45
Experiment
1D
Number of Scans
256
Relaxation Delay
0.3000
Spectrometer Frequency
125.77
Nucleus
13C
OH
iPr
N
CO2Et
24
f1 (ppm)

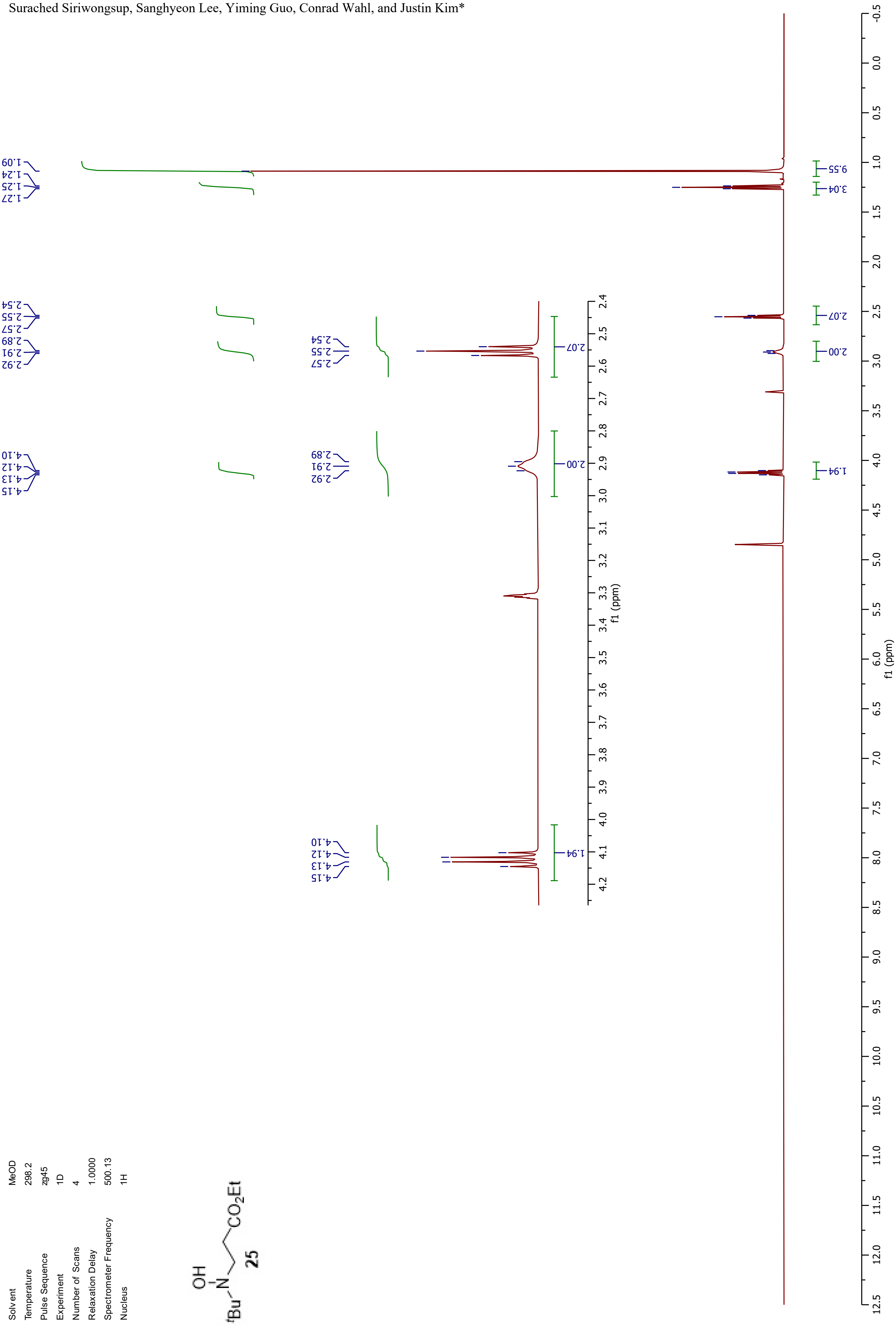

Parameter	Value
Solvent	MeOD
Temperature	298.2
Pulse Sequence	zg45
Experiment	1D
Number of Scans	4
Relaxation Delay	1.0000
Spectrometer Frequency	500.13
Nucleus	1H

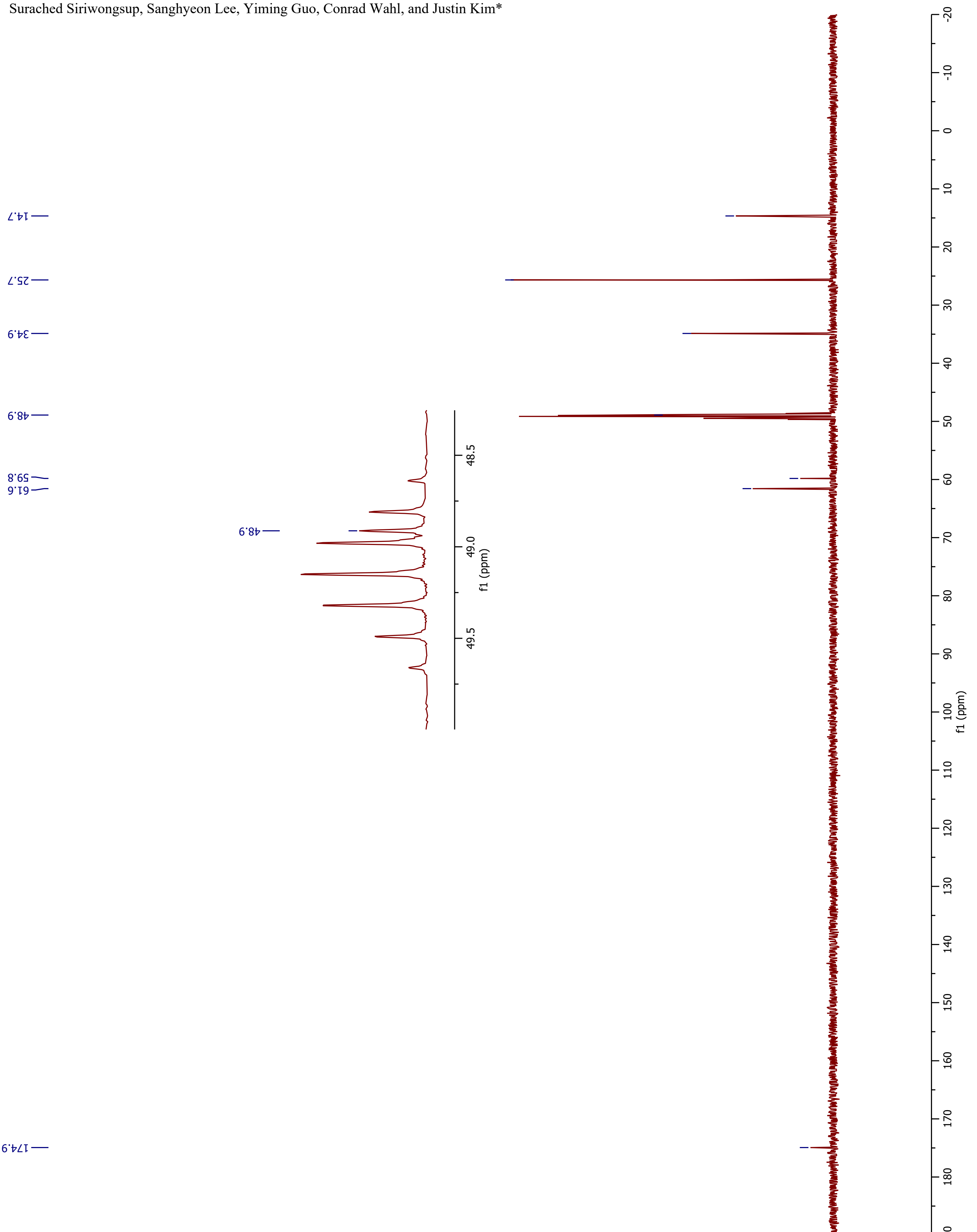

Parameter	Value
Solvent	MeOD
Temperature	298.2
Pulse Sequence	zgpg45
Experiment	1D
Number of Scans	128
Relaxation Delay	0.3000
Spectrometer Frequency	125.77
Nucleus	13C

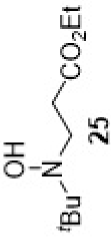

25

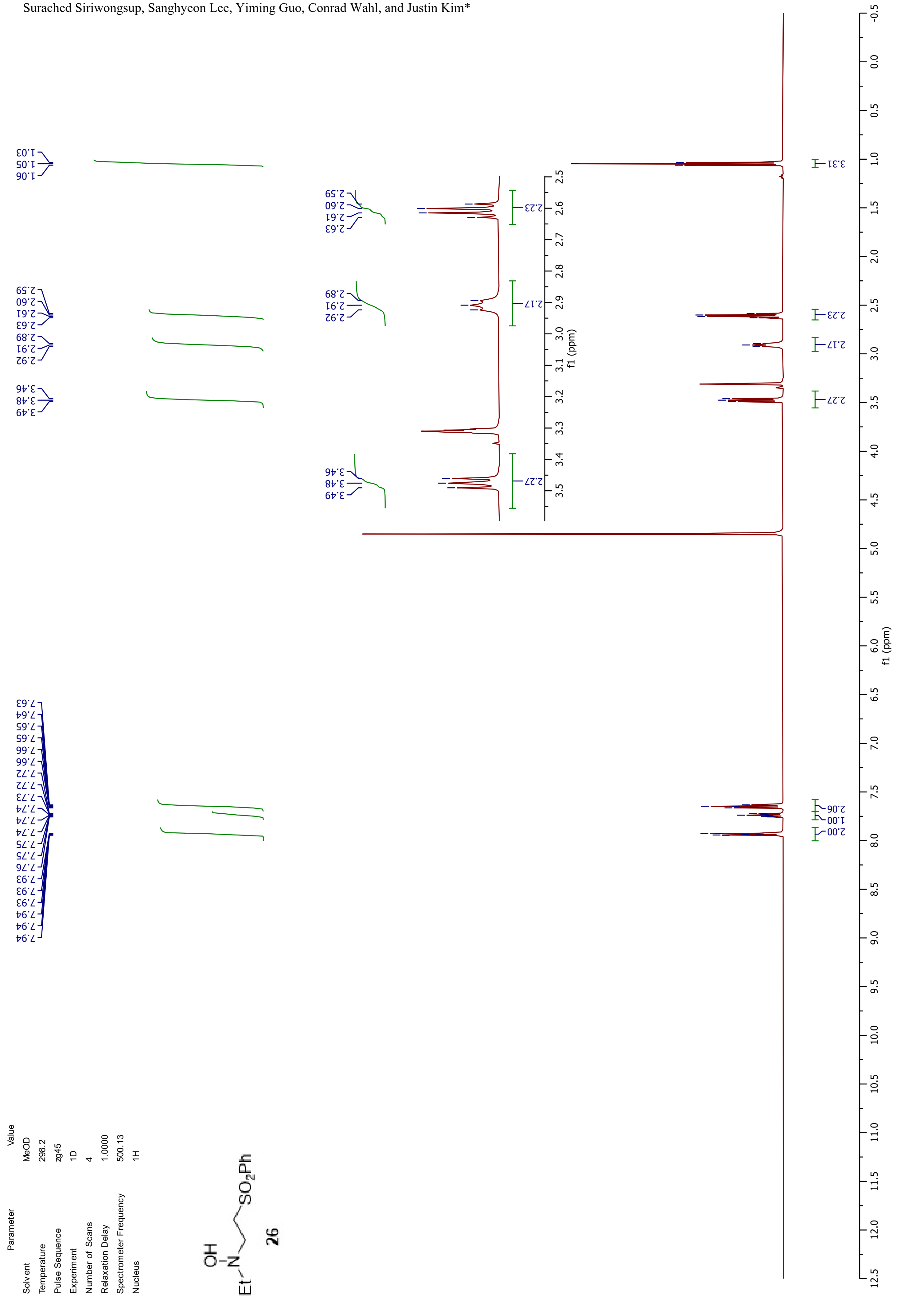
Parameter Value
Solvent MeOD
Temperature 298.2
Pulse Sequence zg45
Experiment 1D
Number of Scans 4
Relaxation Delay 1.0000
Spectrometer Frequency 500.13
Nucleus 1H
Et–N(OH)–CH2CH2–SO2Ph
26
f1 (ppm)

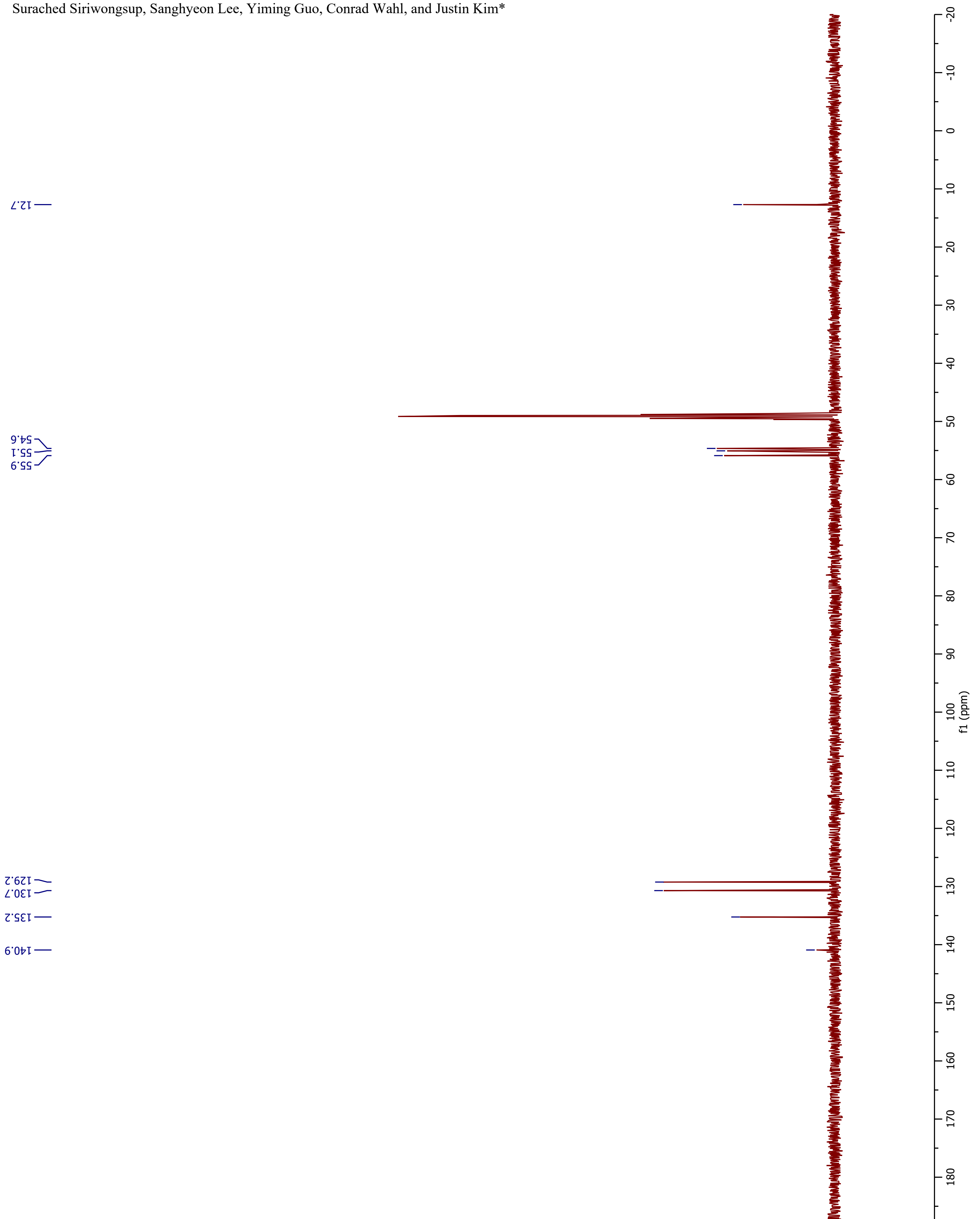

Parameter	Value
Solvent	MeOD
Temperature	298.2
Pulse Sequence	zgpg45
Experiment	1D
Number of Scans	128
Relaxation Delay	0.3000
Spectrometer Frequency	125.77
Nucleus	13C

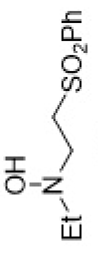

26

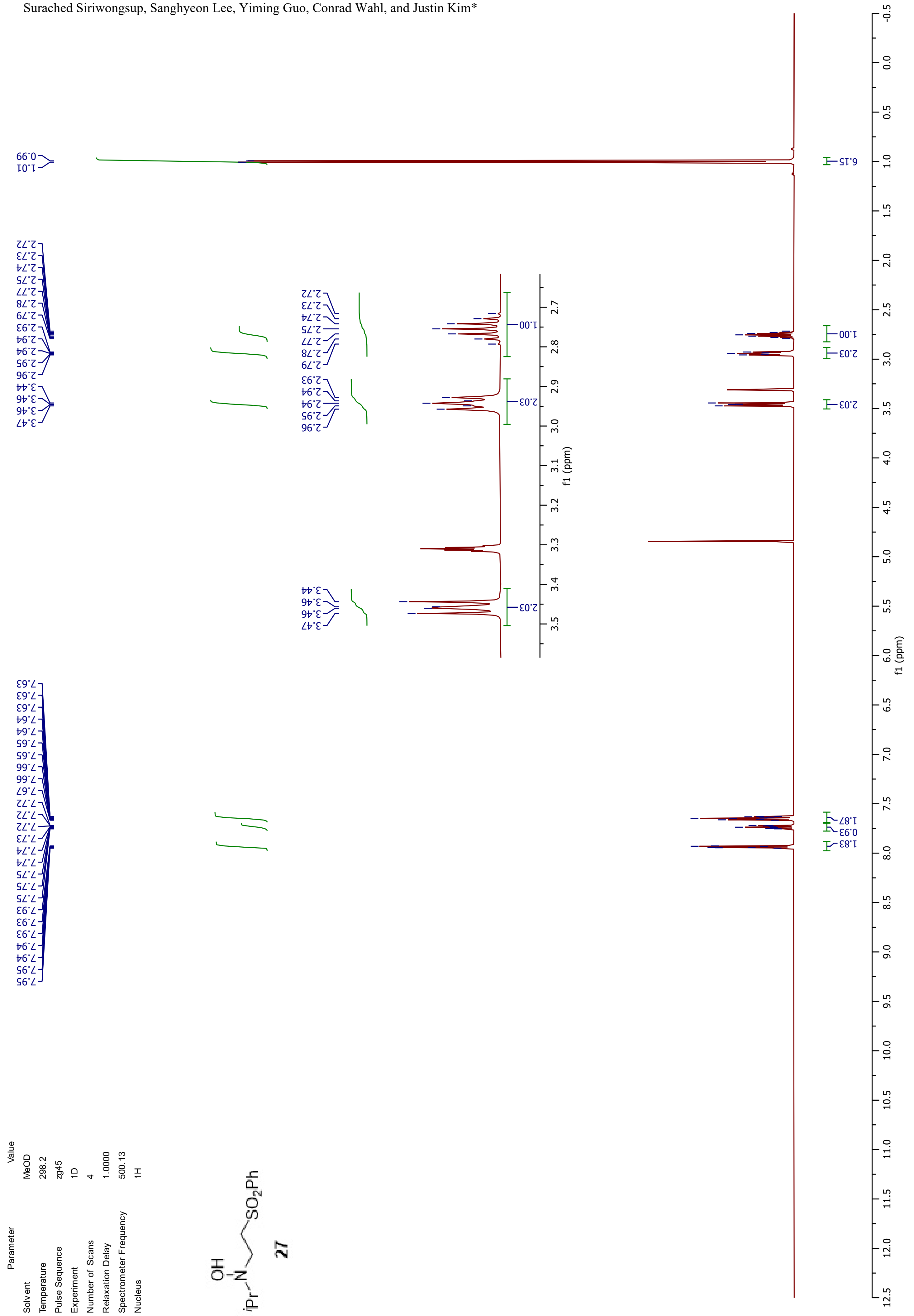
Parameter Value
Solvent MeOD
Temperature 298.2
Pulse Sequence zg45
Experiment 1D
Number of Scans 4
Relaxation Delay 1.0000
Spectrometer Frequency 500.13
Nucleus 1H
27
7.95
7.95
7.94
7.94
7.93
7.93
7.93
7.75
7.75
7.75
7.74
7.74
7.73
7.72
7.72
7.67
7.66
7.66
7.65
7.65
7.64
7.64
7.63
7.63
7.63
3.47
3.46
3.46
3.44
2.96
2.95
2.94
2.94
2.93
2.79
2.78
2.77
2.75
2.74
2.73
2.72
1.01
0.99
1.83
0.93
1.87
2.03
2.03
1.00
6.15
f1 (ppm)

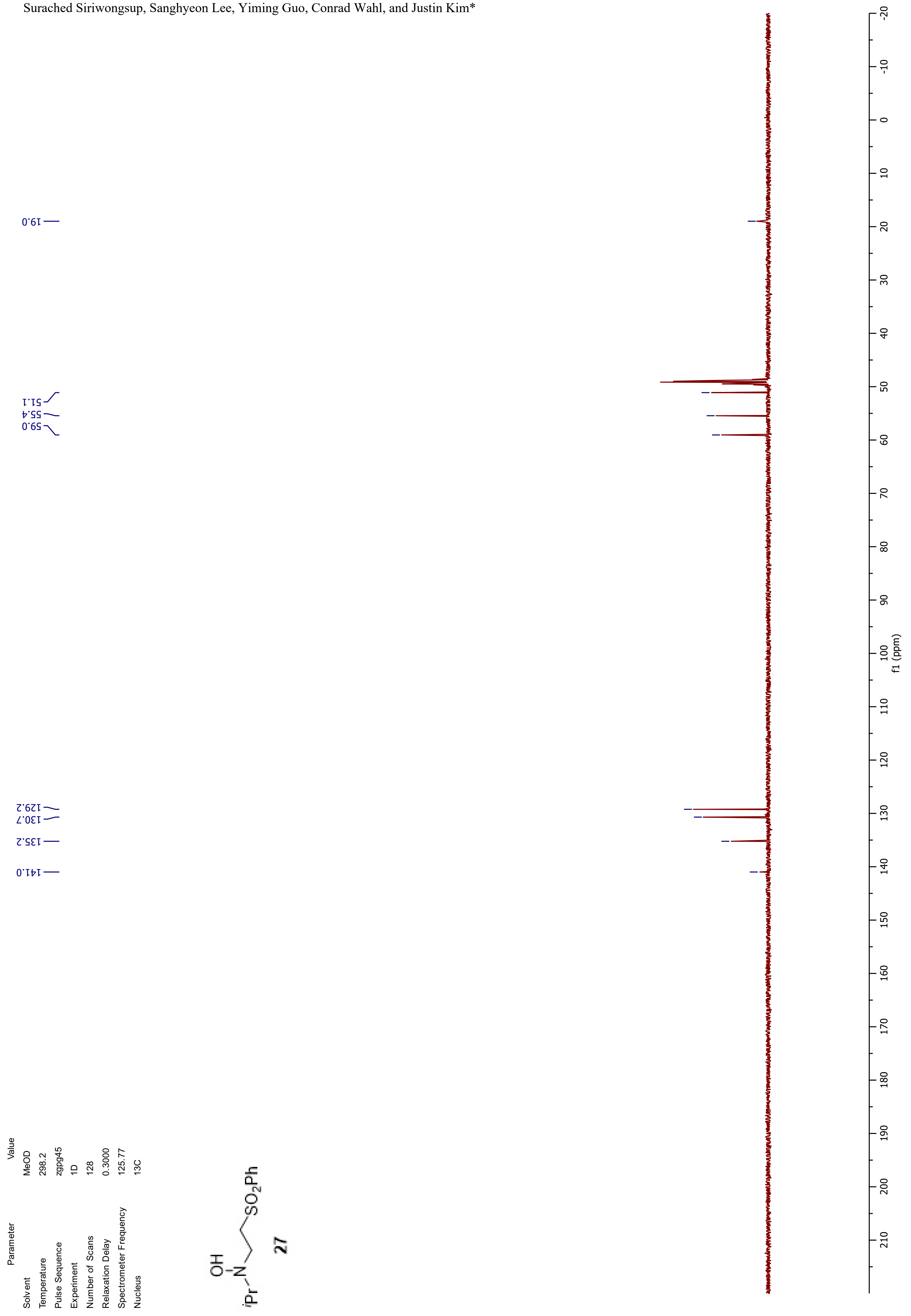
19.0
51.1
55.4
59.0
129.2
130.7
135.2
141.0
f1 (ppm)
Parameter | Value
Solvent | MeOD
Temperature | 298.2
Pulse Sequence | zgpg45
Experiment | 1D
Number of Scans | 128
Relaxation Delay | 0.3000
Spectrometer Frequency | 125.77
Nucleus | 13C
OH
iPr
N
SO2Ph
27

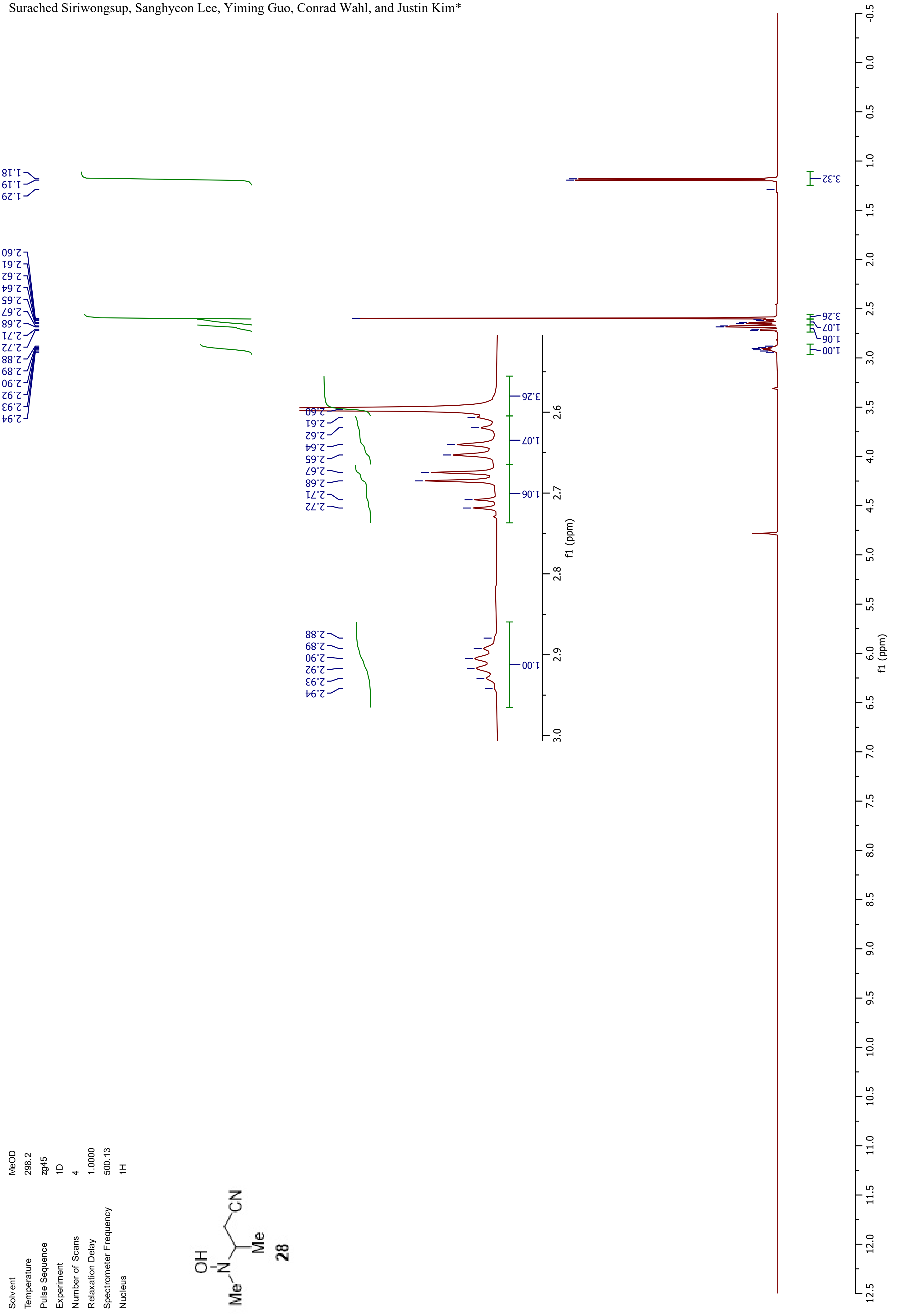

Parameter	Value
Solvent	MeOD
Temperature	298.2
Pulse Sequence	zg45
Experiment	1D
Number of Scans	4
Relaxation Delay	1.0000
Spectrometer Frequency	500.13
Nucleus	1H

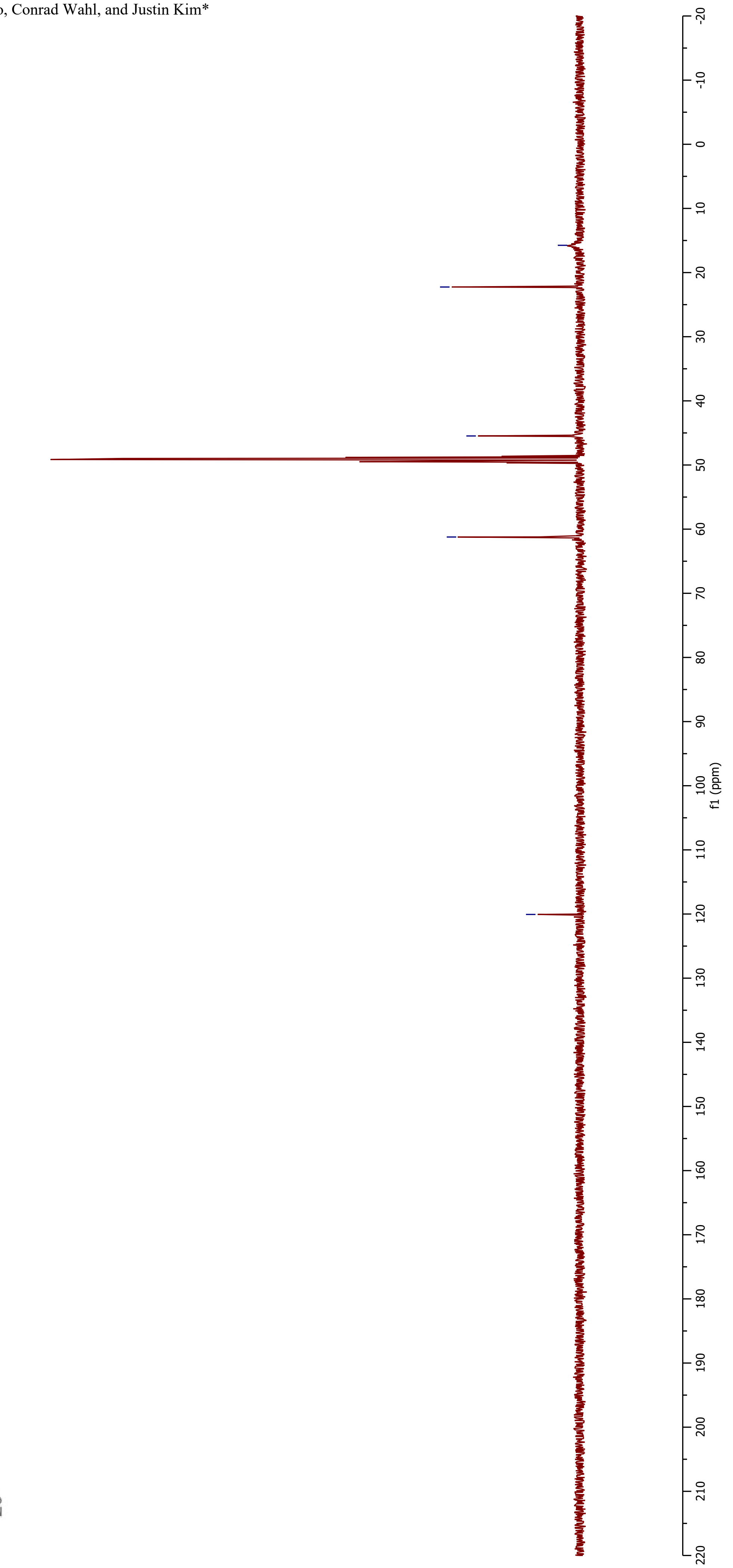

Parameter	Value
Solvent	MeOD
Temperature	298.1
Pulse Sequence	zgpg45
Experiment	1D
Number of Scans	512
Relaxation Delay	0.3000
Spectrometer Frequency	125.77
Nucleus	13C

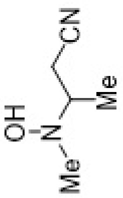

28

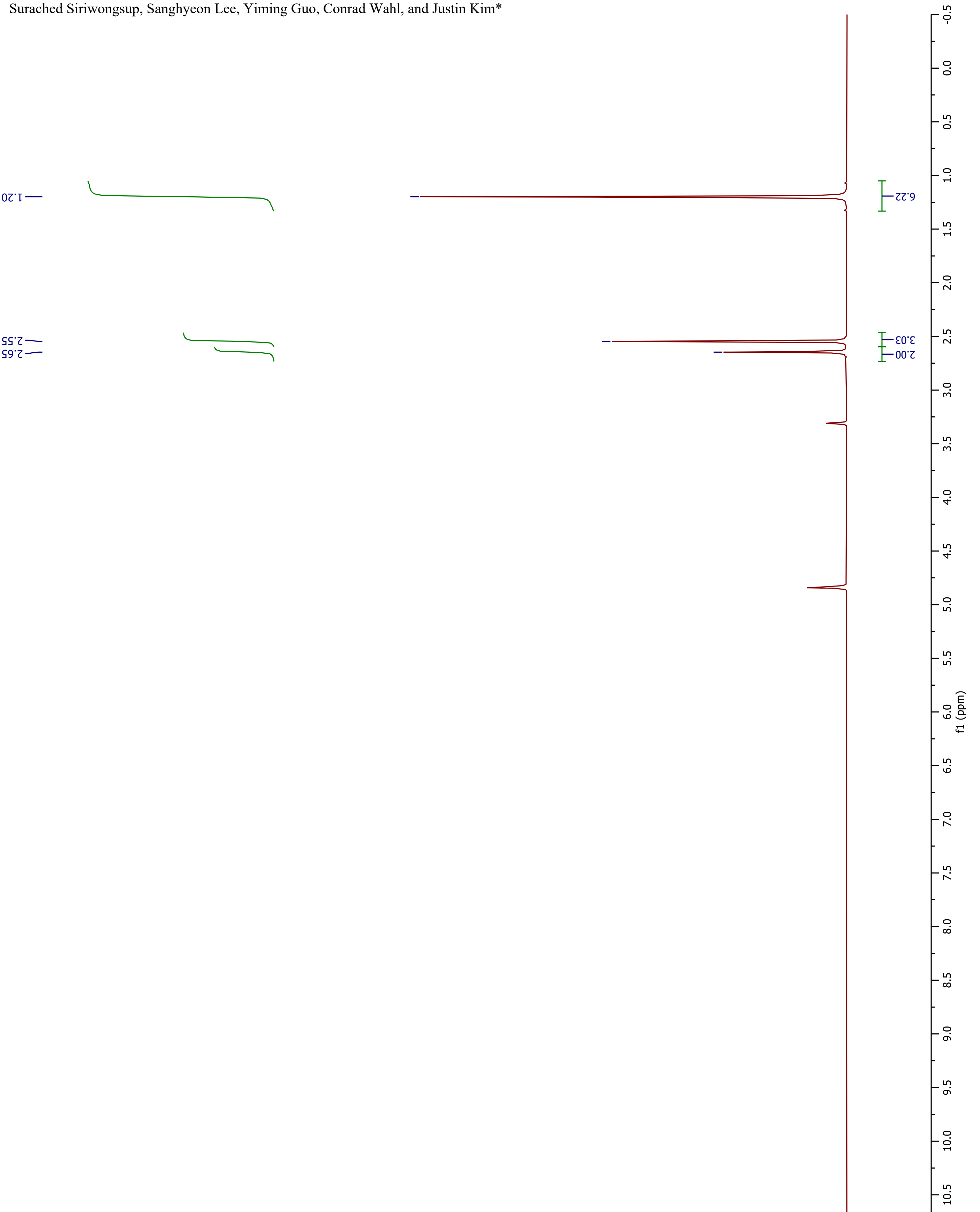

Parameter	Value
Solvent	MeOD
Temperature	298.1
Pulse Sequence	zg45
Experiment	1D
Number of Scans	4
Relaxation Delay	1.0000
Spectrometer Frequency	500.13
Nucleus	1H

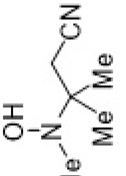

29

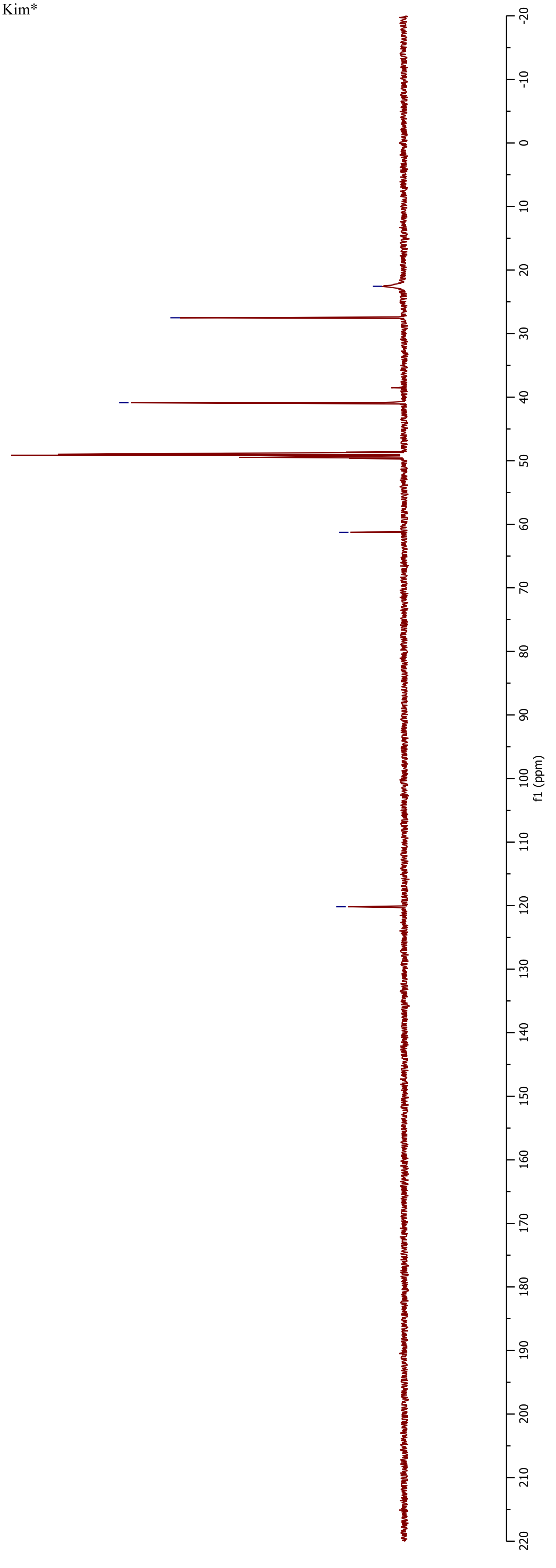

Parameter	Value
Solvent	MeOD
Temperature	298.1
Pulse Sequence	zgpg45
Experiment	1D
Number of Scans	512
Relaxation Delay	0.3000
Spectrometer Frequency	125.77
Nucleus	13C

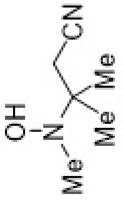

29

Parameter	Value
Solvent	MeOD
Temperature	298.2
Pulse Sequence	zg45
Experiment	1D
Number of Scans	4
Relaxation Delay	1.0000
Spectrometer Frequency	500.13
Nucleus	1H

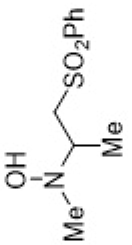

30

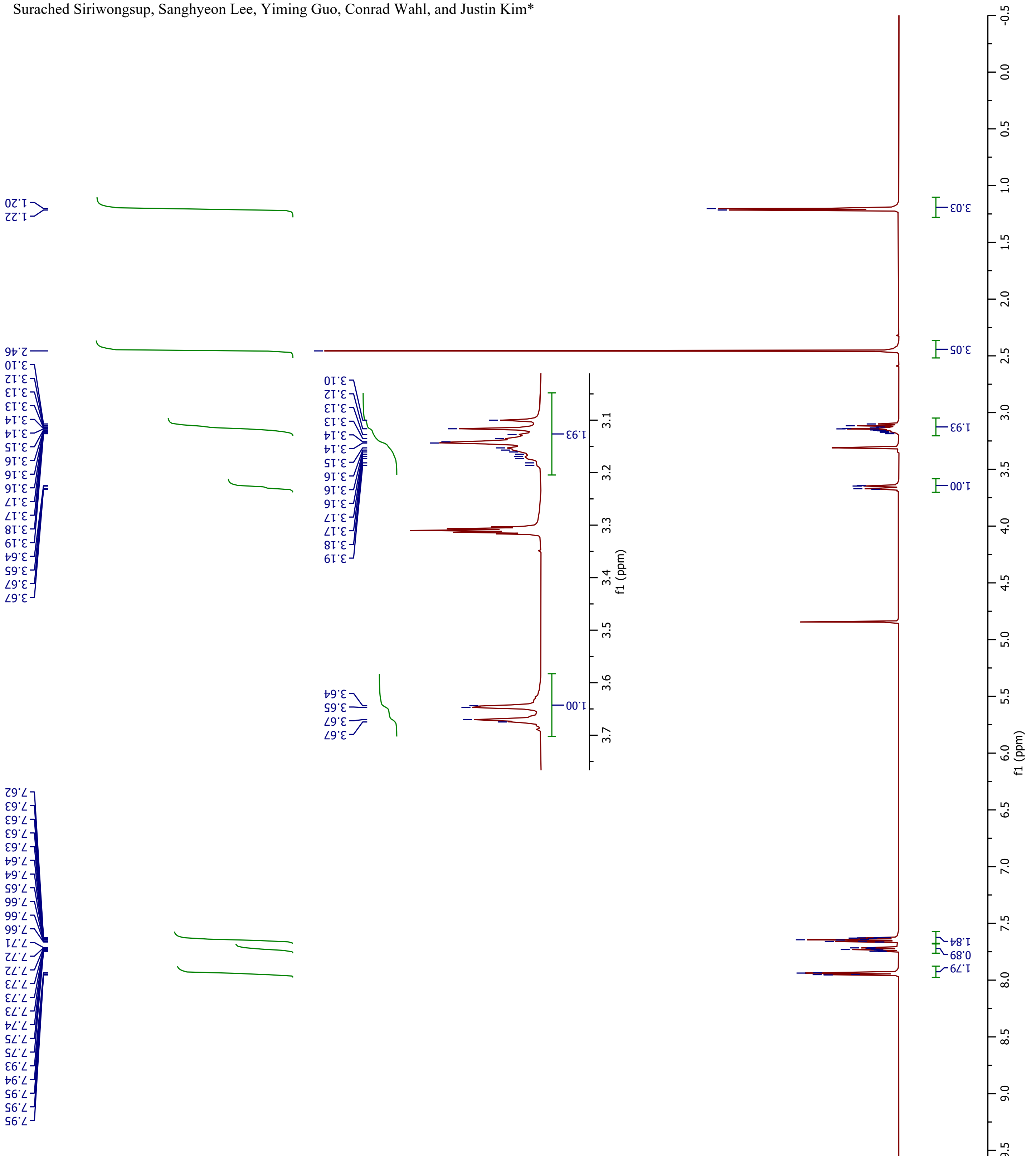

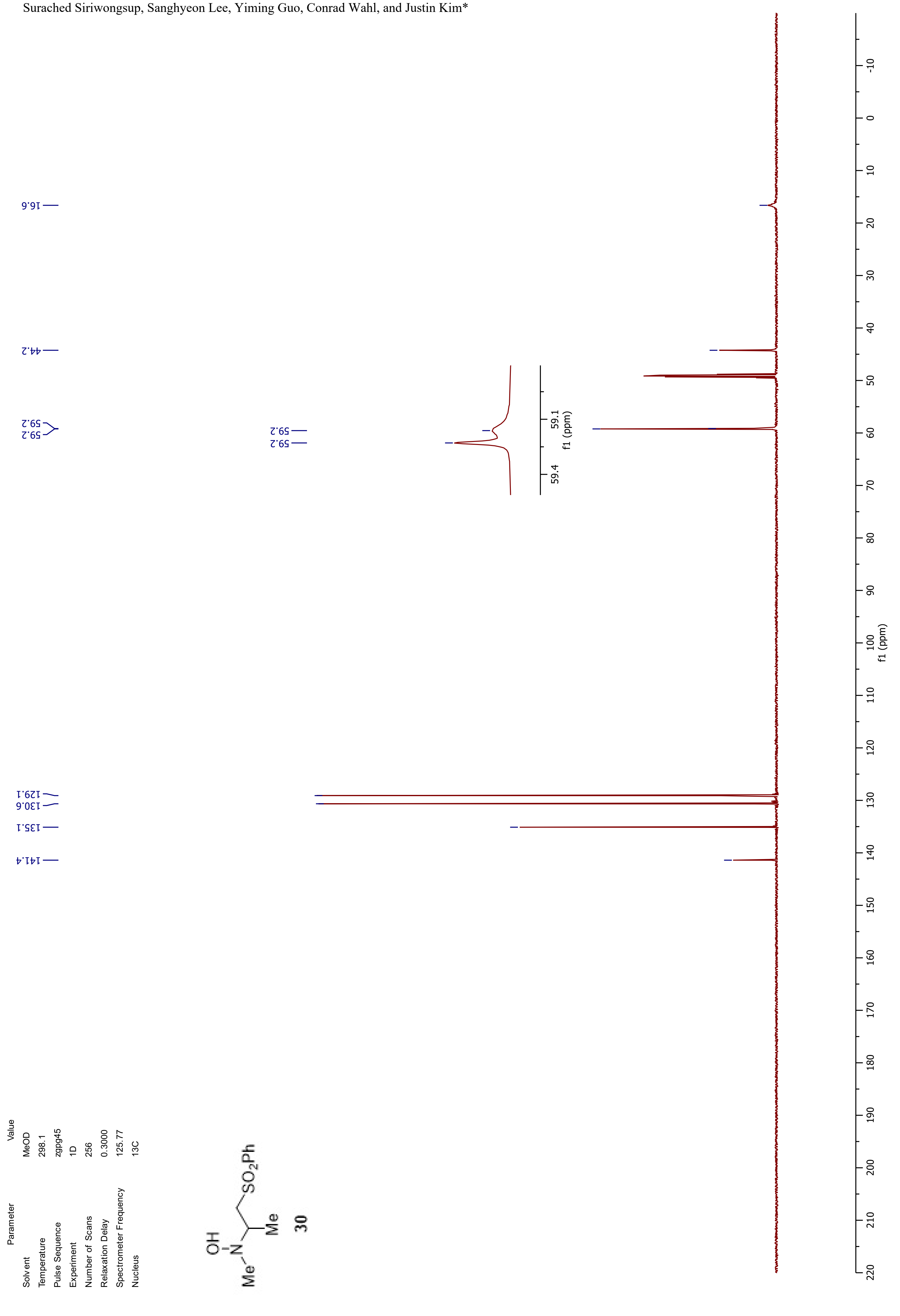
Parameter
Value
Solvent
MeOD
Temperature
298.1
Pulse Sequence
zgpg45
Experiment
1D
Number of Scans
256
Relaxation Delay
0.3000
Spectrometer Frequency
125.77
Nucleus
13C
30
16.6
44.2
59.2
59.2
129.1
130.6
135.1
141.4
59.1
59.4
f1 (ppm)

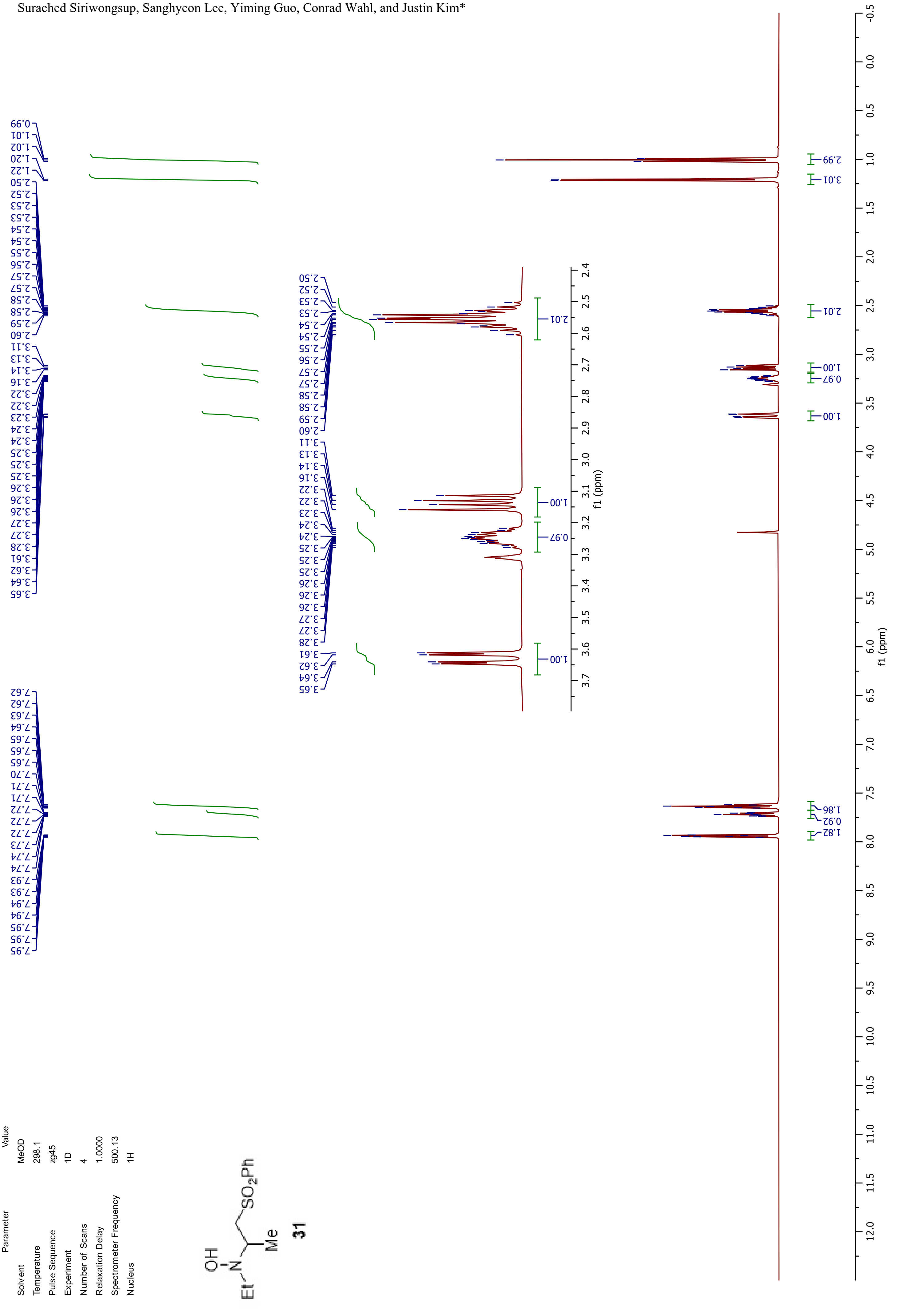
Parameter | Value
Solvent | MeOD
Temperature | 298.1
Pulse Sequence | zg45
Experiment | 1D
Number of Scans | 4
Relaxation Delay | 1.0000
Spectrometer Frequency | 500.13
Nucleus | 1H
31
0.99 1.01 1.02 1.20 1.22
2.50 2.52 2.53 2.53 2.54 2.54 2.55 2.56 2.57 2.57 2.58 2.58 2.59 2.60
3.11 3.13 3.14 3.16 3.22 3.22 3.23 3.24 3.24 3.25 3.25 3.25 3.26 3.26 3.26 3.27 3.27 3.28
3.61 3.62 3.64 3.65
7.62 7.62 7.63 7.64 7.65 7.65 7.65 7.70 7.71 7.71 7.72 7.72 7.72 7.73 7.74 7.74 7.93 7.93 7.94 7.94 7.95 7.95 7.95
2.99 3.01 2.01 1.00 0.97 1.00 1.86 0.92 1.82
f1 (ppm)

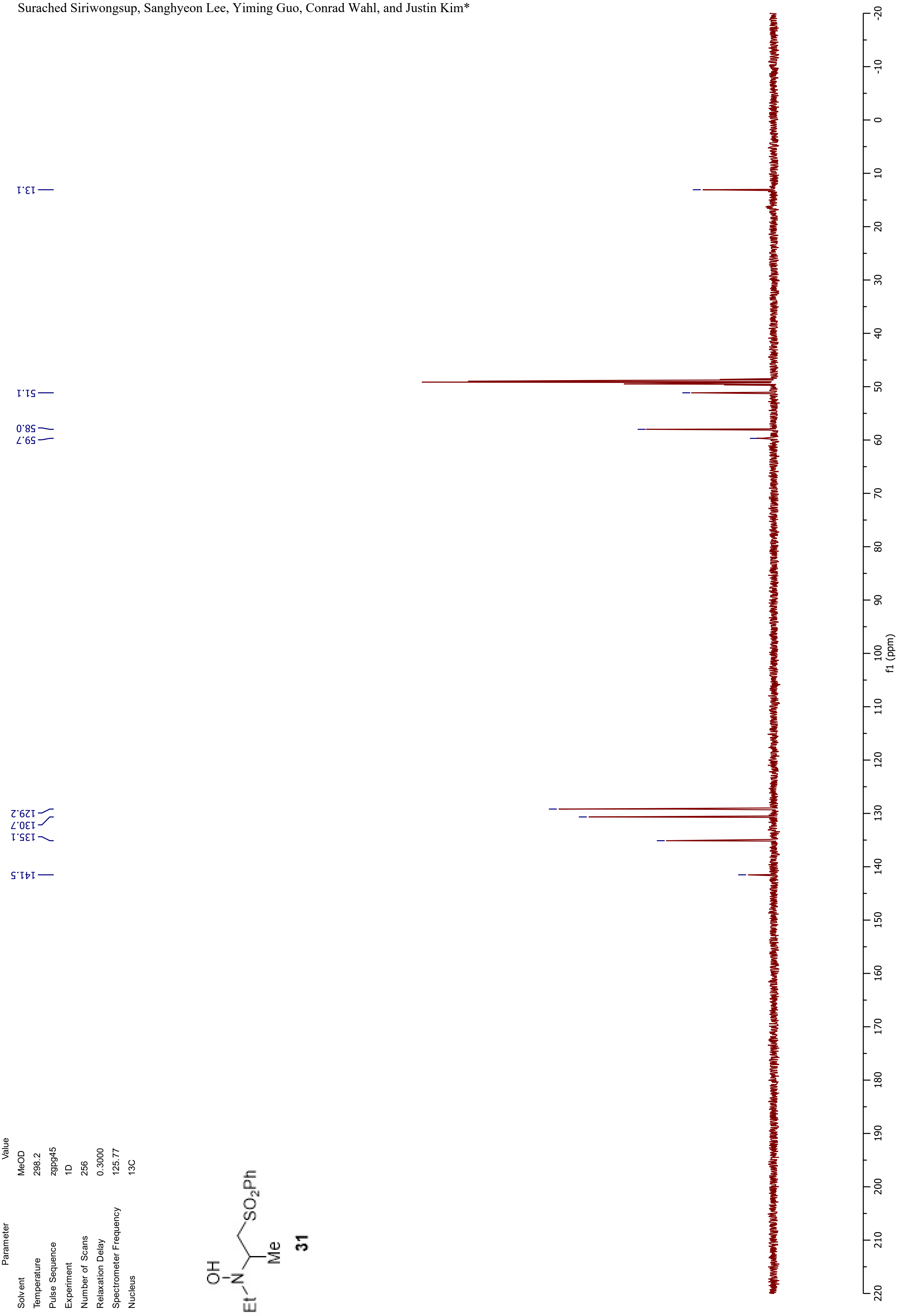

Parameter	Value
Solvent	MeOD
Temperature	298.2
Pulse Sequence	zgpg45
Experiment	1D
Number of Scans	256
Relaxation Delay	0.3000
Spectrometer Frequency	125.77
Nucleus	13C

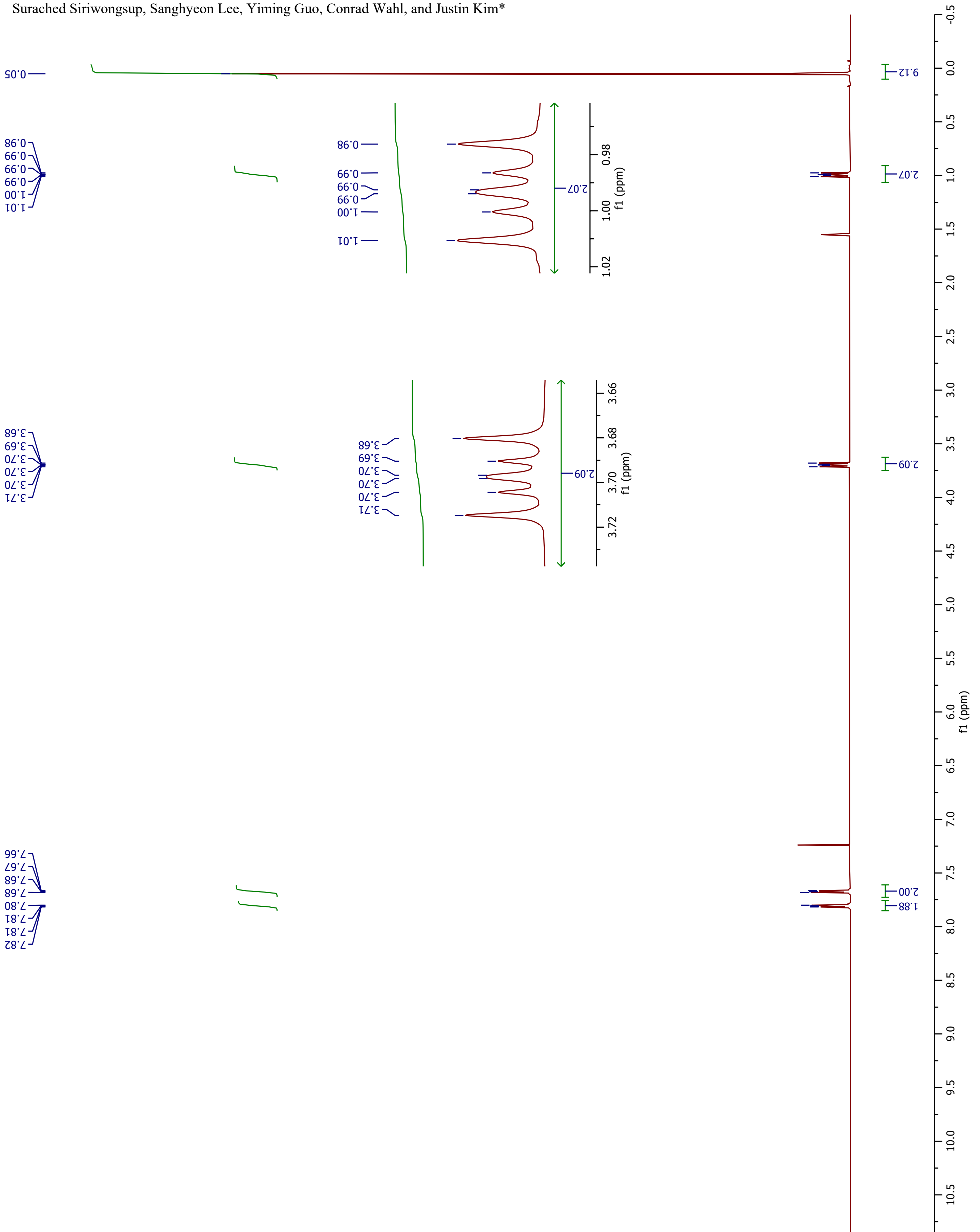

Parameter	Value
Solvent	CDCl3
Temperature	298.1
Pulse Sequence	zg45
Experiment	1D
Number of Scans	4
Relaxation Delay	1.0000
Spectrometer Frequency	500.13
Nucleus	1H

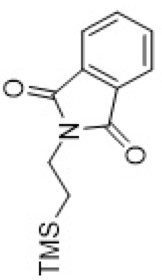

S3

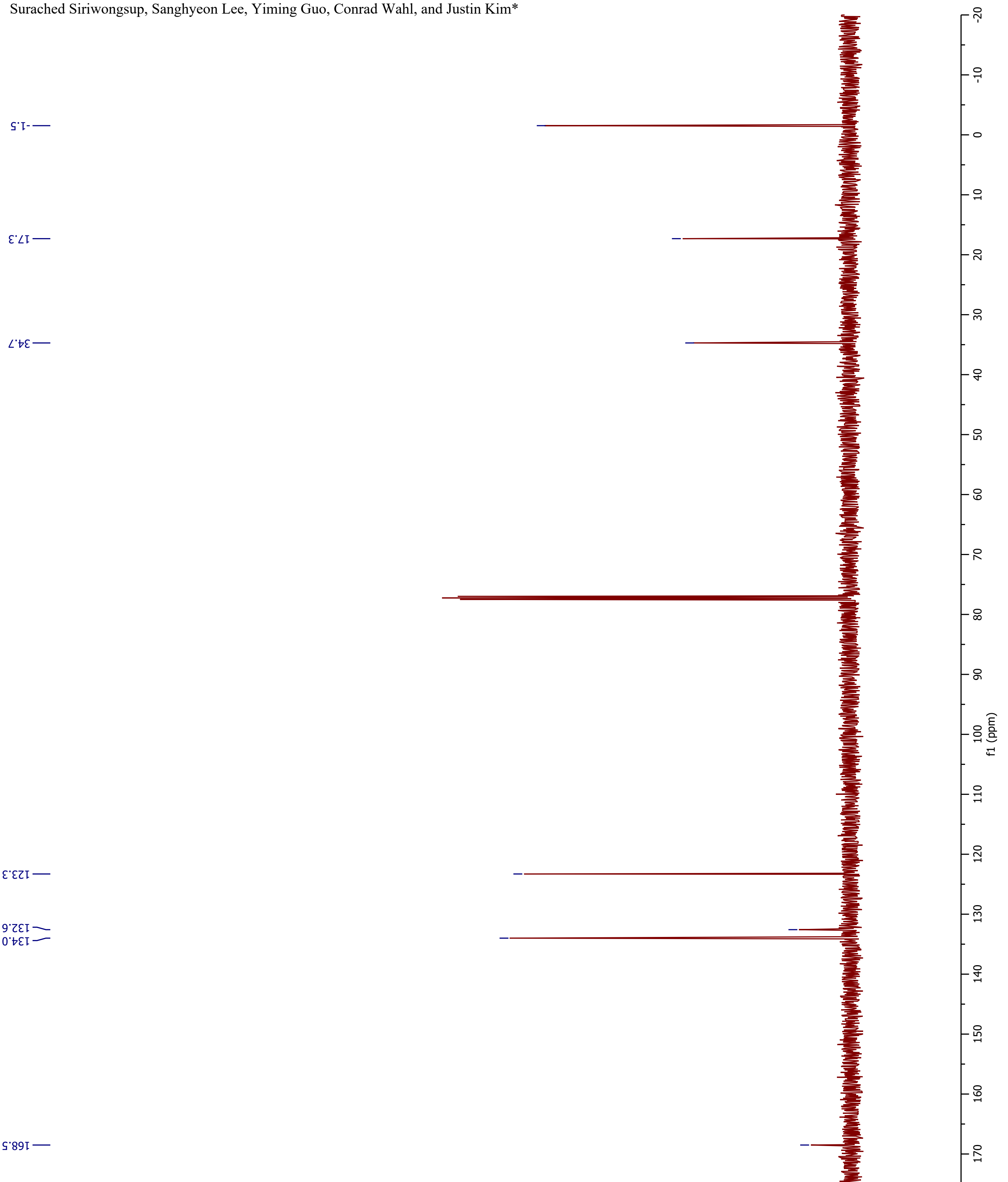

Parameter	Value
Solvent	CDCl3
Temperature	298.2
Pulse Sequence	zgpg45
Experiment	1D
Number of Scans	256
Relaxation Delay	0.3000
Spectrometer Frequency	125.77
Nucleus	13C

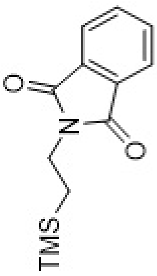

S3

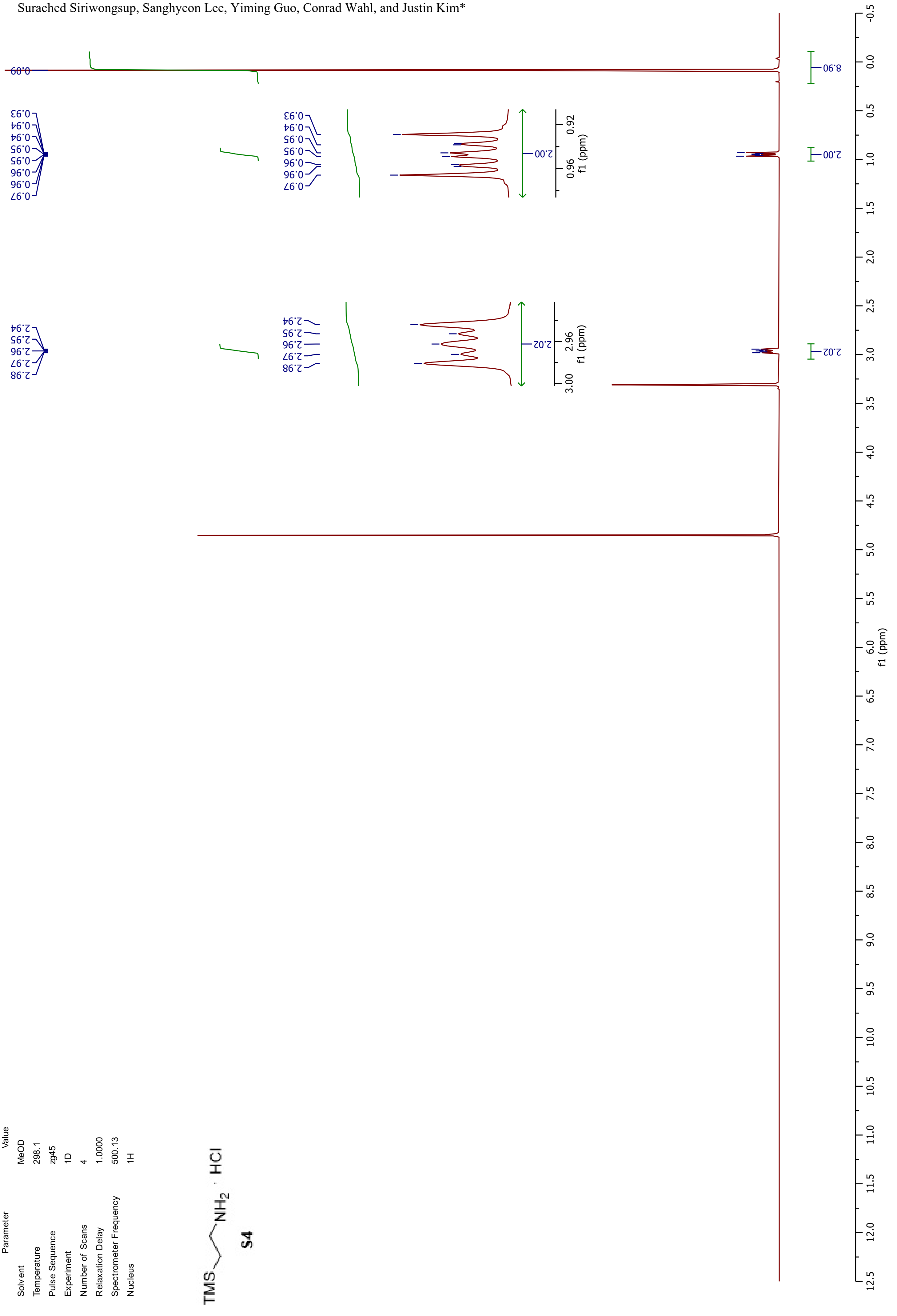
Parameter
Value
Solvent
MeOD
Temperature
298.1
Pulse Sequence
zg45
Experiment
1D
Number of Scans
4
Relaxation Delay
1.0000
Spectrometer Frequency
500.13
Nucleus
1H
TMS
NH2 · HCl
S4
0.09
0.93
0.94
0.94
0.95
0.95
0.96
0.96
0.97
2.94
2.95
2.96
2.97
2.98
8.90
2.00
2.02
f1 (ppm)

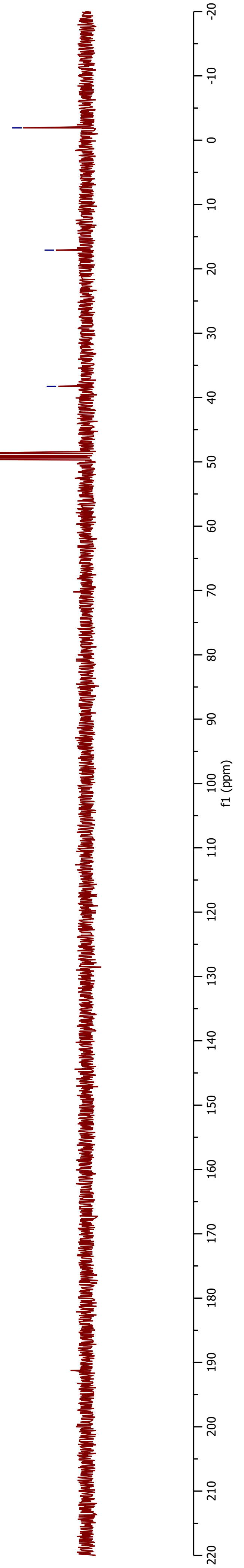

Parameter	Value
Solvent	MeOD
Temperature	298.2
Pulse Sequence	zgpg45
Experiment	1D
Number of Scans	128
Relaxation Delay	0.3000
Spectrometer Frequency	125.77
Nucleus	13C

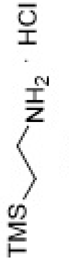

S4

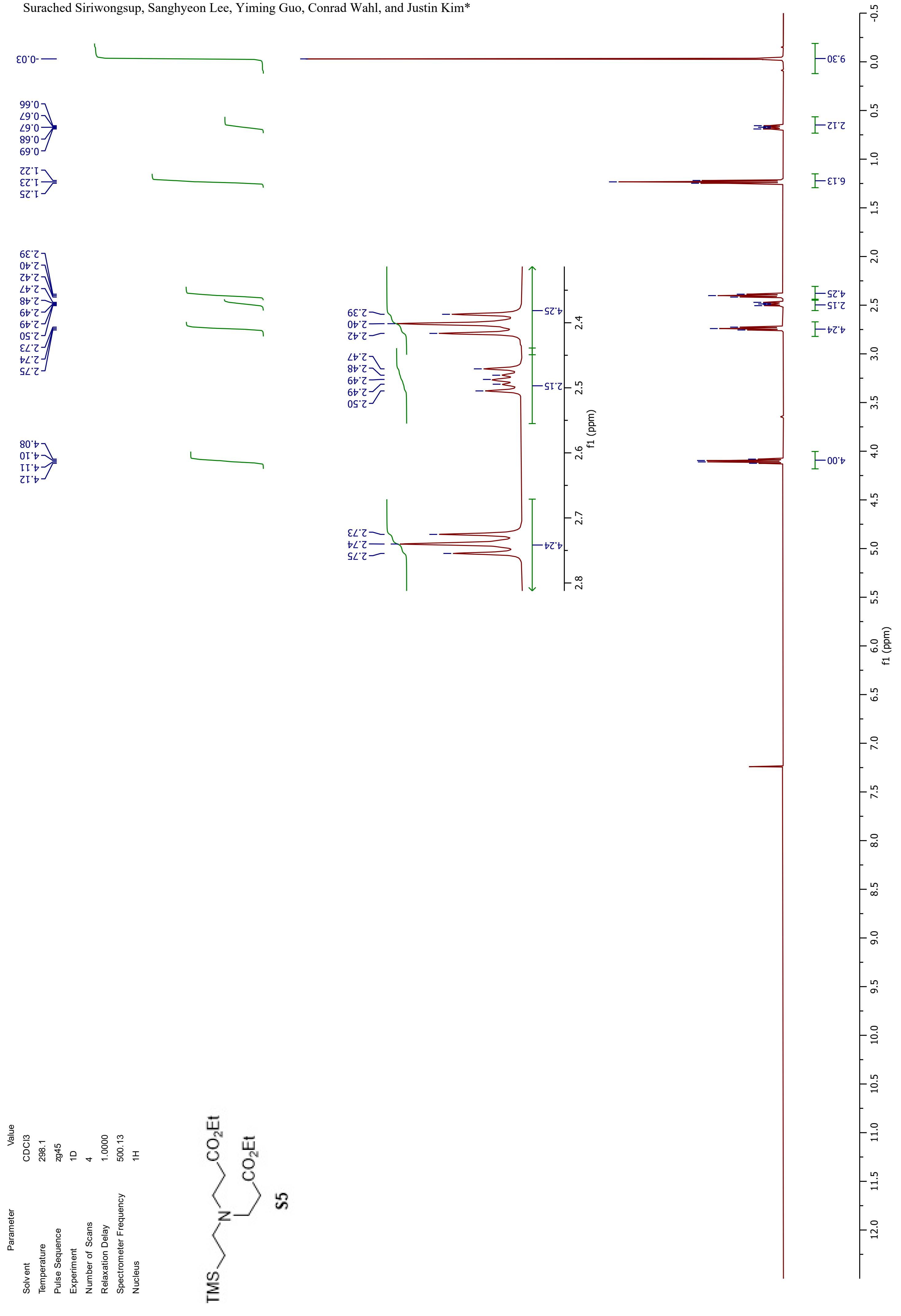
-0.03
0.66
0.67
0.67
0.68
0.69
1.22
1.23
1.25
2.39
2.40
2.42
2.47
2.48
2.49
2.49
2.50
2.73
2.74
2.75
4.08
4.10
4.11
4.12
9.30
2.12
6.13
4.25
2.15
4.24
4.00
f1 (ppm)
Parameter
Value
Solvent
CDCl3
Temperature
298.1
Pulse Sequence
zg45
Experiment
1D
Number of Scans
4
Relaxation Delay
1.0000
Spectrometer Frequency
500.13
Nucleus
1H
TMS
N
CO_2Et
CO_2Et
S5

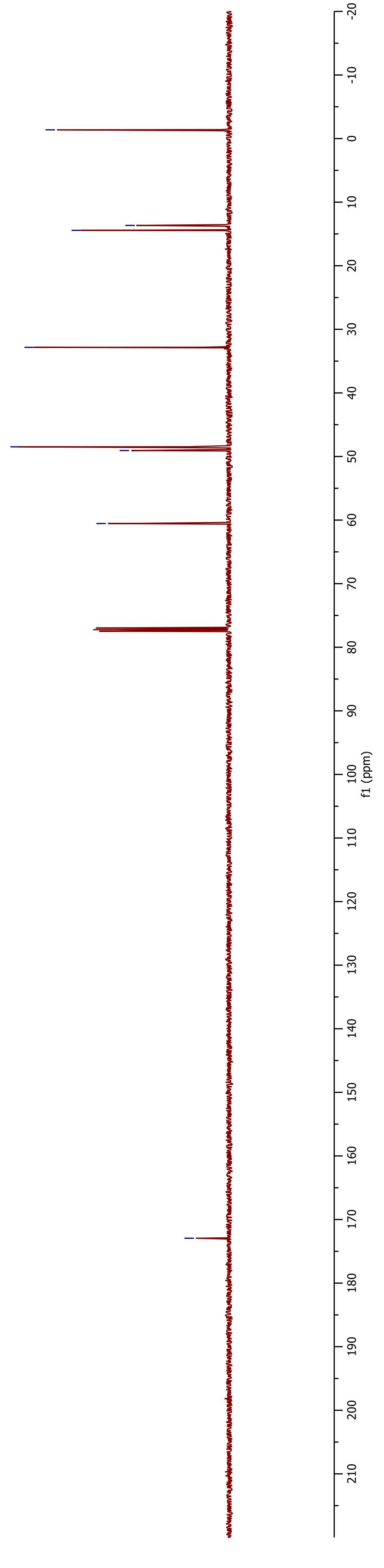

Parameter	Value
Solvent	CDCl3
Temperature	298.1
Pulse Sequence	zgpg45
Experiment	1D
Number of Scans	512
Relaxation Delay	0.3000
Spectrometer Frequency	125.77
Nucleus	13C

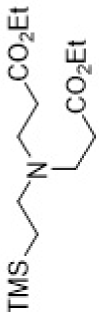

S5

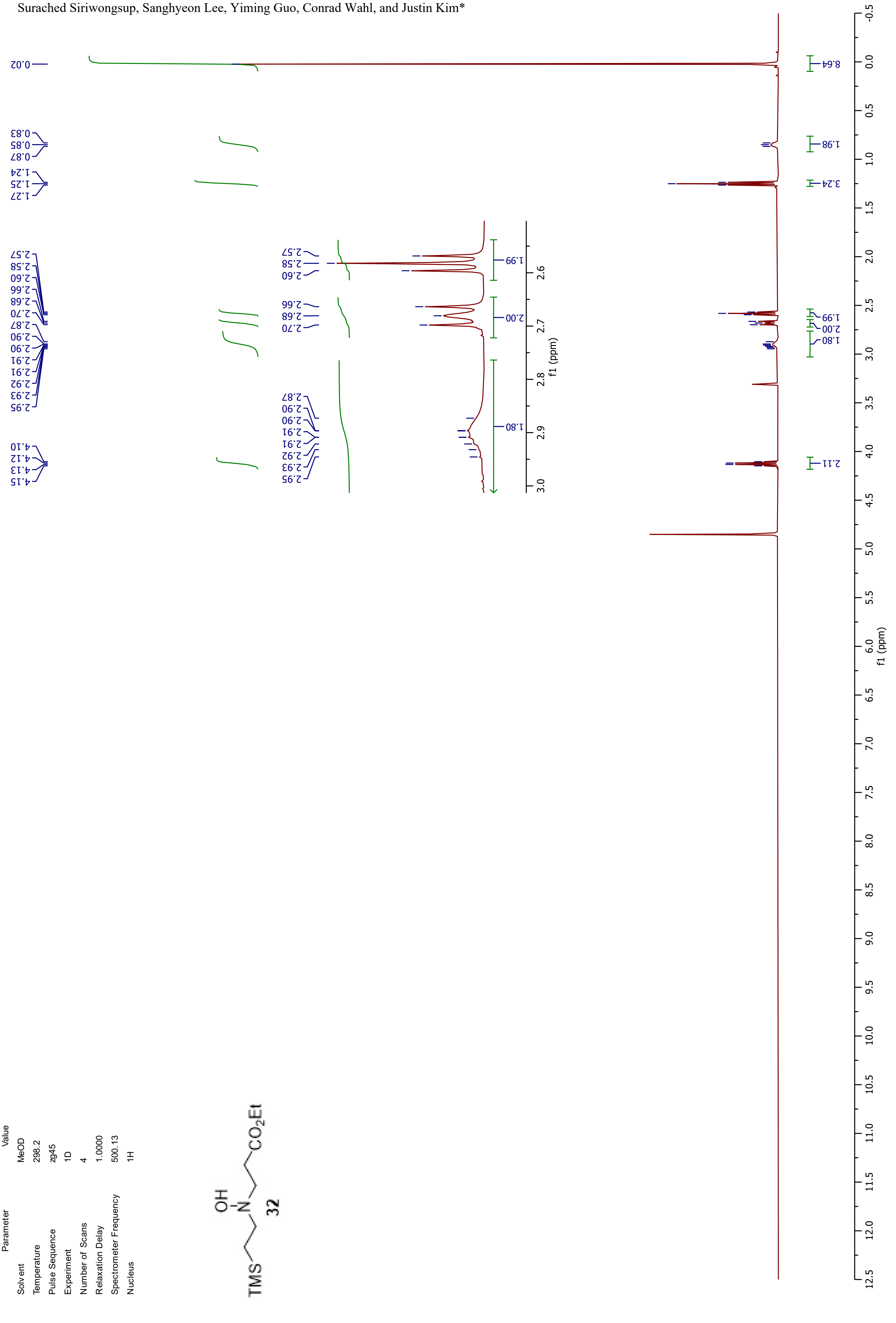
Parameter Value
Solvent MeOD
Temperature 298.2
Pulse Sequence zg45
Experiment 1D
Number of Scans 4
Relaxation Delay 1.0000
Spectrometer Frequency 500.13
Nucleus 1H
TMS
OH
N
CO2Et
32
f1 (ppm)

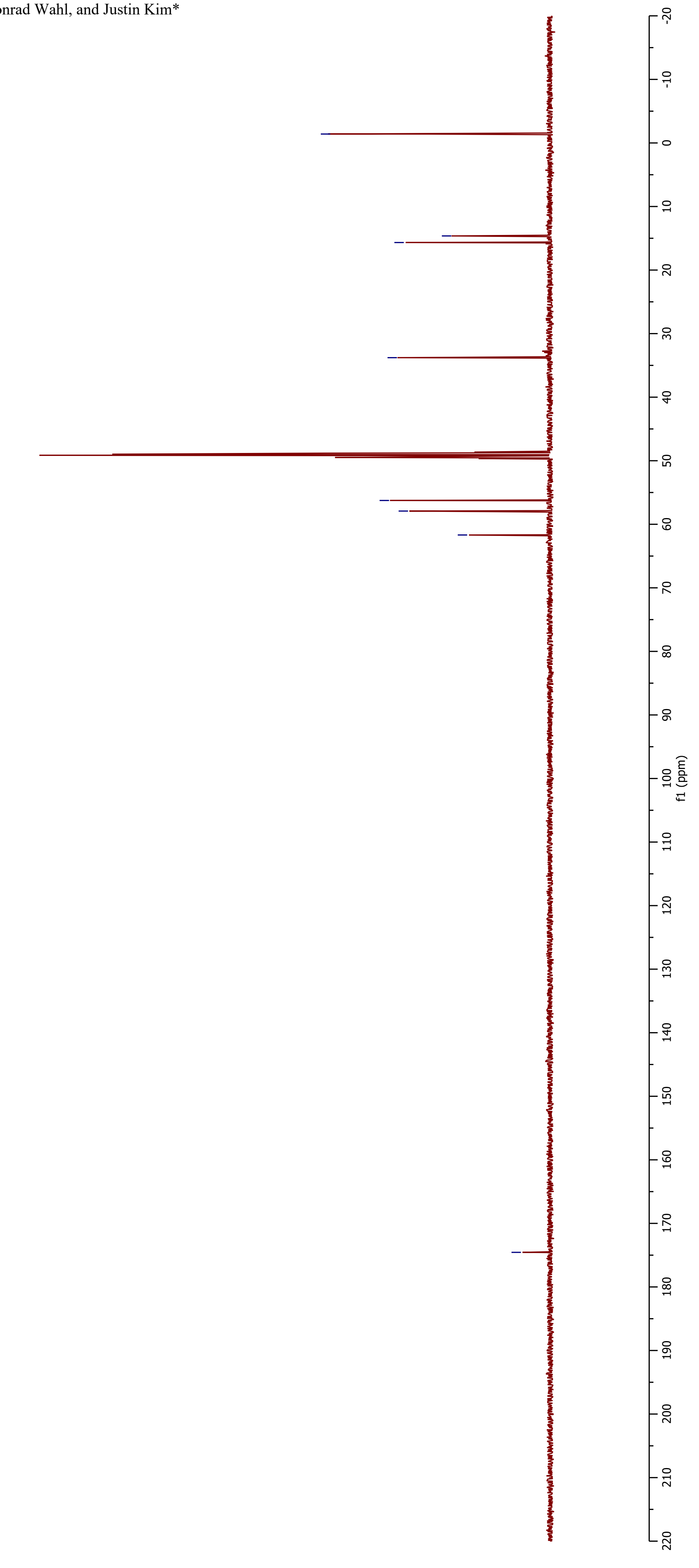

Parameter	Value
Solvent	MeOD
Temperature	298.1
Pulse Sequence	zgpg45
Experiment	1D
Number of Scans	512
Relaxation Delay	0.3000
Spectrometer Frequency	125.77
Nucleus	13C

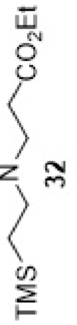

32

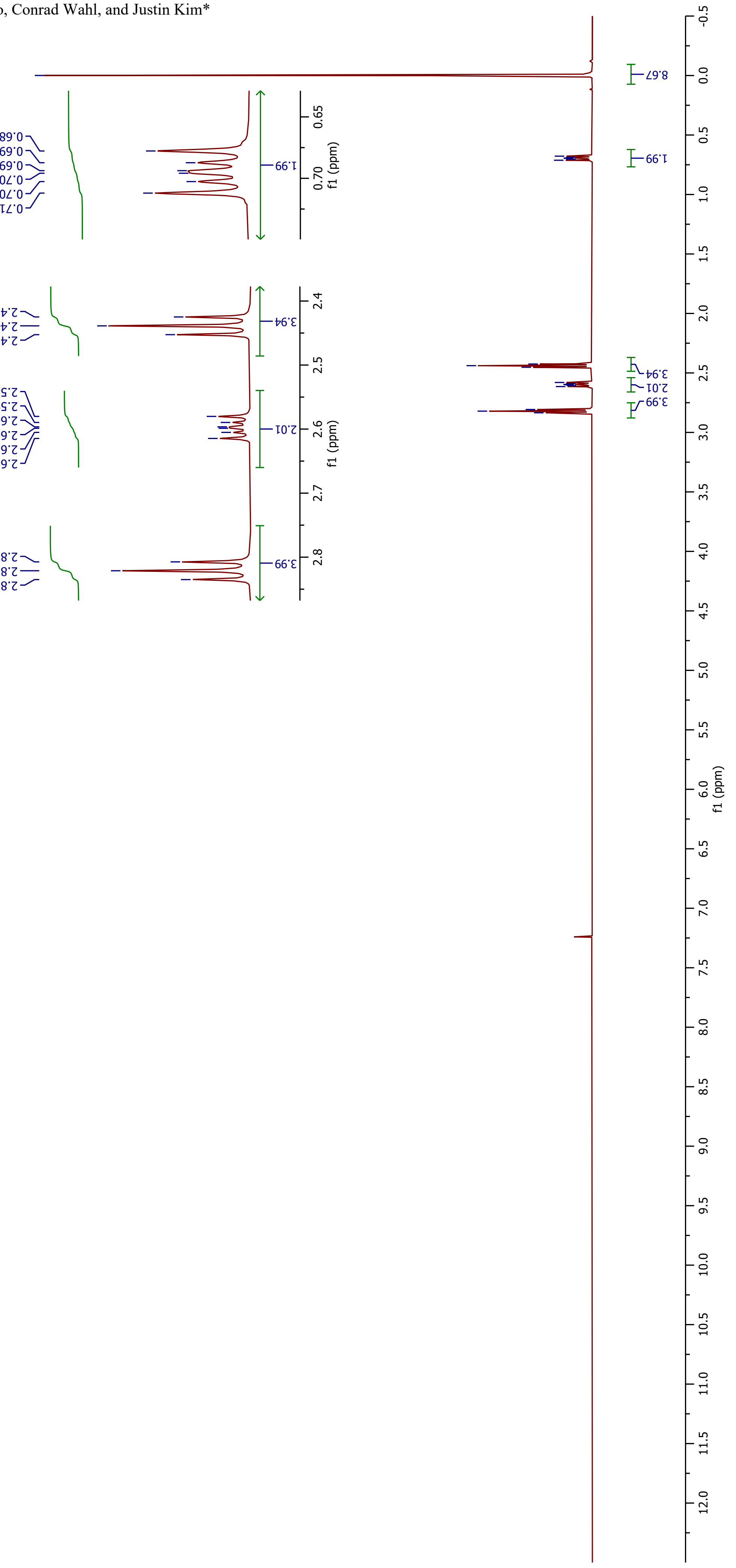

Parameter	Value
Solvent	CDCl3
Temperature	298.1
Pulse Sequence	zg45
Experiment	1D
Number of Scans	4
Relaxation Delay	1.0000
Spectrometer Frequency	500.13
Nucleus	1H

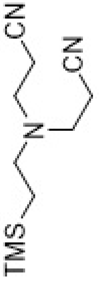

S6

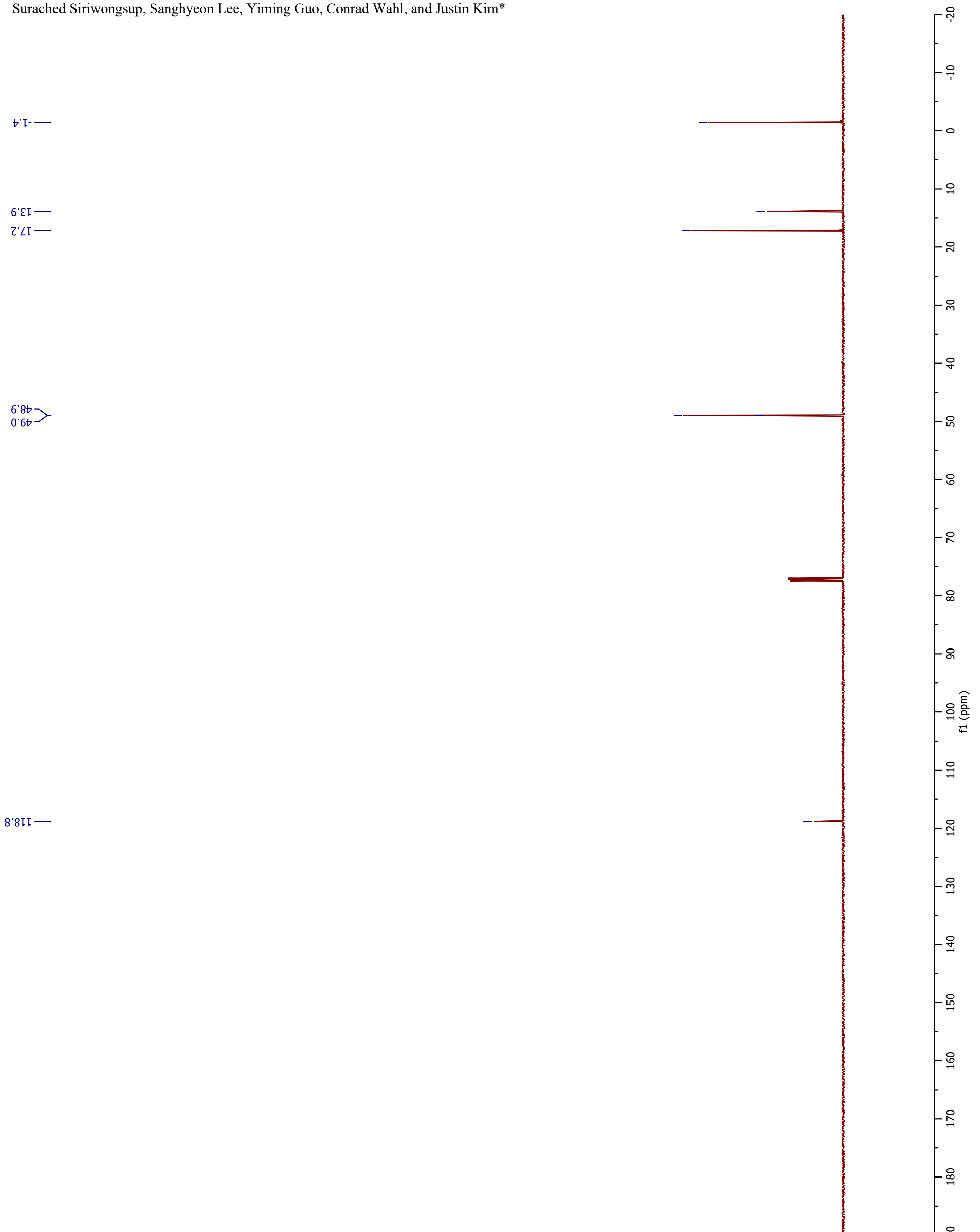

Parameter	Value
Solvent	CDCl3
Temperature	298.2
Pulse Sequence	zgpg45
Experiment	1D
Number of Scans	512
Relaxation Delay	0.3000
Spectrometer Frequency	125.77
Nucleus	13C

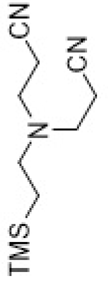

S6

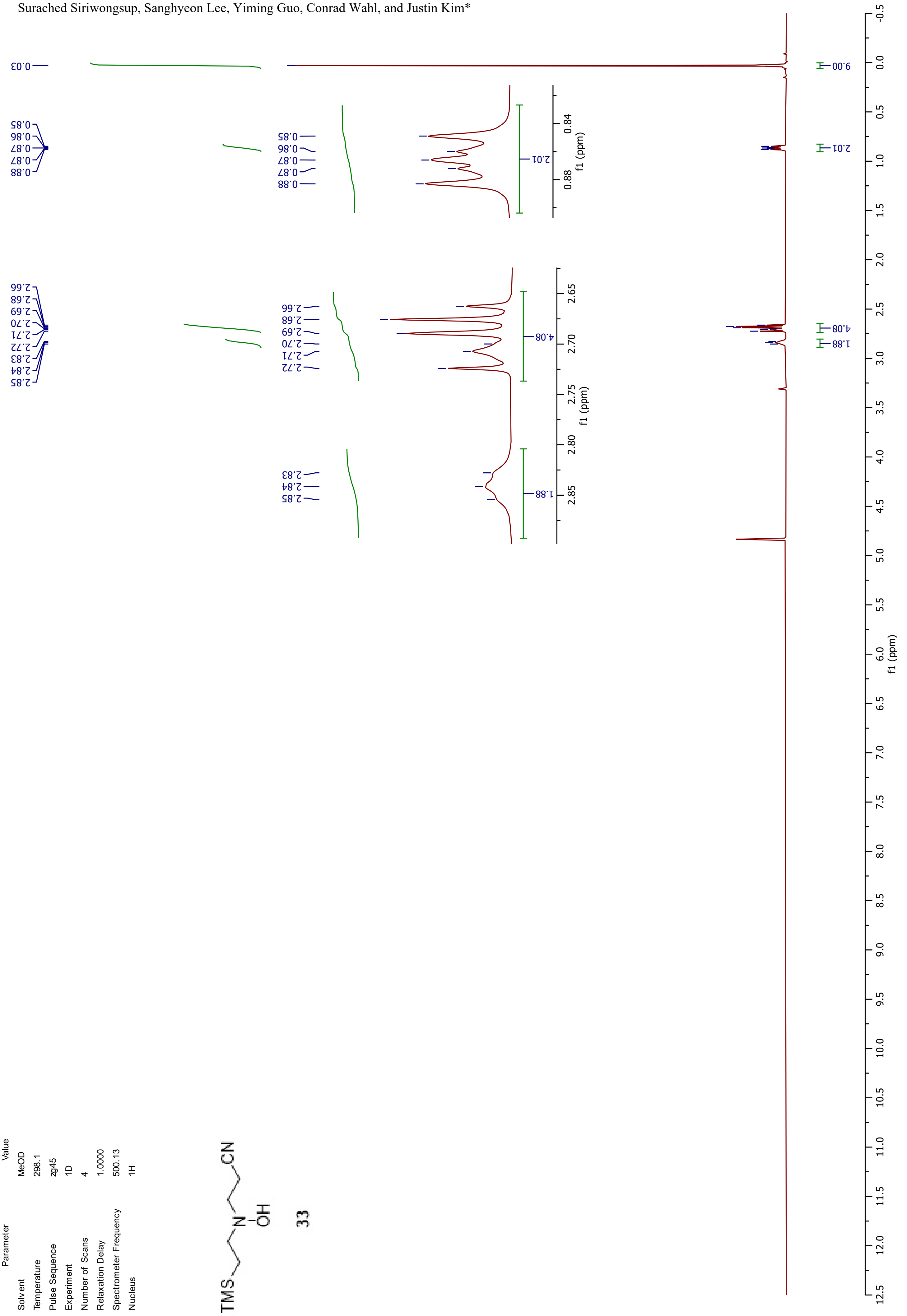
Parameter Value
Solvent MeOD
Temperature 298.1
Pulse Sequence zg45
Experiment 1D
Number of Scans 4
Relaxation Delay 1.0000
Spectrometer Frequency 500.13
Nucleus 1H
TMS
N
OH
CN
33
f1 (ppm)
9.00
2.01
4.08
1.88

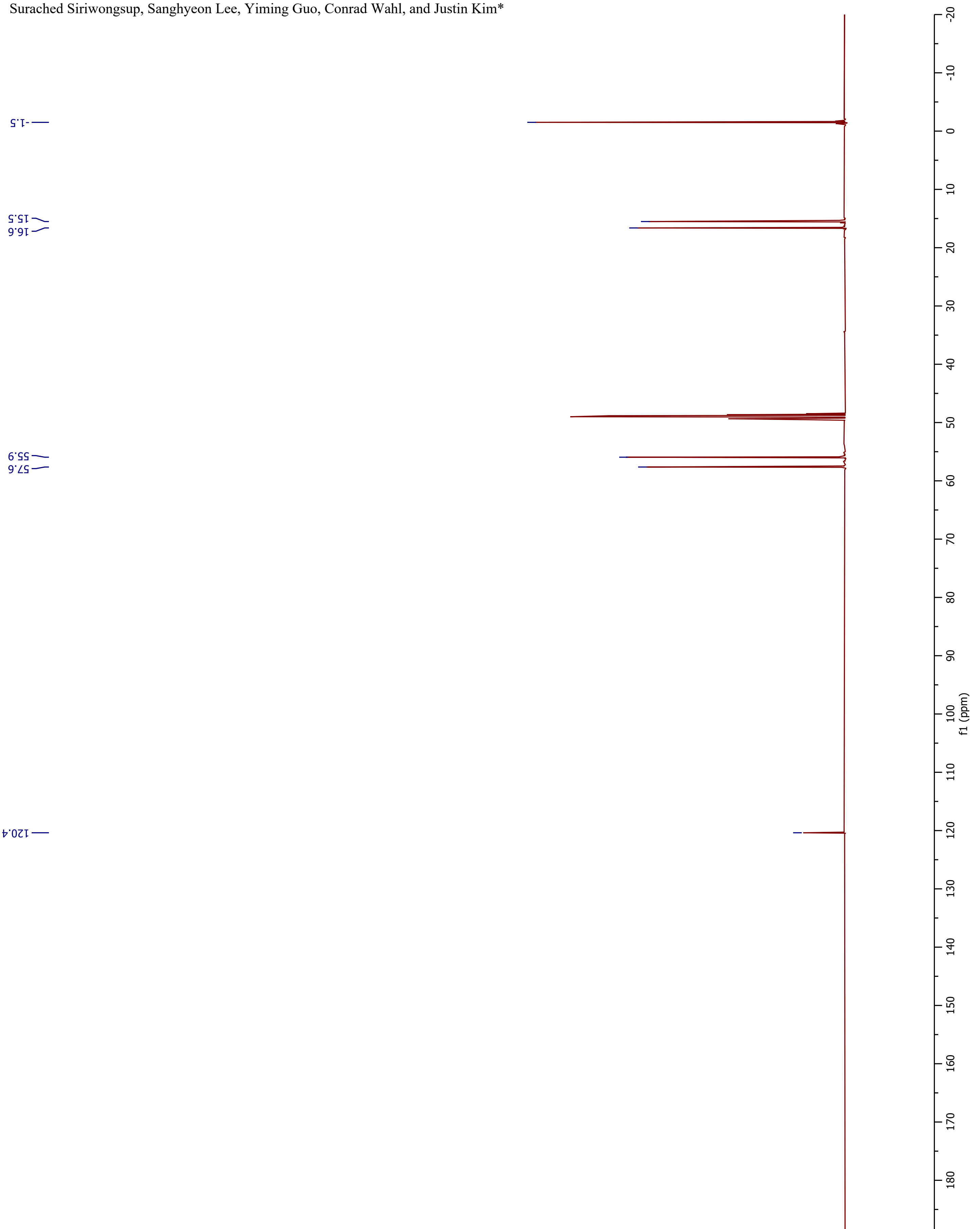

Parameter	Value
Solvent	MeOD
Temperature	298.1
Pulse Sequence	zgpg45
Experiment	1D
Number of Scans	4096
Relaxation Delay	0.3000
Spectrometer Frequency	125.77
Nucleus	13C

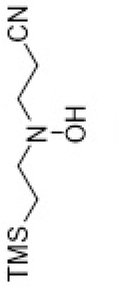

33

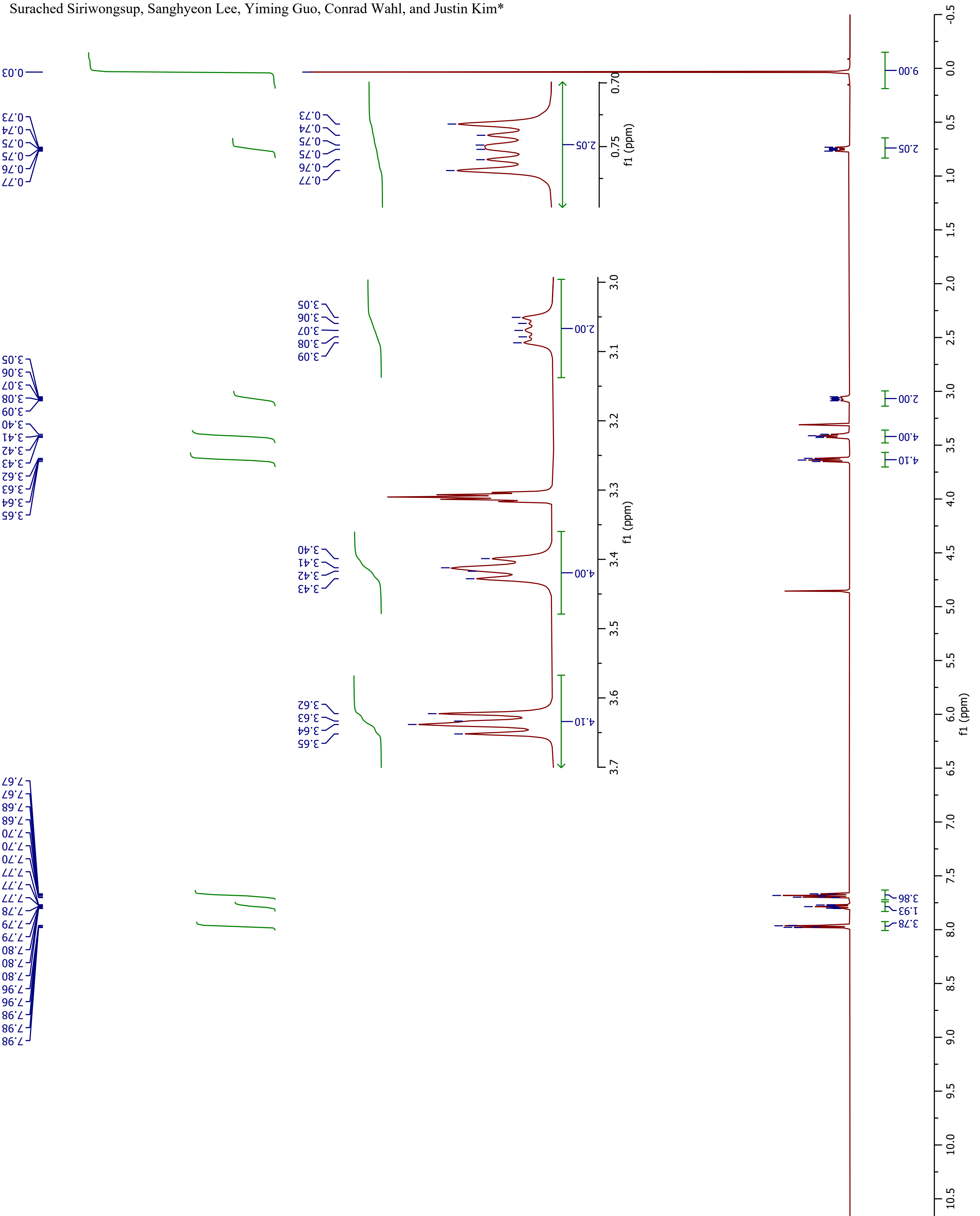

Parameter	Value
Solvent	MeOD
Temperature	298.2
Pulse Sequence	zg45
Experiment	1D
Number of Scans	4
Relaxation Delay	1.0000
Spectrometer Frequency	500.13
Nucleus	1H

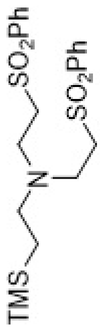

S7

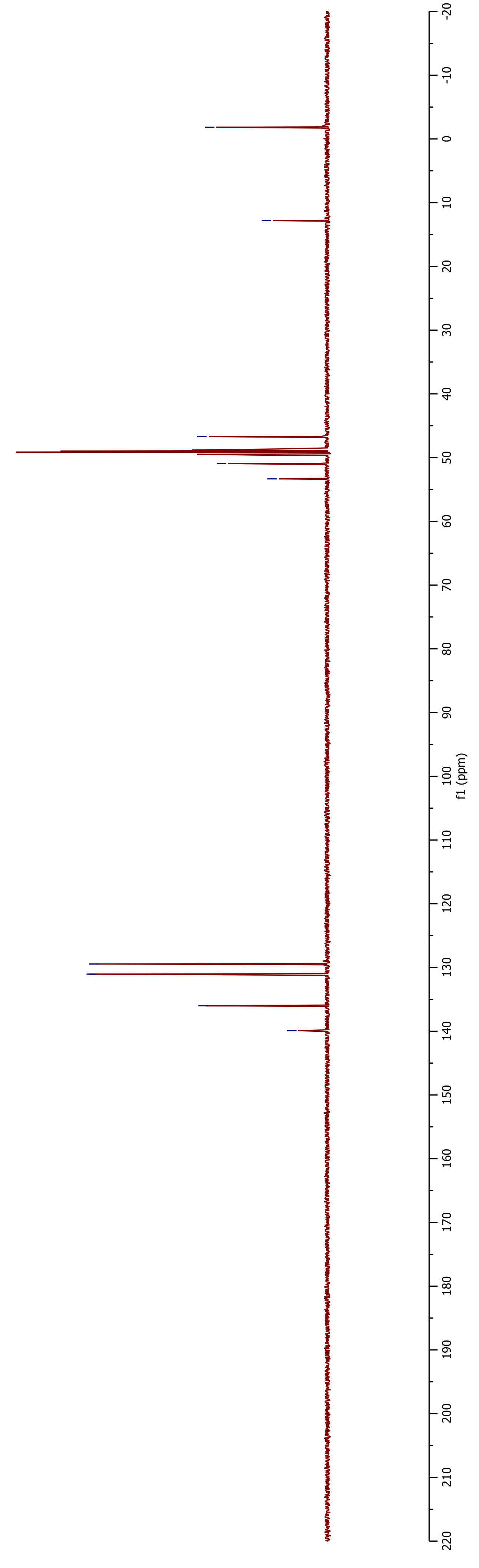

Parameter	Value
Solvent	MeOD
Temperature	298.2
Pulse Sequence	zgpg45
Experiment	1D
Number of Scans	256
Relaxation Delay	0.3000
Spectrometer Frequency	125.77
Nucleus	13C

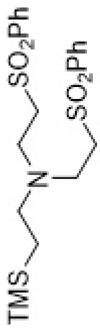

S7

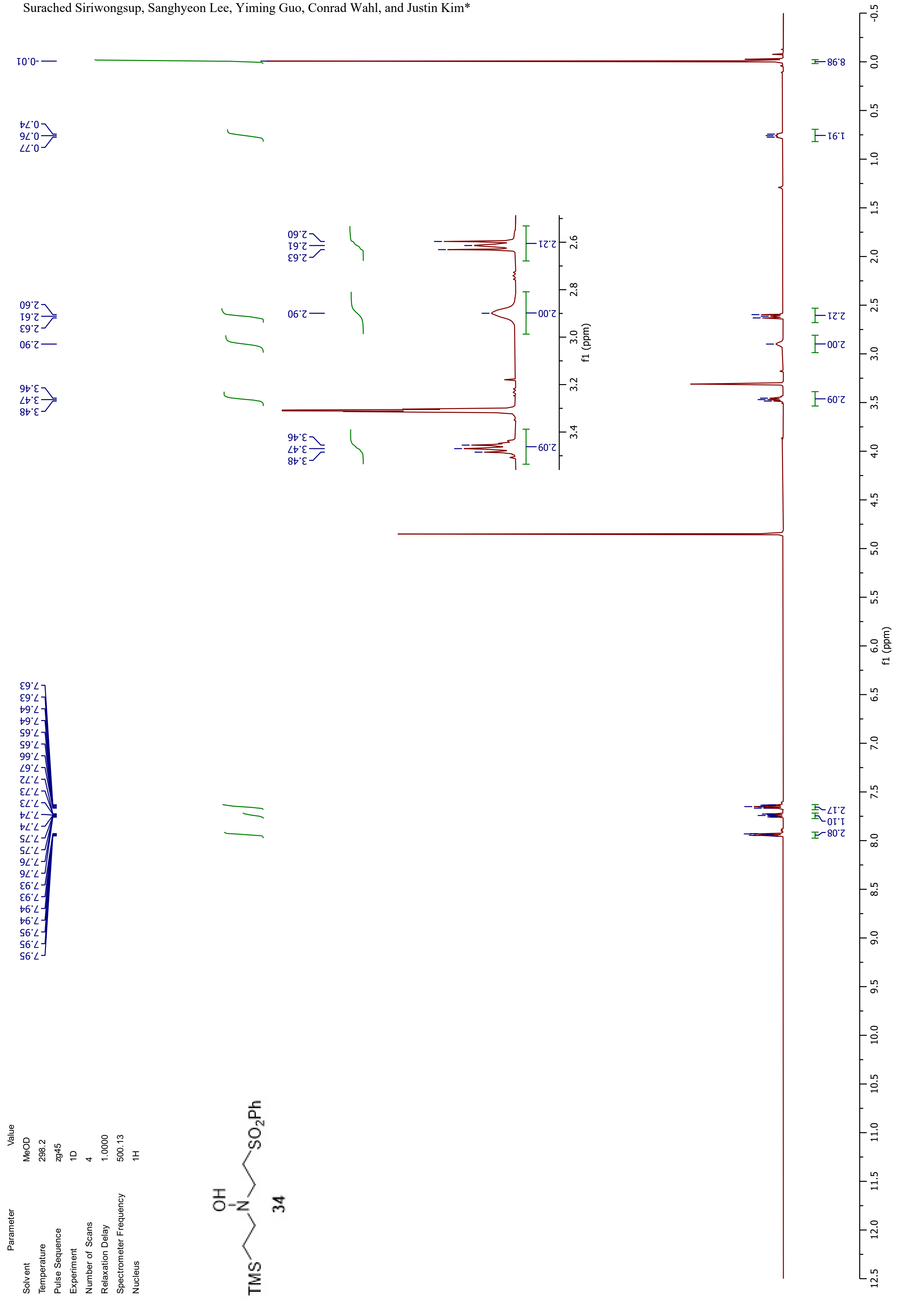
Parameter
Value
Solvent
MeOD
Temperature
298.2
Pulse Sequence
zg45
Experiment
1D
Number of Scans
4
Relaxation Delay
1.0000
Spectrometer Frequency
500.13
Nucleus
1H
34
f1 (ppm)

Surached Siriwongsup, Sanghyeon Lee, Yiming Guo, Conrad Wahl, and Justin Kim*

Parameter	Value
Solvent	MeOD
Temperature	298.1
Pulse Sequence	zgpg45
Experiment	1D
Number of Scans	512
Relaxation Delay	0.3000
Spectrometer Frequency	125.77
Nucleus	13C

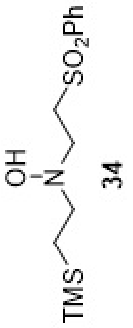

34

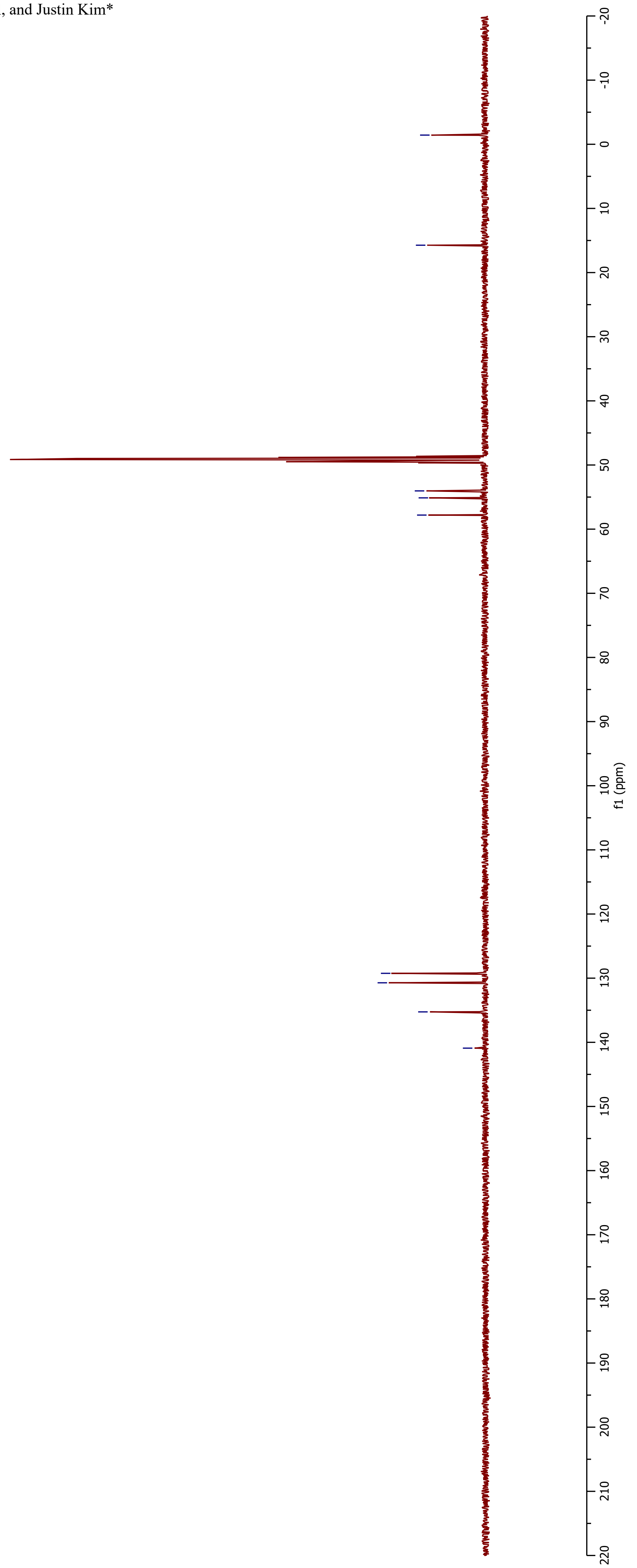

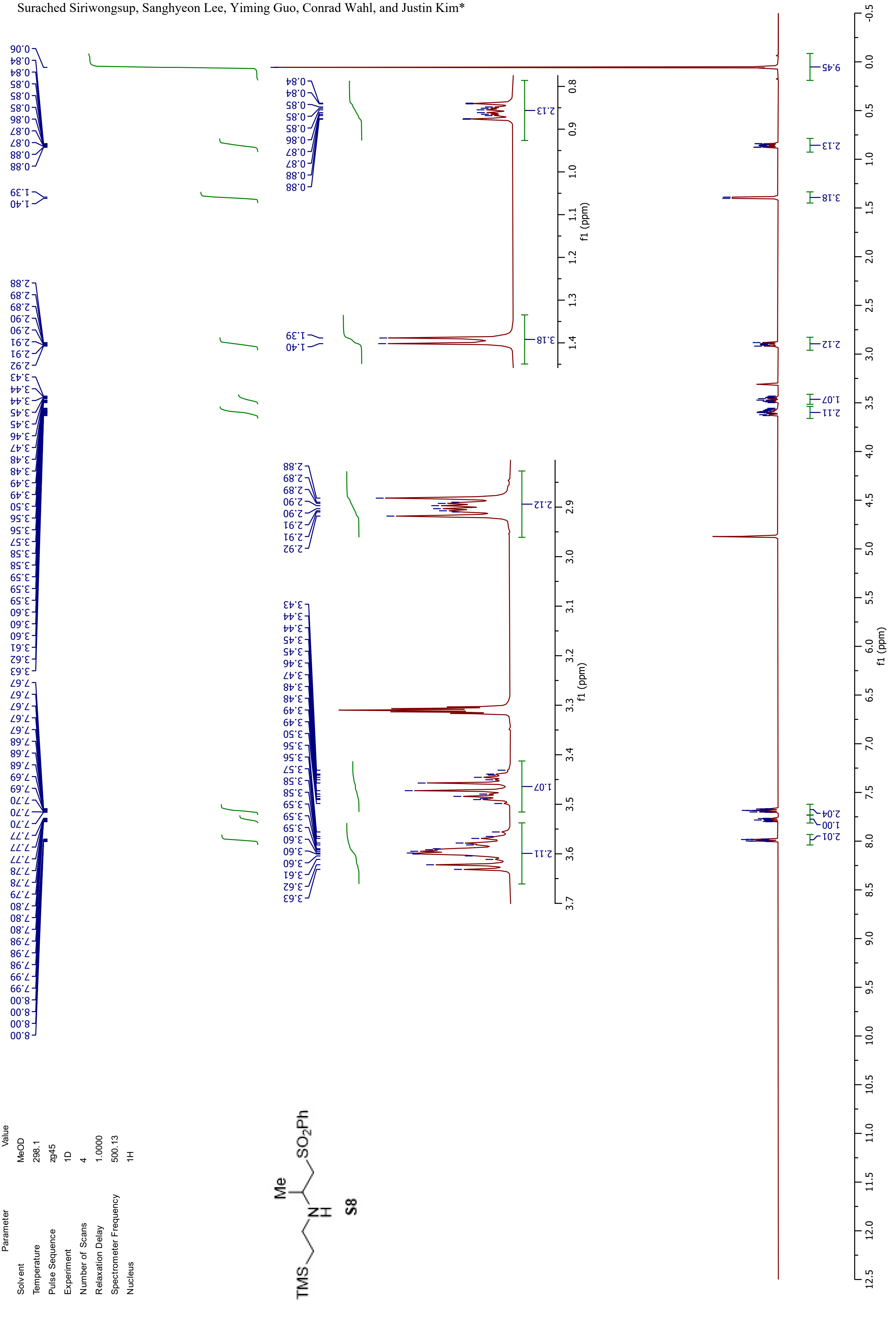
Parameter | Value
Solvent | MeOD
Temperature | 298.1
Pulse Sequence | zg45
Experiment | 1D
Number of Scans | 4
Relaxation Delay | 1.0000
Spectrometer Frequency | 500.13
Nucleus | 1H
S8
f1 (ppm)

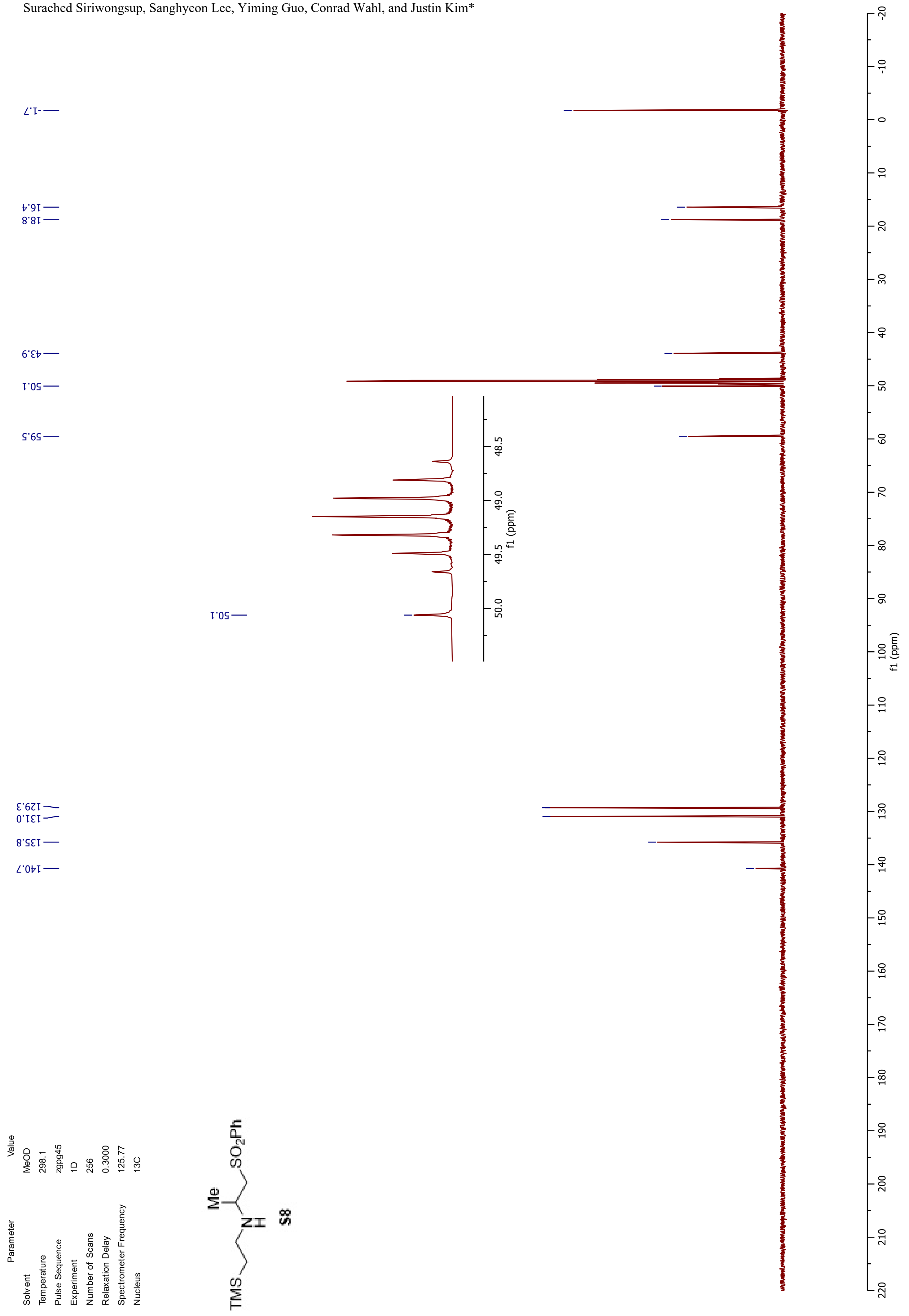
-1.7
16.4
18.8
43.9
50.1
59.5
129.3
131.0
135.8
140.7
50.1
48.5
49.0
49.5
50.0
f1 (ppm)
f1 (ppm)
Parameter
Value
Solvent
MeOD
Temperature
298.1
Pulse Sequence
zgpg45
Experiment
1D
Number of Scans
256
Relaxation Delay
0.3000
Spectrometer Frequency
125.77
Nucleus
13C
TMS
Me
N
H
SO2Ph
S8

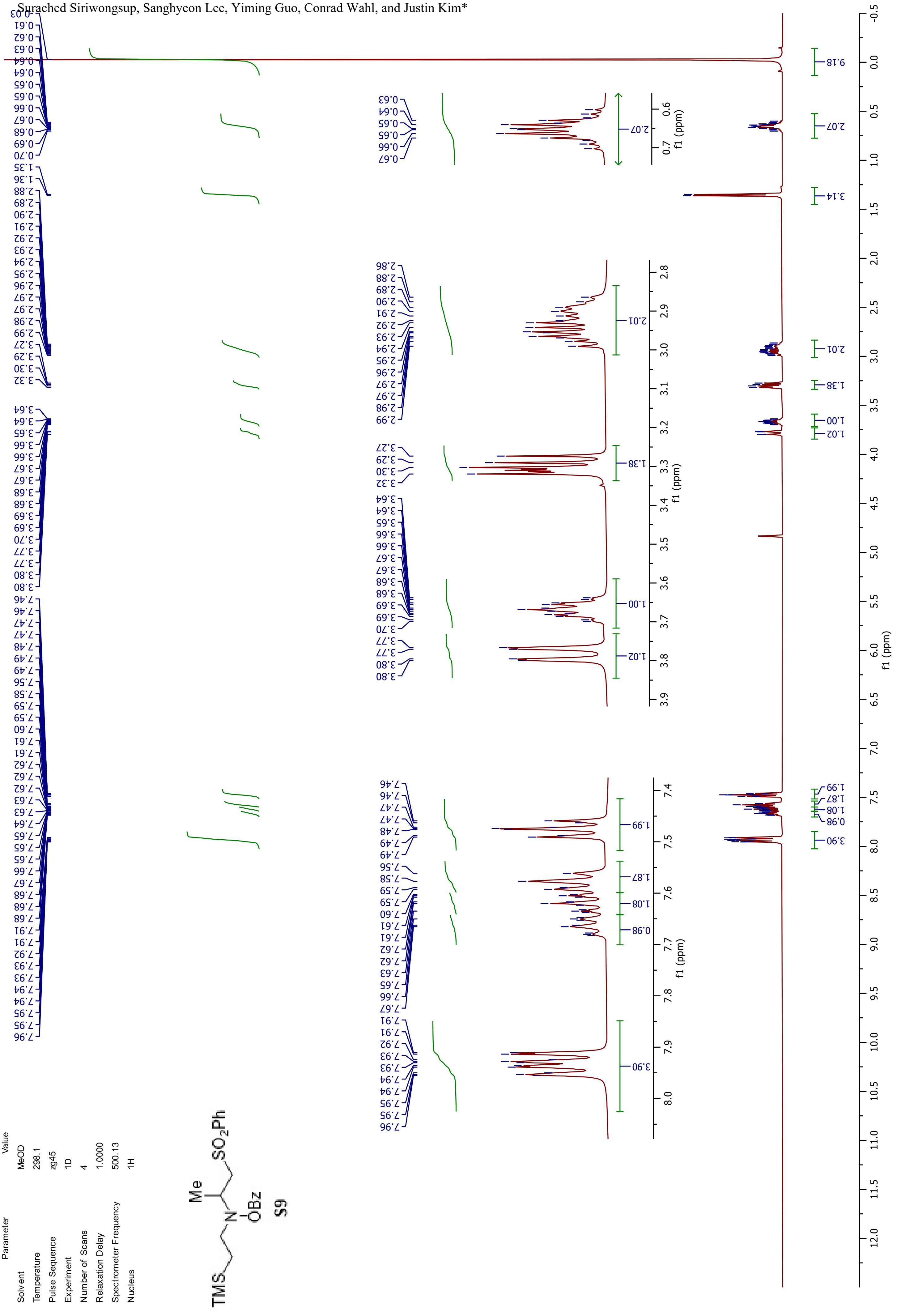
Parameter | Value
Solvent | MeOD
Temperature | 298.1
Pulse Sequence | zg45
Experiment | 1D
Number of Scans | 4
Relaxation Delay | 1.0000
Spectrometer Frequency | 500.13
Nucleus | 1H
S9
f1 (ppm)

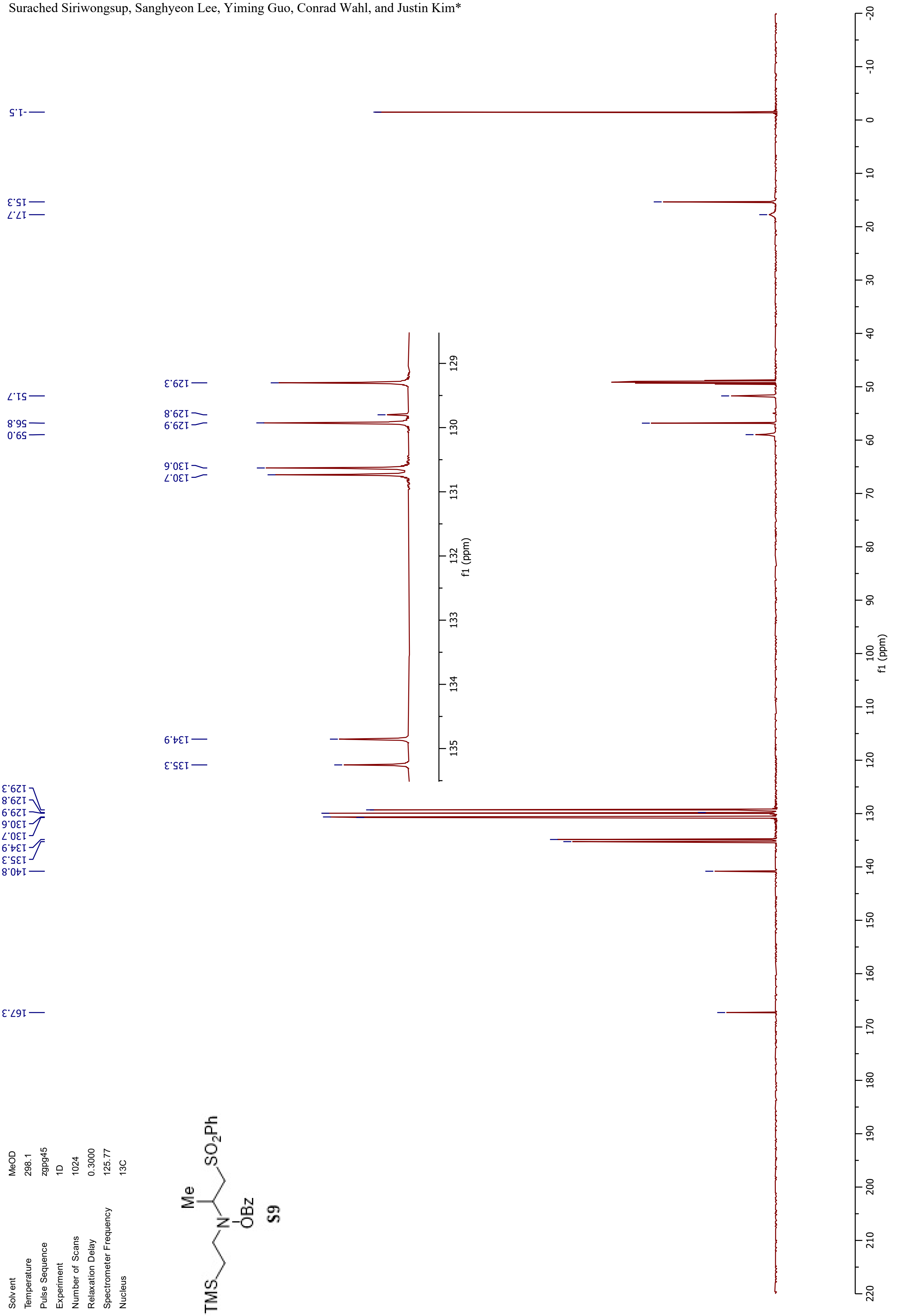

Parameter	Value
Solvent	MeOD
Temperature	298.1
Pulse Sequence	zgpg45
Experiment	1D
Number of Scans	1024
Relaxation Delay	0.3000
Spectrometer Frequency	125.77
Nucleus	13C

S9

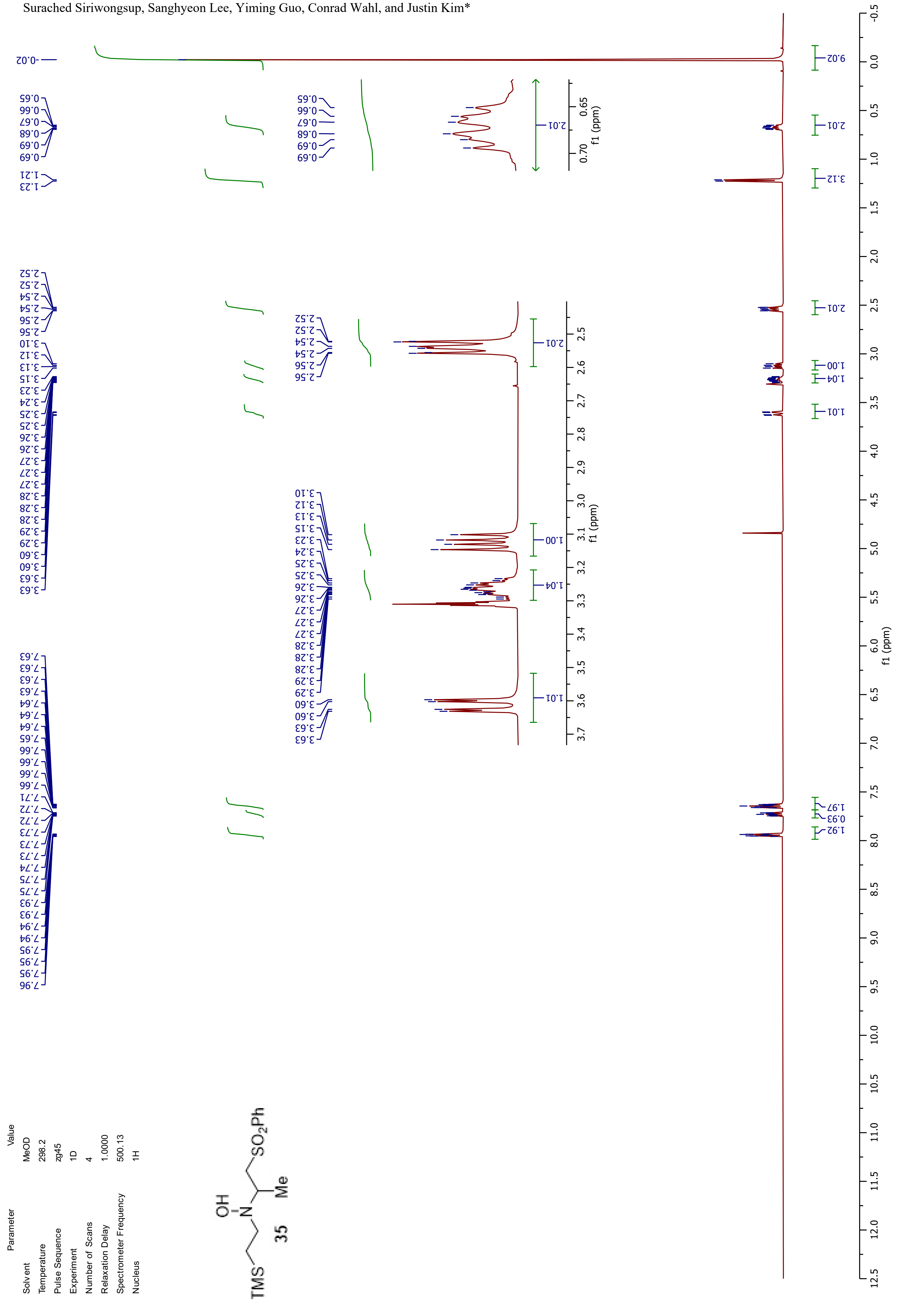
Parameter
Value
Solvent
MeOD
Temperature
298.2
Pulse Sequence
zg45
Experiment
1D
Number of Scans
4
Relaxation Delay
1.0000
Spectrometer Frequency
500.13
Nucleus
1H
TMS
OH
N
SO2Ph
Me
35
f1 (ppm)

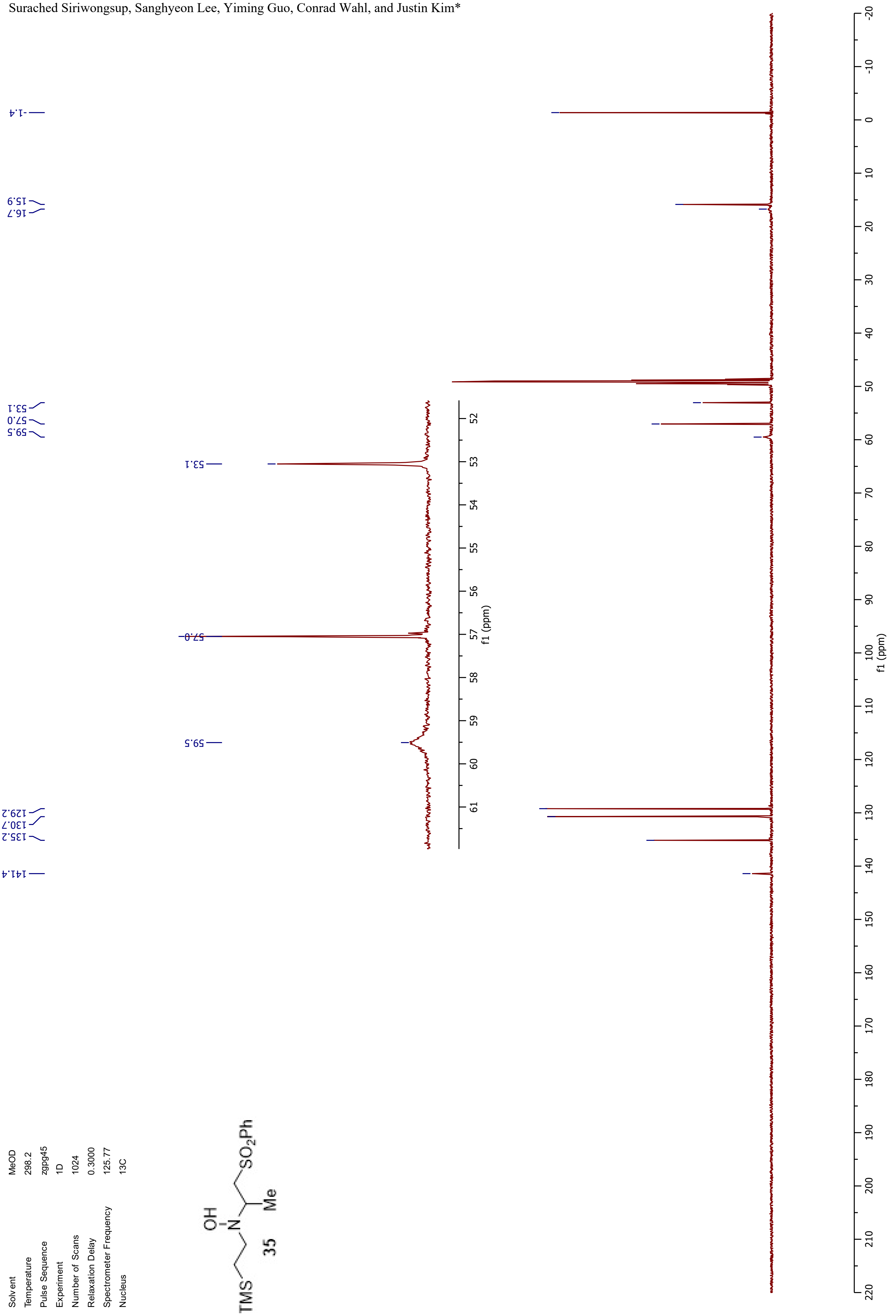

Parameter	Value
Solvent	MeOD
Temperature	298.2
Pulse Sequence	zgpg45
Experiment	1D
Number of Scans	1024
Relaxation Delay	0.3000
Spectrometer Frequency	125.77
Nucleus	13C

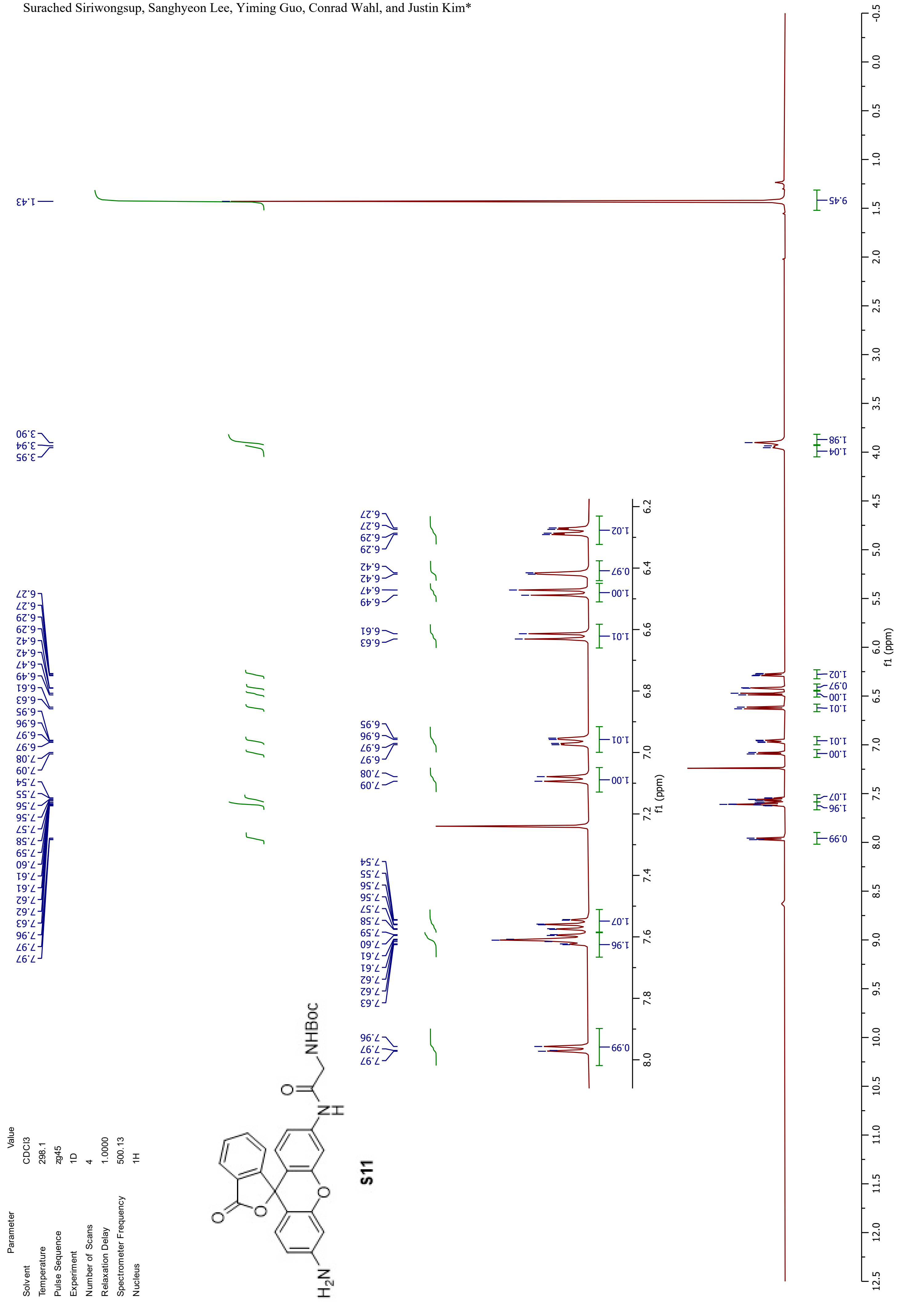
Parameter
Value
Solvent
CDCl3
Temperature
298.1
Pulse Sequence
zg45
Experiment
1D
Number of Scans
4
Relaxation Delay
1.0000
Spectrometer Frequency
500.13
Nucleus
1H
H2N
NHBoc
S11
f1 (ppm)

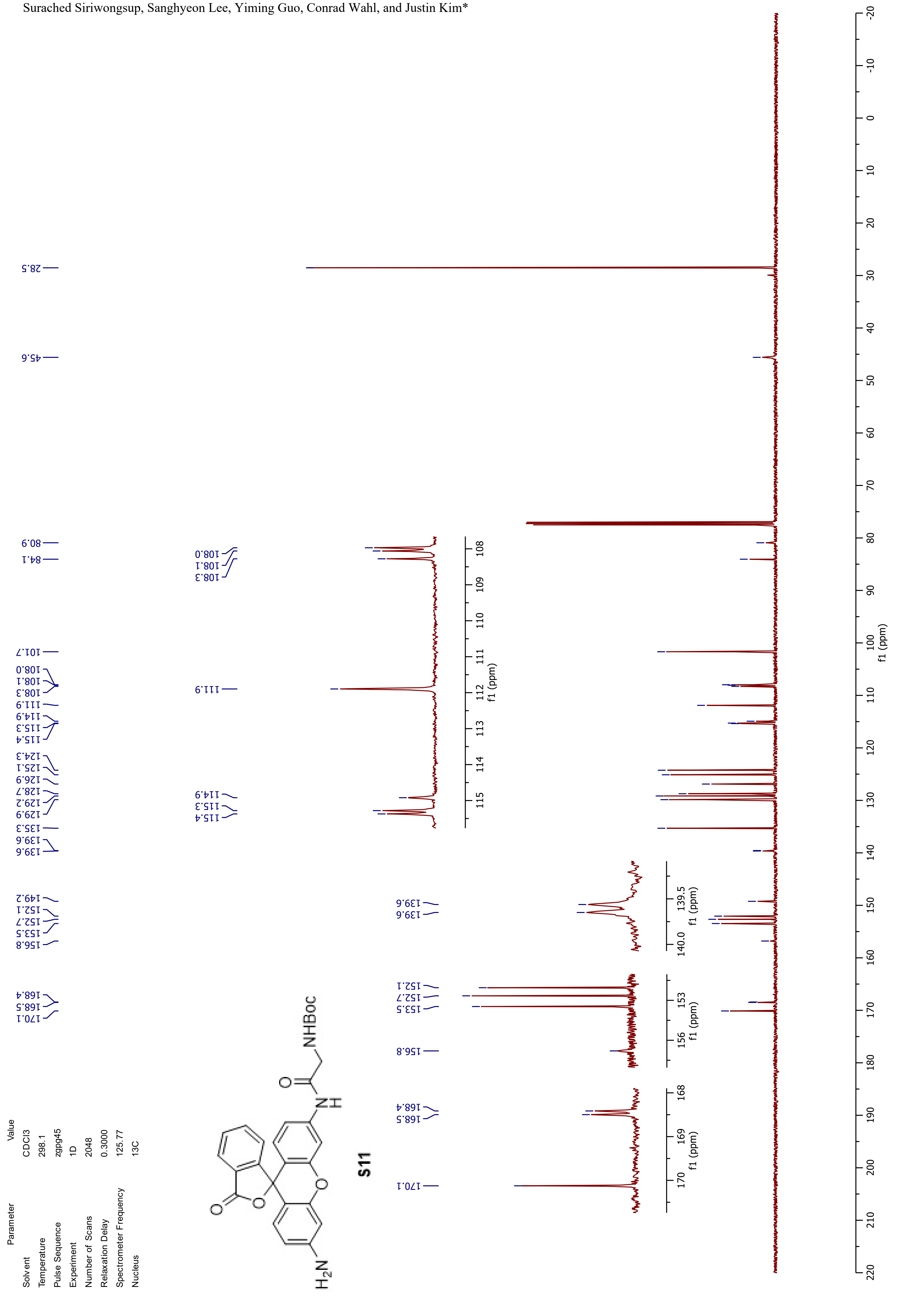
Parameter Value
Solvent CDCl3
Temperature 298.1
Pulse Sequence zgpg45
Experiment 1D
Number of Scans 2048
Relaxation Delay 0.3000
Spectrometer Frequency 125.77
Nucleus 13C
S11
28.5
45.6
80.9
84.1
101.7
108.0
108.1
108.3
111.9
114.9
115.3
115.4
124.3
125.1
126.9
128.7
129.2
129.9
135.3
139.6
139.6
149.2
152.1
152.7
153.5
156.8
168.4
168.5
170.1
f1 (ppm)

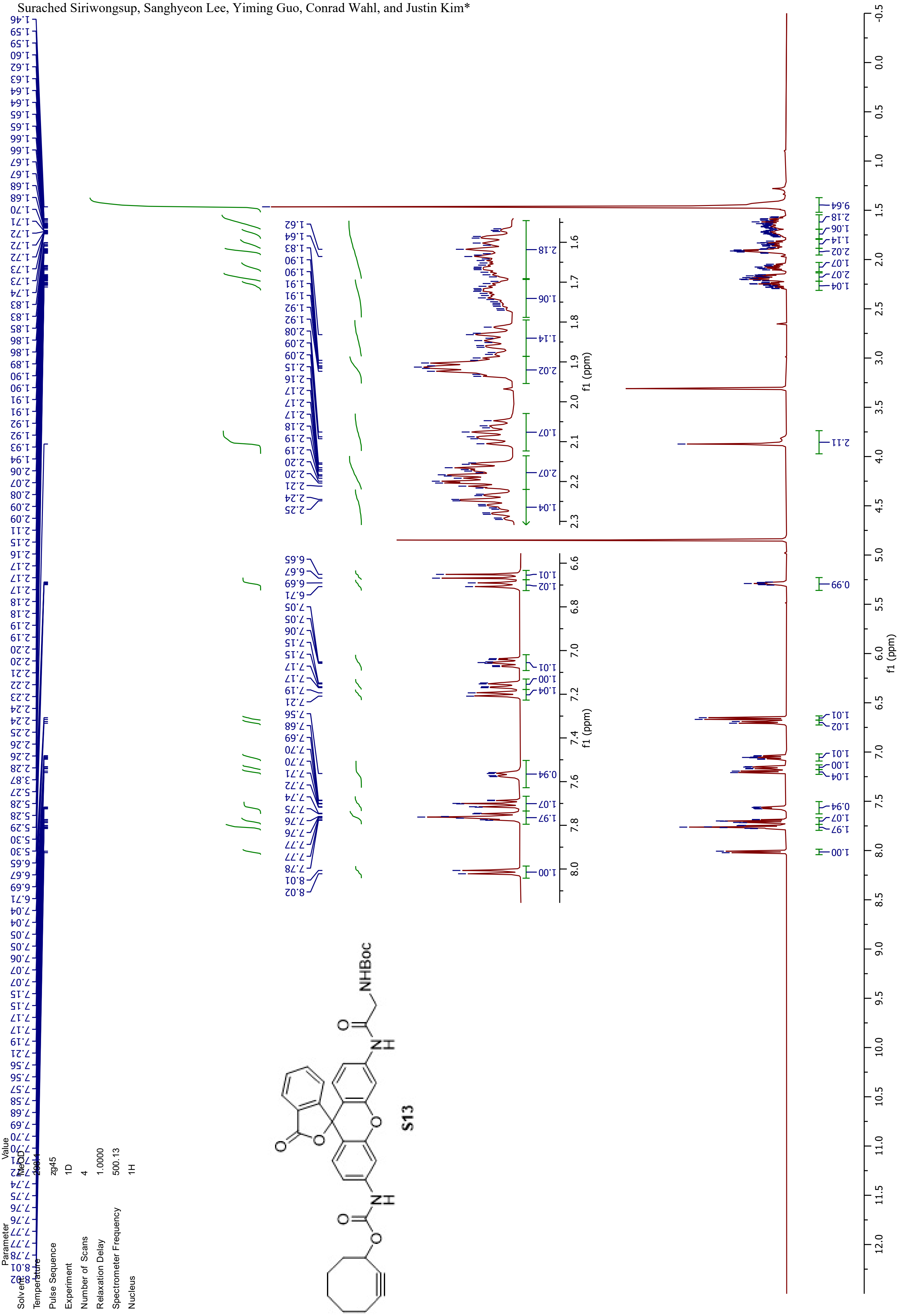

S13

Parameter	Value
Solvent	MeOD
Temperature	[illegible]
Pulse Sequence	zg45
Experiment	1D
Number of Scans	4
Relaxation Delay	1.0000
Spectrometer Frequency	500.13
Nucleus	1H

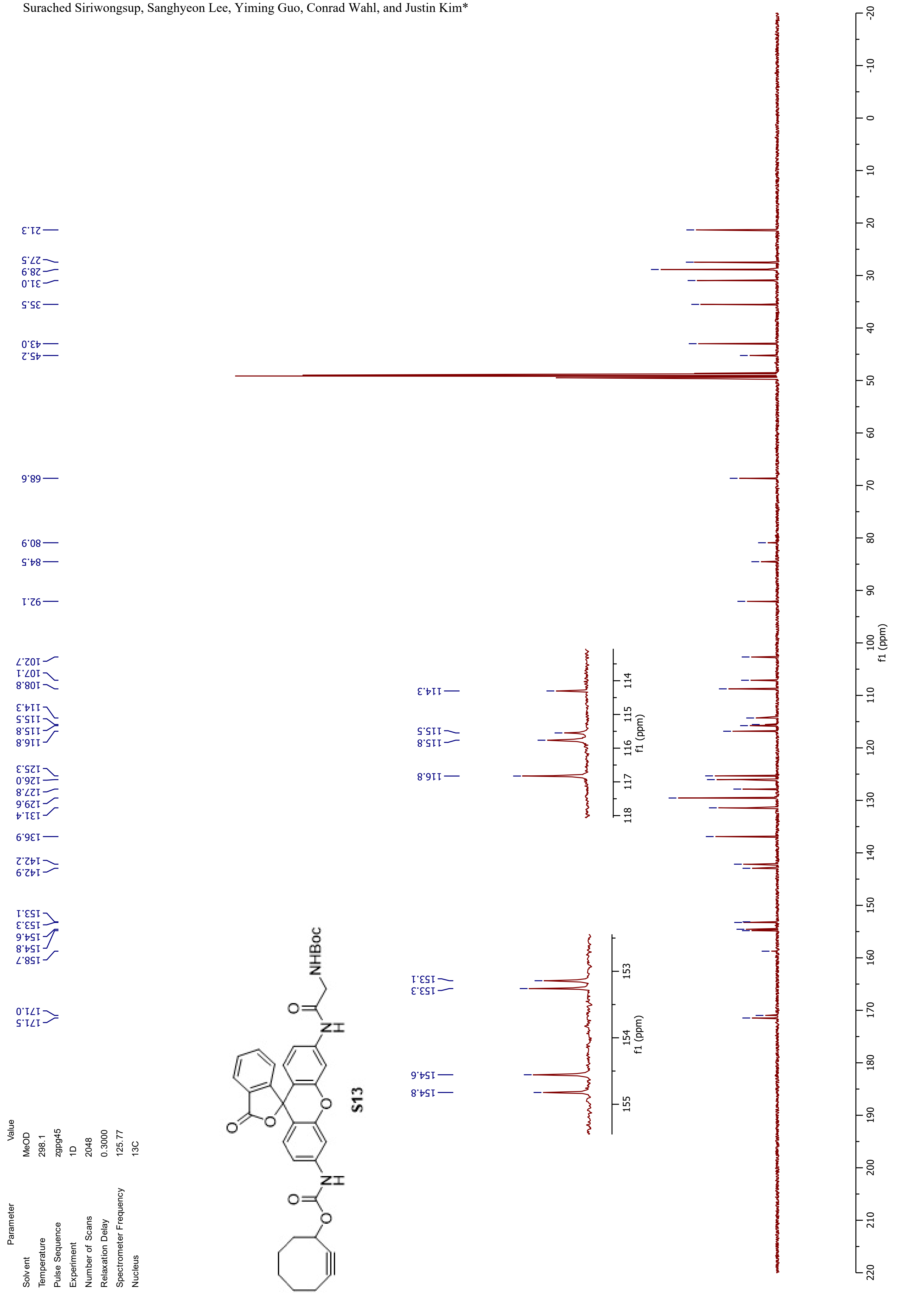
Parameter
Value
Solvent
MeOD
Temperature
298.1
Pulse Sequence
zgpg45
Experiment
1D
Number of Scans
2048
Relaxation Delay
0.3000
Spectrometer Frequency
125.77
Nucleus
13C
NHBoc
S13
21.3
27.5
28.9
31.0
35.5
43.0
45.2
68.6
80.9
84.5
92.1
102.7
107.1
108.8
114.3
115.5
115.8
116.8
125.3
126.0
127.8
129.6
131.4
136.9
142.2
142.9
153.1
153.3
154.6
154.8
158.7
171.0
171.5
f1 (ppm)

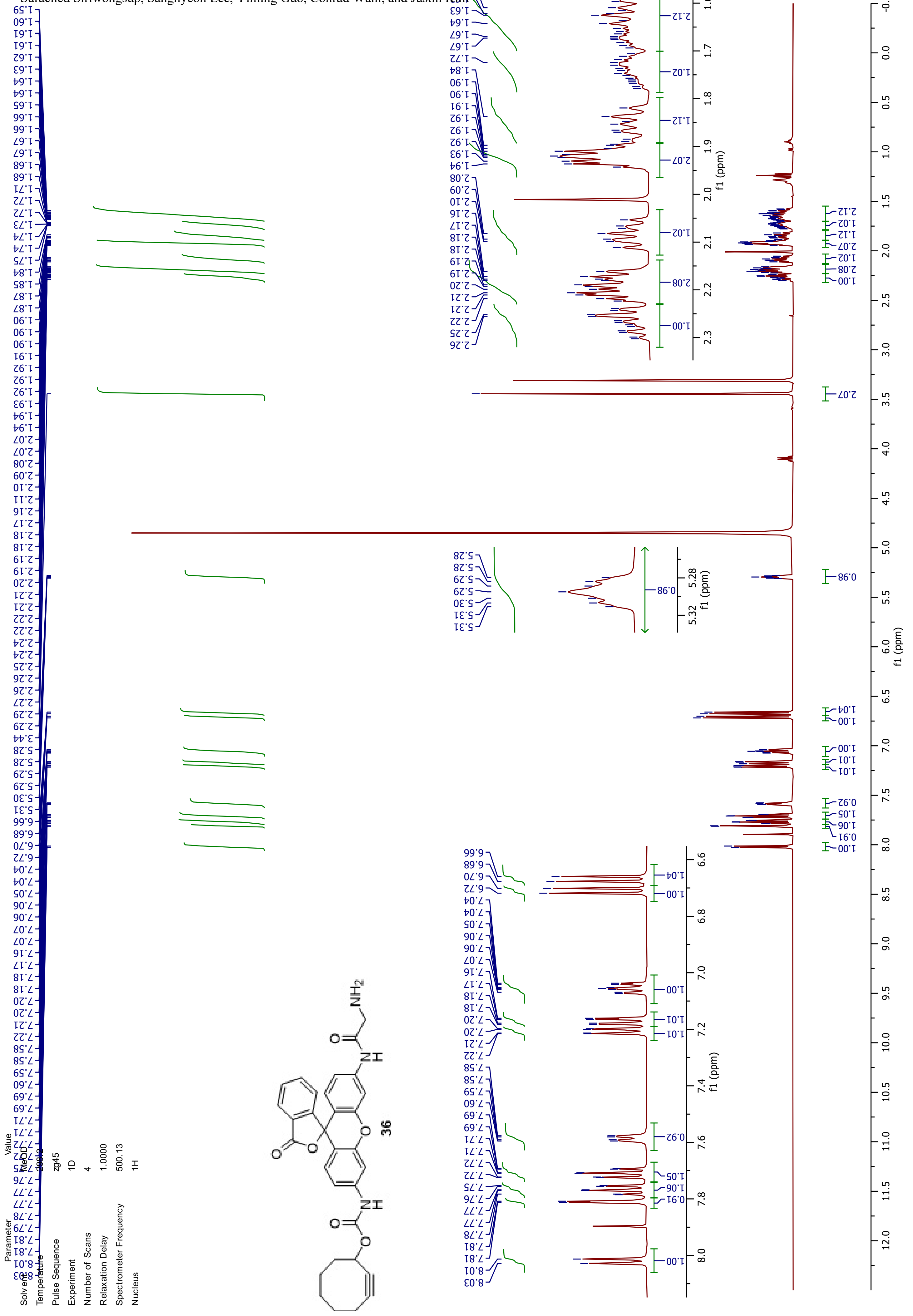
Parameter
Value
Solvent
MeOD
Temperature
Pulse Sequence
zg45
Experiment
1D
Number of Scans
4
Relaxation Delay
1.0000
Spectrometer Frequency
500.13
Nucleus
1H
36
f1 (ppm)

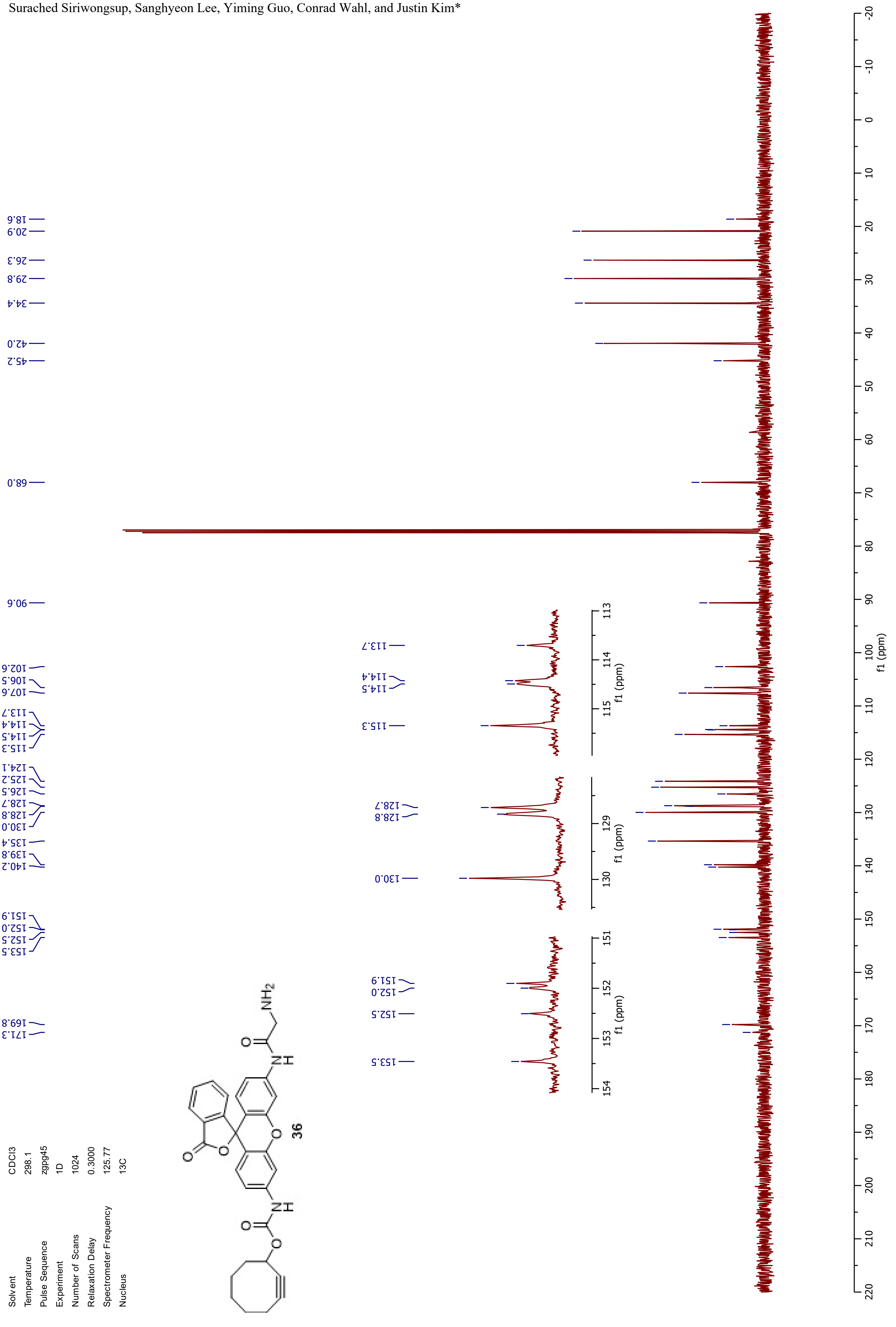

Parameter	Value
Solvent	CDCl3
Temperature	298.1
Pulse Sequence	zgpg45
Experiment	1D
Number of Scans	1024
Relaxation Delay	0.3000
Spectrometer Frequency	125.77
Nucleus	13C

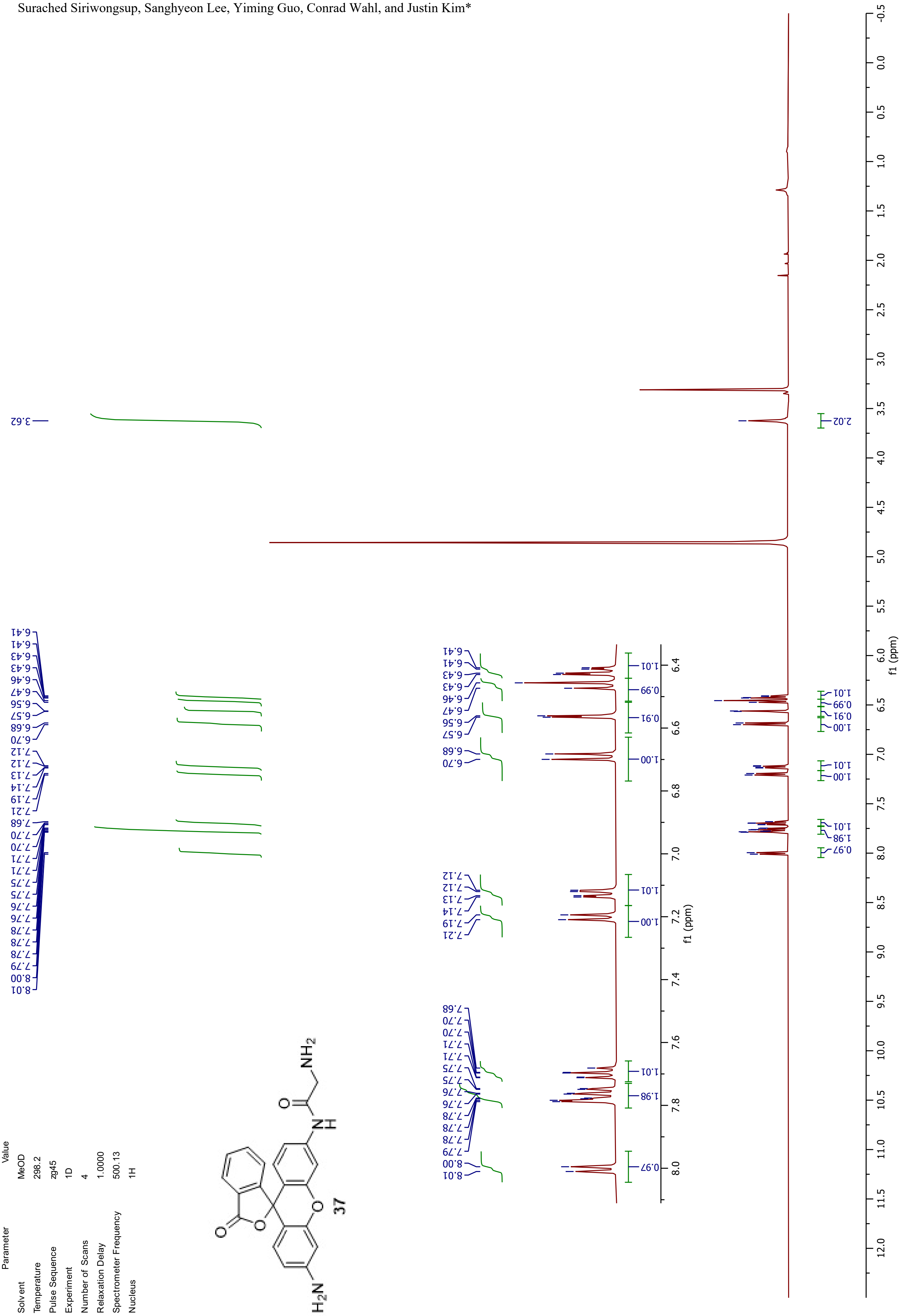

Parameter	Value
Solvent	MeOD
Temperature	298.2
Pulse Sequence	zg45
Experiment	1D
Number of Scans	4
Relaxation Delay	1.0000
Spectrometer Frequency	500.13
Nucleus	1H

Parameter	Value
Solvent	MeOD
Temperature	298.1
Pulse Sequence	zgpg45
Experiment	1D
Number of Scans	4096
Relaxation Delay	0.3000
Spectrometer Frequency	125.77
Nucleus	13C

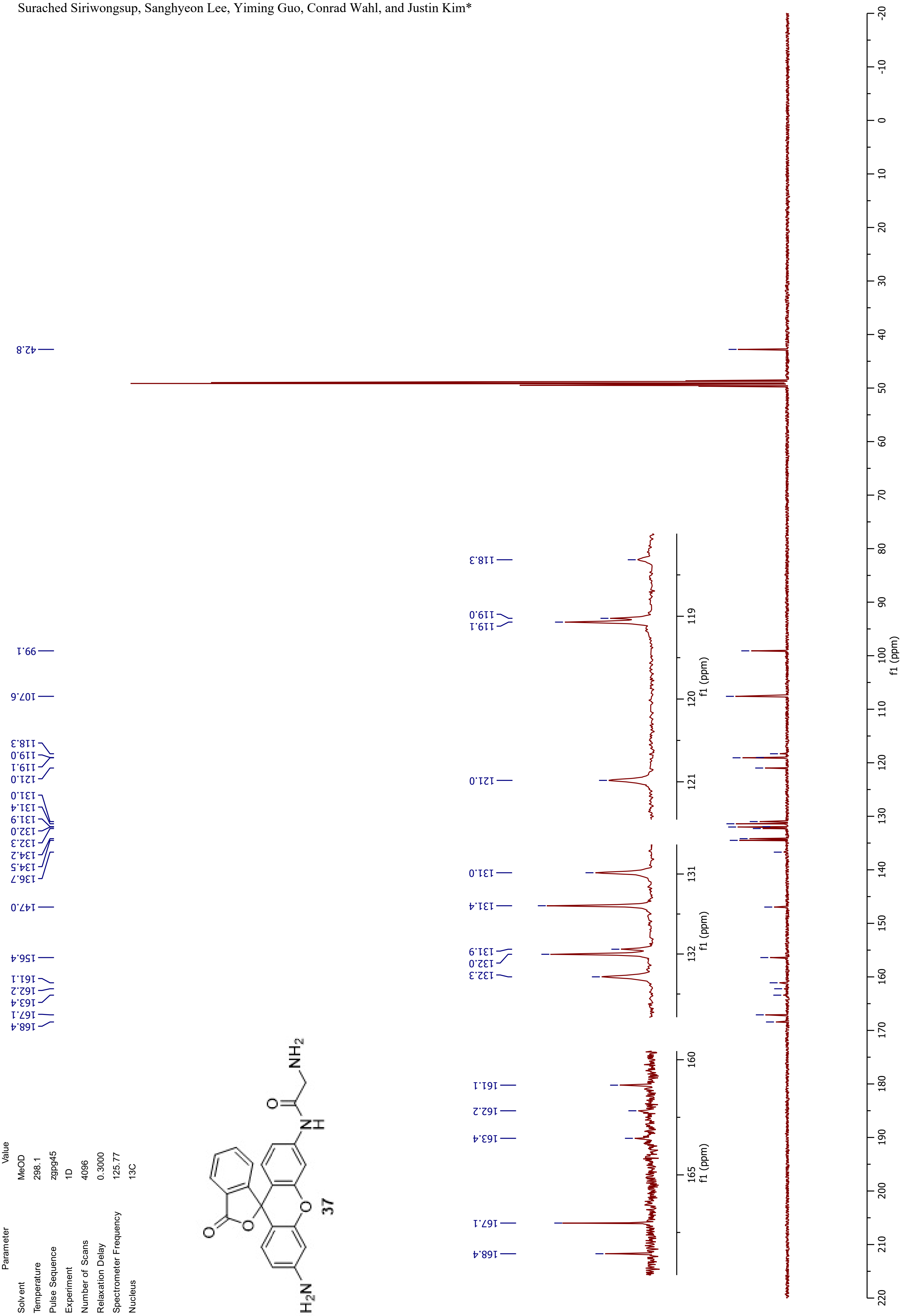

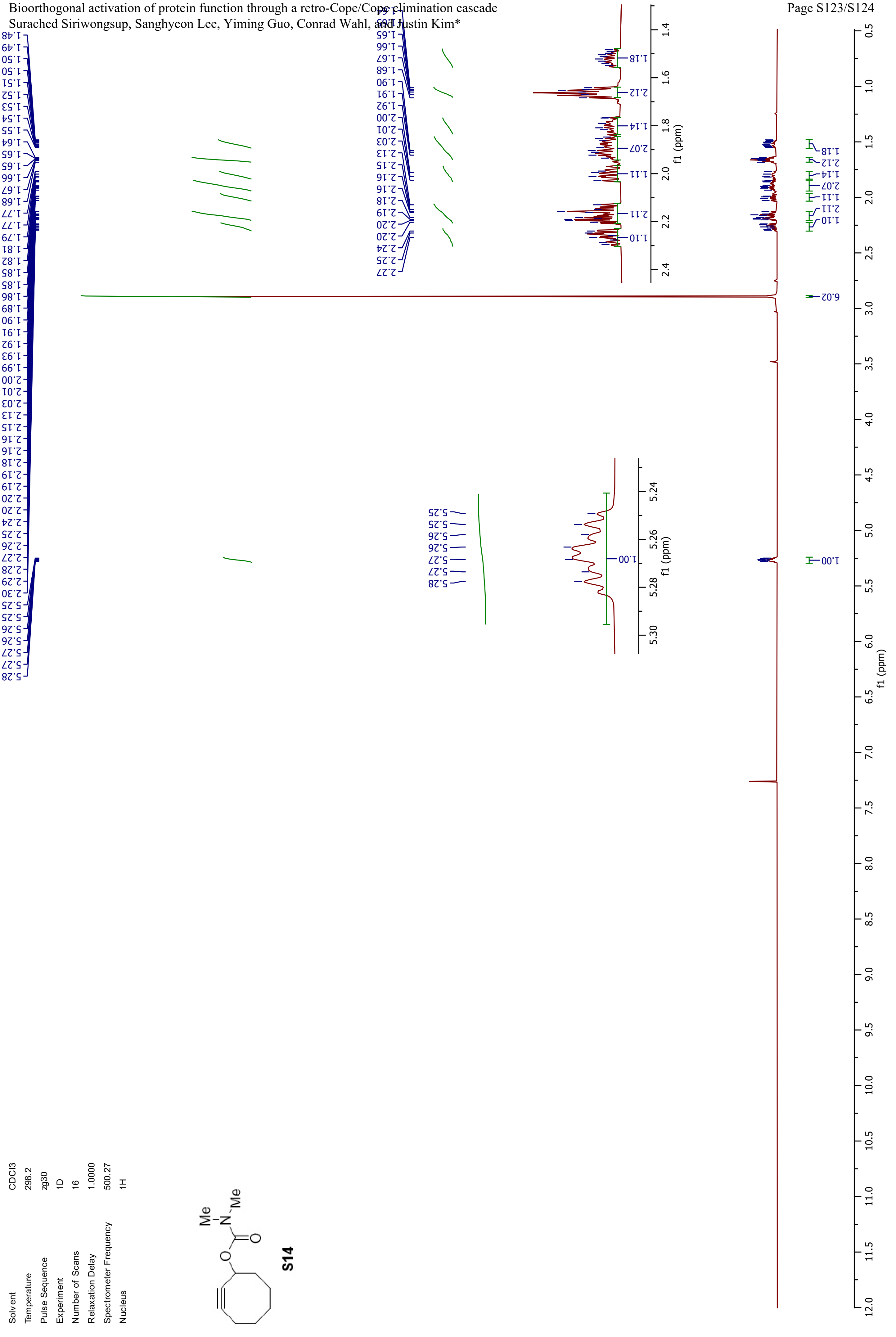

S14

Parameter	Value
Solvent	CDCl3
Temperature	298.2
Pulse Sequence	zg30
Experiment	1D
Number of Scans	16
Relaxation Delay	1.0000
Spectrometer Frequency	500.27
Nucleus	1H

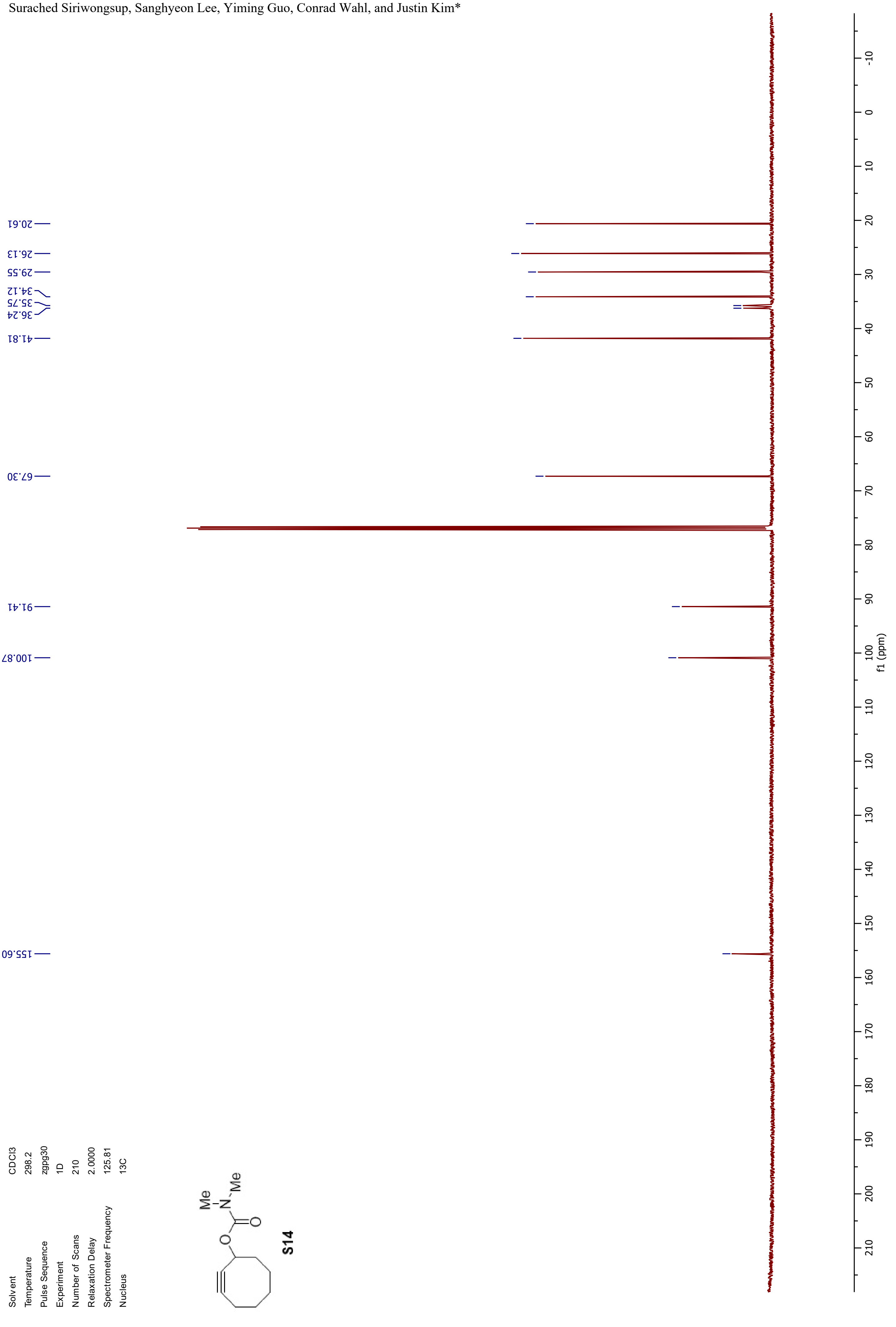

Parameter	Value
Solvent	CDCl3
Temperature	298.2
Pulse Sequence	zgpg30
Experiment	1D
Number of Scans	210
Relaxation Delay	2.0000
Spectrometer Frequency	125.81
Nucleus	13C
